# The reaction specificity of mammalian ALOX15B orthologs does not depend on the evolutionary ranking of the animals

**DOI:** 10.1016/j.jlr.2025.100768

**Published:** 2025-03-03

**Authors:** Eda Gündem, Sabine Stehling, Astrid Borchert, Hartmut Kuhn

**Affiliations:** Charité – Universitätsmedizin Berlin, Corporate Member of Freie Universität Berlin and Humboldt Universität zu Berlin, Department of Biochemistry, Berlin, Germany

**Keywords:** eicosanoids, lipid peroxidation, oxidative stress, enzyme evolution, ferroptosis

## Abstract

Arachidonic acid lipoxygenases (ALOXs) play important roles in cell differentiation and in the pathogenesis of cardiovascular, hyperproliferative, neurodegenerative, and metabolic diseases. The human genome involves six intact *ALOX* genes and knockout studies of the corresponding mouse orthologs indicated that the coding multiplicity of ALOX isoforms is not an indication for functional redundancy. Despite their evolutionary relatedness human and mouse ALOX15 and ALOX15B orthologs exhibit different catalytic properties. Human ALOX15 oxygenates arachidonic acid mainly to 15S-hydroperoxy-5Z,8Z,11Z,13E-eicosatetraenoic acid but 12S-hydroperoxy-5Z,8Z,10E,14Z-eicosatetraenoic acid is the dominant oxygenation product of mouse Alox15. This functional difference is the results of a targeted enzyme evolution but the driving forces for this process have not been well defined. For human and mouse ALOX15B orthologs similar functional differences have been reported but for the time being it was unclear whether these differences might also be a consequence of targeted enzyme evolution. To address this question, we systematically searched the public databases for *ALOX15B* genes, expressed selected enzymes, and characterized their functional properties. We found that functional *ALOX15B* genes frequently occur in *Prototheria* and *Eutheria* but orthologous genes are rare in *Metatheria*. The vast majority of mammalian ALOX15B orthologs constitute arachidonic acid 15-lipoxygenating enzymes and this property did not depend on the evolutionary ranking of the animals. Only several *Muridae* species including *M. musculus*, *M. pahari*, *M. caroli*, *M. coucha, and A. niloticus* express arachidonic acid 8-lipoxygenating ALOX15B orthologs. Consequently, the difference in the reaction specificity of mouse and human ALOX15B orthologs may not be considered a functional consequence of targeted enzyme evolution.

## Graphical Abstract

**Figure:**
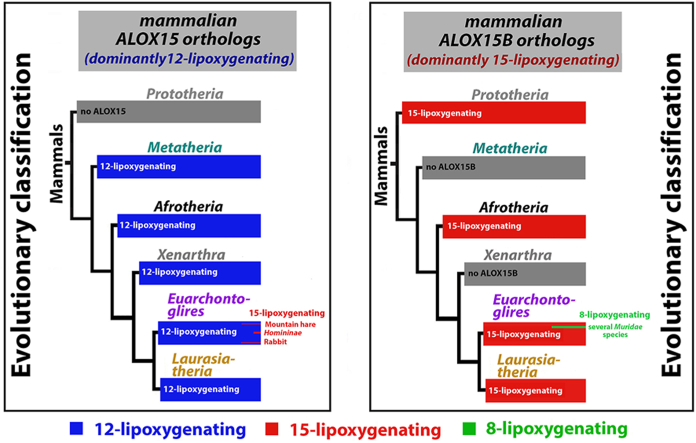

Arachidonic acid lipoxygenases (ALOX isoforms) catalyze the oxygenation of polyunsaturated fatty acids (PUFAs) to hydroperoxides (1, 2), which are subsequently rapidly reduced to the more stable hydroxy derivatives by the catalytic activities of glutathione peroxidases (3, 4). In the human reference genome six functional *ALOX* genes (*ALOX5, ALOXE3, ALOX15, ALOX12, ALOX12B, and ALOX15B*) have been detected and knockout studies of the murine orthologs indicated that each isoenzyme exhibits isoform-specific functions (5). In other words, the coding multiplicity of these enzymes should not be interpreted as sign of functional redundancy. ALOX5 has been implicated in the biosynthesis of proinflammatory leukotrienes (6) and leukotriene receptor antagonist is currently prescribed to patients suffering from bronchial asthma (7). In contrast, ALOX12B and ALOXE3 are not capable of synthesizing leukotrienes but play important roles in epidermal differentiation and skin development (8, 9). Mammalian ALOX15 orthologs have been implicated in cell differentiation (10, 11, 12) but these enzymes might also play a role in inflammatory resolution (13, 14). The biological functions of mammalian ALOX15B orthologs (15, 16) are not well defined (17, 18) but lentiviral shRNA-based expression silencing has implicated this enzyme in atherogenesis (19).

According to the conventional concept of the arachidonic acid cascade ALOX isoforms exhibit their biological activities via the biosynthesis of bioactive lipid mediators (20). The chemical structures of these signaling molecules strongly depend on the reaction specificity of the enzymes. Arachidonic acid (AA) 5-lipoxygenating ALOX isoforms convert arachidonic acid to 5-HpETE, which is further metabolized to proinflammatory leukotrienes (6). In contrast, AA 12-lipoxygenating ALOX isoforms oxygenate the same substrate to 12S-hydroperoxy-5Z,8Z,10E,14Z-eicosatetraenoic acid (HpETE) but this intermediate cannot be further converted to leukotrienes. However, 12-HpETE may serve as substrate for the biosynthesis of hepoxilins and these oxylipins exhibit different biological functions than leukotrienes (21, 22). In other words, for those ALOX isoforms, which exhibit their biological functions via the formation of specific oxylipins, the reaction specificity of the enzymes is of major importance.

For mammalian ALOX15 orthologs, it has recently been shown that the reaction specificity was altered during mammalian evolution (23, 24). Most (>90%) mammals ranked in evolution below gibbons express AA 12-lipoxygenating ALOX15 orthologs and this is also the case for mice (25) and rats (26, 27). On the other hand, mammals ranked in evolution above gibbons including orangutans, gorillas, chimpanzees, as well as extant and extinct human subspecies express AA 15-lipoxygenating ALOX15 orthologs (23, 24). Although the driving forces for this targeted enzyme evolution has not been studied in detail, AA 12-lipoxygenting ALOX15 orthologs exhibit a reduced biosynthetic capacity of proresolving lipoxins (23) and resolvins (24). Thus, the evolutionary change in reaction specificity of mammalian ALOX15 orthologs might be interpreted as part of an optimizing strategy of the mammalian immune system to better control the inflammatory reaction (23, 24).

Similar differences have been reported for the reaction specificities of human and mouse ALOX15B orthologs. Human ALOX15B, which was first described in 1997 (15), constitutes an AA 15-lipoxygenating enzyme that converts AA almost exclusively to 15S-hydroxy-5Z,8Z,11Z,13E-eicosatetraenoic acid (HETE). In contrast, its mouse ortholog, which was originally named mouse 8S-LOX, oxygenates AA to 8S-HpETE (16, 28). Site-directed mutagenesis studies on recombinant human and mouse ALOX15B orthologs indicated that Tyr603Asp+His604Val exchange in mouse Alox15b completely humanized the reaction specificity of the mouse enzyme. The inverse mutagenesis strategy of the corresponding amino acids (Jisaka determinants) in human recombinant ALOX15B (Asp602Tyr+Val603His exchange) partly murinized the reaction specificity (29). In fact, a mixture of 8S-HETE and 15S-HETE was formed from AA by the recombinant human ALOX15B double mutant (29). This mutagenesis strategy does not only work with recombinant ALOX15B preparations but also under in vivo conditions. In fact, PMA stimulated keratinocytes of knock-in mice carrying an Alox15b gene, which encodes for the Tyr603Asp+His604Val double mutant of Alox15b instead of the wild-type gene, convert AA mainly to 15-HETE (30). Unfortunately, except for mouse and human ALOX15B the reaction specificities of other mammalian ALOX15B orthologs have not been studied. Thus, it remains unclear whether the different reaction specificities of mouse and human ALOX15B orthologs might also be the results of a targeted enzyme evolution as it has previously been suggested for mammalian ALOX15 orthologs (23, 24).

To address this question, we extracted the cDNA sequences of different mammalian ALOX15B orthologs from online sequence databases and found that the vast majority (>90%) of mammalian ALOX15B orthologs carry an Asp+Val, Asp+Ile, Asp+Met, and His-Ala (HA) motif at the Jisaka positions. These sequence motifs were highly suggestive for AA 15-lipoxygenating enzymes. However, the ALOX15B orthologs of several of old-world mouse species including *Mus musculus*, *Mus pahari*, *Mus caroli*, *Mastomys coucha,* and *Arvicanthis niloticus* carry a Tyr+His motif at the Jisaka positions and these amino acids suggested AA 8-lipoxygenating ALOX15B orthologs. Recombinant expression of 20 mammalian ALOX15B orthologs representing different clades of *Prototheria* and *Eutheria* confirmed these functional predictions. In *Metatheria,* functional *ALOX15B* genes rarely occur. In summary, it can be concluded that the reaction specificity of mammalian ALOX15B isoforms has not been systematically modified during mammalian evolution and that the AA 8S-lipoxygenating activity of mouse Alox15b represents an evolutionary exception.

## Materials and Methods

### Chemicals

The chemicals used for this study were obtained from the following sources: AA, EPA, DHA, and authentic HPLC standards of HETE isomers (5*S/R*-HETE, 5*S*-HETE, 8*S/R*-HETE, 8*S*-HETE, 12*S/R*-HETE, 12*S*-HETE, 15*S/R*-HETE, and 15*S*-HETE) from Cayman Chem. Acetic acid from Carl Roth GmbH (Karlsruhe, Germany); sodium borohydride from Life Technologies, Inc. (Eggenstein, Germany); IPTG from Carl Roth GmbH (Karlsruhe, Germany); restriction enzymes from Thermo Fisher Scientific (Schwerte, Germany); the *Escherichia coli* strain Rosetta2 DE3 pLysS from Novagen (Merck Millipore, Darmstadt, Germany); HEK293 cells from the German Collection of Microorganisms and Cell Culture GmbH (DSMZ, Braunschweig, Germany). The Bac-to-Bac® baculovirus expression system and Sf9 insect cells were purchased from Invitrogen Life Technologies (Thermo Fisher Scientific, Schwerte, Germany). Oligonucleotides were synthesized by BioTeZ Berlin Buch GmbH (Berlin, Germany). Nucleic acid sequencing was performed at Eurofins MWG Operon (Ebersberg, Germany). HPLC grade solvents and water were purchased from Thermo Fisher Scientific (New Hampshire, USA).

### Database searches

The amino acid sequences of different mammalian ALOX15B orthologs were extracted from the NCBI database (https://www.ncbi.nlm.nih.gov/). For those mammals, for which no complete ALOX15B cDNA sequences were found, we searched the genomic databases with the genomic sequences of human ALOX15. The cDNA sequences were extracted and were aligned with human ALOX15B to identify the Jisaka determinants.

### Cloning of mammalian ALOX15B orthologs

To characterize the functional properties of selected ALOX15B orthologs, their cDNA sequences were chemically synthesized. Before synthesis a *Sal*I recognition site was introduced immediately upstream of the start codon and the starting ATG was kept unaltered. Next, a *Hind*III recognition site was designed immediately downstream the stop codon and internal *Hind*III and *Sal*I restriction sites were eliminated by silent nucleotide exchanges. Then, the cDNA sequences were optimized for bacterial expression and the constructs were chemically synthesized into the multicloning region of the pUC57 cloning vector (BioCat GmbH, Heidelberg, Germany). For prokaryotic expression, the modified coding region of the ALOX15B cDNAs were excised from the synthesizing vector and cloned into the expression plasmid pET28b (Novagen/Merck, Darmstadt, Germany). Recombinant expression plasmids were tested for the presence of the ALOX15B inserts by *Sal*I + *Hind*III digestion and the final expression constructs were sequenced (Eurofins Genomics Germany GmbH, Ebersberg, Germany).

### Bacterial expression of ALOX15B orthologs

Bacterial expression of recombinant ALOX15B orthologs was performed as described in (24) for mammalian ALOX15 orthologs. In brief, competent *E. coli* cells (strain Rosetta 2 DE3 pLysS) were transformed with 50–100 ng of the recombinant expression plasmid and the cells were grown overnight on kanamycin/chloramphenicol-containing agar plates. An isolated bacterial clone was picked and two 1 ml bacterial liquid cultures (LB medium with 50 μg/ml kanamycin and 35 μg/ml chloramphenicol) were grown for 6–8 h at 37°C under gentle agitation at 180 rpm. An appropriate volume of the precultures was then added to 50 ml of sterile culture medium (ENPRESSO B kit, Enpresso GmbH, Berlin, Germany) in ultrayield culture flasks (Thomson Instrument Company, Oceanside) containing kanamycin (50 μg/ml) and chloramphenicol (35 μg/ml) as antibiotics. The cells were grown overnight at 30°C and 250 rpm until the A_600_ had reached values above 5. Expression of the recombinant proteins was induced by the addition of IPTG to reach a final concentration of 1 mM. Then, the cultures were maintained at 22°C for 18 h at 230–250 rpm agitation. Bacteria were harvested, the resulting pellet was reconstituted in a total volume of 5 ml PBS, and bacteria were lysed by sonication (digital sonifier, W-250D Microtip Model 102, 50% maximal sonication amplitude; Branson Ultraschall, Fürth, Germany). Cell debris was spun down (15 min, 15,000 *g*, 4°C) and the bacterial lysate supernatants were used for activity assays, protein quantification, SDS-PAGE, and Western blot analyses.

### Eukaryotic expression of ALOX15 orthologs in HEK293 cells

Mammalian ALOX15B orthologs, for which the expression levels in *E. coli* were not high enough, were expressed in HEK293 cells (24). For this purpose, the coding sequence including the starting methionine and the his-tag epitope of the prokaryotic expression plasmid was excised from the recombinant expression plasmid employing *XbaI* and *HindIII* restriction endonucleases. After linearization of the eukaryotic expression vector pcDNA 3.1 (−) (Thermo Fisher Scientific, Schwerte, Germany) with the same enzyme combination, the 2 kbp restriction fragment (ALOX insert) was ligated into the linearized vector using the Rapid DNA Ligation Kit (Thermo Fisher Scientific). The recombinant plasmid was amplified in *E. coli XL-1 Blue* competent cells (ThermoFisher Scientific, Schwerte, Germany). HEK239 cells were seeded at a density of about 4 × 10^5^ cells per 2 ml in DMEM (4.5 g/l glucose, L-glutamine, 1mM sodium pyruvate, 3.7 g/l NaHCO3 containing 10% FCS) into each well of a 6-well plate (Sarstedt, Nümbrecht, Germany). Cells were allowed to attach to the plastic dishes for 24 h prior to transfection. After attachment the cells were transfected using the TransIT-LT1 transfection kit (Mirus Bio, Madison). For this purpose, 2 μg of plasmid DNA were mixed with 6 μl TransIT-LT1 in 194 μl Opti-MEM (Life Technologies, Inc., Eggenstein, Germany) and transfection complexes were allowed to form according to the manufacturer's protocol. Finally, the transfection mixture was added to each well and the cells were incubated with the transfection mixture at 37°C. After 48 h incubation, the cells were washed with PBS, spun down (1,000 *g* for 5 min), and the cell pellet was reconstituted in 0.5 ml of PBS. Cells were disrupted by sonication using an UP50H tip sonifier (Hielscher Ultrasound Technology, Teltow, Germany). Cell debris was spun down (15,000 *g*, 20 min, 4°C) and the lysis supernatant was used for activity assays, protein quantification, SDS-PAGE and Western blot analyses.

### Expression of ALOX15 orthologs in Sf9 insect cells

ALOX15B orthologs, which could neither be effectively expressed in *E. coli* nor in HEK293 cells, were expressed as N-terminal recombinant hexa-his-tag fusion proteins in Sf9 insect cells (24). For this purpose, the coding regions of the ALOX15B cDNA including the starting Met of the N-terminal his-tag fusion proteins were excised from the recombinant bacterial expression plasmids and this construct was ligated into the pFastBac HT vector. The bacmids and the recombinant baculoviruses were generated according to the manufacturer's instructions (Bac-to-Bac® Baculovirus Expression System, Invitrogen Life Technologies/Thermo Fisher Scientific, Schwerte, Germany). Protein expression was initiated in *Sf9* cells (ATCC® CRL-1711) cultures using the Insect XPRESS Medium (Biozym Scientific GmbH, Hessisch Oldendorf, Germany) supplemented with 4 mM glutamine and 0.5% FCS. The cells were infected with the amplified baculovirus and subsequently incubated at 27°C and 120–130 rpm on an agitation platform. After 72 h of incubation (30% dead cells), the cells were harvested by centrifugation, lysed by sonication and the lysate supernatant were used for activity assays, protein quantification, SDS-PAGE, and Western blot analyses.

### SDS-PAGE and quantitative Western blotting

To quantify ALOX15B expression in *E. coli* aliquots (0.1–20 μl) of the lysis supernatants were analyzed by SDS-PAGE on a 7.5% polyacrylamide gel. Separated proteins were transferred to a nitrocellulose membrane (Thermo Fisher Scientific GmbH, Schwerte, Germany) by a Western blotting method (ProSieve Ex Western Blot Transfer Buffer 10x, Biozym Scientific GmbH, Hessisch-Oldendorf, Germany). The membranes were blocked with blocking solution (10-fold BlueBlock PF for Blotting, SERVA Electrophoresis GmbH, Heidelberg, Germany), washed three times with PBS containing 0.3% Tween 20, and were finally incubated with an anti-His-HRP antibody (Miltenyi Biotec GmbH, Bergisch Gladbach, Germany) for 1–2 h at room temperature. After several steps of washing, the membranes were stained using the SERVALight Polaris CL HRP WB Substrate Kit (SERVA Electrophoresis GmbH, Heidelberg, Germany) for 5 min at room temperature. Chemiluminescence was quantified using the FUJIFILM Luminescent Image Analyzer LAS-1000plus (Fujifilm Europe GmbH, Düsseldorf, Germany). For quantification of the intensity scale of the immunoblots known amounts of pure recombinant ALOX of *Myxococcus fulvus*, which was also expressed as N-terminal his-tag fusion protein in *E. coli* (31), were applied to Western blot analysis. The intensities of the immunoreactive bands were quantified using the ImageJ software package and from the band intensities we calculated the amounts ALOX proteins in the different enzyme preparations.

### Site-directed mutagenesis

Site-directed mutagenesis was carried out using the PfuUltra II Hotstart PCR kit (Agilent Technologies Germany GmbH & Co. KG, Waldbronn, Germany). For PCR 10–50 ng recombinant plasmid DNA were incubated with the specific primer pair (1 μl of 5 μM solution each) and 12.5 μl Pfu UltraI II Hot Start PCR Master Mix in a total volume of 25 μl adjusted with sterile water. The following PCR protocol was used: 95°C for 1 min initial denaturation, cycle: 30 s at 95°C (denaturation phase), and then 60 s at 55°C (annealing phase) followed by the synthesis phase (10 min at 68°C). This cycle was repeated 18 times. Subsequently the parent cDNA was digested with 1 μl DpnI (Thermo Fisher Scientific, Schwerte, Germany) for 30 min and the digestion was stopped by incubating the samples at 80°C for 10 min. Eight microliters of the PCR sample were used for transformation of competent *E. coli* XL-1 Blue cells (Agilent Technologies Inc., Santa Clara). After incubation for 30 min on ice, the cells were heat-shocked for 45 s at 42°C, kept on ice for two additional min, and then 400 μl super optimal broth with catabolite repression medium was added. After 1 h incubation at 37°C, the cells were plated on an LB-agar plate supplemented with 50 μg/ml kanamycin and incubated overnight at 37°C. Four isolated growing bacterial clones were selected and 1 ml bacterial liquid cultures were grown overnight in LB medium. Finally, plasmid DNA was prepared using the NucleoSpin Plasmid kit (Macherey & Nagel, Düren, Germany), and DNA sequencing of the mutated plasmid region was carried out by the Eurofins Genomics Germany GmbH (Ebersberg, Germany) to confirm that the nucleotide exchanges that were introduced with our in vitro mutagenesis strategy were actually present in the plasmid DNA.

### In vitro ALOX activity assays

To assay the catalytic activity of the recombinant enzymes, variable amounts of the enzyme preparations (bacterial lysate supernatants, HEK293 lysate supernatants, Sf9 insect cell supernatants) were added to 0.5 ml PBS containing 100 μM of AA as ALOX substrate. After a 5 min of incubation period, the hydroperoxy AA derivatives formed were reduced to the corresponding alcohols (addition 1 mg of solid sodium borohydride), the samples were acidified (35 μl of acetic acid), proteins were precipitated (addition of 0.5 ml of ice-cold acetonitrile), and the precipitate was spun down. Aliquots of the protein-free supernatants (50–300 μl) were injected to reverse-phase high performance liquid chromatography (RP-HPLC) of the ALOX products. For this purpose, a Shimadzu instrument equipped with a diode array detector (SPD-M20A) and an autoinjector (SIL-20AC) was used and metabolites were separated on a Nucleodur C18 Gravity column (Macherey-Nagel, Düren, Germany; 250 × 4 mm, 5 μm particle size) coupled with a guard column (8 × 4 mm, 5 μm particle size). A solvent system consisting of acetonitrile:water:acetic acid (70:30:0.1, by vol) was employed at a flow rate of 1 ml/min and analytes were eluted isocratically at 25°C. For quantification, the chromatographic scale was calibrated by injecting known amounts of 15-HETE (six-point calibration curve). Similar activity assays were carried out with EPA and DHA and the oxygenation products were analyzed by RP-HPLC as described for the AA oxygenation products.

### Data evaluation and statistics

Statistic evaluation of the activity data and quantification of the patterns of AA oxygenation products was carried out with the two-sided Student's *t* test using the Microsoft Excel software package (Excel 2016). Numeric *P* values <0.05 were considered statistically significant.

## Results

### Functional ALOX15B genes frequently occur in the genomes of *Prototheria* and *Eutheria* but they are absent in *Metatheria*

**Fig. 1:**
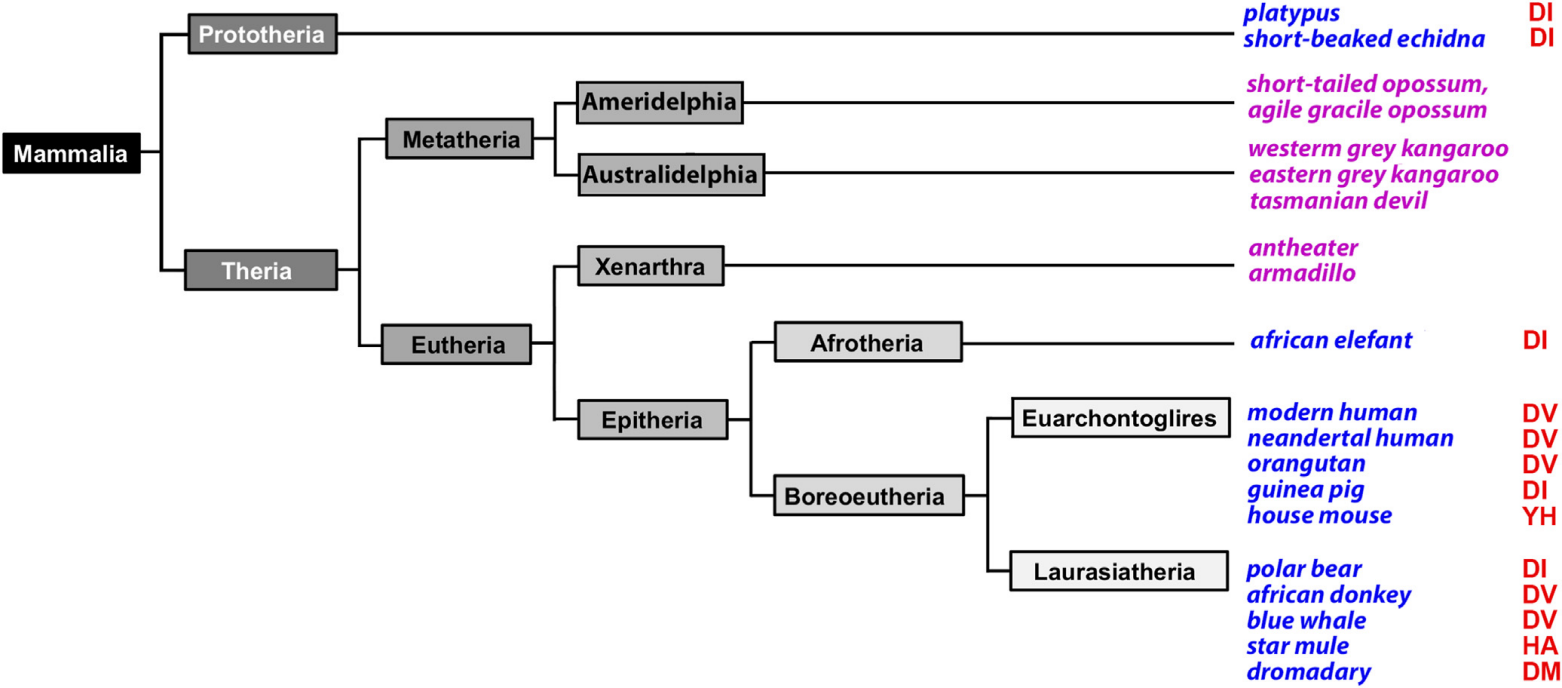
Simplified classification of mammals and Jisaka determinants of ALOX15B orthologs of selected mammalian species. The evolutionary class of mammals is divided in two subclasses (*Prototheria* and *Theria*). Platypus and four different Echidna species represent the currently living *Prototheria*. The genomes of platypus and of the short-beaked echidna involve functional ALOX15B genes and the Jisaka determinants are occupied Asp-Ile (DI) motif (red letters on the right). *Theria* can further be subclassified into *Metatheria* and *Eutheria* and the two clades can further be subdivided. According to their geographic origin, Metatheria can be subclassified into *Ameridelphia* and *Australidelphia,* but in the genomes of indicated species, we could not find functional ALOX15B genes. Similarly, the genomes of armadillo and of different anteater species, which represent the eutherian superorder *Xenarthra*, do neither involve functional *ALOX15B* genes. In contrast, functional ALOX15B genes were detected in the genomes of many representatives of the eutherian superorders *Afrotheria*, *Euarchontoglires,* and *Laurasiatheria* and the Jisaka determinants are occupied by four different amino acid motifs: Asp-Ile (DI), Asp-Val (DV), Asp-Met (DM), and His-Ala (HA). Species, in the genomes of which functional *ALOX15B* genes were identified, are labeled in blue. Mammalian species lacking functional *ALOX15B* genes are labeled in magenta. The one-letter code for the amino acids is used in the image. The time scale of the tree was not normalized. AA, arachidonic acid; ALOX, arachidonic acid lipoxygenase.

According to their evolutionary ranking, *Mammalia* can be classified in *Prototheria*, *Metatheria,* and *Eutheria* (Fig. 1) and today each of these three evolutionary clades are represented by extant crown groups. *Monotremata* represent *Prothotheria*, *Marsupialia* represent *Metatheria,* and *Placentalia* represent *Eutheria*. In addition to these crown groups, extinct species are classified into the three clades, which are more closely related to the corresponding crown-group than to any other living animals. To explore whether the reaction specificity of mammalian ALOX15B orthologs depends on the evolutionary ranking of the species, we first extracted *ALOX15B* genes from public genomic databases, retrieved the cDNA sequences, translated them into the amino acid sequences of the corresponding proteins, and carried out amino acid sequence alignment with human and mouse AOLX15B orthologs to identify the Jisaka determinants (amino acids aligning with Asp602and Val603 of human ALOX15B). These residues are of major importance for the reaction specificity of mouse and human ALOX15B orthologs (29).

As indicated in Table 1, we identified close to complete *ALOX15B* gene sequences in the two tested prototherian mammals (*Tachyglossus aculeatus and Ornithorhynchus anatinus*), in two of the eight tested *Metatheria* (*Aspergillus flavipes, Sarcophilus harisii*) and in a large number of eutherian species. Dual amino acid sequence alignments (supplemental Figs. S1–S92) with human ALOX15B indicated that the sizes of the encoded proteins (676–678) were similar and that the proteinogenic iron ligand were highly conserved in most cases. These data suggest the functionality of the corresponding enzymes. However, the extracted cDNA sequences also indicated that some ALOX15B orthologs (e.g. *S. harisii*, *Galeopterus variegatus, Celosia cristata, Lipotes vexillifer)* were smaller in size, whereas others involved additional amino acids (*Jaculus jaculus, Chlorocebus sabaeus, Orcinus orca, Suricata suricatta, Equus przewalskii*). In the absence of direct functional data, it is difficult to predict whether these genes encode for functional enzymes.

**Table 1:** Sequence characteristics of mammalian ALOX15B orthologs

No. (Number)	Species	Systematic Name	Jisaka Positions	Amino Acids	Expressions Systems			Major Product
*Prototheria*								
1	Short-beaked echidna	*T. aculeatus*	DI	676	*E. coli*, Sf9			15-HETE
2	Platypus	*O. anatinus*	DI	677	Sf9			15-HETE
*Metatheria*								
*Australidelphia*								
3	Yellow-footed pouch mouse	*A. flavipes*	HA	676	*E. coli*			15-HETE
4	Tasmanian devil	*S. harrisii*	HA	645	Not expressed			
5	Western gray kangaroo	*M. fuliginosus*			No intact ALOX15B gene			
6	Eastern gray kangaroo	*M. gigantis*			No intact ALOX15B gene			
*Ameridelphia*								
7	Short-tailed opossum	*M. domestica*			No intact ALOX15B gene			
8	Agile gracile opossum	*G. agilis*			No intact ALOX15B gene			
*Eutheria*								
*Xenarthra*								
9	Armadillo	*D. novemcinctus*			No intact ALOX15B gene			
10	Giant anteater	*M. tridactyla*			No intact ALOX15B gene			
*Afrotheria*								
11	African elephant	*L. africana*	DI	676	Not expressed			
*Euarchontoglires*								
12	Neanderthal (extinct human)	*H. neanderthalensis*	DV	676	*E. coli*			15-HETE
13	Modern human	*H. sapiens*	DV	676	*E. coli*			15-HETE
14	Denisovan (extinct human)	*H. denisovan*	DV	676	*E. coli*			15-HETE
15	Gorilla	*G. gorilla*	DI	676	*E. coli*			15-HETE
16	Orangutan	*P. pygmaeus*	DV	676	*E. coli*			15-HETE
17	Chimpanzee	*P. troglodytes*	DI	676	*E. coli*			15-HETE
18	Anubis baboon	*P. anubis*	DV	676	Not expressed			
19	Rhesus macaque	*M. mulatta*	DV	676	Not expressed			
20	Sooty mangabey	*C. atys*	DV	676	Not expressed			
21	Southern pig-tailed macaque	*M. nemestrina*	DV	677	Not expressed			
22	Crab-eating macaque	*M. fascicularis*	DV	676	Not expressed			
23	Gelada	*T. gelada*	DV	676	Not expressed			
24	Drill	*M. leucophaeus*	DV	676	Not expressed			
25	Philippine tarsier	*C. syrichta*	DV	677	Not expressed			
26	Nancy Ma’s night monkey	*A. nancymaae*	DV	677	Not expressed			
27	Angola colobus	*C. angolensis pall.*	DV	676	Not expressed			
28	Ugandan red colobus	*P. tephrosceles*	DV	676	Not expressed			
29	Golden snub-nosed monkey	*R. roxellana*	DV	676	Not expressed			
30	Black and white snub-nosed monkey	*R. bieti*	DV	676	Not expressed			
31	Malayan flying lemur	*G. variegatus*	DV	662	Not expressed			
32	Gray mouse lemur	*M. murinus*	DI	677	Not expressed			
33	Ring-tailed lemur	*L. catta*	DV	677	Not expressed			
34	Green monkey	*C. sabaeus*	DV	727	Not expressed			
35	Coquerel’s sifaka	*P. coquereli*	DV	677	Not expressed			
36	Northern greater galago	*O. garnettii*	DV	678	Not expressed			
37	Arctic ground squirrel	*U. parryii*	DI	678	Not expressed			
38	Yellow-bellied marmot	*M. flaviventris*	DI	678	Not expressed			
39	Banner-tailed kangaroo rat	*D. spectabilis*	DV	676	Not expressed			
40	North American beaver	*C. canadensis*	DV	677	Not expressed			
41	Golden hamster	*M. auratus*	DV	677	Not expressed			
42	Chinese hamster	*C. griseus*	DV	677	Not expressed			
43	Lesser Egyptian jerboa	*J. jaculus*	DV	713	Not expressed			
44	Deer mouse	*P. maniculatus ba.*	DV	677	Not expressed			
45	Prairie vole	*M. ochrogaster*	DV	677	Not expressed			
46	Creeping vole	*M. oregoni*	DV	677	Not expressed			
47	European water vole	*A. amphibius*	DV	677	Not expressed			
48	Common degu	*O. degus*	DI	677	Not expressed			
49	European Rabbit	*O. cuniculus*	DV	678	Not expressed			
50	Grammomys	*G. surdaster*	DV	678	Not expressed			
51	House mouse	*M. musculus*	YH	677	*E. coli*	8-HETE		
52	Sikkim mouse	*M. pahari*	YH	677	*E. coli*	8-HETE		
53	Ryukyu mouse	*M. caroli*	YH	677	*E. coli*	8-HETE		
54	Southern multi-mammate mouse	*M. coucha*	YH	677	*E. coli*	8-HETE		
55	Long-tailed chinchilla	*C. lanigera*	DI	677	*E. coli*	15-HETE		
56	Guinea pig	*C. porcellus*	DI	677	*E. coli*	15-HETE		
57	American pika	*O. princeps*	DI	677	*E. coli*	15-HETE		
58	Plateau pika	*O. curzoniae*	DI	677	Not expressed			
59	Brown rat	*R. norvegicus*	DV	677	Not expressed			
60	Black rat	*R. rattus*	DV	677	Not expressed			
61	African grass rat	*A. niloticus*	YH	677	Not expressed			
*Laurasiatheria*								
62	Star-nosed mole	*C. cristata*	HA	673	Sf9	Inactive		
63	Domestic cat	*F. catus*	DI	677	Not expressed			
64	Striped hyena	*H. hyaena*	DI	676	Not expressed			
65	Leopard	*P. pardus*	DI	677	Not expressed			
66	Cheetah	*A. jubatus*	DI	677	Not expressed			
67	Giant panda	*A. melanoleuca*	DI	677	Not expressed			
68	Domestic dog	*C. lupus familiaris*	DI	677	Not expressed			
69	Red fox	*V. vulpes*	DI	677	Not expressed			
70	Northern fur seal	*C. ursinus*	DI	677	Not expressed			
71	California sea lion	*Z. californianus*	DI	677	Not expressed			
72	Steller sea lion	*E. jubatus*	DI	678	Not expressed			
73	Pacific walrus	*O. rosmarus div.*	DI	677	Not expressed			
74	Gray seal	*H. grypus*	DI	677	Not expressed			
75	Harbor seal	*P. vitulina*	DI	677	Not expressed			
76	Blue whale	*B. musculus*	DV	677	HEK			15-HETE
77	Chinese river dolphin	*L. vexillifer*	DV	659	Not expressed			
78	Vaquita	*P. sinus*	DV	677	Not expressed			
79	Orca	*O. orca*	DV	684	Not expressed			
80	Narwhale	*M. monoceros*	DV	677	Not expressed			
81	Common bottlenose dolphin	*T. truncatus*	DV	684	*E. coli*		15-HETE	
82	Polar bear	*U. maritimus*	DI	677	*E. coli*		15-HETE	
83	African wild donkey	*E. asinus*	DV	677	*E. coli*		15-HETE	
84	Przewalski's horse	*E. przewalskii*	DV	772	Not expressed			
85	Cattle	*B. taurus*	DV	677	Not expressed			
86	Bactrian camel	*C. ferus*	DM	668	Sf9		Inactive	
87	Dromedary	*C. dromedarius*	DM	678	HEK		15-HETE	
88	Meerkat	*S. suricatta*	DI	685	Not expressed			
89	Ferret	*M. puterius furo*	DI	677	Not expressed			
90	Stoat	*M. erminea*	DI	677	Not expressed			

The cDNA sequences of mammalian ALOX15B orthologs were extracted from the genomic databases. The nucleotide sequence was translated and dual amino acid alignments with human and mouse ALOX15B orthologs identified the Jisaka determinants, which are predictive for the reaction specificity of mammalian ALOX15B orthologs. Asp+Val (DV), Asp+Ile (DI), and Asp+Met (DM) motifs at the Jisaka positions suggest AA 15-lipoxygenating ALOX15B orthologs but the Tyr+His (YH) motif suggests AA 8-lipoxygenating enzymes. Not expressed, we did not express these enzymes in any of the recombinant expression systems.

**Fig. 2:**
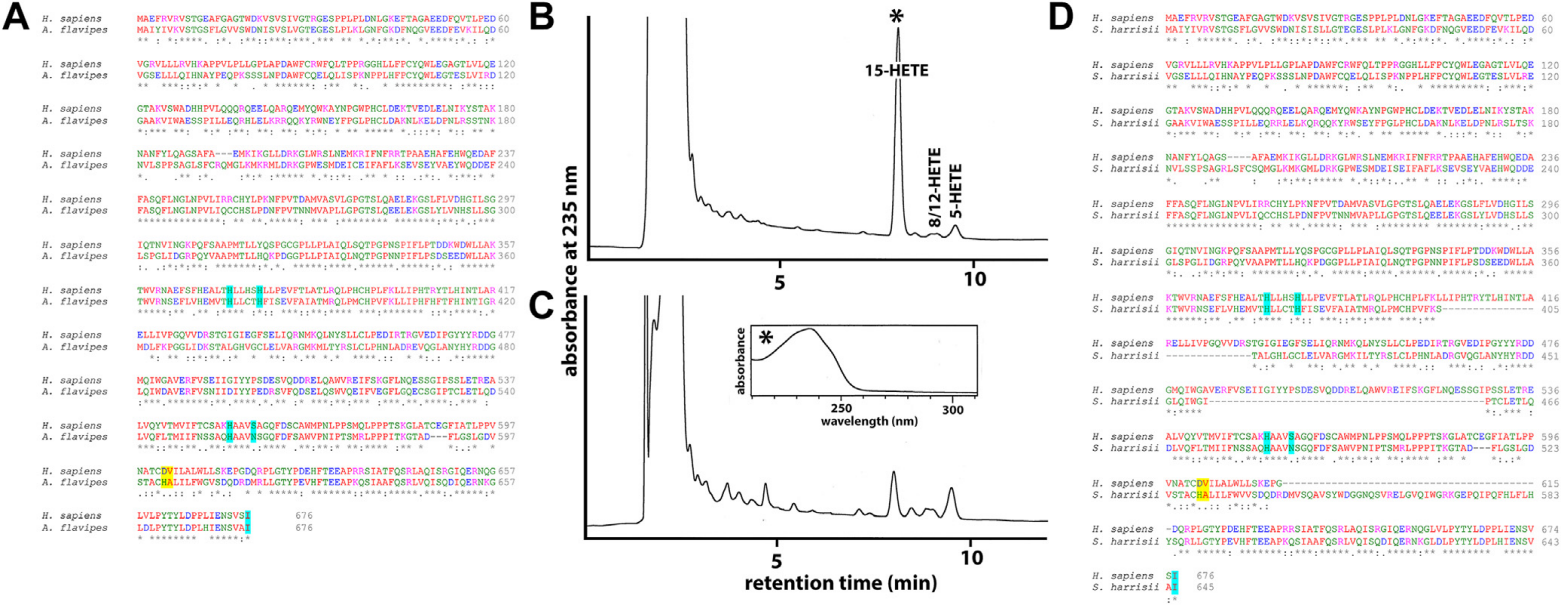
Dual sequence alignments of selected metatherian ALOX15 orthologs with human ALOX15B and catalytic activity of recombinant *A. flavipes* ALOX15B. A: Dual amino acid alignment *A. flavipes* ALOX15B with human ALOX15B. B: RP-HPLC analysis of the AA oxygenation products formed by recombinant *A. flavipes* ALOX15B during in vitro activity assays. C: AA oxygenation products formed during no enzyme control incubations. Inset: UV-spectrum of the major AA oxygenation product formed by *A. flavipes* ALOX15B D: Dual amino acid alignment of *S. harrisii* ALOX15B with human ALOX15B. AA, arachidonic acid; ALOX, arachidonic acid lipoxygenase.

To identify *ALOX15B* genes in *Metatheria,* we searched the genomes six *Australidelphia* and two *Ameridelphia* (Table 1). In the genome of *A. flavipes* representing *Australidelphia,* we detected a functional *ALOX15B* gene. The Jisaka positions were occupied by an HA motif, the iron ligands were conserved (Fig. 2A), and the recombinant enzyme (expressed in *E. coli*) oxygenated AA almost exclusively to 15-HETE (Fig. 2B). When no enzyme control incubations (transformation of *E. coli* with a nonrecombinant expression plasmid) were carried out only small amounts of 15-HETE were detected by HPLC in our in vitro activity assays (Fig. 2C). These data suggested the functionality of the *A. flavipes ALOX15B* gene. The genome of the Tasmanian devil (*S. harrisii)*, which is also classified as *Australidelphia*, did also involve an *ALOX15B* gene. However, when we extracted the corresponding cDNA and carried out a dual amino acid alignment with human ALOX15B, we found that the encoded protein did only comprise 645 amino acids (Fig. 2D). The Jisaka positions are occupied by the HA motif (Fig. 2D) but between the iron cluster 1 (H-L-L-C-T-H) and iron cluster 2 (H-A-A-V-N) a large number of amino acids are lacking. In addition, extra amino acids are present in the C-terminal part of the *S. harrisii ALOX15B*, which are absent in human ALOX15B (Fig. 2C). These structural data suggest that the *S. harrisii* ALOX15B gene may not encode for a functional enzyme.

When we search the genomes of *Macropus fuliginosus* (64-fold genome coverage), *Machairodus gigantis* (70-fold genome coverage), *Plethodon cinereuus* (57-fold genome coverage), and *Vombatus ursinus* (87-fold genome coverage), which also represent *Australidelphia*, we did not find complete ALOX15B genes. However, we detected a number of partial ALOX15B sequences in all of these genomes. A similar situation was observed when we searched the genomes of *Mus domesticus* (33-fold genome coverage) and *Gracilinanus agiles* (100-fold genome coverage) representing *Ameridelphia* (Table 1). Here again, we only detected partial ALOX15B sequences, which did not encode for functional enzymes. In *Eutheria,* functional ALOX15B genes do frequently occur (Table 1). Although we did not find such genes in the genomes of *Dasypus novemcinctus and Myrmecophaga tridactyla* representing *Xenarthra,* we identified such genes in *Afrotheria (Loxodonta africana)* and in a large number of *Euarchontoglires* and *Laurasiatheria* (Table 1).

In summary, our database searches indicated that functional ALOX15B genes frequently occur in *Prototheria* and *Eutheria* but that they are rare in *Metatheria*. However, the presence of incomplete ALOX15B sequences in the genomes of several *Metatheria* suggested that ALOX15B genes might have been present in ancient *Metatheria* but were heavily mutated during later evolution. Thus, functional ALOX15B orthologs are obviously not essential for most *Metatheria*.

### Mammalian ALOX15B orthologs carry Asp+Val, Asp+Ile, Asp-Met, and His+Ala motifs at the Jisaka positions

**Fig. 3:**
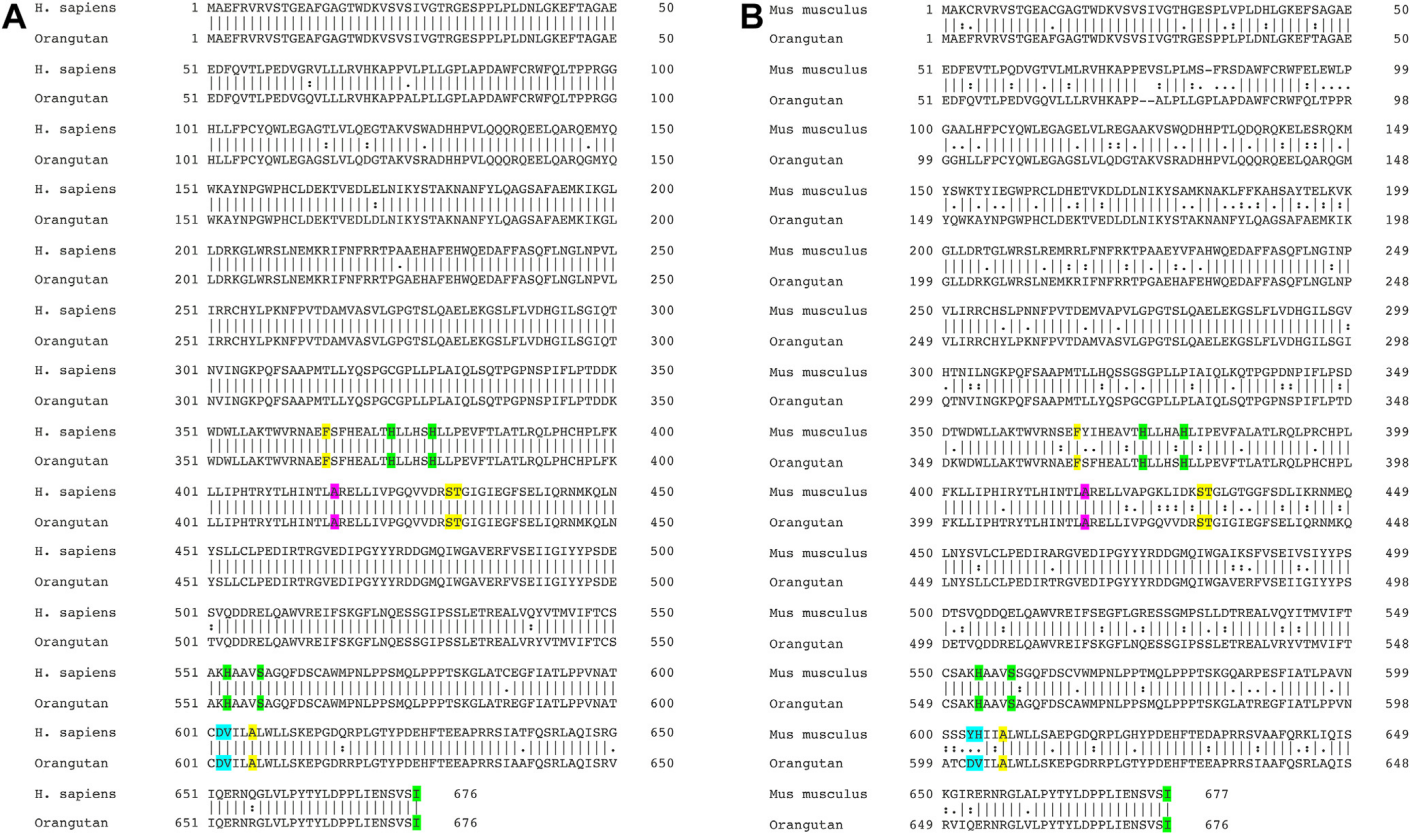
Dual amino acid sequence alignments of orangutan ALOX15B with the corresponding sequences of human and mouse ALOX15B orthologs. A: Dual amino acid alignment of human and orangutan ALOX15B. B: Dual amino acid alignment of mouse and orangutan ALOX15B. The following color code was used for specification of functionally relevant amino acids: green: proteinogenic iron ligand; yellow: triad determinants; red: Coffa/Brash determinants; and blue: Jisaka determinants. AA, arachidonic acid; ALOX, arachidonic acid lipoxygenase.

In the genomes of *Euarchontoglires* and *Laurasiatheria,* functional ALOX15B genes frequently occur (Table 1). To identify the Jisaka determinants (29), we next performed dual amino acid sequence alignments of the novel mammalian ALOX15B orthologs with human and mouse ALOX15B. In Figure 3A, an example for the dual amino acid alignments of the orangutan ALOX15B with its human ortholog is shown. The three enzymes (human ALOX15B, mouse Alox15b, orangutan ALOX15B) share a high degree of amino acid identity and the iron ligands are highly conserved. As in human ALOX15B, the Jisaka positions are occupied by the Asp+Val (DV) motif and this data suggests that the orangutan enzyme constitutes an AA 15-lipoxygenating protein. Although the triad determinants are not of major functional relevance for mammalian ALOX15B orthologs (32), they are also strictly conserved between the three ALOX15B orthologs. We also observed conservation of the Ala417 (Coffa-Brash determinant), which classifies orangutan ALOX15B as S-lipoxygenating enzyme. Taken together, these sequence data prompted the prediction that orangutan ALOX15B is a functional enzyme, which oxygenates free AA almost exclusively to 15S-HETE.

A similar amino acid sequence alignment with mouse Alox15b (Fig. 3B) confirmed conservation of the iron ligands, the triad determinants and the Coffa-Brash determinant. However, at the Jisaka positions (29), mouse Alox15b carries a Tyr+His (YH) motif instead of the Asp+Val sequence. This difference predicts that the reaction specificity of the two enzymes should be different. In fact, mouse Alox15b is an AA 8S-lipoxygenating enzyme (16, 28) but for orangutan ALOX15B AA 15S-lipoxygenation has been predicted on the basis of its structural properties.

When similar amino acid alignments (supplemental Figs. S1–S92) were carried out for the other novel mammalian ALOX15B orthologs, we found that the vast majority of these enzymes (Table 1) involve either of the following four sequence motifs at the Jisaka positions: Asp+Val (DV), Asp+Ile (DI), Asp+Met (DM), and His+Ala (HA). Only the ALOX15B orthologs of several old-world mouse species including *M. musculus*, *M. pahari*, *M. caroli*, *M. coucha* and *A. niloticus* (blue in Table 1) carry the Tyr+His (YH) sequence motif at this position. Since the *M. musculus* Alox15b catalyzes specific AA 8S-lipoxygenation it was predicted that the enzyme orthologs of *M. pahari, M. caroli*, *M. coucha,* and *A. niloticus* may also oxygenate AA predominantly to 8S-HETE. However, in the absence of direct functional data this structure-based conclusion might not be correct and thus, direct functional investigations are required to test this working hypothesis.

### Functional consequences of amino acid exchanges of the Jisaka determinants in human ALOX15B

**Fig. 4:**
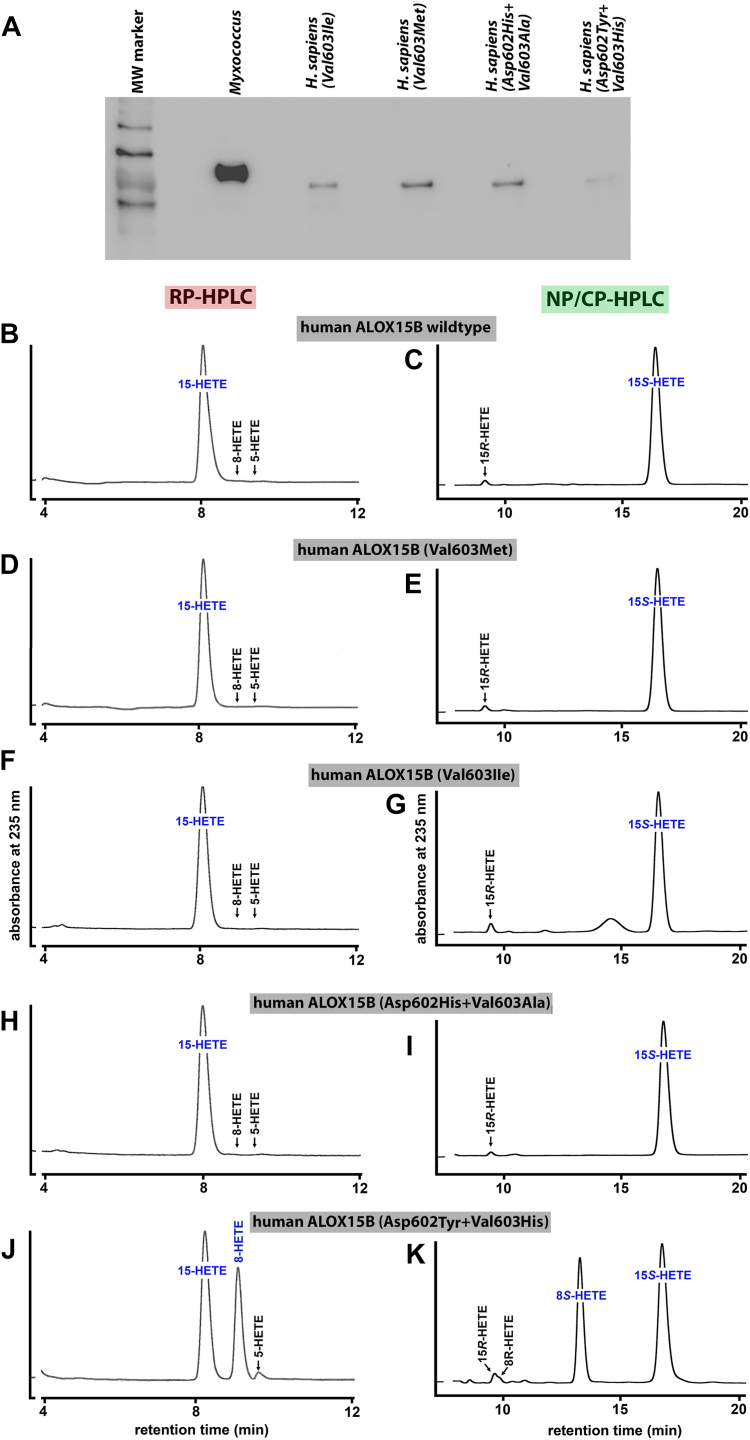
HPLC analysis of the AA oxygenation products formed during in vitro activity assays by human ALOX15B mutants. Wild-type human ALOX15B and different Jisaka mutants of the enzyme were expressed in *E. coli*. In vitro activity assays and HPLC analysis (consecutive RP-HPLC and combined NP/CP-HPLC) of the reaction products were carried out as described in Materials and methods. The chemical identity of the oxygenation products was concluded from the retention times and from the UV-spectral properties of the conjugated dienes. A: Quantitative Western blot analysis of recombinant expression of human ALOX15B variants. B, D, F, H, J: RP-HPLC analysis separating the HETE positional isomers. C, E, G, I, K: NP/CP-HPLC analysis separating the HETE enantiomers. AA, arachidonic acid; ALOX, arachidonic acid lipoxygenase.

To explore the functional consequences of side-directed mutagenesis at the Jisaka determinants for the reaction specificity of human ALOX15B, we first expressed wildtype and mutant human ALOX15B as catalytically active recombinant N-terminal his-tag fusion protein and compared the relative expression levels of the his-tag fusion proteins by quantitative Western blotting (Fig. 4A). As reference protein, we applied defined amounts (1 μg) of the *M. fulvus* ALOX, which was also expressed as recombinant N-terminal his-tag fusion protein and was purified to electrophoretic homogeneity by affinity chromatography on Ni-agarose. As indicated in Figure 4A, all enzyme variants are expressed in *E. coli* although the expression levels were somewhat different.

Next, we performed in vitro activity assays using recombinant wild-type human ALOX15B and the expressed Jisaka mutants as catalyst and analyzed the product patterns. From Figure 4B, C, it can be seen that wild-type recombinant human ALOX15B was catalytically active and oxygenated AA almost exclusively to 15S-HETE. Other HETE isomers, such as 8-HETE, 5-HETE, as well as 15R-HETE were absent. Val603Met exchange (DM motif) did not alter the reaction specificity of human ALOX15B (Fig. 4D+E) and similar results were obtained for the Val60Ile mutant (Fig. 4F, G) and the Asp602His+Val603Al double mutant (Fig. 4H+I). Thus, as wild-type human ALOX15B the mutant enzymes carrying the DM, the DI, and the DV motifs remain AA 15-lipoxygenating ALOX15B variants. In contrast, when the Jisaka determinants were mutated to the corresponding residues present at these positions in mouse Alox15b (Asp602Tyr+Val603His, YH motif), we observed a dramatic change in the reaction specificity (Fig. 4J, K). This double mutant oxygenated AA to similar amounts of 15S-HETE and 8S-HETE and these data are consistent with previous results (29). In other words, this mutagenesis strategy partly murinized the reaction specificity of human ALOX15B.

On the basis of these mutagenesis results (Fig. 4) and on the sequence data reported in Table 1, the following conclusions on the functionality of mammalian ALOX15B orthologs can be drawn: *i*) ALOX15B orthologs carrying the Asp+Val (DV), Asp-Met (DM), Asp+Ile (DI) and HA motifs at the Jisaka positions oxygenate AA predominantly to 15S-HETE. Thus, the majority of mammalian ALOX15B orthologs constitute AA15-lipoxygenating enzymes. *ii*) The ALOX15B orthologs of *M. pahari*, *M. caroli,* and *M. coucha,* which carry the Tyr+His (YH) motif, may catalyze major AA 8S-lipoxygenation. *iii*) If these conclusions are correct the reaction specificity of mammalian ALOX15B orthologs does not depend on the evolutionary ranking of the animals as it is the case for mammalian ALOX15 orthologs (23, 24).

**Fig. 5:**
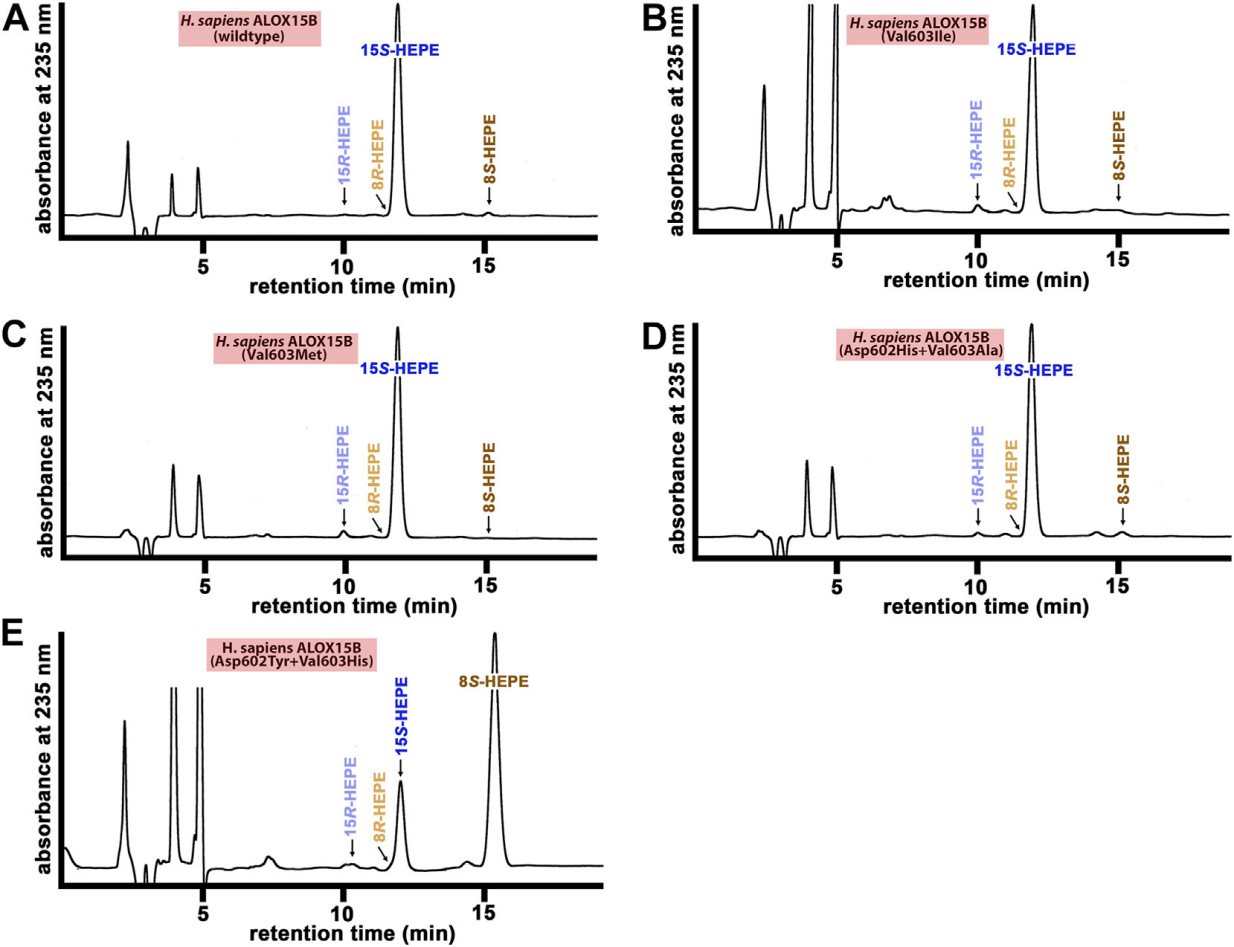
NP/CP-HPLC analysis of the EPA oxygenation products formed during in vitro activity assays by relevant human ALOX15B mutants. Wild-type human ALOX15B and the different Jisaka mutants were expressed in *E. coli* and in vitro activity assays with EPA as substrate were carried out. The major conjugated dienes were prepared by RP-HPLC and further analyzed by NP/CP-HPLC. A: Product pattern of EPA oxygenation by wild-type human ALOX15B (DV motif). B: Product pattern of EPA oxygenation by the V603I mutant (DI motif). C: Product pattern of EPA oxygenation by the V603M mutant (DM motif). D: Product pattern of EPA oxygenation by the D602H+V603A mutant (HA motif). E: Product pattern of EPA oxygenation by the D602H-V603Y mutant (HY motif). ALOX, arachidonic acid lipoxygenase.

To explore whether mutation of the Jisaka determinants might also modify the pattern of EPA oxygenation products, we carried out in vitro activity assays using EPA as substrate. As expected, for wild-type human ALOX15B 15S-HEPE was the major EPA oxygenation products (Fig. 5A). Val603Ile exchange (DI motif at the Jisaka positions) did not modify the pattern of EPA oxygenation products (Fig. 5B) and similar results (Fig. 5C+D) were obtained for the Val603Met mutant (DM motif at the Jisaka positions) and for the Asp602His+Val603Ala double mutant (HA motif at the Jisaka positions). The major EPA oxygenation product of the Asp602His+Val603Tyr double mutant (HY motif at the Jisaka determinants) was also 8S-HEPE, but this enzyme mutant did also produce significant amounts of 15S-HETE (Fig. 5E). In other words, the reaction specificity of the relevant Jisaka mutants was similar when AA and EPA were used as substrate. Interestingly, the degree of murinization of the reaction specificity of the Asp602His+Val603Tyr double mutant (HY motif at the Jisaka positions) was more complete for EPA oxygenation than with AA oxygenation. In fact, the relative share of 8S-HEPE formation (75%) by the mutant enzyme (Fig. 5E) was higher than that of 8-HETE (50%) formation (Fig. 4J+K).

### Mammalian ALOX15B orthologs are expressed in different quantities in *E. coli*

Although the mutagenesis studies on human ALOX15B provided a solid basis for the prediction of the catalytic properties of other mammalian ALOX15B orthologs, direct functional data are needed to proof the predictions. To achieve this goal, we next expressed selected mammalian ALOX15B orthologs as recombinant enzymes and explored their functional characteristics.

**Fig. 6:**
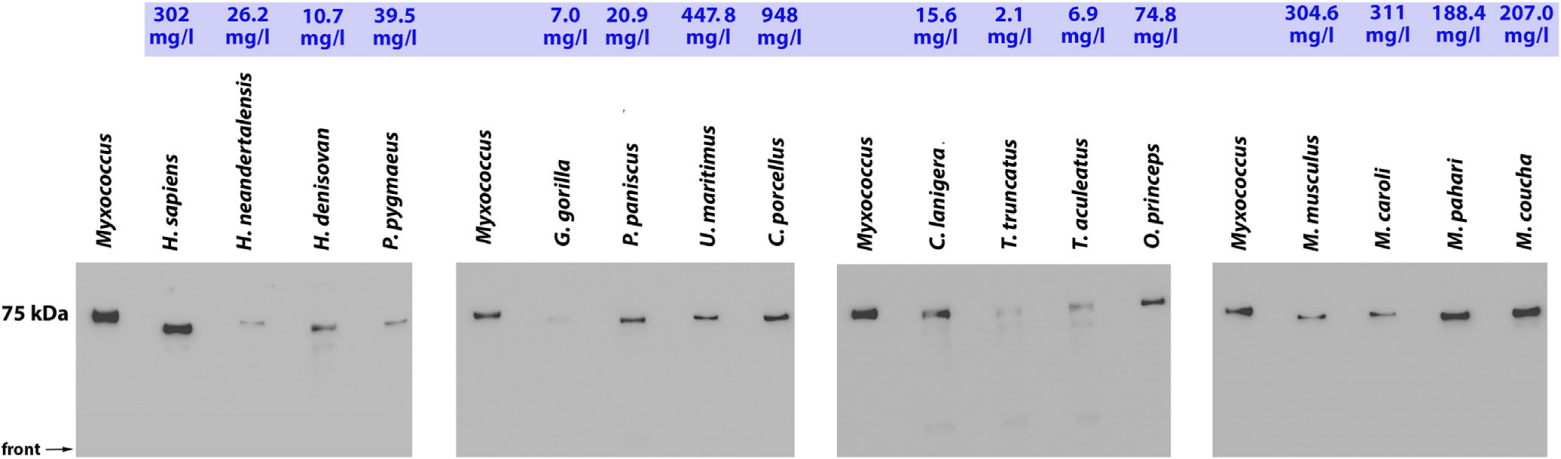
Western blot analysis of recombinantly expressed mammalian ALOX15B orthologs. Selected mammalian ALOX15B orthologs were expressed in *E. coli,* and aliquots of the stroma-free cell lysate supernatants were applied for SDS-PAGE. Protein bands were transferred to a nitrocellulose membrane and the blots were stained with an anti-his-tag antibody. The intensities of the immunoreactive bands were quantified (see Materials and Methods) and the expression levels of the different ALOX15B isoforms were calculated in milligrams recombinant his-tag fusion protein per liter bacterial liquid culture (blue numbers above the electropherograms). Since different volumes of the bacterial lysate supernatants were applied for the different enzyme preparations the band intensities do not directly mirror the expression levels. ALOX, arachidonic acid lipoxygenase.

Most mammalian ALOX15B orthologs could be expressed in *E. coli* and for these enzymes the expression levels were quantified by quantitative Western blotting (see Materials and methods). Although all mammalian ALOX15B orthologs share a high degree of amino acid identity and although we optimized the codon usage for bacterial expression, we observed big differences in the expression efficiency of the different enzymes. As indicated in Figure 6, ALOX15B orthologs of *Cavia porcellus* (guinea pig), *Ursus maritimus* (polar beat), *M. musculus* (house mouse), and *Homo sapiens* (modern human) were expressed at high levels (>300 mg his-tag fusion protein per liter bacterial liquid culture). In contrast, only low-level expression (<10 mg his-tag fusion protein per liter bacterial liquid culture) was observed for the ALOX15B orthologs of *Tursiops truncates* (bottlenose dolphin) and *Gorilla gorilla* (gorilla). We also expressed the ALOX15B orthologs of two extinct human subspecies (*H. neanderthalensis, H. denisovan*) and their expression levels were also low.

The ALOX15B orthologs of platypus (*O. anatinus*), star mole rat (*C. cristata*), wild bacterian camel (*Camelus ferus*), dromedary (*Camelus dromedarius*), and blue whale (*Balaenoptera musculus*) were not well expressed in *E. coli* and thus eucaryotic expression systems (HEK cells, Sf9 cells) were used to express these proteins (Table 1). Although we did not quantify the expression levels of the enzymes, in vitro activity assays indicated ALOX15B activities in the cellular lysate supernatants. No ALOX15B activities were observed when HEK cells were transfected with nonrecombinant expression plasmids and when Sf9 cells were infected with a nonrecombinant baculovirus. Interestingly, the ALOX15B ortholog of the star mole rat (*C. cristata,* representative of *Laurasiatheria*), which carries the HA motif at the Jisaka positions, was well expressed in Sf9 cells and could be purified by affinity chromatography on Ni-agarose. However, in vitro activity assays using the purified recombinant protein as catalyst indicated that the enzyme was catalytically inactive. The molecular basis for the catalytical silence was not explored.

### Functional characterization of mammalian ALOX15B orthologs confirmed the structure-based predictions on the reaction specificity of these enzymes

**Fig. 7:**
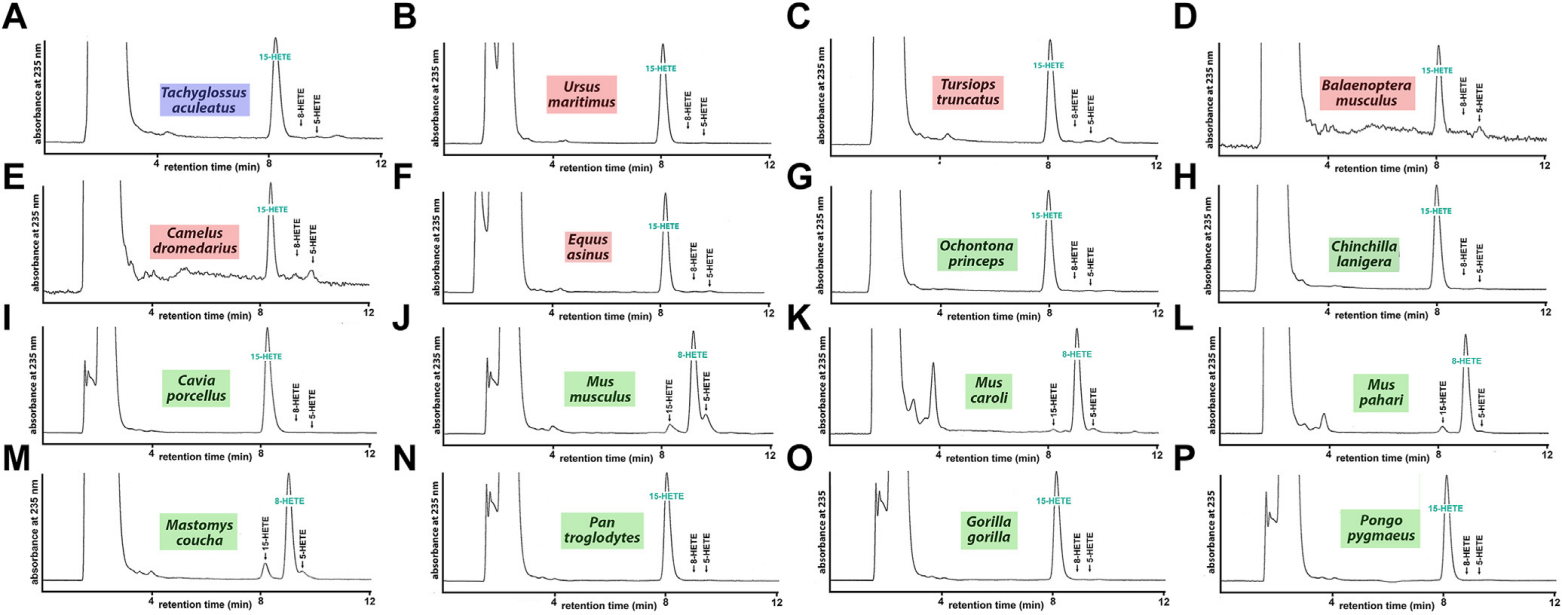
RP-HPLC analysis of the AA oxygenation products formed during in vitro activity assays by different mammalian ALOX15B orthologs. Wild-type mammalian ALOX15B orthologs were expressed and in vitro activity assays were performed using the cellular lysate supernatants as enzyme source. Human ALOX15B and the different double mutant at the Jisaka positions were expressed in *E. coli*. In vitro activity assays and HPLC analysis of the reaction products were carried out as described in Materials and methods. The chemical identity of the oxygenation products was concluded from the retention times of the major conjugated dienes formed during the activity assays. Prototherian ALOX15B orthologs are labeled in blue, laurasiatherian ALOX15B orthologs are labeled in red, and euarchontoglirian ALOX15B orthologs are labeled in green. The different panels (A–P) give representative RP-HPLC chromatograms of the oxygenation products formed by the ALOX15B orthologs of different mammalian species, which are indicated by the colored boxes above the traces. AA, arachidonic acid; ALOX, arachidonic acid lipoxygenase.

We recently (33) characterized the ALOX15B orthologs of two extant *Prototheria* (platypus, short-beaked echidna). Both enzymes carry Asp+Ile (DI) motif at the Jisaka positions (Table 1) and thus AA 15S-lipoxygenation was concluded. In vitro activity assays with the recombinant enzyme expressed in Sf9 cells confirmed this functional conclusion (33). For the present study, we re-expressed the short-beaked echidna (*T. aculeatus)* ALOX15B in *E. coli* and confirmed dominant formation of 15-HETE (Fig. 7A). Next, we also expressed the ALOX15B orthologs of five different *Laurasiatheria* and all of them carried either the Asp+Ile (DI), the Asp+Val (DV), the Asp+Met (DM), or the His+Ala (HA) motif at the Jisaka positions (Table 1). Thus, an AA 15S-lipoxygenating activity was predicted for these enzyme orthologs and this prediction was confirmed by in vitro activity assays (Fig. 7B–F). Similar results were obtained for the euarchontoglirian ALOX15B orthologs of the American pika (*Ochotona princeps,* Fig. 7G), of the long-tailed chinchilla (*Ceratovacuna lanigera,* Fig. 7H) and of the guinea pig (*Cavia porcellus*, Fig. 7I). As reported before (29), the ALOX15B ortholog of house mouse (*M. musculus*), which carries the Tyr+His motif at the Jisaka positions, converts AA to 8-HETE and we confirmed this finding in the present study (Fig. 7J). Other representatives of the genus *Mus,* such as *M. caroli* (Fig. 7K) and *M. pahari* (Fig. 7L) but also a representative of the related genus *Mastomys* (*M. coucha,* Fig. 7M) also expresses AA 8-lipoxygenating enzyme variants.

As expected from the sequence data, this is not the case for the ALOX15B orthologs of *Hominidae*. In fact, the enzymes of chimpanzees (*Pan troglodytes,* Fig. 7N), of gorillas (*G. gorilla,* Fig. 7O) and of orangutans (*Pongo pygmaeus,* Fig. 7P) constitute AA 15-lipoxygenating enzyme variants and these findings are consistent with our observation that these ALOX15B orthologs carry either the Asp+Val or the Asp+Ile motif at the Jisaka positions (Table 1).

**Fig. 8:**
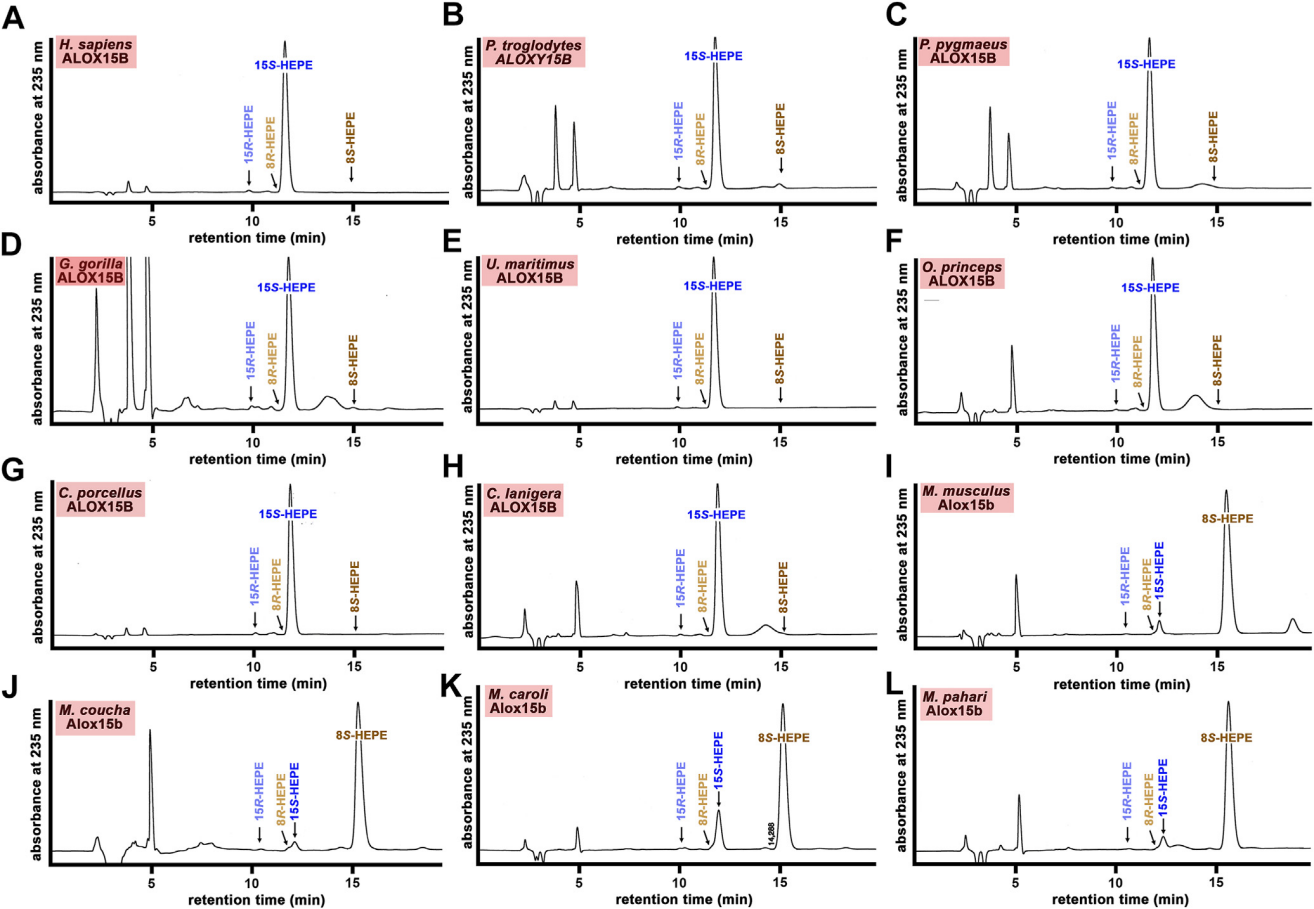
NP/CP HPLC analysis of the EPA oxygenation products formed by mammalian ALOX15B orthologs. Different mammalian ALOX15B orthologs were expressed in *E. coli* and in vitro activity assays were carried out using EPA as substrate. The different panels (A–L) show representative chromatograms and the mammalian species are indicated. ALOX, arachidonic acid lipoxygenase; NP/CP, normal-phase/chiral-phase.

To explore whether the reaction specificities of the ALOX15B orthologs with EPA as substrate are similar than those of AA oxygenation, we also performed in vitro activity assays using EPA as substrate and analyzed the product patterns. Here, we found the AA 15-lipoxygenating ALOX15B orthologs convert EPA almost exclusively to 15S-HEPE (Fig. 8A–H). In contrast, the AA 8-lipoxygenating enzymes (Fig. 8I–L) synthesize 8S-HEPE as dominant EPA oxygenation product. 15S-HETE was also detected as minor EPA oxygenation product (5–10%) but for ALOX15B ortholog of *M. caroli* the share of 8S-HETE formation (Fig. 8K) was somewhat higher (15–20%).

AA and EPA have similar structures. The only structural difference is that EPA involves an additional *cis-*double bond between C17 and C18, which is lacking in AA. Because of this structural similarity it was not surprising that all mammalian ALOX15B orthologs oxygenate EPA with similar reaction specificity as AA. However, the structures of AA and DHA are more different. DHA has a longer hydrocarbon chain (C20 in AA versus C22 in DHA), involves more double bonds (4 double bonds in AA versus 6 double bonds in AA) and the double bonds are located at different positions (5,8,11,14 in AA versus 4,7,10,13,16,18 in DHA). Because of these structural differences it is difficult to predict the reaction specificities of mammalian ALOX15B orthologs with DHA as substrate from the pattern of AA oxygenation products. To explore the reaction specificity of mammalian ALOX15B orthologs with DHA, we performed additional in vitro activity assays with DHA as substrate and analyzed the product pattern by RP-HPLC. As indicated in Table 2, for all ALOX15B orthologs oxygenating AA to 15-HETE, 17-hydroxy-4Z,7Z,10Z,13Z,15E,19Z-docosahexaenoic acid (HDHA) was identified as dominant DHA oxygenation product. This includes the AA 15-lipoxygenating mutants of human ALOX15B. Interestingly, the Asp602Tyr+Val603His double mutant of human ALOX15B, which catalyzed dominant AA 8-lipoxygenation, exhibited a low-catalytic activity with DHA and thus reliable product analysis was not possible. For the AA 8-lipoxygenating enzymes, a mixture of 10-HDHA and 17-HDHA was observed (Table 2). 10-HDHA was always the major (two-thirds) DHA oxygenation product but 17-HDHA contributed about 30% to the sum of the DHA oxygenation products.

**Table 2:** Major DHA oxygenation products formed by mammalian ALOX15B orthologs

Species	AA Oxygenation Product	17-HDHA (%)	10-HDHA (%)
*H. sapiens* wildtype	15-HETE	100	0
*H. sapiens* V603I	15-HETE	100	0
*H. sapiens* V603M	15-HETE	100	0
*H. sapiens D602H+V603A*	15-HETE	95.3	4.7
*P. troglodytes*	15-HETE	100	0
*P. pygmaeus*	15-HETE	100	0
*G. gorilla*	15-HETE	100	0
*C. porcellus*	15-HETE	100	0
*C. lanigera*	15-HETE	100	0
*O. princeps*	15-HETE	100	0
*U. maritimus*	15-HETE	100	0
*M. musculus*	8-HETE	28.7	71.3
*M. pahari*	8-HETE	27.9	72.1
*M. caroli*	8-HETE	33.5	66.5
*M. coucha*	8-HETE	34.8	65.2

AA, arachidonic acid; DHA, docosahexaenoic acid.

Randomly selected mammalian ALOX15B orthologs were expressed and in vitro activity assays were performed using DHA (100 μM) as substrate. After lipid extraction the pattern of DHA oxygenation products were analyzed by RP-HPLC as described in the Materials and methods section. The chemical identity of the oxygenation products was concluded from the retention times of the major conjugated dienes formed during the activity assays.

### Reaction specificity of ALOX15B orthologs of extinct human subspecies

**Fig. 9:**
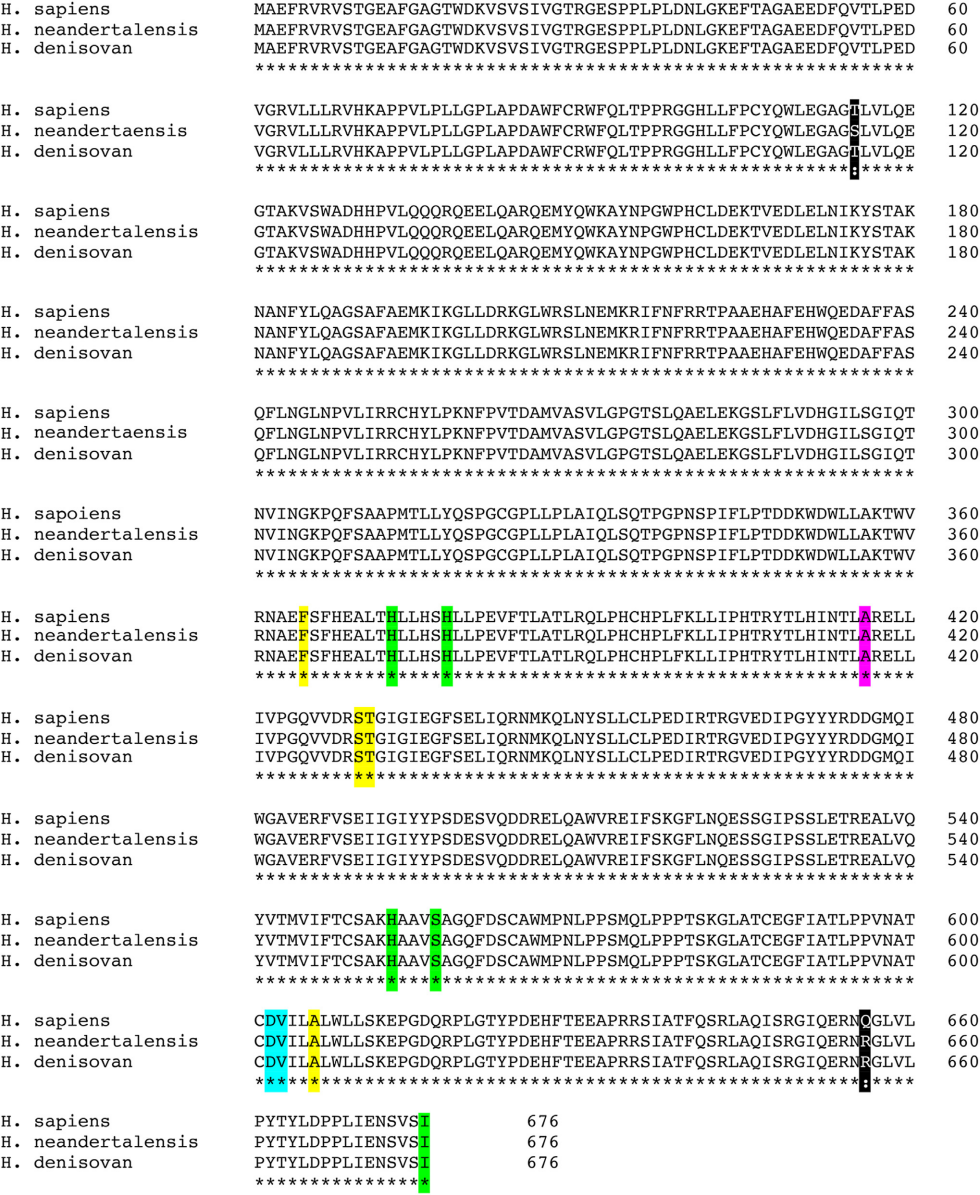
Multiple amino acid sequence alignments of the ALOX15B orthologs of *H. sapiens*, *H. neanderthalensis,* and *H. denisovan*. The following color code was used for specification of functionally relevant amino acids: green: proteinogenic iron ligand; yellow: triad determinants; red: Coffa/Brash determinants; and blue: Jisaka determinants. The amino acid sequence differences between the three enzymes are indicated in black. ALOX, arachidonic acid lipoxygenase.

In the genomes of extinct human subspecies (*H. neanderthalensis, H. denisovan*) ALOX15 orthologs have previously been identified and functional characterization of the recombinant enzymes indicated dominant AA 15-lipoxygenation (24, 34). When we searched the genomes of extinct human subspecies for ALOX15B orthologs, we also detected functional *ALOX15B* genes and an amino acid sequence alignments of the encoded proteins (Fig. 9) indicated that the three enzymes share a very high degree of amino acid conservation. We only observed two amino acid exchanges (Thr115Ser and Gln656Arg) when *H. sapiens* ALOX15B and the *H. neanderthalensis* ortholog were compared. Comparing the ALOX15B amino acid sequences of *H. sapiens* and *H. denisovan,* a single amino acid exchange (Gl656Arg) was identified. Interestingly, Arg656, which is a Gln in the *H. sapiens* sequence, was conserved between *H. neandertalensis* and *H. denisovan* ALOX15B. However, Gln656Arg exchange in human ALOX15B did not modify the reaction specificity of the mutant enzyme. From these sequence data it was concluded that the ALOX15B orthologs of *H. neanderthalensis* and *H. denisovan* should oxygenate AA almost exclusively to 15-HETE.

**Fig. 10:**
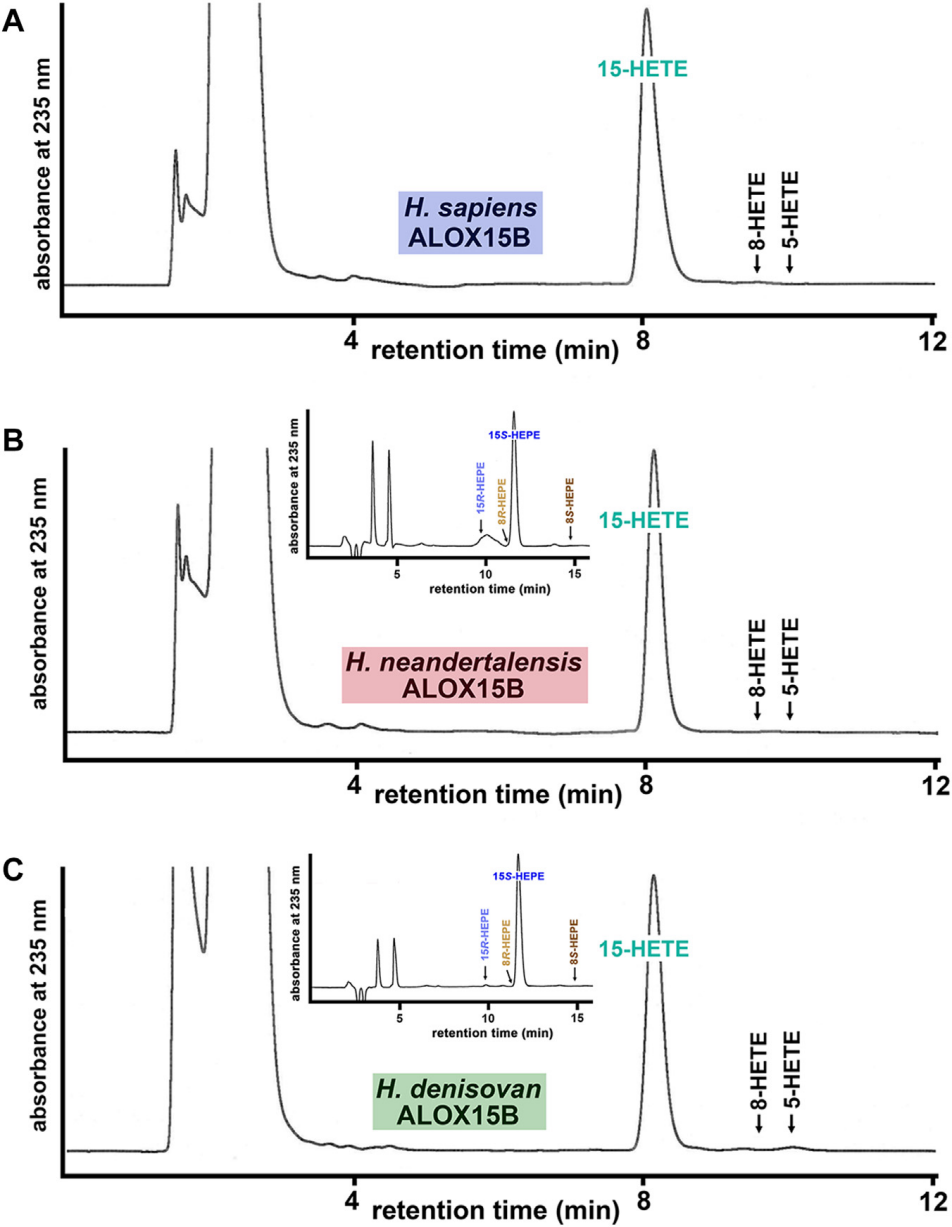
AA oxygenation products formed during in vitro activity assays by human ALOX15B orthologs. Wild-type ALOX15B orthologs of *H. sapiens*, *H. neanderthalensis,* and *H. denisovan* were expressed in *E. coli* and in vitro activity assays were performed using the bacterial lysate supernatants as enzyme source (see Materials and Methods). In vitro activity assays and HPLC analysis of the reaction products were carried out as described in Materials and methods. A: *H. sapiens* ALOX15B with AA as substrate. B: *H. neanderthalensis* ALOX15B with AA as substrate. C: *H. denisovan* ALOX15B with AA as substrate. Insets to panels B+C: Additional in vitro activity assays were carried out with EPA as substrate, and the reaction products were prepared by RP-HPLC and further analyzed by combined NP/CP-HPLC (see Materials and Methods). AA, arachidonic acid; ALOX, arachidonic acid lipoxygenase.

To confirm the functional predictions made on the basis of the amino acid sequences, the three proteins were expressed in *E. coli* as N-terminal his-tag fusion proteins and in vitro activity assays were carried out with AA as oxygenation substrate. As indicated in Figure 10, 15-HETE was the dominant AA oxygenation product for the three ALOX15B orthologs and other positional HETE isomers were only present in trace amounts. For the *H. sapiens* ALOX15B ortholog, our results confirm previous finding (15, 29), but for the enzyme orthologs of the two extinct human subspecies these data are novel.

## Discussion

### Degree of novelty and advancement of science

Human ALOX15B was first described in 1997 as AA 15S-lipoxygenting enzyme and because of its functional similarity with human ALOX15 (15-LOX) it was named human 15-LOX2 (15). In the following years, the enzyme was well characterized with respect to its enzymatic properties (17, 18) and its crystal structure was solved in 2014 (35). It has been implicated in a large number of physiological processes but also in the pathogenesis of various diseases (17, 18). A similar enzyme was described in phorbol ester-treated mouse skin (16, 28) but since it oxygenated AA almost exclusively to 8S-HETE, the evolutionary relatedness between the two ALOX isoforms was not immediately recognized. However, later sequence data indicated a high degree of amino acid identity between the two enzymes (29) and sequence comparison of the human and mouse genomes suggested that human 15-LOX2 (ALOX15B) and mouse 8-LOX (Alox15b) were enzyme orthologs. Unfortunately, hardly any structural and functional information is currently available on ALOX15B orthologs of other mammalian species. Recent studies indicated that these enzymes frequently occur in fish (36, 37) and the genomes of extant *Prototheria* do also involve functional ALOX15B genes (33). Here, we report for the first time that functional ALOX15B genes frequently occur in the genomes of *Eutheria* and that most of the encoded enzymes are functional. We also identified single copy ALOX15B genes in the genomes of extinct human subspecies and expressed the corresponding enzyme as functional proteins.

Mouse and human ALOX15 orthologs exhibit different reaction specificities with free PUFA (24, 25, 38). In early days of lipoxygenase research, these findings prompted the conclusions that the AA 12-lipoxygenating (leukocyte-type 12-LOX) and AA 15-lipoxygenating (reticulocyte-type 15-LOX) ALOX15 orthologs might simultaneously occur in a given mammalian species. Today, we know that this is not the case. In the genomes of a large number of mammalian species, only one single copy *ALOX15* gene is present and in the vast majority (>90%) of mammals including mice this gene encodes for an AA 12-lipoxygenating enzyme variant (23, 24). However, in modern humans (38) and in extinct human subspecies (34), the *ALOX15* gene encodes for an AA 15-lipoxygenating enzyme. A single amino acid exchange (Leu353Phe) at the active site humanized the reactions specificity of mouse Alox15 in vitro and in vivo and an inverse mutagenesis strategy murinized the human enzyme (39). According to the evolutionary hypothesis of ALOX15 specificity (23, 24) mammals ranked in evolution below gibbons including nonhuman primates (32, 40) but also cattle (41), mice (25), and rats (26, 27) express AA 12-lipoxygenating ALOX15 orthologs. In contrast, *Hominidae* including human, orangutans, gorillas, and chimpanzees, express AA 15-lipoxygenating ALOX15 orthologs. In other words, the reaction specificity of mammalian ALOX15 orthologs depends on evolutionary ranking of the animals (23, 24).

Mouse and human ALOX15B orthologs do also exhibit different reaction specificities (15, 29) and the molecular basis for this difference is an exchange of two consecutive amino acids at the active site of the two enzymes (29). However, it has not been explored in the past whether this functional difference is also a consequence of a targeted enzyme evolution as it has been suggested for mammalian ALOX15 orthologs (23, 24). The structural basis for the different reaction specificities of mouse and human ALOX15B have been explored (29), the biological consequences of these differences are far from clear. Our study did not contribute to answer these questions but we provided the following structural and functional information on mammalian ALOX15 orthologs, which may help to answer this question in the future. *i*) ALOX15B orthologs are expressed in a large number but not in all *Eutheria*. *ii*) Functional *ALOX15B* genes are also present in *Prototheria* but the rarely occur in the metatherian species we explored. *iii*) The Jisaka determinants are not only important for the reaction specificity of human and mouse ALOX15B orthologs [this has been shown before (29)] but also for the new ALOX15B orthologs characterized in our study. *iv*) The difference in the reaction specificity of mouse and human ALOX15B orthologs are not a functional consequence of a targeted evolutionary process as it has been suggested for mammalian ALOX15 orthologs. *v*) Among eutherian ALOX15B orthologs only the enzymes of some but not of all *Muridae* express AA 8S-lipoxygenating enzymes.

### Biological roles of ALOX15B orthologs and potential relevance of the reaction specificities

In principle, ALOX isoforms exhibit their biological activities via three different mechanistic scenarios (2): *i*) Formation of bioactive oxylipins, which function as signaling molecules: Such lipid mediators are released by the synthesizing cells, bind at cell surface receptors of target cells and induce intracellular signaling cascades that modify the cellular phenotype (42, 43). For receptor binding, a defined chemical structure of the ligands is required and thus the reaction specificity of the biosynthesizing enzymes is of major functional relevance. AA 15-lipoxygeating ALOX isoforms biosynthesize different oxylipins than AA 8-lipoxygenating enzymes and thus mouse and human ALOX15B orthologs are likely to induce different functional alterations in target cells.

*ii*) Structural and functional modifications of complex lipid-protein assemblies such as biomembranes and lipoproteins: ALOX catalyzed oxygenation of the PUFAs esterified in the ester lipids of biomembranes and lipoproteins alter the structure of these lipid-protein assemblies and thus their biological properties. ALOX15 catalyzed oxygenation of biomembranes has been implicated in cell differentiation (10, 12) and functional silencing of the Alox15 gene in mice induced a defective erythropoietic phenotype (11). ALOX15 catalyzed oxygenation of LDLs renders this lipid transport particle atherogenic (44, 45) and thus ALOX15 has been implicated in the pathogenesis of atherosclerosis (46, 47). ALOX15B is also capable of oxygenating complex ester lipids when these substrates are offered to the enzyme in form of nanodiscs (48). Interestingly, in that study, it was reported that the product pattern of human ALOX15B- and mouse Alox15b-catalyzed reactions are similar and esterified 15-HETE was identified as major oxygenation product for both enzymes (48). Unfortunately, comparative studies of mouse and human ALOX15B orthologs with native biomembranes and lipoproteins have not been carried out. However, the functional consequences of membrane phospholipid oxygenation may not dramatically depend on the reaction specificity of the enzymes. In other words, it might not be important whether a given enzyme forms 15-HETE–containing, 12-HETE–containing, or 8-HETE–containing phospholipids. In all cases, the hydrophobic interactions within the membrane phospholipid bilayer are likely to be disturbed in a similar way and thus the functional consequences should also be similar.

*iii*) Modification of the cellular redox state: Since ALOX isoforms oxygenate their substrates to hydroperoxy derivative the ALOX reaction leads to an increase in the intracellular oxidation potential. In this mechanistic scenario, it may also be irrelevant whether 15-HpETE (human ALOX15B, human ALOX15) or 8-HpETE (mouse Alox15b) or 12-HpETE (mouse Alox15, human, and mouse ALOX12) is formed as major reaction product since all these peroxy lipids exhibit similar redox properties. ALOX-induced oxidative stress may regulate the intracellular activity of redox-sensitive transcription factors and/or redox-sensitive protein kinases (49, 50) and thus alters the gene expression pattern of the cells. In other words, for this mechanistic scenario, the different positional specificities of mouse and human ALOX15B orthologs may neither be of biological relevance.

Human ALOX15B has previously been implicated in atherogenesis (51, 52) but the detailed pathophysiological enzyme functions have not been specified. If the enzyme is involved in oxidative modification of LDL (46), the difference in the reaction specificity between human and mouse ALOX15 may not be of major relevance. Unfortunately, the reactivities of human and mouse ALOX15B orthologs with LDL have never been compared and thus it remains unclear whether the two enzymes have similar LDL-oxidizing activities. Since atherosclerosis is an inflammatory disease of the vessel wall (53), ALOX15B orthologs might also impact progression of the disease via the formation of pro-inflammatory and/or anti-inflammatory mediators. In this scenario, the different catalytic properties of human and mouse Alox15 might well be of pathophysiological relevance. Transgenic mice, which express the AA 15-lipoxygenating human ALOX15 under the control of the lysozyme promoter (macrophage specific expression) in addition to the endogenous AA 12-lipoxygenating mouse Alox15, are protected from arterial lipid deposition in a mouse atherosclerosis model. This protective effect correlated with increased levels of pro-resolving mediators such as lipoxin isomers (54). The transgenic AA 15-lipoxygenating human ALOX15B may also contribute to lipoxin biosynthesis but the endogenous AA 8S-lipoxygenating mouse Alox15b may not. In fact, primary AA 8S-lipoxygenation does actually prevent lipoxin biosynthesis.

### Limitations of the study and suggestions for further research directions

Mouse Alox15b exhibits a different reaction specificity with free PUFAs as substrate than its human ortholog and this is also the case for the corresponding enzymes of other species of the genus *Mus* (*M. pahari, M. paroli*). The ALOX15B ortholog of *M. coucha* and *A. niloticus*, which belong to another genus (*Mastomys, Arvicanthis*) of *Muridae* exhibits a similar reaction specificity as the *M. musculus* Alox15B. Unfortunately, it remains unclear at the moment whether the ALOX15B orthologs of other representatives of the genus *Mus* or other evolutionary categories of *Muridae* exhibit a *M. musculus*–like or a *H. sapiens*–like reaction specificity. The ALOX15B ortholog of different rats (Table 1), which are also classified as *Muridae*, exhibit a *H. sapiens*–like reaction specificity and these data indicate that not all *Muridae* exhibit a *M. musculus*–like reaction specificity.

Our data show that the ALOX15B orthologs of some *Muridea* species exhibit a different reaction specificity with free PUFA than the vast majority of mammalian ALOX15B orthologs (Table 1). However, the data do not answer the question why several *Muridae* species express AA 8S-lipoxygenating, whereas the majority of these enzymes is AA 15-lipoxygenating. One possible answer of this question is that the reaction specificity with free PUFAs may not be important for the biological function of these enzymes. Since ALOX15B orthologs are also capable of oxygenating complex ester lipids it might be much more important which products are formed from such complex substrates. In a more recent study (48), it has been shown that both human and mouse ALOX15B convert AA-containing phospholipids mainly to 15-HETE–containing ester lipids. Thus, in this experimental model system, there was no difference in the reaction specificity of mouse and human ALOX15B orthologs. Unfortunately, it has not been tested whether this might also be the case for naturally occurring complex lipid-protein assemblies such as biomembranes and lipoproteins. Corresponding experiments are underway in our group and preliminary data suggest that the reaction specificity of mammalian ALOX15B orthologs with complex substrates is rather variable and strongly depends on the chemical composition of complex substrates.

### Data availability

The experimental raw data obtained in this study can be obtained upon request from E. G. and H. K.

## Supplemental data

This article contains supplemental data.

## Conflict of interest

The authors declare that they have no conflicts of interest with the contents of this article.

## Supplementary data

Supplemental images
