# Supplementary material for: The reaction specificity of mammalian ALOX15B orthologs does not depend on the evolutionary ranking of the animals
Source: J Lipid Res. 2025 Mar 3;66(4):100768. doi: 10.1016/j.jlr.2025.100768 (PMC11999201; doi:10.1016/j.jlr.2025.100768)
Supplement: Supplemental images [file mmc1.docx]

Short-beaked echidna (*Tachyglossus aculeatus*)

H.sapiens 1 MAEFRVRVSTGEAFGAGTWDKVSVSIVGTRGESPPLPLDNLGKEFTAGAE 50

||.:.||:.||...||||||.:|:|:||:.|:||||.||..||:|:.|||

T.aculeatus 1 MAVYGVRIITGGFLGAGTWDHISISLVGSEGKSPPLHLDTCGKDFSRGAE 50

H.sapiens 51 EDFQVTLPEDVGRVLLLRVHKAPPVL--PLLGPLAPDAWFCRWFQLTPPR 98

|:|.| ..|.||.:|.|::||||..| ||..||.||||||....|.||.

T.aculeatus 51 EEFTV-CSEPVGSLLFLQLHKAPLWLPTPLPLPLPPDAWFCSSVHLIPPE 99

H.sapiens 99 GGHLLFPCYQWLEGAGTLVLQEGTAKVSWADHHPVLQQQRQEELQARQEM 148

|..|.||||||||||.||||:|||||::.:|..|:|.:||:.|||.||..

T.aculeatus 100 GPPLNFPCYQWLEGACTLVLREGTAKLASSDTVPLLLEQRRGELQKRQGT 149

H.sapiens 149 YQWKAYNPGWPHCLDEKTVEDLELNIKYSTAKNANFYLQAGSAFAEMKIK 198

|:||.|.||||||:|.:.||:|..::.||..|:.:|:|:|.|||.::|:|

T.aculeatus 150 YRWKTYAPGWPHCVDVEAVEELSPDVHYSLGKSISFFLKASSAFIKLKMK 199

H.sapiens 199 GLLDRKGLWRSLNEMKRIFNFRRTPAAEHAFEHWQEDAFFASQFLNGLNP 248

|||:....|:.|.::::||:.::||..|:..|||:||..|||||||||||

T.aculeatus 200 GLLNDASPWKDLKDLRQIFSIQKTPVYEYVVEHWKEDELFASQFLNGLNP 249

H.sapiens 249 VLIRRCHYLPKNFPVTDAMVASVLGPGTSLQAELEKGSLFLVDHGILSGI 298

:||:||..||.:|||||||||..||.||||.|||::|::||||:.:|.||

T.aculeatus 250 ILIQRCRQLPPHFPVTDAMVAPFLGRGTSLDAELKRGTMFLVDYKLLDGI 299

H.sapiens 299 QTNVINGKPQFSAAPMTLLYQSPGCGPLLPLAIQLSQTPGPNSPIFLPTD 348

....||||.|:.|||:.||.|.|| .||.|:||||||||||:||||||:|

T.aculeatus 300 PAGQINGKQQYVAAPLCLLQQPPG-RPLRPIAIQLSQTPGPDSPIFLPSD 348

H.sapiens 349 DKWDWLLAKTWVRNAEFSFHEALTHLLHSHLLPEVFTLATLRQLPHCHPL 398

.:||||.||||||:|||:.|||::||..:|||||||::|||||||.||||

T.aculeatus 349 SEWDWLTAKTWVRSAEFAIHEAVSHLGRAHLLPEVFSIATLRQLPLCHPL 398

H.sapiens 399 FKLLIPHTRYTLHINTLARELLIVPGQVVDRSTGIGIEGFSELIQRNMKQ 448

:||||||||||||||.|||:|||.||.||.|||.:|:.||:||::|:::|

T.aculeatus 399 YKLLIPHTRYTLHINVLARQLLISPGGVVQRSTAVGVSGFTELVRRDLEQ 448

H.sapiens 449 LNYSLLCLPEDIRTRGVEDIPGYYYRDDGMQIWGAVERFVSEIIGIYYPS 498

|.||.||||||:|.|||.::|||:|||||::||||:|.:||||:|.||.|

T.aculeatus 449 LTYSTLCLPEDVRARGVSNLPGYHYRDDGLKIWGAIESYVSEIVGFYYGS 498

H.sapiens 499 DESVQDDRELQAWVREIFSKGFLNQESSGIPSSLETREALVQYVTMVIFT 548

|.:|:.|.||||||.|||::|||.:|:||:|.|||:|.||::|:||||||

T.aculeatus 499 DAAVEGDMELQAWVWEIFAEGFLGRETSGVPPSLESRAALIRYLTMVIFT 548

H.sapiens 549 CSAKHAAVSAGQFDSCAWMPNLPPSMQLPPPTSKGLATCEGFIATLPPVN 598

|||:|||.||||||..|||||.||||||||||:||||..|.|:|:||.:|

T.aculeatus 549 CSAQHAAFSAGQFDFSAWMPNFPPSMQLPPPTAKGLAGPESFLASLPDIN 598

H.sapiens 599 ATCDVILALWLLSKEPGDQRPLGTYPDEHFTEEAPRRSIATFQSRLAQIS 648

||||:|:.||||||||||.|.||.:||.||||||||||...|.:|||:||

T.aculeatus 599 ATCDIIIVLWLLSKEPGDLRSLGCFPDAHFTEEAPRRSQEAFAARLAEIS 648

H.sapiens 649 RGIQERNQGLVLPYTYLDPPLIENSVSI 676

|.|||||..|.|||||||||.|||||:|

T.aculeatus 649 RQIQERNARLPLPYTYLDPPNIENSVAI 676

Platypus (*Ornithorhynchus anatinus*)

H.sapiens 1 MAEFRVRVSTGEAFGAGTWDKVSVSIVGTRGESPPLPLDNLGKEFTAGAE 50

||.:||||.||...||||||.:|:|:||:.|:||||.||..||:|:.|||

O.anatinus 1 MAVYRVRVITGGFMGAGTWDHISISLVGSEGKSPPLYLDACGKDFSRGAE 50

H.sapiens 51 EDFQVTLPEDVGRVLLLRVHKA----PPVLPLLGPLAPDAWFCRWFQLTP 96

|:|.:...|.||.:|.|.:||| ||.||| ||.||||||....|||

O.anatinus 51 EEFTILCSEPVGSLLFLHLHKAPLAFPPPLPL--PLPPDAWFCCSVHLTP 98

H.sapiens 97 PRGGHLLFPCYQWLEGAGTLVLQEGTAKVSWADHHPVLQQQRQEELQARQ 146

|.|..:.||||||||||.||.|:|||||:..:|..|:|.:||..|||.||

O.anatinus 99 PEGPPMNFPCYQWLEGACTLALREGTAKLVSSDTVPLLLEQRSGELQKRQ 148

H.sapiens 147 EMYQWKAYNPGWPHCLDEKTVEDLELNIKYSTAKNANFYLQAGSAFAEMK 196

..|:||.|.||||.|:|.:|||:|..::.||..|:.||||:|.|||.::|

O.anatinus 149 GTYRWKTYTPGWPRCVDVETVEELSPDVHYSLGKSINFYLKASSAFIKLK 198

H.sapiens 197 IKGLLDRKGLWRSLNEMKRIFNFRRTPAAEHAFEHWQEDAFFASQFLNGL 246

:||||:....|:.|.::|:||:.::||..|:..|||:||..|||||||||

O.anatinus 199 MKGLLEEASPWKDLKDLKQIFSIQKTPVYEYVVEHWKEDELFASQFLNGL 248

H.sapiens 247 NPVLIRRCHYLPKNFPVTDAMVASVLGPGTSLQAELEKGSLFLVDHGILS 296

||||||||..||.:||||:||||.:||..|||.|||::|::||||:.:|.

O.anatinus 249 NPVLIRRCSQLPPHFPVTNAMVAPLLGGRTSLDAELKRGTMFLVDYKLLD 298

H.sapiens 297 GIQTNVINGKPQFSAAPMTLLYQSPGCGPLLPLAIQLSQTPGPNSPIFLP 346

||....||||.|..|||:.||:|..| .||.|:||||||||||:||||||

O.anatinus 299 GIPAGQINGKQQHVAAPLCLLHQPLG-RPLRPIAIQLSQTPGPDSPIFLP 347

H.sapiens 347 TDDKWDWLLAKTWVRNAEFSFHEALTHLLHSHLLPEVFTLATLRQLPHCH 396

:|.:||||.||||||:|||:.|||:|||.|:|||||||:||||||||.||

O.anatinus 348 SDSEWDWLTAKTWVRSAEFAIHEAVTHLGHAHLLPEVFSLATLRQLPLCH 397

H.sapiens 397 PLFKLLIPHTRYTLHINTLARELLIVPGQVVDRSTGIGIEGFSELIQRNM 446

|::|:||||||||||||.|||:|||....||.|||.:|:.|||||::|::

O.anatinus 398 PVYKMLIPHTRYTLHINVLARQLLISLDGVVQRSTAVGVSGFSELVRRDL 447

H.sapiens 447 KQLNYSLLCLPEDIRTRGVEDIPGYYYRDDGMQIWGAVERFVSEIIGIYY 496

:||.||.||||||::||||.|:|||:|||||::||||:|.:||||:|.||

O.anatinus 448 EQLTYSTLCLPEDVQTRGVSDLPGYHYRDDGLKIWGAIESYVSEIVGFYY 497

H.sapiens 497 PSDESVQDDRELQAWVREIFSKGFLNQESSGIPSSLETREALVQYVTMVI 546

.||.:|:.|.||||||.|||::|||.:|:||||||||:|..|::|:||||

O.anatinus 498 LSDAAVKGDLELQAWVWEIFTEGFLGRETSGIPSSLESRATLIRYLTMVI 547

H.sapiens 547 FTCSAKHAAVSAGQFDSCAWMPNLPPSMQLPPPTSKGLATCEGFIATLPP 596

|||||:|||.|.||||..|||||.|||||||||.:||||..:.|:||||.

O.anatinus 548 FTCSAQHAAFSTGQFDFSAWMPNFPPSMQLPPPKAKGLADPQSFLATLPD 597

H.sapiens 597 VNATCDVILALWLLSKEPGDQRPLGTYPDEHFTEEAPRRSIATFQSRLAQ 646

||.|||:|:.|||||||||||||||::||.||||||||||...|.:||.:

O.anatinus 598 VNTTCDIIIVLWLLSKEPGDQRPLGSFPDAHFTEEAPRRSQEAFAARLGE 647

H.sapiens 647 ISRGIQERNQGLVLPYTYLDPPLIENSVSI 676

||:.|||||..|.|||||||||.|||||:|

O.anatinus 648 ISQQIQERNAHLPLPYTYLDPPNIENSVAI 677

Yellow-footed antechinus (*Antechinus flavipes*)

H.sapiens 1 MAEFRVRVSTGEAFGAGTWDKVSVSIVGTRGESPPLPLDNLGKEFTAGAE 50

||.:.|:||||...|..:||.:|||:|||.|||.||.|.|.||:|..|.|

A.flavipes 1 MAIYIVKVSTGSFLGVVSWDNISVSLVGTEGESLPLKLGNFGKDFNQGVE 50

H.sapiens 51 EDFQVTLPEDVGRVLLLRVHKAPPVLPLLGPLAPDAWFCRWFQLTPPRGG 100

|||:|.:.:|||..|||::|.|.|..|....|.||||||:..||..|:..

A.flavipes 51 EDFEVKILQDVGSELLLQIHNAYPEQPKSSSLNPDAWFCQELQLISPKNP 100

H.sapiens 101 HLLFPCYQWLEGAGTLVLQEGTAKVSWADHHPVLQQQRQEELQARQEMYQ 150

.|.|||||||||..:||:::|.|||.||:..|:|.:||..||:.||:.|:

A.flavipes 101 PLHFPCYQWLEGTESLVIRDGAAKVIWAESSPILLEQRHLELKRRQQKYR 150

H.sapiens 151 WKAYNPGWPHCLDEKTVEDLELNIKYSTAKNANFYLQAGSAFAE---MKI 197

|..|.||.|||||.|.:::|:.|::.||.||......||.:|.. :|:

A.flavipes 151 WNEYFPGLPHCLDAKNLKELDPNLRSSTNKNVLSPPSAGLSFCRQMGLKM 200

H.sapiens 198 KGLLDRKGLWRSLNEMKRIFNFRRTPAAEHAFEHWQEDAFFASQFLNGLN 247

|.:|||||.|.|::|:..||.|.::..:|:..|:||:|.|||||||||||

A.flavipes 201 KRMLDRKGPWESMDEICEIFAFLKSEVSEYVAEYWQDDEFFASQFLNGLN 250

H.sapiens 248 PVLIRRCHYLPKNFPVTDAMVASVLGPGTSLQAELEKGSLFLVDHGILSG 297

||||:.||.||.|||||:.|||.:||||||||.|||||||:||:|.:|||

A.flavipes 251 PVLIQCCHSLPDNFPVTNNMVAPLLGPGTSLQEELEKGSLYLVNHSLLSG 300

H.sapiens 298 IQTNVINGKPQFSAAPMTLLYQSPGCGPLLPLAIQLSQTPGPNSPIFLPT 347

:...:|:|:||:.|||||||:|.|..|||||:||||:||||||:|||||:

A.flavipes 301 LSPGLIDGRPQYVAAPMTLLHQKPDGGPLLPIAIQLNQTPGPNNPIFLPS 350

H.sapiens 348 DDKWDWLLAKTWVRNAEFSFHEALTHLLHSHLLPEVFTLATLRQLPHCHP 397

|.:.|||||||||||:||..||.:||||.:|.:.|||.:||:||||.|||

A.flavipes 351 DSEEDWLLAKTWVRNSEFLVHEMVTHLLCTHFISEVFAIATMRQLPMCHP 400

H.sapiens 398 LFKLLIPHTRYTLHINTLARELLIVPGQVVDRSTGIGIEGFSELIQRNMK 447

:|||||||..:|.||||:.|..|..||.::|:||.:|..|..||:.|.||

A.flavipes 401 VFKLLIPHFHFTFHINTIGRMDLFKPGGLIDKSTALGHVGCLELVARGMK 450

H.sapiens 448 QLNYSLLCLPEDIRTRGVEDIPGYYYRDDGMQIWGAVERFVSEIIGIYYP 497

.|.|..||||.::..|.|:.:..|:|||||:|||.|||||||.||.||||

A.flavipes 451 MLTYRSLCLPHNLADREVQGLANYHYRDDGLQIWDAVERFVSNIIDIYYP 500

H.sapiens 498 SDESVQDDRELQAWVREIFSKGFLNQESSGIPSSLETREALVQYVTMVIF 547

.|.||..|.|||:||:|||.:|||.||.||||:.|||.:.|||::||:||

A.flavipes 501 EDRSVFQDSELQSWVQEIFVEGFLGQECSGIPTCLETLQDLVQFLTMIIF 550

H.sapiens 548 TCSAKHAAVSAGQFDSCAWMPNLPPSMQLPPPTSKGLATCEGFIATLPPV 597

..||:||||::||||..||:||:|.||:||||.:||.| .|:.:|..|

A.flavipes 551 NSSAQHAAVNSGQFDFSAWVPNIPTSMRLPPPITKGTA---DFLGSLGDV 597

H.sapiens 598 NATCDVILALWLLSKEPGDQRPLGTYPDEHFTEEAPRRSIATFQSRLAQI 647

:..|..::..|.:|.:..|.|.|||||:.|||||||::|||.|||||.||

A.flavipes 598 STACHALILFWGVSDQDRDMRLLGTYPEVHFTEEAPKQSIAAFQSRLVQI 647

H.sapiens 648 SRGIQERNQGLVLPYTYLDPPLIENSVSI 676

|:.|||||:||.||||||||..|||||:|

A.flavipes 648 SQDIQERNKGLDLPYTYLDPLHIENSVAI 676

Tasmanian devil (*Sarcophilus harrisii*)

H.sapiens 1 MAEFRVRVSTGEAFGAGTWDKVSVSIVGTRGESPPLPLDNLGKEFTAGAE 50

||.:.||||||...|..:||.:|:|::||.|||.||.|.|.||:|..|.|

S.harrisii 1 MAIYIVRVSTGSFLGVVSWDNISISLLGTEGESLPLKLGNFGKDFNQGVE 50

H.sapiens 51 EDFQVTLPEDVGRVLLLRVHKAPPVLPLLGPLAPDAWFCRWFQLTPPRGG 100

|||:|.:.:|||..|||::|.|.|..|....|.||||||:..||..|:..

S.harrisii 51 EDFEVKILQDVGSELLLQIHNAYPEQPKSSSLNPDAWFCQELQLISPKNP 100

H.sapiens 101 HLLFPCYQWLEGAGTLVLQEGTAKVSWADHHPVLQQQRQEELQARQEMYQ 150

.|.|||||||||..:|||:||.|||.||:..|:|.:||:.||:.||:.|:

S.harrisii 101 PLHFPCYQWLEGTESLVLREGAAKVIWAESSPILLEQRRLELKQRQQKYR 150

H.sapiens 151 WKAYNPGWPHCLDEKTVEDLELNIKYSTAKNA---------NFYLQAGSA 191

|..|.||.|||||.|.:::|:.|::..|:||. :|..|.|

S.harrisii 151 WSEYFPGLPHCLDAKNLKELDPNLRSLTSKNVLSSPSAGRLSFCSQMG-- 198

H.sapiens 192 FAEMKIKGLLDRKGLWRSLNEMKRIFNFRRTPAAEHAFEHWQEDAFFASQ 241

:|:||:|||||.|.|::|:..||.|.::..:|:..||||:|.|||||

S.harrisii 199 ---LKMKGMLDRKGPWESMDEISEIFAFLKSEVSEYVAEHWQDDEFFASQ 245

H.sapiens 242 FLNGLNPVLIRRCHYLPKNFPVTDAMVASVLGPGTSLQAELEKGSLFLVD 291

||||||||||:.||.||.|||||:.|||.:||||||||.|||||||:|||

S.harrisii 246 FLNGLNPVLIQCCHSLPDNFPVTNNMVAPLLGPGTSLQEELEKGSLYLVD 295

H.sapiens 292 HGILSGIQTNVINGKPQFSAAPMTLLYQSPGCGPLLPLAIQLSQTPGPNS 341

|.:|||:...:|:|:||:.|||||||:|.|..|||||:||||:||||||:

S.harrisii 296 HSLLSGLSPGLIDGRPQYVAAPMTLLHQKPDGGPLLPIAIQLNQTPGPNN 345

H.sapiens 342 PIFLPTDDKWDWLLAKTWVRNAEFSFHEALTHLLHSHLLPEVFTLATLRQ 391

|||||:|.:.|||||||||||:||..||.:||||.:|.:.|||.:||:||

S.harrisii 346 PIFLPSDSEEDWLLAKTWVRNSEFLVHEMVTHLLCTHFISEVFAIATMRQ 395

H.sapiens 392 LPHCHPLFKLLIPHTRYTLHINTLARELLIVPGQVVDRSTGIGIEGFSEL 441

||.|||:|| ||.:|..|..||

S.harrisii 396 LPMCHPVFK-----------------------------STALGHLGCLEL 416

H.sapiens 442 IQRNMKQLNYSLLCLPEDIRTRGVEDIPGYYYRDDGMQIWGAVERFVSEI 491

:.|.||.|.|..||||.::..|||:.:..|:|||||:|||

S.harrisii 417 VARGMKILTYRSLCLPHNLADRGVQGLANYHYRDDGLQIW---------- 456

H.sapiens 492 IGIYYPSDESVQDDRELQAWVREIFSKGFLNQESSGIPSSLETREALVQY 541

|||:.|||.:.|||:

S.harrisii 457 -----------------------------------GIPTCLETLQDLVQF 471

H.sapiens 542 VTMVIFTCSAKHAAVSAGQFDSCAWMPNLPPSMQLPPPTSKGLATCEGFI 591

:||:||..||:||||::||||..||:||:|.||:||||.:||.| .|:

S.harrisii 472 LTMIIFNSSAQHAAVNSGQFDFSAWVPNIPTSMRLPPPITKGTA---DFL 518

H.sapiens 592 ATLPPVNATCDVILALWLLSKEPGD------------------------- 616

.:|..|:..|..::..|::|.:..|

S.harrisii 519 GSLGDVSTACHALILFWVVSDQDRDMVSQAVSYWDGGNQSVRELGVQIWG 568

H.sapiens 617 -----------------QRPLGTYPDEHFTEEAPRRSIATFQSRLAQISR 649

||.|||||:.|||||||::|||.|||||.|||:

S.harrisii 569 RKGEPQIPQFHLFLHYSQRLLGTYPEVHFTEEAPKQSIAAFQSRLVQISQ 618

H.sapiens 650 GIQERNQGLVLPYTYLDPPLIENSVSI 676

.|||||:||.||||||||..|||||:|

S.harrisii 619 DIQERNKGLDLPYTYLDPLHIENSVAI 645

African elephant (*Loxodonta africana*)

H.sapiens 1 MAEFRVRVSTGEAFGAGTWDKVSVSIVGTRGESPPLPLDNLGKEFTAGAE 50

||.||:|||||||||||||||||||||||||||||||||:|||||.||||

L.africana 1 MATFRLRVSTGEAFGAGTWDKVSVSIVGTRGESPPLPLDHLGKEFNAGAE 50

H.sapiens 51 EDFQVTLPEDVGRVLLLRVHKAPPVLPL-LGPLAPDAWFCRWFQLTPPRG 99

|||:||:|:|||.||||||||||..||. ||||.||||||||||||||.|

L.africana 51 EDFEVTVPQDVGPVLLLRVHKAPLALPCSLGPLVPDAWFCRWFQLTPPGG 100

H.sapiens 100 GHLLFPCYQWLEGAGTLVLQEGTAK------------VSWADHHPVLQQQ 137

..|.|||||||||||:|||:|||.. ...|::.|....

L.africana 101 ATLRFPCYQWLEGAGSLVLREGTGDGGNGQGTLLDFLKPLANYLPAPPS- 149

H.sapiens 138 RQEELQARQEMYQWKAYNPGWPHCLDEKTVEDLELNIKYSTAKNANFYLQ 187

.||.|||||||||:|||.:||:||||||||||.||||:

L.africana 150 ------------SWKIYNPGWPHCLNEKTTKDLDLNIKYSTAKNTNFYLR 187

H.sapiens 188 AGSAFAEMKIKGLLDRKGLWRSLNEMKRIFNFRRTPAAEHAFEHWQEDAF 237

..|||||||||||||||..|:||.||:|:||||:|||||:.|.||:||||

L.africana 188 GISAFAEMKIKGLLDRKESWKSLKEMRRVFNFRKTPAAEYVFAHWREDAF 237

H.sapiens 238 FASQFLNGLNPVLIRRCHYLPKNFPVTDAMVASVLGPGTSLQAELEKGSL 287

||||||||||||||.||..|||||||||||||.|||||||||||||:|||

L.africana 238 FASQFLNGLNPVLIHRCRSLPKNFPVTDAMVAPVLGPGTSLQAELERGSL 287

H.sapiens 288 FLVDHGILSGIQTNVINGKPQFSAAPMTLLYQSPGCGPLLPLAIQLSQTP 337

:||||||||.::||||||:|||||||||||||.||.||||||||||||.|

L.africana 288 YLVDHGILSRVRTNVINGRPQFSAAPMTLLYQRPGDGPLLPLAIQLSQAP 337

H.sapiens 338 GPNSPIFLPTDDKWDWLLAKTWVRNAEFSFHEALTHLLHSHLLPEVFTLA 387

||:||||||:|..|||||||||||:||||.|||||||||:||:||||.||

L.africana 338 GPDSPIFLPSDSPWDWLLAKTWVRHAEFSVHEALTHLLHAHLVPEVFALA 387

H.sapiens 388 TLRQLPHCHPLFKLLIPHTRYTLHINTLARELLIVPGQVVDRSTGIGIEG 437

||||||.|||||||||||.|||||||||||||||.|.||||||||:|..|

L.africana 388 TLRQLPRCHPLFKLLIPHIRYTLHINTLARELLIAPNQVVDRSTGLGTGG 437

H.sapiens 438 FSELIQRNMKQLNYSLLCLPEDIRTRGVEDIPGYYYRDDGMQIWGAVERF 487

|||||||||:|||||.||||||||||||||||||||||||:|||||||||

L.africana 438 FSELIQRNMEQLNYSTLCLPEDIRTRGVEDIPGYYYRDDGIQIWGAVERF 487

H.sapiens 488 VSEIIGIYYPSDESVQDDRELQAWVREIFSKGFLNQESSGIPSSLETREA 537

||||||||||||.||:||.||||||.||||:|||.:||||:||:|||:||

L.africana 488 VSEIIGIYYPSDVSVRDDSELQAWVWEIFSEGFLGRESSGMPSTLETQEA 537

H.sapiens 538 LVQYVTMVIFTCSAKHAAVSAGQFDSCAWMPNLPPSMQLPPPTSKGLATC 587

||||||||||.|||||:::|.||||..||||||||:||||||||||.||.

L.africana 538 LVQYVTMVIFNCSAKHSSISMGQFDFSAWMPNLPPTMQLPPPTSKGQATP 587

H.sapiens 588 EGFIATLPPVNATCDVILALWLLSKEPGDQRPLGTYPDEHFTEEAPRRSI 637

|||:|||||||||||:|:.|||||||||..||||:||:|:||||||:|||

L.africana 588 EGFVATLPPVNATCDIIVTLWLLSKEPGAPRPLGSYPEENFTEEAPQRSI 637

H.sapiens 638 ATFQSRLAQISRGIQERNQGLVLPYTYLDPPLIENSVSI 676

|.|||.||||||.||:|||||.|||||:|||||||||:|

L.africana 638 AAFQSHLAQISRDIQKRNQGLALPYTYMDPPLIENSVAI 676

Neanderthal (*Homo neanderthalensis*)

H.sapiens 1 MAEFRVRVSTGEAFGAGTWDKVSVSIVGTRGESPPLPLDNLGKEFTAGAE 50

||||||||||||||||||||||||||||||||||||||||||||||||||

H.neanderthal. 1 MAEFRVRVSTGEAFGAGTWDKVSVSIVGTRGESPPLPLDNLGKEFTAGAE 50

H.sapiens 51 EDFQVTLPEDVGRVLLLRVHKAPPVLPLLGPLAPDAWFCRWFQLTPPRGG 100

||||||||||||||||||||||||||||||||||||||||||||||||||

H.neanderthal. 51 EDFQVTLPEDVGRVLLLRVHKAPPVLPLLGPLAPDAWFCRWFQLTPPRGG 100

H.sapiens 101 HLLFPCYQWLEGAGTLVLQEGTAKVSWADHHPVLQQQRQEELQARQEMYQ 150

||||||||||||||:|||||||||||||||||||||||||||||||||||

H.neanderthal. 101 HLLFPCYQWLEGAGSLVLQEGTAKVSWADHHPVLQQQRQEELQARQEMYQ 150

H.sapiens 151 WKAYNPGWPHCLDEKTVEDLELNIKYSTAKNANFYLQAGSAFAEMKIKGL 200

||||||||||||||||||||||||||||||||||||||||||||||||||

H.neanderthal. 151 WKAYNPGWPHCLDEKTVEDLELNIKYSTAKNANFYLQAGSAFAEMKIKGL 200

H.sapiens 201 LDRKGLWRSLNEMKRIFNFRRTPAAEHAFEHWQEDAFFASQFLNGLNPVL 250

||||||||||||||||||||||||||||||||||||||||||||||||||

H.neanderthal. 201 LDRKGLWRSLNEMKRIFNFRRTPAAEHAFEHWQEDAFFASQFLNGLNPVL 250

H.sapiens 251 IRRCHYLPKNFPVTDAMVASVLGPGTSLQAELEKGSLFLVDHGILSGIQT 300

||||||||||||||||||||||||||||||||||||||||||||||||||

H.neanderthal. 251 IRRCHYLPKNFPVTDAMVASVLGPGTSLQAELEKGSLFLVDHGILSGIQT 300

H.sapiens 301 NVINGKPQFSAAPMTLLYQSPGCGPLLPLAIQLSQTPGPNSPIFLPTDDK 350

||||||||||||||||||||||||||||||||||||||||||||||||||

H.neanderthal. 301 NVINGKPQFSAAPMTLLYQSPGCGPLLPLAIQLSQTPGPNSPIFLPTDDK 350

H.sapiens 351 WDWLLAKTWVRNAEFSFHEALTHLLHSHLLPEVFTLATLRQLPHCHPLFK 400

||||||||||||||||||||||||||||||||||||||||||||||||||

H.neanderthal. 351 WDWLLAKTWVRNAEFSFHEALTHLLHSHLLPEVFTLATLRQLPHCHPLFK 400

H.sapiens 401 LLIPHTRYTLHINTLARELLIVPGQVVDRSTGIGIEGFSELIQRNMKQLN 450

||||||||||||||||||||||||||||||||||||||||||||||||||

H.neanderthal. 401 LLIPHTRYTLHINTLARELLIVPGQVVDRSTGIGIEGFSELIQRNMKQLN 450

H.sapiens 451 YSLLCLPEDIRTRGVEDIPGYYYRDDGMQIWGAVERFVSEIIGIYYPSDE 500

||||||||||||||||||||||||||||||||||||||||||||||||||

H.neanderthal. 451 YSLLCLPEDIRTRGVEDIPGYYYRDDGMQIWGAVERFVSEIIGIYYPSDE 500

H.sapiens 501 SVQDDRELQAWVREIFSKGFLNQESSGIPSSLETREALVQYVTMVIFTCS 550

||||||||||||||||||||||||||||||||||||||||||||||||||

H.neanderthal. 501 SVQDDRELQAWVREIFSKGFLNQESSGIPSSLETREALVQYVTMVIFTCS 550

H.sapiens 551 AKHAAVSAGQFDSCAWMPNLPPSMQLPPPTSKGLATCEGFIATLPPVNAT 600

||||||||||||||||||||||||||||||||||||||||||||||||||

H.neanderthal. 551 AKHAAVSAGQFDSCAWMPNLPPSMQLPPPTSKGLATCEGFIATLPPVNAT 600

H.sapiens 601 CDVILALWLLSKEPGDQRPLGTYPDEHFTEEAPRRSIATFQSRLAQISRG 650

||||||||||||||||||||||||||||||||||||||||||||||||||

H.neanderthal. 601 CDVILALWLLSKEPGDQRPLGTYPDEHFTEEAPRRSIATFQSRLAQISRG 650

H.sapiens 651 IQERNQGLVLPYTYLDPPLIENSVSI 676

|||||:||||||||||||||||||||

H.neanderthal. 651 IQERNRGLVLPYTYLDPPLIENSVSI 676

Denisovan (*Homo denisovan*)

H.sapiens 1 MAEFRVRVSTGEAFGAGTWDKVSVSIVGTRGESPPLPLDNLGKEFTAGAE 50

||||||||||||||||||||||||||||||||||||||||||||||||||

H.denisovan 1 MAEFRVRVSTGEAFGAGTWDKVSVSIVGTRGESPPLPLDNLGKEFTAGAE 50

H.sapiens 51 EDFQVTLPEDVGRVLLLRVHKAPPVLPLLGPLAPDAWFCRWFQLTPPRGG 100

||||||||||||||||||||||||||||||||||||||||||||||||||

H.denisovan 51 EDFQVTLPEDVGRVLLLRVHKAPPVLPLLGPLAPDAWFCRWFQLTPPRGG 100

H.sapiens 101 HLLFPCYQWLEGAGTLVLQEGTAKVSWADHHPVLQQQRQEELQARQEMYQ 150

||||||||||||||||||||||||||||||||||||||||||||||||||

H.denisovan 101 HLLFPCYQWLEGAGTLVLQEGTAKVSWADHHPVLQQQRQEELQARQEMYQ 150

H.sapiens 151 WKAYNPGWPHCLDEKTVEDLELNIKYSTAKNANFYLQAGSAFAEMKIKGL 200

||||||||||||||||||||||||||||||||||||||||||||||||||

H.denisovan 151 WKAYNPGWPHCLDEKTVEDLELNIKYSTAKNANFYLQAGSAFAEMKIKGL 200

H.sapiens 201 LDRKGLWRSLNEMKRIFNFRRTPAAEHAFEHWQEDAFFASQFLNGLNPVL 250

||||||||||||||||||||||||||||||||||||||||||||||||||

H.denisovan 201 LDRKGLWRSLNEMKRIFNFRRTPAAEHAFEHWQEDAFFASQFLNGLNPVL 250

H.sapiens 251 IRRCHYLPKNFPVTDAMVASVLGPGTSLQAELEKGSLFLVDHGILSGIQT 300

||||||||||||||||||||||||||||||||||||||||||||||||||

H.denisovan 251 IRRCHYLPKNFPVTDAMVASVLGPGTSLQAELEKGSLFLVDHGILSGIQT 300

H.sapiens 301 NVINGKPQFSAAPMTLLYQSPGCGPLLPLAIQLSQTPGPNSPIFLPTDDK 350

||||||||||||||||||||||||||||||||||||||||||||||||||

H.denisovan 301 NVINGKPQFSAAPMTLLYQSPGCGPLLPLAIQLSQTPGPNSPIFLPTDDK 350

H.sapiens 351 WDWLLAKTWVRNAEFSFHEALTHLLHSHLLPEVFTLATLRQLPHCHPLFK 400

||||||||||||||||||||||||||||||||||||||||||||||||||

H.denisovan 351 WDWLLAKTWVRNAEFSFHEALTHLLHSHLLPEVFTLATLRQLPHCHPLFK 400

H.sapiens 401 LLIPHTRYTLHINTLARELLIVPGQVVDRSTGIGIEGFSELIQRNMKQLN 450

||||||||||||||||||||||||||||||||||||||||||||||||||

H.denisovan 401 LLIPHTRYTLHINTLARELLIVPGQVVDRSTGIGIEGFSELIQRNMKQLN 450

H.sapiens 451 YSLLCLPEDIRTRGVEDIPGYYYRDDGMQIWGAVERFVSEIIGIYYPSDE 500

||||||||||||||||||||||||||||||||||||||||||||||||||

H.denisovan 451 YSLLCLPEDIRTRGVEDIPGYYYRDDGMQIWGAVERFVSEIIGIYYPSDE 500

H.sapiens 501 SVQDDRELQAWVREIFSKGFLNQESSGIPSSLETREALVQYVTMVIFTCS 550

||||||||||||||||||||||||||||||||||||||||||||||||||

H.denisovan 501 SVQDDRELQAWVREIFSKGFLNQESSGIPSSLETREALVQYVTMVIFTCS 550

H.sapiens 551 AKHAAVSAGQFDSCAWMPNLPPSMQLPPPTSKGLATCEGFIATLPPVNAT 600

||||||||||||||||||||||||||||||||||||||||||||||||||

H.denisovan 551 AKHAAVSAGQFDSCAWMPNLPPSMQLPPPTSKGLATCEGFIATLPPVNAT 600

H.sapiens 601 CDVILALWLLSKEPGDQRPLGTYPDEHFTEEAPRRSIATFQSRLAQISRG 650

||||||||||||||||||||||||||||||||||||||||||||||||||

H.denisovan 601 CDVILALWLLSKEPGDQRPLGTYPDEHFTEEAPRRSIATFQSRLAQISRG 650

H.sapiens 651 IQERNQGLVLPYTYLDPPLIENSVSI 676

|||||:||||||||||||||||||||

H.denisovan 651 IQERNRGLVLPYTYLDPPLIENSVSI 676

Gorilla (*Gorilla gorilla*)

H.sapiens 1 MAEFRVRVSTGEAFGAGTWDKVSVSIVGTRGESPPLPLDNLGKEFTAGAE 50

|:||||||||||||||||||||||||||||||||||||||||||||||||

G.gorilla 1 MSEFRVRVSTGEAFGAGTWDKVSVSIVGTRGESPPLPLDNLGKEFTAGAE 50

H.sapiens 51 EDFQVTLPEDVGRVLLLRVHKAPPVLPLLGPLAPDAWFCRWFQLTPPRGG 100

||||||.|||||||||||||||||||||||||||||||||||||||||||

G.gorilla 51 EDFQVTFPEDVGRVLLLRVHKAPPVLPLLGPLAPDAWFCRWFQLTPPRGG 100

H.sapiens 101 HLLFPCYQWLEGAGTLVLQEGTAKVSWADHHPVLQQQRQEELQARQEMYQ 150

|||||||||||||||||||||||||||||||||||.||||||||||||||

G.gorilla 101 HLLFPCYQWLEGAGTLVLQEGTAKVSWADHHPVLQLQRQEELQARQEMYQ 150

H.sapiens 151 WKAYNPGWPHCLDEKTVEDLELNIKYSTAKNANFYLQAGSAFAEMKIKGL 200

||||||||||||||||||||:||||||||||||||||.||||||||||||

G.gorilla 151 WKAYNPGWPHCLDEKTVEDLDLNIKYSTAKNANFYLQTGSAFAEMKIKGL 200

H.sapiens 201 LDRKGLWRSLNEMKRIFNFRRTPAAEHAFEHWQEDAFFASQFLNGLNPVL 250

|||||||||||||||||||||||.||||||||||||||||||||||||||

G.gorilla 201 LDRKGLWRSLNEMKRIFNFRRTPGAEHAFEHWQEDAFFASQFLNGLNPVL 250

H.sapiens 251 IRRCHYLPKNFPVTDAMVASVLGPGTSLQAELEKGSLFLVDHGILSGIQT 300

||||||||||||||||||||||||||||||||||||||||||||||||||

G.gorilla 251 IRRCHYLPKNFPVTDAMVASVLGPGTSLQAELEKGSLFLVDHGILSGIQT 300

H.sapiens 301 NVINGKPQFSAAPMTLLYQSPGCGPLLPLAIQLSQTPGPNSPIFLPTDDK 350

||||||||||.|||||||||||||||||||||||||||||||||||||||

G.gorilla 301 NVINGKPQFSVAPMTLLYQSPGCGPLLPLAIQLSQTPGPNSPIFLPTDDK 350

H.sapiens 351 WDWLLAKTWVRNAEFSFHEALTHLLHSHLLPEVFTLATLRQLPHCHPLFK 400

||||||||||||||||||||||||||||||||||||||||||||||||||

G.gorilla 351 WDWLLAKTWVRNAEFSFHEALTHLLHSHLLPEVFTLATLRQLPHCHPLFK 400

H.sapiens 401 LLIPHTRYTLHINTLARELLIVPGQVVDRSTGIGIEGFSELIQRNMKQLN 450

||||||||||||||||||||||||||||||||||||||||||:|||||||

G.gorilla 401 LLIPHTRYTLHINTLARELLIVPGQVVDRSTGIGIEGFSELIRRNMKQLN 450

H.sapiens 451 YSLLCLPEDIRTRGVEDIPGYYYRDDGMQIWGAVERFVSEIIGIYYPSDE 500

||||||||||||||||||||||||||||||||||||||||||||||||||

G.gorilla 451 YSLLCLPEDIRTRGVEDIPGYYYRDDGMQIWGAVERFVSEIIGIYYPSDE 500

H.sapiens 501 SVQDDRELQAWVREIFSKGFLNQESSGIPSSLETREALVQYVTMVIFTCS 550

||||||||||||||||||||||||||||||||||||||||||||||||||

G.gorilla 501 SVQDDRELQAWVREIFSKGFLNQESSGIPSSLETREALVQYVTMVIFTCS 550

H.sapiens 551 AKHAAVSAGQFDSCAWMPNLPPSMQLPPPTSKGLATCEGFIATLPPVNAT 600

||||||||||||||||||||||||||||||||||||||||||||||||||

G.gorilla 551 AKHAAVSAGQFDSCAWMPNLPPSMQLPPPTSKGLATCEGFIATLPPVNAT 600

H.sapiens 601 CDVILALWLLSKEPGDQRPLGTYPDEHFTEEAPRRSIATFQSRLAQISRG 650

||:|||||||||||||:|||||||||||||||||||||||||||||||||

G.gorilla 601 CDIILALWLLSKEPGDRRPLGTYPDEHFTEEAPRRSIATFQSRLAQISRG 650

H.sapiens 651 IQERNQGLVLPYTYLDPPLIENSVSI 676

|||||:||||||||||||||||||||

G.gorilla 651 IQERNRGLVLPYTYLDPPLIENSVSI 676

Orangutan (*Pongo pygmaeus*)

H.sapiens 1 MAEFRVRVSTGEAFGAGTWDKVSVSIVGTRGESPPLPLDNLGKEFTAGAE 50

||||||||||||||||||||||||||||||||||||||||||||||||||

P.pygmaeus 1 MAEFRVRVSTGEAFGAGTWDKVSVSIVGTRGESPPLPLDNLGKEFTAGAE 50

H.sapiens 51 EDFQVTLPEDVGRVLLLRVHKAPPVLPLLGPLAPDAWFCRWFQLTPPRGG 100

||||||||||||:|||||||||||.|||||||||||||||||||||||||

P.pygmaeus 51 EDFQVTLPEDVGQVLLLRVHKAPPALPLLGPLAPDAWFCRWFQLTPPRGG 100

H.sapiens 101 HLLFPCYQWLEGAGTLVLQEGTAKVSWADHHPVLQQQRQEELQARQEMYQ 150

||||||||||||||:||||:||||||.|||||||||||||||||||.|||

P.pygmaeus 101 HLLFPCYQWLEGAGSLVLQDGTAKVSRADHHPVLQQQRQEELQARQGMYQ 150

H.sapiens 151 WKAYNPGWPHCLDEKTVEDLELNIKYSTAKNANFYLQAGSAFAEMKIKGL 200

||||||||||||||||||||:|||||||||||||||||||||||||||||

P.pygmaeus 151 WKAYNPGWPHCLDEKTVEDLDLNIKYSTAKNANFYLQAGSAFAEMKIKGL 200

H.sapiens 201 LDRKGLWRSLNEMKRIFNFRRTPAAEHAFEHWQEDAFFASQFLNGLNPVL 250

|||||||||||||||||||||||.||||||||||||||||||||||||||

P.pygmaeus 201 LDRKGLWRSLNEMKRIFNFRRTPGAEHAFEHWQEDAFFASQFLNGLNPVL 250

H.sapiens 251 IRRCHYLPKNFPVTDAMVASVLGPGTSLQAELEKGSLFLVDHGILSGIQT 300

||||||||||||||||||||||||||||||||||||||||||||||||||

P.pygmaeus 251 IRRCHYLPKNFPVTDAMVASVLGPGTSLQAELEKGSLFLVDHGILSGIQT 300

H.sapiens 301 NVINGKPQFSAAPMTLLYQSPGCGPLLPLAIQLSQTPGPNSPIFLPTDDK 350

||||||||||||||||||||||||||||||||||||||||||||||||||

P.pygmaeus 301 NVINGKPQFSAAPMTLLYQSPGCGPLLPLAIQLSQTPGPNSPIFLPTDDK 350

H.sapiens 351 WDWLLAKTWVRNAEFSFHEALTHLLHSHLLPEVFTLATLRQLPHCHPLFK 400

||||||||||||||||||||||||||||||||||||||||||||||||||

P.pygmaeus 351 WDWLLAKTWVRNAEFSFHEALTHLLHSHLLPEVFTLATLRQLPHCHPLFK 400

H.sapiens 401 LLIPHTRYTLHINTLARELLIVPGQVVDRSTGIGIEGFSELIQRNMKQLN 450

||||||||||||||||||||||||||||||||||||||||||||||||||

P.pygmaeus 401 LLIPHTRYTLHINTLARELLIVPGQVVDRSTGIGIEGFSELIQRNMKQLN 450

H.sapiens 451 YSLLCLPEDIRTRGVEDIPGYYYRDDGMQIWGAVERFVSEIIGIYYPSDE 500

||||||||||||||||||||||||||||||||||||||||||||||||||

P.pygmaeus 451 YSLLCLPEDIRTRGVEDIPGYYYRDDGMQIWGAVERFVSEIIGIYYPSDE 500

H.sapiens 501 SVQDDRELQAWVREIFSKGFLNQESSGIPSSLETREALVQYVTMVIFTCS 550

:||||||||||||||||||||||||||||||||||||||:||||||||||

P.pygmaeus 501 TVQDDRELQAWVREIFSKGFLNQESSGIPSSLETREALVRYVTMVIFTCS 550

H.sapiens 551 AKHAAVSAGQFDSCAWMPNLPPSMQLPPPTSKGLATCEGFIATLPPVNAT 600

||||||||||||||||||||||||||||||||||||.|||||||||||||

P.pygmaeus 551 AKHAAVSAGQFDSCAWMPNLPPSMQLPPPTSKGLATREGFIATLPPVNAT 600

H.sapiens 601 CDVILALWLLSKEPGDQRPLGTYPDEHFTEEAPRRSIATFQSRLAQISRG 650

||||||||||||||||:|||||||||||||||||||||.||||||||||.

P.pygmaeus 601 CDVILALWLLSKEPGDRRPLGTYPDEHFTEEAPRRSIAAFQSRLAQISRV 650

H.sapiens 651 IQERNQGLVLPYTYLDPPLIENSVSI 676

|||||:||||||||||||||||||||

P.pygmaeus 651 IQERNRGLVLPYTYLDPPLIENSVSI 676

Chimpanzee (*Pan troglodytes*)

H.sapiens 1 MAEFRVRVSTGEAFGAGTWDKVSVSIVGTRGESPPLPLDNLGKEFTAGAE 50

||||||||||||||||||||||||||||||||||||||||||||||||||

P.troglodytes 1 MAEFRVRVSTGEAFGAGTWDKVSVSIVGTRGESPPLPLDNLGKEFTAGAE 50

H.sapiens 51 EDFQVTLPEDVGRVLLLRVHKAPPVLPLLGPLAPDAWFCRWFQLTPPRGG 100

||||||||||||||||||||||||||||||||||||||||||||||||||

P.troglodytes 51 EDFQVTLPEDVGRVLLLRVHKAPPVLPLLGPLAPDAWFCRWFQLTPPRGG 100

H.sapiens 101 HLLFPCYQWLEGAGTLVLQEGTAKVSWADHHPVLQQQRQEELQARQEMYQ 150

||||||||||||||||||||||||||||||||||||||||||||||||||

P.troglodytes 101 HLLFPCYQWLEGAGTLVLQEGTAKVSWADHHPVLQQQRQEELQARQEMYQ 150

H.sapiens 151 WKAYNPGWPHCLDEKTVEDLELNIKYSTAKNANFYLQAGSAFAEMKIKGL 200

|||.||||||||||||||||||||||||||||||||||||||||||||||

P.troglodytes 151 WKACNPGWPHCLDEKTVEDLELNIKYSTAKNANFYLQAGSAFAEMKIKGL 200

H.sapiens 201 LDRKGLWRSLNEMKRIFNFRRTPAAEHAFEHWQEDAFFASQFLNGLNPVL 250

|||||||||||||||||||:|||.||||||||||||||||||||||||||

P.troglodytes 201 LDRKGLWRSLNEMKRIFNFQRTPGAEHAFEHWQEDAFFASQFLNGLNPVL 250

H.sapiens 251 IRRCHYLPKNFPVTDAMVASVLGPGTSLQAELEKGSLFLVDHGILSGIQT 300

||||||||||||||||||||||||||||||||||||||||||||||||||

P.troglodytes 251 IRRCHYLPKNFPVTDAMVASVLGPGTSLQAELEKGSLFLVDHGILSGIQT 300

H.sapiens 301 NVINGKPQFSAAPMTLLYQSPGCGPLLPLAIQLSQTPGPNSPIFLPTDDK 350

||||||||||||||||||||||||||||||||||||||||||||||||||

P.troglodytes 301 NVINGKPQFSAAPMTLLYQSPGCGPLLPLAIQLSQTPGPNSPIFLPTDDK 350

H.sapiens 351 WDWLLAKTWVRNAEFSFHEALTHLLHSHLLPEVFTLATLRQLPHCHPLFK 400

||||||||||||||||||||||||||||||||||||||||||||||||||

P.troglodytes 351 WDWLLAKTWVRNAEFSFHEALTHLLHSHLLPEVFTLATLRQLPHCHPLFK 400

H.sapiens 401 LLIPHTRYTLHINTLARELLIVPGQVVDRSTGIGIEGFSELIQRNMKQLN 450

||||||||||||||||||||||||||||||||||||||||||||||||||

P.troglodytes 401 LLIPHTRYTLHINTLARELLIVPGQVVDRSTGIGIEGFSELIQRNMKQLN 450

H.sapiens 451 YSLLCLPEDIRTRGVEDIPGYYYRDDGMQIWGAVERFVSEIIGIYYPSDE 500

||||||||||||||||||||||||||||||||||||||||||||||||||

P.troglodytes 451 YSLLCLPEDIRTRGVEDIPGYYYRDDGMQIWGAVERFVSEIIGIYYPSDE 500

H.sapiens 501 SVQDDRELQAWVREIFSKGFLNQESSGIPSSLETREALVQYVTMVIFTCS 550

||||||||||||||||||||||||||||||||||||||||||||||||||

P.troglodytes 501 SVQDDRELQAWVREIFSKGFLNQESSGIPSSLETREALVQYVTMVIFTCS 550

H.sapiens 551 AKHAAVSAGQFDSCAWMPNLPPSMQLPPPTSKGLATCEGFIATLPPVNAT 600

||||||||||||||||||||||||||||||||||||||||||||||||||

P.troglodytes 551 AKHAAVSAGQFDSCAWMPNLPPSMQLPPPTSKGLATCEGFIATLPPVNAT 600

H.sapiens 601 CDVILALWLLSKEPGDQRPLGTYPDEHFTEEAPRRSIATFQSRLAQISRG 650

||:|||||||||||||:|||||||||||||||||||||||||||||||||

P.troglodytes 601 CDIILALWLLSKEPGDRRPLGTYPDEHFTEEAPRRSIATFQSRLAQISRG 650

H.sapiens 651 IQERNQGLVLPYTYLDPPLIENSVSI 676

|||||:||||||||||||||||||||

P.troglodytes 651 IQERNRGLVLPYTYLDPPLIENSVSI 676

Anubis baboon (*Papio anubis*)

H.sapiens 1 MAEFRVRVSTGEAFGAGTWDKVSVSIVGTRGESPPLPLDNLGKEFTAGAE 50

||:||||||||:||||||||||||||||||.|||||||||||||||||||

P.anubis 1 MADFRVRVSTGKAFGAGTWDKVSVSIVGTREESPPLPLDNLGKEFTAGAE 50

H.sapiens 51 EDFQVTLPEDVGRVLLLRVHKAPPVLPLLGPLAPDAWFCRWFQLTPPRGG 100

||||||||:|||:|||||||||||.|||||||||||||||||||||||||

P.anubis 51 EDFQVTLPKDVGQVLLLRVHKAPPALPLLGPLAPDAWFCRWFQLTPPRGG 100

H.sapiens 101 HLLFPCYQWLEGAGTLVLQEGTAKVSWADHHPVLQQQRQEELQARQEMYQ 150

.|||||||||||||:|||||||||||.|||||:|||||||||||||||||

P.anubis 101 PLLFPCYQWLEGAGSLVLQEGTAKVSRADHHPLLQQQRQEELQARQEMYQ 150

H.sapiens 151 WKAYNPGWPHCLDEKTVEDLELNIKYSTAKNANFYLQAGSAFAEMKIKGL 200

|||||||||||||::||:||:|||||||||||||||||||||||||||||

P.anubis 151 WKAYNPGWPHCLDKRTVKDLDLNIKYSTAKNANFYLQAGSAFAEMKIKGL 200

H.sapiens 201 LDRKGLWRSLNEMKRIFNFRRTPAAEHAFEHWQEDAFFASQFLNGLNPVL 250

||||||||||||||||||||:|||||||||||||||||||||||||||||

P.anubis 201 LDRKGLWRSLNEMKRIFNFRKTPAAEHAFEHWQEDAFFASQFLNGLNPVL 250

H.sapiens 251 IRRCHYLPKNFPVTDAMVASVLGPGTSLQAELEKGSLFLVDHGILSGIQT 300

||||||||||||||||||||||||||||||||||||||||||||||||.|

P.anubis 251 IRRCHYLPKNFPVTDAMVASVLGPGTSLQAELEKGSLFLVDHGILSGIHT 300

H.sapiens 301 NVINGKPQFSAAPMTLLYQSPGCGPLLPLAIQLSQTPGPNSPIFLPTDDK 350

|||||||||||||||||||||||||||||||||||:||||||||||||||

P.anubis 301 NVINGKPQFSAAPMTLLYQSPGCGPLLPLAIQLSQSPGPNSPIFLPTDDK 350

H.sapiens 351 WDWLLAKTWVRNAEFSFHEALTHLLHSHLLPEVFTLATLRQLPHCHPLFK 400

||||||||||||||||||||||||||||||||||||||||||||||||||

P.anubis 351 WDWLLAKTWVRNAEFSFHEALTHLLHSHLLPEVFTLATLRQLPHCHPLFK 400

H.sapiens 401 LLIPHTRYTLHINTLARELLIVPGQVVDRSTGIGIEGFSELIQRNMKQLN 450

|||||||||||||.||||||||||||||||||||||||||||||||||||

P.anubis 401 LLIPHTRYTLHINMLARELLIVPGQVVDRSTGIGIEGFSELIQRNMKQLN 450

H.sapiens 451 YSLLCLPEDIRTRGVEDIPGYYYRDDGMQIWGAVERFVSEIIGIYYPSDE 500

|||||||||||||||||||||||||||||||.||||||||||||||||||

P.anubis 451 YSLLCLPEDIRTRGVEDIPGYYYRDDGMQIWDAVERFVSEIIGIYYPSDE 500

H.sapiens 501 SVQDDRELQAWVREIFSKGFLNQESSGIPSSLETREALVQYVTMVIFTCS 550

|||||||||||||||||||||||||||||||||||||||||||||||.||

P.anubis 501 SVQDDRELQAWVREIFSKGFLNQESSGIPSSLETREALVQYVTMVIFNCS 550

H.sapiens 551 AKHAAVSAGQFDSCAWMPNLPPSMQLPPPTSKGLATCEGFIATLPPVNAT 600

||||||||||||:|||||||||||||||||||||||.|||||||||||||

P.anubis 551 AKHAAVSAGQFDACAWMPNLPPSMQLPPPTSKGLATREGFIATLPPVNAT 600

H.sapiens 601 CDVILALWLLSKEPGDQRPLGTYPDEHFTEEAPRRSIATFQSRLAQISRG 650

||||||||||||||||:|||||||||||||||||||||||||.||||||.

P.anubis 601 CDVILALWLLSKEPGDRRPLGTYPDEHFTEEAPRRSIATFQSHLAQISRA 650

H.sapiens 651 IQERNQGLVLPYTYLDPPLIENSVSI 676

|||||:||||||||||||||||||||

P.anubis 651 IQERNRGLVLPYTYLDPPLIENSVSI 676

Rhesus macaque (*Macaca mulatta*)

H.sapiens 1 MAEFRVRVSTGEAFGAGTWDKVSVSIVGTRGESPPLPLDNLGKEFTAGAE 50

||:||||||||:|||||||||||||||||:.|||||||||||||||||||

M.mulatta 1 MADFRVRVSTGKAFGAGTWDKVSVSIVGTQEESPPLPLDNLGKEFTAGAE 50

H.sapiens 51 EDFQVTLPEDVGRVLLLRVHKAPPVLPLLGPLAPDAWFCRWFQLTPPRGG 100

||||||||:|||:|||||||||||.|||||||||||||||||||||||||

M.mulatta 51 EDFQVTLPKDVGQVLLLRVHKAPPALPLLGPLAPDAWFCRWFQLTPPRGG 100

H.sapiens 101 HLLFPCYQWLEGAGTLVLQEGTAKVSWADHHPVLQQQRQEELQARQEMYQ 150

.|||||||||||||:|||||||||||.|||||:|||||||||||||||||

M.mulatta 101 PLLFPCYQWLEGAGSLVLQEGTAKVSRADHHPLLQQQRQEELQARQEMYQ 150

H.sapiens 151 WKAYNPGWPHCLDEKTVEDLELNIKYSTAKNANFYLQAGSAFAEMKIKGL 200

|||||||||||||::||:||:|||||||||||||||||||||||||||||

M.mulatta 151 WKAYNPGWPHCLDKRTVKDLDLNIKYSTAKNANFYLQAGSAFAEMKIKGL 200

H.sapiens 201 LDRKGLWRSLNEMKRIFNFRRTPAAEHAFEHWQEDAFFASQFLNGLNPVL 250

||||||||||||||||||||:|||||||||||||||||||||||||||||

M.mulatta 201 LDRKGLWRSLNEMKRIFNFRKTPAAEHAFEHWQEDAFFASQFLNGLNPVL 250

H.sapiens 251 IRRCHYLPKNFPVTDAMVASVLGPGTSLQAELEKGSLFLVDHGILSGIQT 300

||||||||||||||||||||||||||||||||||||||||||||||||.|

M.mulatta 251 IRRCHYLPKNFPVTDAMVASVLGPGTSLQAELEKGSLFLVDHGILSGIHT 300

H.sapiens 301 NVINGKPQFSAAPMTLLYQSPGCGPLLPLAIQLSQTPGPNSPIFLPTDDK 350

|||||||||||||||||||||||||||||||||||:|||:||||||||||

M.mulatta 301 NVINGKPQFSAAPMTLLYQSPGCGPLLPLAIQLSQSPGPHSPIFLPTDDK 350

H.sapiens 351 WDWLLAKTWVRNAEFSFHEALTHLLHSHLLPEVFTLATLRQLPHCHPLFK 400

||||||||||||||||||||||||||||||||||||||||||||||||||

M.mulatta 351 WDWLLAKTWVRNAEFSFHEALTHLLHSHLLPEVFTLATLRQLPHCHPLFK 400

H.sapiens 401 LLIPHTRYTLHINTLARELLIVPGQVVDRSTGIGIEGFSELIQRNMKQLN 450

|||||||||||||.||||||||||||||||||||||||||||||||||||

M.mulatta 401 LLIPHTRYTLHINMLARELLIVPGQVVDRSTGIGIEGFSELIQRNMKQLN 450

H.sapiens 451 YSLLCLPEDIRTRGVEDIPGYYYRDDGMQIWGAVERFVSEIIGIYYPSDE 500

|||||||||||||||||||||||||||||||||||||||||||||||:||

M.mulatta 451 YSLLCLPEDIRTRGVEDIPGYYYRDDGMQIWGAVERFVSEIIGIYYPNDE 500

H.sapiens 501 SVQDDRELQAWVREIFSKGFLNQESSGIPSSLETREALVQYVTMVIFTCS 550

|||||||||||||||||||||||.|||||||||||||||||||||||.||

M.mulatta 501 SVQDDRELQAWVREIFSKGFLNQGSSGIPSSLETREALVQYVTMVIFNCS 550

H.sapiens 551 AKHAAVSAGQFDSCAWMPNLPPSMQLPPPTSKGLATCEGFIATLPPVNAT 600

||||||||||||:|||||||||||||||||||||||.|||||||||||||

M.mulatta 551 AKHAAVSAGQFDACAWMPNLPPSMQLPPPTSKGLATREGFIATLPPVNAT 600

H.sapiens 601 CDVILALWLLSKEPGDQRPLGTYPDEHFTEEAPRRSIATFQSRLAQISRG 650

||||||||||||||||:|||||||||||||||||||||.|||.|||||..

M.mulatta 601 CDVILALWLLSKEPGDRRPLGTYPDEHFTEEAPRRSIAAFQSHLAQISGA 650

H.sapiens 651 IQERNQGLVLPYTYLDPPLIENSVSI 676

|||||:||||||||||||||||||||

M.mulatta 651 IQERNRGLVLPYTYLDPPLIENSVSI 676

Sooty mangabey (*Cercocebus atys*)

H.sapiens 1 MAEFRVRVSTGEAFGAGTWDKVSVSIVGTRGESPPLPLDNLGKEFTAGAE 50

||:||||||||:||||||||||||||||||.|||||||||||||||||||

C.atys 1 MADFRVRVSTGKAFGAGTWDKVSVSIVGTREESPPLPLDNLGKEFTAGAE 50

H.sapiens 51 EDFQVTLPEDVGRVLLLRVHKAPPVLPLLGPLAPDAWFCRWFQLTPPRGG 100

||||||||:|||:|||||||||||.|||||||||||||||||||||||||

C.atys 51 EDFQVTLPKDVGQVLLLRVHKAPPALPLLGPLAPDAWFCRWFQLTPPRGG 100

H.sapiens 101 HLLFPCYQWLEGAGTLVLQEGTAKVSWADHHPVLQQQRQEELQARQEMYQ 150

.|||||||||||||:|||||||||||.|:|||:|||||||||||||||||

C.atys 101 PLLFPCYQWLEGAGSLVLQEGTAKVSRANHHPLLQQQRQEELQARQEMYQ 150

H.sapiens 151 WKAYNPGWPHCLDEKTVEDLELNIKYSTAKNANFYLQAGSAFAEMKIKGL 200

|||||||||||||::||:||:|||||||||||||||||||||||||||||

C.atys 151 WKAYNPGWPHCLDKRTVKDLDLNIKYSTAKNANFYLQAGSAFAEMKIKGL 200

H.sapiens 201 LDRKGLWRSLNEMKRIFNFRRTPAAEHAFEHWQEDAFFASQFLNGLNPVL 250

||||||||||||||||||||:|||||||||||||||||||||||||||||

C.atys 201 LDRKGLWRSLNEMKRIFNFRKTPAAEHAFEHWQEDAFFASQFLNGLNPVL 250

H.sapiens 251 IRRCHYLPKNFPVTDAMVASVLGPGTSLQAELEKGSLFLVDHGILSGIQT 300

||.|||||||||||||||||||||||||||||||||||||||||||||.|

C.atys 251 IRHCHYLPKNFPVTDAMVASVLGPGTSLQAELEKGSLFLVDHGILSGIHT 300

H.sapiens 301 NVINGKPQFSAAPMTLLYQSPGCGPLLPLAIQLSQTPGPNSPIFLPTDDK 350

|||||||||||||||||||||||||||||||||||:|||:||||||||||

C.atys 301 NVINGKPQFSAAPMTLLYQSPGCGPLLPLAIQLSQSPGPHSPIFLPTDDK 350

H.sapiens 351 WDWLLAKTWVRNAEFSFHEALTHLLHSHLLPEVFTLATLRQLPHCHPLFK 400

||||||||||||||||||||||||||||||||||||||||||||||||||

C.atys 351 WDWLLAKTWVRNAEFSFHEALTHLLHSHLLPEVFTLATLRQLPHCHPLFK 400

H.sapiens 401 LLIPHTRYTLHINTLARELLIVPGQVVDRSTGIGIEGFSELIQRNMKQLN 450

|||||||||||||.||||||||||||||||||||||||||||||||||||

C.atys 401 LLIPHTRYTLHINMLARELLIVPGQVVDRSTGIGIEGFSELIQRNMKQLN 450

H.sapiens 451 YSLLCLPEDIRTRGVEDIPGYYYRDDGMQIWGAVERFVSEIIGIYYPSDE 500

||||||||||||||||||||||||||||||||||||||||||||||||||

C.atys 451 YSLLCLPEDIRTRGVEDIPGYYYRDDGMQIWGAVERFVSEIIGIYYPSDE 500

H.sapiens 501 SVQDDRELQAWVREIFSKGFLNQESSGIPSSLETREALVQYVTMVIFTCS 550

|||||||||||||||||||||||:|||||||||||||||||||||||.||

C.atys 501 SVQDDRELQAWVREIFSKGFLNQKSSGIPSSLETREALVQYVTMVIFNCS 550

H.sapiens 551 AKHAAVSAGQFDSCAWMPNLPPSMQLPPPTSKGLATCEGFIATLPPVNAT 600

||||||||||||:|||||||||||||||||||||||.|||||||||||||

C.atys 551 AKHAAVSAGQFDACAWMPNLPPSMQLPPPTSKGLATREGFIATLPPVNAT 600

H.sapiens 601 CDVILALWLLSKEPGDQRPLGTYPDEHFTEEAPRRSIATFQSRLAQISRG 650

||||||||||||||||:|||||||||||||||||||||||||.||||||.

C.atys 601 CDVILALWLLSKEPGDRRPLGTYPDEHFTEEAPRRSIATFQSHLAQISRA 650

H.sapiens 651 IQERNQGLVLPYTYLDPPLIENSVSI 676

|||||:||||||||||||||||||||

C.atys 651 IQERNRGLVLPYTYLDPPLIENSVSI 676

Southern pig-tailed macaque (*Macaca nemestrina*)

H.sapiens 1 MAEFRVRVSTGEAFGAGTWDKVSVSIVGTRGESPPLPLDNLGKEFTAGAE 50

||:||||||||:|||||||||||||||||:.|||||||||||||||||||

M.nemestrina 1 MADFRVRVSTGKAFGAGTWDKVSVSIVGTQEESPPLPLDNLGKEFTAGAE 50

H.sapiens 51 EDFQVTLPEDVGRVLLLRVHKAPPVLPLLGPLAPDAWFCRWFQLTPPRGG 100

||||||||:|||:|||||||||||.|||||||||||||||||||||||||

M.nemestrina 51 EDFQVTLPKDVGQVLLLRVHKAPPALPLLGPLAPDAWFCRWFQLTPPRGG 100

H.sapiens 101 HLLFPCYQWLEGAGTLVLQEGTAKVSWADHHPVLQQQRQEELQARQEMYQ 150

.|||||||||||||:|||||||||||.|||||:|||||||||||||||||

M.nemestrina 101 PLLFPCYQWLEGAGSLVLQEGTAKVSRADHHPLLQQQRQEELQARQEMYQ 150

H.sapiens 151 WKAYNPGWPHCLDEKTVEDLELNIKYSTAKNANFYLQAGSAFAEMKIKGL 200

|||||||||||||::||:||:|||||||||||||||||||||||||||||

M.nemestrina 151 WKAYNPGWPHCLDKRTVKDLDLNIKYSTAKNANFYLQAGSAFAEMKIKGL 200

H.sapiens 201 LDRKGLWRSLNEMKRIFNFRRTPAAEHAFEHWQEDAFFASQFLNGLNPVL 250

||||||||||||||||||||:|||||||||||||||||||||||||||||

M.nemestrina 201 LDRKGLWRSLNEMKRIFNFRKTPAAEHAFEHWQEDAFFASQFLNGLNPVL 250

H.sapiens 251 IRRCHYLPKNFPVTDAMVASVLGPGTSLQAELEKGSLFLVDHGILSGIQT 300

||||||||||||||||||||||||||||||||||||||||||||||||.|

M.nemestrina 251 IRRCHYLPKNFPVTDAMVASVLGPGTSLQAELEKGSLFLVDHGILSGIHT 300

H.sapiens 301 NVINGKPQFSAAPMTLLYQSPGCGPLLPLAIQLSQTPGPNSPIFLPTDDK 350

|||||||||||||||||||||||||||||||||||:|||:|||||||||:

M.nemestrina 301 NVINGKPQFSAAPMTLLYQSPGCGPLLPLAIQLSQSPGPHSPIFLPTDDE 350

H.sapiens 351 WDWLLAKTWVRNAEFSFHEALTHLLHSHLLPEVFTLATLRQLPHCHPLFK 400

||||||||||||||||||||||||||||||||.|||||||||||||||||

M.nemestrina 351 WDWLLAKTWVRNAEFSFHEALTHLLHSHLLPEAFTLATLRQLPHCHPLFK 400

H.sapiens 401 LLIPHTRYTLHINTLARELLIVPGQVVDRSTGIGIEGFSELIQRNMKQLN 450

|||||||||||||.||||||||||||||||||||||||||||||||||||

M.nemestrina 401 LLIPHTRYTLHINMLARELLIVPGQVVDRSTGIGIEGFSELIQRNMKQLN 450

H.sapiens 451 YSLLCLPEDIRTRGVEDIPGYYYRDDGMQIWGAVERFVSEIIGIYYPSDE 500

|||||||||||||||||||||||||||||||||||||||||||||||:||

M.nemestrina 451 YSLLCLPEDIRTRGVEDIPGYYYRDDGMQIWGAVERFVSEIIGIYYPNDE 500

H.sapiens 501 SVQDDRELQAWVREIFSKGFLNQESSGIPSSLETREALVQYVTMVIFTCS 550

|||||||||||||||||||||||.|||||||||||||||||||||||.||

M.nemestrina 501 SVQDDRELQAWVREIFSKGFLNQGSSGIPSSLETREALVQYVTMVIFNCS 550

H.sapiens 551 AKHAAVSAGQFDSCAWMPNLPPSMQLPPPTSKGLATCEGFIATLPPVNAT 600

||||||||||||:|||||||||||||||||||||||.|||||||||||||

M.nemestrina 551 AKHAAVSAGQFDACAWMPNLPPSMQLPPPTSKGLATREGFIATLPPVNAT 600

H.sapiens 601 CDVILALWLLSKEPGDQRPLGTYPDEHFTEEAPRRSIATFQSRLAQISRG 650

||||||||||||||||:..:| ..:||.: .|

M.nemestrina 601 CDVILALWLLSKEPGDRVSVG------------------LGARLGR--TG 630

H.sapiens 651 IQERNQGLVLPYTYLDPPLIENSVSI------------------------ 676

::| |::........|..:.:..:

M.nemestrina 631 VRE---GVMFTLLAQTQPCHQGAGPLAPLTLQWDTRALDISQRWSNGKMR 677

Crab-eating macaque (*Macaca fascicularis*)

H.sapiens 1 MAEFRVRVSTGEAFGAGTWDKVSVSIVGTRGESPPLPLDNLGKEFTAGAE 50

||:||||||||:|||||||||||||||||:.|||||||||||||||||||

M.fasciculari 1 MADFRVRVSTGKAFGAGTWDKVSVSIVGTQEESPPLPLDNLGKEFTAGAE 50

H.sapiens 51 EDFQVTLPEDVGRVLLLRVHKAPPVLPLLGPLAPDAWFCRWFQLTPPRGG 100

||||||||:|||:|||||||||||.|||||||||||||||||||||||||

M.fascicularis 51 EDFQVTLPKDVGQVLLLRVHKAPPALPLLGPLAPDAWFCRWFQLTPPRGG 100

H.sapiens 101 HLLFPCYQWLEGAGTLVLQEGTAKVSWADHHPVLQQQRQEELQARQEMYQ 150

.|||||||||||||:|||||||||||.|||||:|||||||||||||||||

M.fascicularis 101 PLLFPCYQWLEGAGSLVLQEGTAKVSRADHHPLLQQQRQEELQARQEMYQ 150

H.sapiens 151 WKAYNPGWPHCLDEKTVEDLELNIKYSTAKNANFYLQAGSAFAEMKIKGL 200

|||||||||||||::||:||:|||||||||||||||||||||||||||||

M.fascicularis 151 WKAYNPGWPHCLDKRTVKDLDLNIKYSTAKNANFYLQAGSAFAEMKIKGL 200

H.sapiens 201 LDRKGLWRSLNEMKRIFNFRRTPAAEHAFEHWQEDAFFASQFLNGLNPVL 250

||||||||||||||||||||:|||||||||||||||||||||||||||||

M.fascicularis 201 LDRKGLWRSLNEMKRIFNFRKTPAAEHAFEHWQEDAFFASQFLNGLNPVL 250

H.sapiens 251 IRRCHYLPKNFPVTDAMVASVLGPGTSLQAELEKGSLFLVDHGILSGIQT 300

||||||||||||||||||||||||||||||||||||||||||||||||.|

M.fascicularis 251 IRRCHYLPKNFPVTDAMVASVLGPGTSLQAELEKGSLFLVDHGILSGIHT 300

H.sapiens 301 NVINGKPQFSAAPMTLLYQSPGCGPLLPLAIQLSQTPGPNSPIFLPTDDK 350

|||||||||||||||||||||||||||||||||||:|||:||||||||||

M.fascicularis 301 NVINGKPQFSAAPMTLLYQSPGCGPLLPLAIQLSQSPGPHSPIFLPTDDK 350

H.sapiens 351 WDWLLAKTWVRNAEFSFHEALTHLLHSHLLPEVFTLATLRQLPHCHPLFK 400

||||||||||||||||||||||||||||||||||||||||||||||||||

M.fascicularis 351 WDWLLAKTWVRNAEFSFHEALTHLLHSHLLPEVFTLATLRQLPHCHPLFK 400

H.sapiens 401 LLIPHTRYTLHINTLARELLIVPGQVVDRSTGIGIEGFSELIQRNMKQLN 450

|||||||||||||.||||||||||||||||||||||||||||||||||||

M.fascicularis 401 LLIPHTRYTLHINMLARELLIVPGQVVDRSTGIGIEGFSELIQRNMKQLN 450

H.sapiens 451 YSLLCLPEDIRTRGVEDIPGYYYRDDGMQIWGAVERFVSEIIGIYYPSDE 500

|||||||||||||||||||||||||||||||||||||||||||||||:||

M.fascicularis 451 YSLLCLPEDIRTRGVEDIPGYYYRDDGMQIWGAVERFVSEIIGIYYPNDE 500

H.sapiens 501 SVQDDRELQAWVREIFSKGFLNQESSGIPSSLETREALVQYVTMVIFTCS 550

|||||||||||||||||||||||.|||||||||||||||||||||||.||

M.fascicularis 501 SVQDDRELQAWVREIFSKGFLNQGSSGIPSSLETREALVQYVTMVIFNCS 550

H.sapiens 551 AKHAAVSAGQFDSCAWMPNLPPSMQLPPPTSKGLATCEGFIATLPPVNAT 600

||||||||||||:|||||||||||||||||||||||.|||||||||||||

M.fascicularis 551 AKHAAVSAGQFDACAWMPNLPPSMQLPPPTSKGLATREGFIATLPPVNAT 600

H.sapiens 601 CDVILALWLLSKEPGDQRPLGTYPDEHFTEEAPRRSIATFQSRLAQISRG 650

||||||||||||||||:|||||||||||||||||||||.|||.|||||..

M.fascicularis 601 CDVILALWLLSKEPGDRRPLGTYPDEHFTEEAPRRSIAAFQSHLAQISGA 650

H.sapiens 651 IQERNQGLVLPYTYLDPPLIENSVSI 676

|||||:||||||||||||||||||||

M.fascicularis 651 IQERNRGLVLPYTYLDPPLIENSVSI 676

Gelada (*Theropithecus gelada*)

H.sapiens 1 MAEFRVRVSTGEAFGAGTWDKVSVSIVGTRGESPPLPLDNLGKEFTAGAE 50

||:||||||||:||||||||||||||||||.|||||||||||||||||||

T.gelada 1 MADFRVRVSTGKAFGAGTWDKVSVSIVGTREESPPLPLDNLGKEFTAGAE 50

H.sapiens 51 EDFQVTLPEDVGRVLLLRVHKAPPVLPLLGPLAPDAWFCRWFQLTPPRGG 100

||||||||:|||:|||||||||||.|||||||||||||||||||||||||

T.gelada 51 EDFQVTLPKDVGQVLLLRVHKAPPALPLLGPLAPDAWFCRWFQLTPPRGG 100

H.sapiens 101 HLLFPCYQWLEGAGTLVLQEGTAKVSWADHHPVLQQQRQEELQARQEMYQ 150

..||||||||||||:|||||||||||.|||||:|||||||||||||||||

T.gelada 101 PHLFPCYQWLEGAGSLVLQEGTAKVSRADHHPLLQQQRQEELQARQEMYQ 150

H.sapiens 151 WKAYNPGWPHCLDEKTVEDLELNIKYSTAKNANFYLQAGSAFAEMKIKGL 200

|||||||||||||::||:||:|||||||||||||||||||||||||||||

T.gelada 151 WKAYNPGWPHCLDKRTVKDLDLNIKYSTAKNANFYLQAGSAFAEMKIKGL 200

H.sapiens 201 LDRKGLWRSLNEMKRIFNFRRTPAAEHAFEHWQEDAFFASQFLNGLNPVL 250

||||||||||||||||||||:|||||||||||||||||||||||||||||

T.gelada 201 LDRKGLWRSLNEMKRIFNFRKTPAAEHAFEHWQEDAFFASQFLNGLNPVL 250

H.sapiens 251 IRRCHYLPKNFPVTDAMVASVLGPGTSLQAELEKGSLFLVDHGILSGIQT 300

||||||||||||||||||||||||||||||||||||||||||||||||.|

T.gelada 251 IRRCHYLPKNFPVTDAMVASVLGPGTSLQAELEKGSLFLVDHGILSGIHT 300

H.sapiens 301 NVINGKPQFSAAPMTLLYQSPGCGPLLPLAIQLSQTPGPNSPIFLPTDDK 350

|||||||||||||||||||||||||||||||||||:|||:||||||||||

T.gelada 301 NVINGKPQFSAAPMTLLYQSPGCGPLLPLAIQLSQSPGPHSPIFLPTDDK 350

H.sapiens 351 WDWLLAKTWVRNAEFSFHEALTHLLHSHLLPEVFTLATLRQLPHCHPLFK 400

||||||||||||||||||||||||||||||||||||||||||||||||||

T.gelada 351 WDWLLAKTWVRNAEFSFHEALTHLLHSHLLPEVFTLATLRQLPHCHPLFK 400

H.sapiens 401 LLIPHTRYTLHINTLARELLIVPGQVVDRSTGIGIEGFSELIQRNMKQLN 450

|||||||||||||.||||||||||||||||||||||||||||||||||||

T.gelada 401 LLIPHTRYTLHINMLARELLIVPGQVVDRSTGIGIEGFSELIQRNMKQLN 450

H.sapiens 451 YSLLCLPEDIRTRGVEDIPGYYYRDDGMQIWGAVERFVSEIIGIYYPSDE 500

||||||||||:|||||||||||||||||||||||||||||||||||||||

T.gelada 451 YSLLCLPEDIQTRGVEDIPGYYYRDDGMQIWGAVERFVSEIIGIYYPSDE 500

H.sapiens 501 SVQDDRELQAWVREIFSKGFLNQESSGIPSSLETREALVQYVTMVIFTCS 550

|||||||||||||||||||||||||||||||||||||||||||||||.||

T.gelada 501 SVQDDRELQAWVREIFSKGFLNQESSGIPSSLETREALVQYVTMVIFNCS 550

H.sapiens 551 AKHAAVSAGQFDSCAWMPNLPPSMQLPPPTSKGLATCEGFIATLPPVNAT 600

||||||||||||:|||||||||||||||||||||||.|||||||||||||

T.gelada 551 AKHAAVSAGQFDACAWMPNLPPSMQLPPPTSKGLATREGFIATLPPVNAT 600

H.sapiens 601 CDVILALWLLSKEPGDQRPLGTYPDEHFTEEAPRRSIATFQSRLAQISRG 650

||||||||||||||||:|||||||||||||||||||||||||.||||||.

T.gelada 601 CDVILALWLLSKEPGDRRPLGTYPDEHFTEEAPRRSIATFQSHLAQISRA 650

H.sapiens 651 IQERNQGLVLPYTYLDPPLIENSVSI 676

|||||:||||||||||||||||||||

T.gelada 651 IQERNRGLVLPYTYLDPPLIENSVSI 676

Drill (*Mandrillus leucophaeus*)

H.sapiens 1 MAEFRVRVSTGEAFGAGTWDKVSVSIVGTRGESPPLPLDNLGKEFTAGAE 50

||:||||||||:||||||||||||||||||.|||||||||||||||||||

M.leucophaeus 1 MADFRVRVSTGKAFGAGTWDKVSVSIVGTREESPPLPLDNLGKEFTAGAE 50

H.sapiens 51 EDFQVTLPEDVGRVLLLRVHKAPPVLPLLGPLAPDAWFCRWFQLTPPRGG 100

||||||||:|||:|||||||||||.|||||||||||||||||||||||||

M.leucophaeus 51 EDFQVTLPKDVGQVLLLRVHKAPPALPLLGPLAPDAWFCRWFQLTPPRGG 100

H.sapiens 101 HLLFPCYQWLEGAGTLVLQEGTAKVSWADHHPVLQQQRQEELQARQEMYQ 150

.|||||||||||||:|||||||||||.|:|||:|||||||||||||||||

M.leucophaeus 101 PLLFPCYQWLEGAGSLVLQEGTAKVSRANHHPLLQQQRQEELQARQEMYQ 150

H.sapiens 151 WKAYNPGWPHCLDEKTVEDLELNIKYSTAKNANFYLQAGSAFAEMKIKGL 200

|||||||||||||::||:||:|||||||||||||||||||||||||||||

M.leucophaeus 151 WKAYNPGWPHCLDKRTVKDLDLNIKYSTAKNANFYLQAGSAFAEMKIKGL 200

H.sapiens 201 LDRKGLWRSLNEMKRIFNFRRTPAAEHAFEHWQEDAFFASQFLNGLNPVL 250

||||||||||||||||||||:|||||||||||||||||||||||||||||

M.leucophaeus 201 LDRKGLWRSLNEMKRIFNFRKTPAAEHAFEHWQEDAFFASQFLNGLNPVL 250

H.sapiens 251 IRRCHYLPKNFPVTDAMVASVLGPGTSLQAELEKGSLFLVDHGILSGIQT 300

||||||||||||||||||||||||||||||||||||||||||||||||.|

M.leucophaeus 251 IRRCHYLPKNFPVTDAMVASVLGPGTSLQAELEKGSLFLVDHGILSGIHT 300

H.sapiens 301 NVINGKPQFSAAPMTLLYQSPGCGPLLPLAIQLSQTPGPNSPIFLPTDDK 350

|||||||||||||:|||||||||||||||||||||:|||:||||||||||

M.leucophaeus 301 NVINGKPQFSAAPVTLLYQSPGCGPLLPLAIQLSQSPGPHSPIFLPTDDK 350

H.sapiens 351 WDWLLAKTWVRNAEFSFHEALTHLLHSHLLPEVFTLATLRQLPHCHPLFK 400

||||||||||||||||||||||||||||||||||||||||||||||||||

M.leucophaeus 351 WDWLLAKTWVRNAEFSFHEALTHLLHSHLLPEVFTLATLRQLPHCHPLFK 400

H.sapiens 401 LLIPHTRYTLHINTLARELLIVPGQVVDRSTGIGIEGFSELIQRNMKQLN 450

|||||||||||||.||||||||||||||||||||||||||||||||||||

M.leucophaeus 401 LLIPHTRYTLHINMLARELLIVPGQVVDRSTGIGIEGFSELIQRNMKQLN 450

H.sapiens 451 YSLLCLPEDIRTRGVEDIPGYYYRDDGMQIWGAVERFVSEIIGIYYPSDE 500

||||||||||||||||||||||||||||||||||||||||||||||||||

M.leucophaeus 451 YSLLCLPEDIRTRGVEDIPGYYYRDDGMQIWGAVERFVSEIIGIYYPSDE 500

H.sapiens 501 SVQDDRELQAWVREIFSKGFLNQESSGIPSSLETREALVQYVTMVIFTCS 550

|||||||||||||||||||||||||||||||||||||||||||||||.||

M.leucophaeus 501 SVQDDRELQAWVREIFSKGFLNQESSGIPSSLETREALVQYVTMVIFNCS 550

H.sapiens 551 AKHAAVSAGQFDSCAWMPNLPPSMQLPPPTSKGLATCEGFIATLPPVNAT 600

||||||||||||:|||||||||||||||||||||||.|||||||||||||

M.leucophaeus 551 AKHAAVSAGQFDACAWMPNLPPSMQLPPPTSKGLATREGFIATLPPVNAT 600

H.sapiens 601 CDVILALWLLSKEPGDQRPLGTYPDEHFTEEAPRRSIATFQSRLAQISRG 650

||||||||||||||||:|||||||||||||||||||||||||.||||||.

M.leucophaeus 601 CDVILALWLLSKEPGDRRPLGTYPDEHFTEEAPRRSIATFQSHLAQISRA 650

H.sapiens 651 IQERNQGLVLPYTYLDPPLIENSVSI 676

|||||:||||||||||||||||||||

M.leucophaeus 651 IQERNRGLVLPYTYLDPPLIENSVSI 676

Philippine tarsier (*Carlito syrichta*)

H.sapiens 1 MAEFRVRVSTGEAFGAGTWDKVSVSIVGTRGESPPLPLDNLGKEFTAGAE 50

|.:|||||||||||||||||||.||||||||||||||||:||||||||||

C.syrichta 1 MTKFRVRVSTGEAFGAGTWDKVFVSIVGTRGESPPLPLDHLGKEFTAGAE 50

H.sapiens 51 EDFQVTLPEDVGRVLLLRVHKAPPVLP-LLGPLAPDAWFCRWFQLTPPRG 99

|||:||||:|||:||||||||.||.|| |||.|.||||||||||||||.|

C.syrichta 51 EDFEVTLPQDVGQVLLLRVHKVPPALPRLLGTLDPDAWFCRWFQLTPPGG 100

H.sapiens 100 GHLLFPCYQWLEGAGTLVLQEGTAKVSWADHHPVLQQQRQEELQARQEMY 149

..|.|||||||||...|||:||.||:|.||.||.||:|||||||||||||

C.syrichta 101 ASLRFPCYQWLEGTRNLVLREGAAKLSRADQHPKLQKQRQEELQARQEMY 150

H.sapiens 150 QWKAYNPGWPHCLDEKTVEDLELNIKYSTAKNANFYLQAGSAFAEMKIKG 199

.||.|.|||||||||.||:||:||.|||||||.:|||:.|||..|::|||

C.syrichta 151 HWKTYIPGWPHCLDEATVKDLDLNTKYSTAKNIHFYLRGGSALTELRIKG 200

H.sapiens 200 LLDRKGLWRSLNEMKRIFNFRRTPAAEHAFEHWQEDAFFASQFLNGLNPV 249

|||||||||||.||:||.|||:|.|||:||||||||.|||||||||.|||

C.syrichta 201 LLDRKGLWRSLTEMRRILNFRKTSAAEYAFEHWQEDTFFASQFLNGFNPV 250

H.sapiens 250 LIRRCHYLPKNFPVTDAMVASVLGPGTSLQAELEKGSLFLVDHGILSGIQ 299

|||||..|||||||||||||.|||||||||.|||:||||||||||||||:

C.syrichta 251 LIRRCRSLPKNFPVTDAMVAPVLGPGTSLQTELERGSLFLVDHGILSGIR 300

H.sapiens 300 TNVINGKPQFSAAPMTLLYQSPGCGPLLPLAIQLSQTPGPNSPIFLPTDD 349

||:|||:|||||||||||||.||||||||||||||||||||||||||:||

C.syrichta 301 TNIINGRPQFSAAPMTLLYQRPGCGPLLPLAIQLSQTPGPNSPIFLPSDD 350

H.sapiens 350 KWDWLLAKTWVRNAEFSFHEALTHLLHSHLLPEVFTLATLRQLPHCHPLF 399

|||||||||||||||||||||||||||:|||||||.:|||||||||||||

C.syrichta 351 KWDWLLAKTWVRNAEFSFHEALTHLLHAHLLPEVFAMATLRQLPHCHPLF 400

H.sapiens 400 KLLIPHTRYTLHINTLARELLIVPGQVVDRSTGIGIEGFSELIQRNMKQL 449

|||||||:||||||||||||||.||||||||||:||.||||||||||:||

C.syrichta 401 KLLIPHTKYTLHINTLARELLIAPGQVVDRSTGLGIGGFSELIQRNMEQL 450

H.sapiens 450 NYSLLCLPEDIRTRGVEDIPGYYYRDDGMQIWGAVERFVSEIIGIYYPSD 499

|||:|||||||||||||||||||:||||:|:|.||||||||||.||||||

C.syrichta 451 NYSVLCLPEDIRTRGVEDIPGYYFRDDGLQVWDAVERFVSEIISIYYPSD 500

H.sapiens 500 ESVQDDRELQAWVREIFSKGFLNQESSGIPSSLETREALVQYVTMVIFTC 549

.|||||.|||||||||||:|||.:||||:|||||||||||:|||||||||

C.syrichta 501 VSVQDDAELQAWVREIFSEGFLRKESSGVPSSLETREALVRYVTMVIFTC 550

H.sapiens 550 SAKHAAVSAGQFDSCAWMPNLPPSMQLPPPTSKGLATCEGFIATLPPVNA 599

||.|:|:|:||||:|||||||||:||||||||||.|..||||||||||||

C.syrichta 551 SAMHSAISSGQFDACAWMPNLPPTMQLPPPTSKGRARPEGFIATLPPVNA 600

H.sapiens 600 TCDVILALWLLSKEPGDQRPLGTYPDEHFTEEAPRRSIATFQSRLAQISR 649

||||.:||||||||||:||||||||||||||||||:||..||.|||||||

C.syrichta 601 TCDVAIALWLLSKEPGEQRPLGTYPDEHFTEEAPRQSITAFQRRLAQISR 650

H.sapiens 650 GIQERNQGLVLPYTYLDPPLIENSVSI 676

.|:||||||.|||||||||||||||:|

C.syrichta 651 DIRERNQGLALPYTYLDPPLIENSVAI 677

Nancy Ma's night monkey (*Aotus nancymaae*)

H.sapiens 1 MAEFRVRVSTGEAFGAGTWDKVSVSIVGTRGESPPLPLDNLGKEFTAGAE 50

|||||||||||||||||||:|||||||||:||||.||||:|||||.||||

A.nancymaae 1 MAEFRVRVSTGEAFGAGTWNKVSVSIVGTQGESPRLPLDHLGKEFNAGAE 50

H.sapiens 51 EDFQVTLPEDVGRVLLLRVHKAPPVLP-LLGPLAPDAWFCRWFQLTPPRG 99

|||||.||||||.||||||||.||.:. ||.|||.|||||||||||||:|

A.nancymaae 51 EDFQVMLPEDVGPVLLLRVHKMPPAMSCLLRPLAQDAWFCRWFQLTPPQG 100

H.sapiens 100 GHLLFPCYQWLEGAGTLVLQEGTAKVSWADHHPVLQQQRQEELQARQEMY 149

...||||||||||||:|||:|||||||||||||:||||||:|||||||||

A.nancymaae 101 SPFLFPCYQWLEGAGSLVLREGTAKVSWADHHPLLQQQRQQELQARQEMY 150

H.sapiens 150 QWKAYNPGWPHCLDEKTVEDLELNIKYSTAKNANFYLQAGSAFAEMKIKG 199

||||||||||||||||:|:||::||||||||||||||::||||.||||||

A.nancymaae 151 QWKAYNPGWPHCLDEKSVKDLDINIKYSTAKNANFYLRSGSAFVEMKIKG 200

H.sapiens 200 LLDRKGLWRSLNEMKRIFNFRRTPAAEHAFEHWQEDAFFASQFLNGLNPV 249

||||||.||||||||||||||:|||||:.|||||||||||||||||||||

A.nancymaae 201 LLDRKGPWRSLNEMKRIFNFRKTPAAEYTFEHWQEDAFFASQFLNGLNPV 250

H.sapiens 250 LIRRCHYLPKNFPVTDAMVASVLGPGTSLQAELEKGSLFLVDHGILSGIQ 299

||||||.||||||||||||||||||||||||||||||||||||||||.|.

A.nancymaae 251 LIRRCHCLPKNFPVTDAMVASVLGPGTSLQAELEKGSLFLVDHGILSNIH 300

H.sapiens 300 TNVINGKPQFSAAPMTLLYQSPGCGPLLPLAIQLSQTPGPNSPIFLPTDD 349

||||||:|||||||||||||.||.|||||||||||||||||||||||:||

A.nancymaae 301 TNVINGRPQFSAAPMTLLYQRPGGGPLLPLAIQLSQTPGPNSPIFLPSDD 350

H.sapiens 350 KWDWLLAKTWVRNAEFSFHEALTHLLHSHLLPEVFTLATLRQLPHCHPLF 399

||||||||||||||||||||||||||||||||||||||||||||||||||

A.nancymaae 351 KWDWLLAKTWVRNAEFSFHEALTHLLHSHLLPEVFTLATLRQLPHCHPLF 400

H.sapiens 400 KLLIPHTRYTLHINTLARELLIVPGQVVDRSTGIGIEGFSELIQRNMKQL 449

|||||||||||||||||||||||||||||:|||:||.||||||||:||||

A.nancymaae 401 KLLIPHTRYTLHINTLARELLIVPGQVVDKSTGLGIGGFSELIQRSMKQL 450

H.sapiens 450 NYSLLCLPEDIRTRGVEDIPGYYYRDDGMQIWGAVERFVSEIIGIYYPSD 499

|||.||||||||||||||||||||||||:|||||||||||||||||||||

A.nancymaae 451 NYSALCLPEDIRTRGVEDIPGYYYRDDGLQIWGAVERFVSEIIGIYYPSD 500

H.sapiens 500 ESVQDDRELQAWVREIFSKGFLNQESSGIPSSLETREALVQYVTMVIFTC 549

|||||||||||||.|||||||||:||||||||||||||||:|||||||||

A.nancymaae 501 ESVQDDRELQAWVGEIFSKGFLNRESSGIPSSLETREALVRYVTMVIFTC 550

H.sapiens 550 SAKHAAVSAGQFDSCAWMPNLPPSMQLPPPTSKGLATCEGFIATLPPVNA 599

||:|:||||||||||||||||||||||||||||..||.|.||.|||||||

A.nancymaae 551 SARHSAVSAGQFDSCAWMPNLPPSMQLPPPTSKSQATPESFINTLPPVNA 600

H.sapiens 600 TCDVILALWLLSKEPGDQRPLGTYPDEHFTEEAPRRSIATFQSRLAQISR 649

||||||.||||||||||.|||||||||||.||||||||||||:|||||||

A.nancymaae 601 TCDVILTLWLLSKEPGDPRPLGTYPDEHFIEEAPRRSIATFQNRLAQISR 650

H.sapiens 650 GIQERNQGLVLPYTYLDPPLIENSVSI 676

.|||||:||.|||||||||:|||||||

A.nancymaae 651 DIQERNRGLALPYTYLDPPIIENSVSI 677

Angola colobus (*Colobus angolensis palliatus*)

H.sapiens 1 MAEFRVRVSTGEAFGAGTWDKVSVSIVGTRGESPPLPLDNLGKEFTAGAE 50

||:||||||||:||||||||||||||||||.||||:||||||||||||||

C.angolensis 1 MADFRVRVSTGKAFGAGTWDKVSVSIVGTREESPPMPLDNLGKEFTAGAE 50

H.sapiens 51 EDFQVTLPEDVGRVLLLRVHKAPPVLPLLGPLAPDAWFCRWFQLTPPRGG 100

||||||||:|||:|||||||||||.||||..||||||||||||||||:|.

C.angolensis 51 EDFQVTLPKDVGQVLLLRVHKAPPALPLLRSLAPDAWFCRWFQLTPPQGS 100

H.sapiens 101 HLLFPCYQWLEGAGTLVLQEGTAKVSWADHHPVLQQQRQEELQARQEMYQ 150

.|||||||||||||:|||||||||||.|||||:|||||||||||||:|||

C.angolensis 101 PLLFPCYQWLEGAGSLVLQEGTAKVSRADHHPLLQQQRQEELQARQKMYQ 150

H.sapiens 151 WKAYNPGWPHCLDEKTVEDLELNIKYSTAKNANFYLQAGSAFAEMKIKGL 200

|||||||||||||::||:||:|||||||||||||||||||||||||||||

C.angolensis 151 WKAYNPGWPHCLDKRTVKDLDLNIKYSTAKNANFYLQAGSAFAEMKIKGL 200

H.sapiens 201 LDRKGLWRSLNEMKRIFNFRRTPAAEHAFEHWQEDAFFASQFLNGLNPVL 250

||||||||||||||||||||:|||||||||||||||||.|||||||||||

C.angolensis 201 LDRKGLWRSLNEMKRIFNFRKTPAAEHAFEHWQEDAFFTSQFLNGLNPVL 250

H.sapiens 251 IRRCHYLPKNFPVTDAMVASVLGPGTSLQAELEKGSLFLVDHGILSGIQT 300

||||||||||||||||||||||||||||||||||||||||||||||||.|

C.angolensis 251 IRRCHYLPKNFPVTDAMVASVLGPGTSLQAELEKGSLFLVDHGILSGIHT 300

H.sapiens 301 NVINGKPQFSAAPMTLLYQSPGCGPLLPLAIQLSQTPGPNSPIFLPTDDK 350

|||||||||||||||||||||||||||||||||||:||||||||||||||

C.angolensis 301 NVINGKPQFSAAPMTLLYQSPGCGPLLPLAIQLSQSPGPNSPIFLPTDDK 350

H.sapiens 351 WDWLLAKTWVRNAEFSFHEALTHLLHSHLLPEVFTLATLRQLPHCHPLFK 400

||||||||||||||||||||||||||||||||||||||||||||||||||

C.angolensis 351 WDWLLAKTWVRNAEFSFHEALTHLLHSHLLPEVFTLATLRQLPHCHPLFK 400

H.sapiens 401 LLIPHTRYTLHINTLARELLIVPGQVVDRSTGIGIEGFSELIQRNMKQLN 450

|||||||||||||.||||||||||||||||||||||||||||||||||||

C.angolensis 401 LLIPHTRYTLHINMLARELLIVPGQVVDRSTGIGIEGFSELIQRNMKQLN 450

H.sapiens 451 YSLLCLPEDIRTRGVEDIPGYYYRDDGMQIWGAVERFVSEIIGIYYPSDE 500

||||||||||||||||||||||||||||||||||||||||||||||||||

C.angolensis 451 YSLLCLPEDIRTRGVEDIPGYYYRDDGMQIWGAVERFVSEIIGIYYPSDE 500

H.sapiens 501 SVQDDRELQAWVREIFSKGFLNQESSGIPSSLETREALVQYVTMVIFTCS 550

.||||||||||||||||||||||||||||||||||||||:|||||||.||

C.angolensis 501 CVQDDRELQAWVREIFSKGFLNQESSGIPSSLETREALVRYVTMVIFNCS 550

H.sapiens 551 AKHAAVSAGQFDSCAWMPNLPPSMQLPPPTSKGLATCEGFIATLPPVNAT 600

||||||||||||||||||||||||||||||||||||.|||||||||||||

C.angolensis 551 AKHAAVSAGQFDSCAWMPNLPPSMQLPPPTSKGLATREGFIATLPPVNAT 600

H.sapiens 601 CDVILALWLLSKEPGDQRPLGTYPDEHFTEEAPRRSIATFQSRLAQISRG 650

||||:|||||||||||||||||||||||||||||||||.|||.||||||.

C.angolensis 601 CDVIIALWLLSKEPGDQRPLGTYPDEHFTEEAPRRSIAAFQSHLAQISRA 650

H.sapiens 651 IQERNQGLVLPYTYLDPPLIENSVSI 676

|||||:||||||||||||.|||||||

C.angolensis 651 IQERNRGLVLPYTYLDPPRIENSVSI 676

Ugandan red colobus (*Piliocolobus tephrosceles*)

H.sapiens 1 MAEFRVRVSTGEAFGAGTWDKVSVSIVGTRGESPPLPLDNLGKEFTAGAE 50

||:||||||||:||||||.|||||||||||.||||:|||||||||||.||

P.tephrosceles 1 MADFRVRVSTGKAFGAGTRDKVSVSIVGTREESPPMPLDNLGKEFTADAE 50

H.sapiens 51 EDFQVTLPEDVGRVLLLRVHKAPPVLPLLGPLAPDAWFCRWFQLTPPRGG 100

||||||||:|||:|||||||||||..|||..|.||||||||||||||:||

P.tephrosceles 51 EDFQVTLPKDVGQVLLLRVHKAPPARPLLRSLTPDAWFCRWFQLTPPQGG 100

H.sapiens 101 HLLFPCYQWLEGAGTLVLQEGTAKVSWADHHPVLQQQRQEELQARQEMYQ 150

.|||||||||||||:|||||||||||.|||||:|||||||||||||:|||

P.tephrosceles 101 PLLFPCYQWLEGAGSLVLQEGTAKVSRADHHPLLQQQRQEELQARQKMYQ 150

H.sapiens 151 WKAYNPGWPHCLDEKTVEDLELNIKYSTAKNANFYLQAGSAFAEMKIKGL 200

|||||||||||||::||:||:|||||||||||||||||||||||||||||

P.tephrosceles 151 WKAYNPGWPHCLDKRTVKDLDLNIKYSTAKNANFYLQAGSAFAEMKIKGL 200

H.sapiens 201 LDRKGLWRSLNEMKRIFNFRRTPAAEHAFEHWQEDAFFASQFLNGLNPVL 250

|:||||||||||||||||||:|||||||||||||||||||||||||||||

P.tephrosceles 201 LNRKGLWRSLNEMKRIFNFRKTPAAEHAFEHWQEDAFFASQFLNGLNPVL 250

H.sapiens 251 IRRCHYLPKNFPVTDAMVASVLGPGTSLQAELEKGSLFLVDHGILSGIQT 300

|.||||||||||||||||||||||||||||||||||||||||.|||||.|

P.tephrosceles 251 IHRCHYLPKNFPVTDAMVASVLGPGTSLQAELEKGSLFLVDHSILSGIHT 300

H.sapiens 301 NVINGKPQFSAAPMTLLYQSPGCGPLLPLAIQLSQTPGPNSPIFLPTDDK 350

|||||||||||||||||||||||||||||||||||:||||||||||:|||

P.tephrosceles 301 NVINGKPQFSAAPMTLLYQSPGCGPLLPLAIQLSQSPGPNSPIFLPSDDK 350

H.sapiens 351 WDWLLAKTWVRNAEFSFHEALTHLLHSHLLPEVFTLATLRQLPHCHPLFK 400

||||||||||||||||||||||||||||||.|||||||||||||||||||

P.tephrosceles 351 WDWLLAKTWVRNAEFSFHEALTHLLHSHLLSEVFTLATLRQLPHCHPLFK 400

H.sapiens 401 LLIPHTRYTLHINTLARELLIVPGQVVDRSTGIGIEGFSELIQRNMKQLN 450

|||||||||||||.|||||||||||||||||||||||||||||||||||:

P.tephrosceles 401 LLIPHTRYTLHINMLARELLIVPGQVVDRSTGIGIEGFSELIQRNMKQLH 450

H.sapiens 451 YSLLCLPEDIRTRGVEDIPGYYYRDDGMQIWGAVERFVSEIIGIYYPSDE 500

||||||||||||||||||||||||||||||||||||||||||||||||:|

P.tephrosceles 451 YSLLCLPEDIRTRGVEDIPGYYYRDDGMQIWGAVERFVSEIIGIYYPSNE 500

H.sapiens 501 SVQDDRELQAWVREIFSKGFLNQESSGIPSSLETREALVQYVTMVIFTCS 550

|||||||||||||||||||||||||||||||||||||||||||||||.||

P.tephrosceles 501 SVQDDRELQAWVREIFSKGFLNQESSGIPSSLETREALVQYVTMVIFNCS 550

H.sapiens 551 AKHAAVSAGQFDSCAWMPNLPPSMQLPPPTSKGLATCEGFIATLPPVNAT 600

||||||||||||||||||||||||||||||||||||.|||||||||||||

P.tephrosceles 551 AKHAAVSAGQFDSCAWMPNLPPSMQLPPPTSKGLATREGFIATLPPVNAT 600

H.sapiens 601 CDVILALWLLSKEPGDQRPLGTYPDEHFTEEAPRRSIATFQSRLAQISRG 650

||||:|||||||||||:||||||||||||||||||||..|||.||||||.

P.tephrosceles 601 CDVIIALWLLSKEPGDRRPLGTYPDEHFTEEAPRRSITAFQSHLAQISRA 650

H.sapiens 651 IQERNQGLVLPYTYLDPPLIENSVSI 676

|||||:||||||||||||||||||||

P.tephrosceles 651 IQERNRGLVLPYTYLDPPLIENSVSI 676

Golden snub-nosed monkey (*Rhinopithecus roxellana*)

H.sapiens 1 MAEFRVRVSTGEAFGAGTWDKVSVSIVGTRGESPPLPLDNLGKEFTAGAE 50

||:||||||||:||||||||||||||||||.||||:||||||||||||.|

R.roxellana 1 MADFRVRVSTGKAFGAGTWDKVSVSIVGTREESPPMPLDNLGKEFTAGTE 50

H.sapiens 51 EDFQVTLPEDVGRVLLLRVHKAPPVLPLLGPLAPDAWFCRWFQLTPPRGG 100

||||||||:|||:|||||:|||||.||||||||||||||||||||||:||

R.roxellana 51 EDFQVTLPKDVGQVLLLRLHKAPPALPLLGPLAPDAWFCRWFQLTPPQGG 100

H.sapiens 101 HLLFPCYQWLEGAGTLVLQEGTAKVSWADHHPVLQQQRQEELQARQEMYQ 150

.|||||||||||||:|||||||||||.|||||:|||||||||||||||||

R.roxellana 101 PLLFPCYQWLEGAGSLVLQEGTAKVSRADHHPLLQQQRQEELQARQEMYQ 150

H.sapiens 151 WKAYNPGWPHCLDEKTVEDLELNIKYSTAKNANFYLQAGSAFAEMKIKGL 200

|||||||||||||::||:||:|||||||||||||||||||||||||||||

R.roxellana 151 WKAYNPGWPHCLDKRTVKDLDLNIKYSTAKNANFYLQAGSAFAEMKIKGL 200

H.sapiens 201 LDRKGLWRSLNEMKRIFNFRRTPAAEHAFEHWQEDAFFASQFLNGLNPVL 250

||||||||||||||||||||:|||||||||||||||||||||||||||||

R.roxellana 201 LDRKGLWRSLNEMKRIFNFRKTPAAEHAFEHWQEDAFFASQFLNGLNPVL 250

H.sapiens 251 IRRCHYLPKNFPVTDAMVASVLGPGTSLQAELEKGSLFLVDHGILSGIQT 300

||||||||||||||||||||||||||||||||||||||||||||||||.|

R.roxellana 251 IRRCHYLPKNFPVTDAMVASVLGPGTSLQAELEKGSLFLVDHGILSGIHT 300

H.sapiens 301 NVINGKPQFSAAPMTLLYQSPGCGPLLPLAIQLSQTPGPNSPIFLPTDDK 350

|||||||||||||||||||||||||||||||||||:||||||||||||||

R.roxellana 301 NVINGKPQFSAAPMTLLYQSPGCGPLLPLAIQLSQSPGPNSPIFLPTDDK 350

H.sapiens 351 WDWLLAKTWVRNAEFSFHEALTHLLHSHLLPEVFTLATLRQLPHCHPLFK 400

||||||||||||||||||||||||||||||||||||||||||||||||||

R.roxellana 351 WDWLLAKTWVRNAEFSFHEALTHLLHSHLLPEVFTLATLRQLPHCHPLFK 400

H.sapiens 401 LLIPHTRYTLHINTLARELLIVPGQVVDRSTGIGIEGFSELIQRNMKQLN 450

|||||||||||||.||||||||||||||||||||||||||||||||||||

R.roxellana 401 LLIPHTRYTLHINMLARELLIVPGQVVDRSTGIGIEGFSELIQRNMKQLN 450

H.sapiens 451 YSLLCLPEDIRTRGVEDIPGYYYRDDGMQIWGAVERFVSEIIGIYYPSDE 500

||||||||||||||||||||||||||||:|||||||||||||||||||||

R.roxellana 451 YSLLCLPEDIRTRGVEDIPGYYYRDDGMRIWGAVERFVSEIIGIYYPSDE 500

H.sapiens 501 SVQDDRELQAWVREIFSKGFLNQESSGIPSSLETREALVQYVTMVIFTCS 550

||||||||||||||||||||||||||||||||.||||||||||||||.||

R.roxellana 501 SVQDDRELQAWVREIFSKGFLNQESSGIPSSLATREALVQYVTMVIFNCS 550

H.sapiens 551 AKHAAVSAGQFDSCAWMPNLPPSMQLPPPTSKGLATCEGFIATLPPVNAT 600

||||||||||||||||||||||||||||||||||||.|||||||||||||

R.roxellana 551 AKHAAVSAGQFDSCAWMPNLPPSMQLPPPTSKGLATREGFIATLPPVNAT 600

H.sapiens 601 CDVILALWLLSKEPGDQRPLGTYPDEHFTEEAPRRSIATFQSRLAQISRG 650

||||||||||||||||||||||||||||||||||||||.||||||||||.

R.roxellana 601 CDVILALWLLSKEPGDQRPLGTYPDEHFTEEAPRRSIAAFQSRLAQISRA 650

H.sapiens 651 IQERNQGLVLPYTYLDPPLIENSVSI 676

|||||:||||||||||||||||||||

R.roxellana 651 IQERNRGLVLPYTYLDPPLIENSVSI 676

Black-and-white snub-nosed monkey (*Rhinopithecus bieti*)

H.sapiens 1 MAEFRVRVSTGEAFGAGTWDKVSVSIVGTRGESPPLPLDNLGKEFTAGAE 50

||:||||||||:||||||||||||||||||.||||:||||||||||||.|

R.bieti 1 MADFRVRVSTGKAFGAGTWDKVSVSIVGTREESPPMPLDNLGKEFTAGTE 50

H.sapiens 51 EDFQVTLPEDVGRVLLLRVHKAPPVLPLLGPLAPDAWFCRWFQLTPPRGG 100

||||||||:|||:|||||:|||||.||||||||||||||||||||||:||

R.bieti 51 EDFQVTLPKDVGQVLLLRLHKAPPALPLLGPLAPDAWFCRWFQLTPPQGG 100

H.sapiens 101 HLLFPCYQWLEGAGTLVLQEGTAKVSWADHHPVLQQQRQEELQARQEMYQ 150

.|||||||||||||:|||||||||||.|||||:|||||||||||||||||

R.bieti 101 PLLFPCYQWLEGAGSLVLQEGTAKVSRADHHPLLQQQRQEELQARQEMYQ 150

H.sapiens 151 WKAYNPGWPHCLDEKTVEDLELNIKYSTAKNANFYLQAGSAFAEMKIKGL 200

|||||||||||||::||:||:|||||||||||||||||||||||||||||

R.bieti 151 WKAYNPGWPHCLDKRTVKDLDLNIKYSTAKNANFYLQAGSAFAEMKIKGL 200

H.sapiens 201 LDRKGLWRSLNEMKRIFNFRRTPAAEHAFEHWQEDAFFASQFLNGLNPVL 250

||||||||||||||||||||:|||||||||||||||||||||||||||||

R.bieti 201 LDRKGLWRSLNEMKRIFNFRKTPAAEHAFEHWQEDAFFASQFLNGLNPVL 250

H.sapiens 251 IRRCHYLPKNFPVTDAMVASVLGPGTSLQAELEKGSLFLVDHGILSGIQT 300

||||||||||||||||||||||||||||||||||||||||||||||||.|

R.bieti 251 IRRCHYLPKNFPVTDAMVASVLGPGTSLQAELEKGSLFLVDHGILSGIHT 300

H.sapiens 301 NVINGKPQFSAAPMTLLYQSPGCGPLLPLAIQLSQTPGPNSPIFLPTDDK 350

|||||||||||||||||||||||||||||||||||:||||||||||||||

R.bieti 301 NVINGKPQFSAAPMTLLYQSPGCGPLLPLAIQLSQSPGPNSPIFLPTDDK 350

H.sapiens 351 WDWLLAKTWVRNAEFSFHEALTHLLHSHLLPEVFTLATLRQLPHCHPLFK 400

||||||||||||||||||||||||||||||||||||||||||||||||||

R.bieti 351 WDWLLAKTWVRNAEFSFHEALTHLLHSHLLPEVFTLATLRQLPHCHPLFK 400

H.sapiens 401 LLIPHTRYTLHINTLARELLIVPGQVVDRSTGIGIEGFSELIQRNMKQLN 450

|||||||||||||.||||||||||||||||||||||||||||||||||||

R.bieti 401 LLIPHTRYTLHINMLARELLIVPGQVVDRSTGIGIEGFSELIQRNMKQLN 450

H.sapiens 451 YSLLCLPEDIRTRGVEDIPGYYYRDDGMQIWGAVERFVSEIIGIYYPSDE 500

||||||||||||||||||||||||||||:|||||||||||||||||||||

R.bieti 451 YSLLCLPEDIRTRGVEDIPGYYYRDDGMRIWGAVERFVSEIIGIYYPSDE 500

H.sapiens 501 SVQDDRELQAWVREIFSKGFLNQESSGIPSSLETREALVQYVTMVIFTCS 550

|||||||||||||||||||||||||||||||||||||||||||||||.||

R.bieti 501 SVQDDRELQAWVREIFSKGFLNQESSGIPSSLETREALVQYVTMVIFNCS 550

H.sapiens 551 AKHAAVSAGQFDSCAWMPNLPPSMQLPPPTSKGLATCEGFIATLPPVNAT 600

||||||||||||||||||||||||||||||||||||.|||||||||||||

R.bieti 551 AKHAAVSAGQFDSCAWMPNLPPSMQLPPPTSKGLATREGFIATLPPVNAT 600

H.sapiens 601 CDVILALWLLSKEPGDQRPLGTYPDEHFTEEAPRRSIATFQSRLAQISRG 650

||||||||||||||||||||||||||||||||||||||.|||.||||||.

R.bieti 601 CDVILALWLLSKEPGDQRPLGTYPDEHFTEEAPRRSIAAFQSHLAQISRA 650

H.sapiens 651 IQERNQGLVLPYTYLDPPLIENSVSI 676

|||||:||||||||||||||||||||

R.bieti 651 IQERNRGLVLPYTYLDPPLIENSVSI 676

Malayan flying lemur (*Galeopterus variegatus*)

H.sapiens 1 MAEFRVRVSTGEAFGAGTWDKVSVSIVGTRGESPPLPLDNLGKEFTAGAE 50

|.|:|||::||...|:||||.||||:|| |...||.||:|..|||

G.variegatus 1 MGEYRVRMATGSFLGSGTWDNVSVSLVG------PAGXDNFGKDFNRGAE 44

H.sapiens 51 EDFQVTLPEDVGRVLLLRVHKAPPVLPLLGPL-APDAWFCRWFQLTPPRG 99

|||:|||.:|||..||||||:|||.|| .|| |||||||.|| .|

G.variegatus 45 EDFEVTLLQDVGPGLLLRVHRAPPALP--RPLXAPDAWFCLWF--XP--- 87

H.sapiens 100 GHLLFPCYQWLEGAGTLVLQEGTAKVSWADHHPVLQQQRQEELQARQEMY 149

|.|||:|||||.|:|||::|.|||||||:||.|||||||||:|::|:|

G.variegatus 88 --LRFPCHQWLEGTGSLVLRQGAAKVSWADYHPKLQQQRQEELRAKEEIY 135

H.sapiens 150 QWKAYNPGWPHCLDEKTVEDLELNIKYSTAKNANFYLQAGSAFAEMKIKG 199

:||.|.||||.||||.||:||:|||:||.||:.||||:.||||||:||||

G.variegatus 136 RWKTYIPGWPRCLDEVTVKDLDLNIRYSIAKDTNFYLRGGSAFAELKIKG 185

H.sapiens 200 LLDRKGLWRSLNEMKRIFNFRRTPAAEHAFEHWQEDAFFASQFLNGLNPV 249

|||.|||||||.||:|:||||:|||||:.|||||||||||||||||||||

G.variegatus 186 LLDCKGLWRSLKEMRRMFNFRKTPAAEYVFEHWQEDAFFASQFLNGLNPV 235

H.sapiens 250 LIRRCHYLPKNFPVTDAMVASVLGPGTSLQAELEKGSLFLVDHGILSGIQ 299

|||||..||||||||||||:.|||||||||||||:|||||||||||||::

G.variegatus 236 LIRRCRCLPKNFPVTDAMVSPVLGPGTSLQAELERGSLFLVDHGILSGVR 285

H.sapiens 300 TNVINGKPQFSAAPMTLLYQSPGCGPLLPLAIQLSQTPGPNSPIFLPTDD 349

||||||:||||||||||||||||.|.||||||||||.||||||||||:||

G.variegatus 286 TNVINGRPQFSAAPMTLLYQSPGAGHLLPLAIQLSQIPGPNSPIFLPSDD 335

H.sapiens 350 KWDWLLAKTWVRNAEFSFHEALTHLLHSHLLPEVFTLATLRQLPHCHPLF 399

||||||||.||||||||.|||||||||.|||||||.||||||||||||||

G.variegatus 336 KWDWLLAKIWVRNAEFSVHEALTHLLHEHLLPEVFALATLRQLPHCHPLF 385

H.sapiens 400 KLLIPHTRYTLHINTLARELLIVPGQVVDRSTGIGIEGFSELIQRNMKQL 449

||||||||||||||||||:|||.||||||||||:|..||||||||||:||

G.variegatus 386 KLLIPHTRYTLHINTLARQLLIAPGQVVDRSTGLGPGGFSELIQRNMQQL 435

H.sapiens 450 NYSLLCLPEDIRTRGVEDIPGYYYRDDGMQIWGAVERFVSEIIGIYYPSD 499

|||:||||||||.||||||||||||||||||||||||||||:||:|||||

G.variegatus 436 NYSVLCLPEDIRARGVEDIPGYYYRDDGMQIWGAVERFVSEMIGVYYPSD 485

H.sapiens 500 ESVQDDRELQAWVREIFSKGFLNQESSGIPSSLETREALVQYVTMVIFTC 549

.||:||||||||||||||:|||.::|||:|||||||||||||||||||||

G.variegatus 486 MSVRDDRELQAWVREIFSEGFLGRQSSGMPSSLETREALVQYVTMVIFTC 535

H.sapiens 550 SAKHAAVSAGQFDSCAWMPNLPPSMQLPPPTSKGLATCEGFIATLPPVNA 599

||||:||||||||..||||||||:||||||||||.|..||||||||||||

G.variegatus 536 SAKHSAVSAGQFDFSAWMPNLPPTMQLPPPTSKGQARPEGFIATLPPVNA 585

H.sapiens 600 TCDVILALWLLSKEPGDQRPLGTYPDEHFTEEAPRRSIATFQSRLAQISR 649

|||||.|||:|||||||||||||||||||||||||||||.||||||||||

G.variegatus 586 TCDVITALWVLSKEPGDQRPLGTYPDEHFTEEAPRRSIAAFQSRLAQISR 635

H.sapiens 650 GIQERNQGLVLPYTYLDPPLIENSVSI 676

.|:|||:||.|||||||||||||||||

G.variegatus 636 DIRERNRGLALPYTYLDPPLIENSVSI 662

Gray mouse lemur (*Microcebus murinus*)

H.sapiens 1 MAEFRVRVSTGEAFGAGTWDKVSVSIVGTRGESPPLPLDNLGKEFTAGAE 50

|||||||||||:|.||||||||||:||||:|||||||||:|||.||||:|

M.murinus 1 MAEFRVRVSTGKACGAGTWDKVSVTIVGTQGESPPLPLDHLGKAFTAGSE 50

H.sapiens 51 EDFQVTLPEDVGRVLLLRVHKAPPVLPL-LGPLAPDAWFCRWFQLTPPRG 99

|||:||||:|||||||||||||||.||. ||||||||||||||||:||||

M.murinus 51 EDFEVTLPQDVGRVLLLRVHKAPPALPRPLGPLAPDAWFCRWFQLSPPRG 100

H.sapiens 100 GHLLFPCYQWLEGAGTLVLQEGTAKVSWADHHPVLQQQRQEELQARQEMY 149

..|.|||||||||||:|||:||.||||||||||||||||:||||||||||

M.murinus 101 APLRFPCYQWLEGAGSLVLREGAAKVSWADHHPVLQQQRREELQARQEMY 150

H.sapiens 150 QWKAYNPGWPHCLDEKTVEDLELNIKYSTAKNANFYLQAGSAFAEMKIKG 199

|||.|.|||||||||.||:||:||||||.|||.|||||..|||.|:||||

M.murinus 151 QWKTYIPGWPHCLDEVTVKDLDLNIKYSKAKNTNFYLQGYSAFTELKIKG 200

H.sapiens 200 LLDRKGLWRSLNEMKRIFNFRRTPAAEHAFEHWQEDAFFASQFLNGLNPV 249

|||||||||||.||:.||:||.|||||:..||||||||||||||||||||

M.murinus 201 LLDRKGLWRSLKEMRSIFDFRTTPAAEYVSEHWQEDAFFASQFLNGLNPV 250

H.sapiens 250 LIRRCHYLPKNFPVTDAMVASVLGPGTSLQAELEKGSLFLVDHGILSGIQ 299

||.||.:|||||||||||||.|||||||||||||:||||||||||||||:

M.murinus 251 LIHRCRHLPKNFPVTDAMVAPVLGPGTSLQAELERGSLFLVDHGILSGIK 300

H.sapiens 300 TNVINGKPQFSAAPMTLLYQSPGCGPLLPLAIQLSQTPGPNSPIFLPTDD 349

|||:||:|||||||||||||.||||||||||||||||||||||||||:||

M.murinus 301 TNVVNGRPQFSAAPMTLLYQRPGCGPLLPLAIQLSQTPGPNSPIFLPSDD 350

H.sapiens 350 KWDWLLAKTWVRNAEFSFHEALTHLLHSHLLPEVFTLATLRQLPHCHPLF 399

|||||||||||||||||||||:|||||:|||.||||||||.|||||||||

M.murinus 351 KWDWLLAKTWVRNAEFSFHEAITHLLHTHLLSEVFTLATLHQLPHCHPLF 400

H.sapiens 400 KLLIPHTRYTLHINTLARELLIVPGQVVDRSTGIGIEGFSELIQRNMKQL 449

|||:||||||||||||||||||.||||||||||:||.||||||||||:||

M.murinus 401 KLLVPHTRYTLHINTLARELLIAPGQVVDRSTGLGIGGFSELIQRNMEQL 450

H.sapiens 450 NYSLLCLPEDIRTRGVEDIPGYYYRDDGMQIWGAVERFVSEIIGIYYPSD 499

|||:||||||||.||||||||||||||||||||||||||||||.||||||

M.murinus 451 NYSVLCLPEDIRARGVEDIPGYYYRDDGMQIWGAVERFVSEIIDIYYPSD 500

H.sapiens 500 ESVQDDRELQAWVREIFSKGFLNQESSGIPSSLETREALVQYVTMVIFTC 549

.||:||||||||||||||:|||::||||:|||||||||||||||||||||

M.murinus 501 LSVRDDRELQAWVREIFSEGFLSRESSGMPSSLETREALVQYVTMVIFTC 550

H.sapiens 550 SAKHAAVSAGQFDSCAWMPNLPPSMQLPPPTSKGLATCEGFIATLPPVNA 599

||||:|||||||||||||||||.:|||||||.||.|..||||||||||||

M.murinus 551 SAKHSAVSAGQFDSCAWMPNLPSTMQLPPPTIKGQARPEGFIATLPPVNA 600

H.sapiens 600 TCDVILALWLLSKEPGDQRPLGTYPDEHFTEEAPRRSIATFQSRLAQISR 649

|||:::|||||||||||:|||||||||||||||||||||.||||||||||

M.murinus 601 TCDIVIALWLLSKEPGDRRPLGTYPDEHFTEEAPRRSIAAFQSRLAQISR 650

H.sapiens 650 GIQERNQGLVLPYTYLDPPLIENSVSI 676

||||||:||.|||||||||||||||||

M.murinus 651 GIQERNRGLALPYTYLDPPLIENSVSI 677

Ring-tailed lemur (Lemur catta)

H.sapiens 1 MAEFRVRVSTGEAFGAGTWDKVSVSIVGTRGESPPLPLDNLGKEFTAGAE 50

|||||||||||:|.|||||||||::||||||||||||||:||||||||||

L.catta 1 MAEFRVRVSTGKACGAGTWDKVSITIVGTRGESPPLPLDHLGKEFTAGAE 50

H.sapiens 51 EDFQVTLPEDVGRVLLLRVHKAPPVLPL-LGPLAPDAWFCRWFQLTPPRG 99

|||:||||:|||.|||||||||||.||. ||||||||||||||||.||||

L.catta 51 EDFEVTLPQDVGPVLLLRVHKAPPALPRPLGPLAPDAWFCRWFQLAPPRG 100

H.sapiens 100 GHLLFPCYQWLEGAGTLVLQEGTAKVSWADHHPVLQQQRQEELQARQEMY 149

..|.|||||||||||:|||:||||||.|||||||||||||||||||||.|

L.catta 101 APLRFPCYQWLEGAGSLVLREGTAKVPWADHHPVLQQQRQEELQARQETY 150

H.sapiens 150 QWKAYNPGWPHCLDEKTVEDLELNIKYSTAKNANFYLQAGSAFAEMKIKG 199

|||.|.|||||||||.||:||:||||||||||.|||||..|||.|:||||

L.catta 151 QWKTYIPGWPHCLDEVTVKDLDLNIKYSTAKNTNFYLQGFSAFTELKIKG 200

H.sapiens 200 LLDRKGLWRSLNEMKRIFNFRRTPAAEHAFEHWQEDAFFASQFLNGLNPV 249

|||||||||||.||:||||||.|||||:..||||||||||||||||||||

L.catta 201 LLDRKGLWRSLKEMRRIFNFRTTPAAEYVSEHWQEDAFFASQFLNGLNPV 250

H.sapiens 250 LIRRCHYLPKNFPVTDAMVASVLGPGTSLQAELEKGSLFLVDHGILSGIQ 299

||.||.||||||||||||||.|||||||||||||:||||||||||||||:

L.catta 251 LIHRCRYLPKNFPVTDAMVAPVLGPGTSLQAELERGSLFLVDHGILSGIK 300

H.sapiens 300 TNVINGKPQFSAAPMTLLYQSPGCGPLLPLAIQLSQTPGPNSPIFLPTDD 349

|||:||:|||||||||||||.||||.|||||||||||||||||||||:||

L.catta 301 TNVVNGRPQFSAAPMTLLYQRPGCGSLLPLAIQLSQTPGPNSPIFLPSDD 350

H.sapiens 350 KWDWLLAKTWVRNAEFSFHEALTHLLHSHLLPEVFTLATLRQLPHCHPLF 399

||||||||||||||||||||||||||||||||||||||||||||||||||

L.catta 351 KWDWLLAKTWVRNAEFSFHEALTHLLHSHLLPEVFTLATLRQLPHCHPLF 400

H.sapiens 400 KLLIPHTRYTLHINTLARELLIVPGQVVDRSTGIGIEGFSELIQRNMKQL 449

|||:||||||||||||||||||.||||||||||:||.||||||||||:||

L.catta 401 KLLVPHTRYTLHINTLARELLISPGQVVDRSTGLGIGGFSELIQRNMEQL 450

H.sapiens 450 NYSLLCLPEDIRTRGVEDIPGYYYRDDGMQIWGAVERFVSEIIGIYYPSD 499

|||:|||||||:||||||||||||||||||||||||.||||:||||||||

L.catta 451 NYSVLCLPEDIQTRGVEDIPGYYYRDDGMQIWGAVECFVSEMIGIYYPSD 500

H.sapiens 500 ESVQDDRELQAWVREIFSKGFLNQESSGIPSSLETREALVQYVTMVIFTC 549

.||:||||||||||||||:|||::||||:|||||||||||||||||||||

L.catta 501 VSVRDDRELQAWVREIFSEGFLSRESSGMPSSLETREALVQYVTMVIFTC 550

H.sapiens 550 SAKHAAVSAGQFDSCAWMPNLPPSMQLPPPTSKGLATCEGFIATLPPVNA 599

|||||||||||||||||||||||:||||||||||.|..||||||||||||

L.catta 551 SAKHAAVSAGQFDSCAWMPNLPPTMQLPPPTSKGQARPEGFIATLPPVNA 600

H.sapiens 600 TCDVILALWLLSKEPGDQRPLGTYPDEHFTEEAPRRSIATFQSRLAQISR 649

||||::|||||||||||:|||||||||||||||||||||.||||||||||

L.catta 601 TCDVVIALWLLSKEPGDRRPLGTYPDEHFTEEAPRRSIAAFQSRLAQISR 650

H.sapiens 650 GIQERNQGLVLPYTYLDPPLIENSVSI 676

||:|||:||.|||||||||||||||||

L.catta 651 GIRERNRGLALPYTYLDPPLIENSVSI 677

Green monkey (*Chlorocebus sabaeus*)

H.sapiens 1 MAEFRVRVSTGEAFGAGTWDKVSVSIVGTRGESPPLPLDNLGKEFTAGAE 50

||:||||||||:||||||||||||||||||.|||||||||||||||||||

C.sabaeus 1 MADFRVRVSTGKAFGAGTWDKVSVSIVGTREESPPLPLDNLGKEFTAGAE 50

H.sapiens 51 EDFQVTLPEDVGRVLLLRVHKAPPVLPLLGPLAPDAWFCRWFQLTPPRGG 100

||||||||:|||:|||||||||||.|||||||||||||||||:||||:||

C.sabaeus 51 EDFQVTLPKDVGQVLLLRVHKAPPALPLLGPLAPDAWFCRWFRLTPPQGG 100

H.sapiens 101 HLLFPCYQWLEGAGTLVLQEGTAKVSWADHHPVLQQQRQEELQARQEMYQ 150

.|||||||||||||:|||||||||||.|||||:|||||||||||||||||

Grünmeerkatz 101 PLLFPCYQWLEGAGSLVLQEGTAKVSRADHHPLLQQQRQEELQARQEMYQ 150

H.sapiens 151 WKAYNPGWPHCLDEKTVEDLELNIKYSTAKNANFYLQAGSAFAEMKIKGL 200

||.||||||||||::||:||:|||||||||||||||||||||||||||||

C.sabaeus 151 WKVYNPGWPHCLDKRTVKDLDLNIKYSTAKNANFYLQAGSAFAEMKIKGL 200

H.sapiens 201 LDRKGLWRSLNEMKRIFNFRRTPAAEHAFEHWQEDAFFASQFLNGLNPVL 250

||||||||||||||||||||:||||||.||||||||||||||||||||||

C.sabaeus 201 LDRKGLWRSLNEMKRIFNFRKTPAAEHTFEHWQEDAFFASQFLNGLNPVL 250

H.sapiens 251 IRRCHYLPKNFPVTDAMVASVLGPGTSLQAELEKGSLFLVDHGILSGIQT 300

||||||||||||||||||||||||||||||||||||||||||||||||.|

C.sabaeus 251 IRRCHYLPKNFPVTDAMVASVLGPGTSLQAELEKGSLFLVDHGILSGIHT 300

H.sapiens 301 NVINGKPQFSAAPMTLLYQSPGCGPLLPLAIQLSQTPGPNSPIFLPTDDK 350

|||||||||||||||||||||||||||||||||||:||||||||||||||

C.sabaeus 301 NVINGKPQFSAAPMTLLYQSPGCGPLLPLAIQLSQSPGPNSPIFLPTDDK 350

H.sapiens 351 WDWLLAKTWVRNAEFSFHEALTHLLHSHLLPEVFTLATLRQLPHCHPLFK 400

||||||||||||||||||||||||||||||||||||||||||||||||||

C.sabaeus 351 WDWLLAKTWVRNAEFSFHEALTHLLHSHLLPEVFTLATLRQLPHCHPLFK 400

H.sapiens 401 LLIPHTRYTLHINTLARELLIVPGQVVDR--------------------- 429

|||||||||:|||.|||||||||||||||

C.sabaeus 401 LLIPHTRYTVHINMLARELLIVPGQVVDRVRAVLGWEWAGHSFNVRNLRS 450

H.sapiens 430 ------------------------------STGIGIEGFSELIQRNMKQL 449

|||||:||||||||||||||

C.sabaeus 451 WGSSGLSPRHQCYLLLSGARSAGPKSPVRQSTGIGVEGFSELIQRNMKQL 500

H.sapiens 450 NYSLLCLPEDIRTRGVEDIPGYYYRDDGMQIWGAVERFVSEIIGIYYPSD 499

||||||||||||||||||||||||||||||||||||||||||||||||||

C.sabaeus 501 NYSLLCLPEDIRTRGVEDIPGYYYRDDGMQIWGAVERFVSEIIGIYYPSD 550

H.sapiens 500 ESVQDDRELQAWVREIFSKGFLNQESSGIPSSLETREALVQYVTMVIFTC 549

||||||||||||||||||||||||||||||||||||||||||||||||.|

C.sabaeus 551 ESVQDDRELQAWVREIFSKGFLNQESSGIPSSLETREALVQYVTMVIFNC 600

H.sapiens 550 SAKHAAVSAGQFDSCAWMPNLPPSMQLPPPTSKGLATCEGFIATLPPVNA 599

|||||||||||||:|||||||||||||||||||||||.||||||||||||

C.sabaeus 601 SAKHAAVSAGQFDACAWMPNLPPSMQLPPPTSKGLATREGFIATLPPVNA 650

H.sapiens 600 TCDVILALWLLSKEPGDQRPLGTYPDEHFTEEAPRRSIATFQSRLAQISR 649

|||||||||||||||||:|||||||||||||||||||||.|||.||||||

C.sabaeus 651 TCDVILALWLLSKEPGDRRPLGTYPDEHFTEEAPRRSIAAFQSHLAQISR 700

H.sapiens 650 GIQERNQGLVLPYTYLDPPLIENSVSI 676

.|||||:||||||||||||||||||||

C.sabaeus 701 AIQERNRGLVLPYTYLDPPLIENSVSI 727

Coquerel's sifaka (*Propithecus coquereli*)

H.sapiens 1 MAEFRVRVSTGEAFGAGTWDKVSVSIVGTRGESPPLPLDNLGKEFTAGAE 50

|||||||||||.|.||||||||||:|||||||||.||||:.|||||||||

P.coquereli 1 MAEFRVRVSTGGACGAGTWDKVSVTIVGTRGESPALPLDHFGKEFTAGAE 50

H.sapiens 51 EDFQVTLPEDVGRVLLLRVHKAPPVLPL-LGPLAPDAWFCRWFQLTPPRG 99

|||:||||:|||.|||||||||||.||. ||||||||||||||||.||||

P.coquereli 51 EDFEVTLPQDVGPVLLLRVHKAPPALPRPLGPLAPDAWFCRWFQLAPPRG 100

H.sapiens 100 GHLLFPCYQWLEGAGTLVLQEGTAKVSWADHHPVLQQQRQEELQARQEMY 149

..|.|||||||||||:|||:||.|||||||||||||||||||||||||||

P.coquereli 101 APLRFPCYQWLEGAGSLVLREGAAKVSWADHHPVLQQQRQEELQARQEMY 150

H.sapiens 150 QWKAYNPGWPHCLDEKTVEDLELNIKYSTAKNANFYLQAGSAFAEMKIKG 199

|||.|.|||||||||.||:||:||||||||||.|||||..|||.|:||||

P.coquereli 151 QWKTYIPGWPHCLDEVTVKDLDLNIKYSTAKNTNFYLQGLSAFTELKIKG 200

H.sapiens 200 LLDRKGLWRSLNEMKRIFNFRRTPAAEHAFEHWQEDAFFASQFLNGLNPV 249

|||||||||||.||:||||||.|||||:..||||||||||||||||||||

P.coquereli 201 LLDRKGLWRSLKEMRRIFNFRTTPAAEYVSEHWQEDAFFASQFLNGLNPV 250

H.sapiens 250 LIRRCHYLPKNFPVTDAMVASVLGPGTSLQAELEKGSLFLVDHGILSGIQ 299

||.||.||||||||||||||.|||||||||||||:||||||||||||||:

P.coquereli 251 LIHRCRYLPKNFPVTDAMVAPVLGPGTSLQAELERGSLFLVDHGILSGIK 300

H.sapiens 300 TNVINGKPQFSAAPMTLLYQSPGCGPLLPLAIQLSQTPGPNSPIFLPTDD 349

|||:||:|||||||||||||.||||.|||||||||||||||||||||:||

P.coquereli 301 TNVVNGRPQFSAAPMTLLYQRPGCGSLLPLAIQLSQTPGPNSPIFLPSDD 350

H.sapiens 350 KWDWLLAKTWVRNAEFSFHEALTHLLHSHLLPEVFTLATLRQLPHCHPLF 399

|||||||||||||||||||||||||||||||||||:||.|||||||||||

P.coquereli 351 KWDWLLAKTWVRNAEFSFHEALTHLLHSHLLPEVFSLAMLRQLPHCHPLF 400

H.sapiens 400 KLLIPHTRYTLHINTLARELLIVPGQVVDRSTGIGIEGFSELIQRNMKQL 449

|||:||||||||||||||||||.||||||||||:||.||||||||||:||

P.coquereli 401 KLLVPHTRYTLHINTLARELLISPGQVVDRSTGLGIGGFSELIQRNMEQL 450

H.sapiens 450 NYSLLCLPEDIRTRGVEDIPGYYYRDDGMQIWGAVERFVSEIIGIYYPSD 499

|||:|||||||||||||||||||||||||||||||||||||:||||||||

P.coquereli 451 NYSVLCLPEDIRTRGVEDIPGYYYRDDGMQIWGAVERFVSEMIGIYYPSD 500

H.sapiens 500 ESVQDDRELQAWVREIFSKGFLNQESSGIPSSLETREALVQYVTMVIFTC 549

.||:||||||||||||||:|||::||||:|||||||||||||||||||||

P.coquereli 501 VSVRDDRELQAWVREIFSEGFLSRESSGMPSSLETREALVQYVTMVIFTC 550

H.sapiens 550 SAKHAAVSAGQFDSCAWMPNLPPSMQLPPPTSKGLATCEGFIATLPPVNA 599

||||:||||||||||||||||||:||||||||||.|..||||||||||||

P.coquereli 551 SAKHSAVSAGQFDSCAWMPNLPPTMQLPPPTSKGQARPEGFIATLPPVNA 600

H.sapiens 600 TCDVILALWLLSKEPGDQRPLGTYPDEHFTEEAPRRSIATFQSRLAQISR 649

||||::|||||||||||:|||||||||||||||||||||.||||||||||

P.coquereli 601 TCDVVIALWLLSKEPGDRRPLGTYPDEHFTEEAPRRSIAAFQSRLAQISR 650

H.sapiens 650 GIQERNQGLVLPYTYLDPPLIENSVSI 676

||:|||||||||||||||||||||:||

P.coquereli 651 GIRERNQGLVLPYTYLDPPLIENSISI 677

Northern greater galago (*Otolemur garnettii*)

H.sapiens 1 MAEFRVRVSTGEAFGAGTWDKVSVSIVGTRGESPPLPLDNLGKEFTAGAE 50

||||||.||||||..|||||||||:||||||||||||||:.||||:||||

O.garnettii 1 MAEFRVTVSTGEACRAGTWDKVSVTIVGTRGESPPLPLDHFGKEFSAGAE 50

H.sapiens 51 EDFQVTLPEDVGRVLLLRVHKAPPV-LPLLGPLAPDAWFCRWFQLTPPRG 99

|||:||||:|||:|||||||||||. ..|||.|.|||||||||||..|:|

O.garnettii 51 EDFKVTLPQDVGQVLLLRVHKAPPASRGLLGHLVPDAWFCRWFQLALPQG 100

H.sapiens 100 GHLL-FPCYQWLEGAGTLVLQEGTAKVSWADHHPVLQQQRQEELQARQEM 148

..|| |||||||||..:|||:||.|||||||||||.||||||||:|||:|

O.garnettii 101 APLLRFPCYQWLEGTRSLVLREGAAKVSWADHHPVFQQQRQEELKARQDM 150

H.sapiens 149 YQWKAYNPGWPHCLDEKTVEDLELNIKYSTAKNANFYLQAGSAFAEMKIK 198

|.||.|.|||||||:|.||:||:|||||||.||.|||||..||||:::||

O.garnettii 151 YHWKTYIPGWPHCLNEMTVKDLDLNIKYSTVKNTNFYLQGISAFADLRIK 200

H.sapiens 199 GLLDRKGLWRSLNEMKRIFNFRRTPAAEHAFEHWQEDAFFASQFLNGLNP 248

|||.||||||||:||||||:|:||||||:.:|||:|||||||||||||||

O.garnettii 201 GLLGRKGLWRSLHEMKRIFSFQRTPAAEYVYEHWKEDAFFASQFLNGLNP 250

H.sapiens 249 VLIRRCHYLPKNFPVTDAMVASVLGPGTSLQAELEKGSLFLVDHGILSGI 298

||||||.||||||||||||||.||||||:||||||:||||||||||||||

O.garnettii 251 VLIRRCRYLPKNFPVTDAMVAPVLGPGTNLQAELERGSLFLVDHGILSGI 300

H.sapiens 299 QTNVINGKPQFSAAPMTLLYQSPGCGPLLPLAIQLSQTPGPNSPIFLPTD 348

|||||||||||||||||||||.||.|||||||||||||||||||||||:|

O.garnettii 301 QTNVINGKPQFSAAPMTLLYQRPGSGPLLPLAIQLSQTPGPNSPIFLPSD 350

H.sapiens 349 DKWDWLLAKTWVRNAEFSFHEALTHLLHSHLLPEVFTLATLRQLPHCHPL 398

|||||||||||||||||||||||||||:||||||||||||||||||||||

O.garnettii 351 DKWDWLLAKTWVRNAEFSFHEALTHLLYSHLLPEVFTLATLRQLPHCHPL 400

H.sapiens 399 FKLLIPHTRYTLHINTLARELLIVPGQVVDRSTGIGIEGFSELIQRNMKQ 448

|||||||||||||||||||||||.|.||||||||:||.||||||||:|:|

O.garnettii 401 FKLLIPHTRYTLHINTLARELLIAPRQVVDRSTGLGIGGFSELIQRSMEQ 450

H.sapiens 449 LNYSLLCLPEDIRTRGVEDIPGYYYRDDGMQIWGAVERFVSEIIGIYYPS 498

||||.||||||||.|||||||||||||||||||.||||||||||.|||||

O.garnettii 451 LNYSDLCLPEDIRNRGVEDIPGYYYRDDGMQIWSAVERFVSEIINIYYPS 500

H.sapiens 499 DESVQDDRELQAWVREIFSKGFLNQESSGIPSSLETREALVQYVTMVIFT 548

|.|||||||||||||||||:|||:::|||:||||||||||||||||||||

O.garnettii 501 DMSVQDDRELQAWVREIFSEGFLSRQSSGVPSSLETREALVQYVTMVIFT 550

H.sapiens 549 CSAKHAAVSAGQFDSCAWMPNLPPSMQLPPPTSKGLATCEGFIATLPPVN 598

||||||||||||||.|||||||||:||||||||||.|..||:||||||||

O.garnettii 551 CSAKHAAVSAGQFDFCAWMPNLPPTMQLPPPTSKGQARSEGYIATLPPVN 600

H.sapiens 599 ATCDVILALWLLSKEPGDQRPLGTYPDEHFTEEAPRRSIATFQSRLAQIS 648

||||||:|||||||||||:||||||||||||||||::|||.|||.|||||

O.garnettii 601 ATCDVIIALWLLSKEPGDRRPLGTYPDEHFTEEAPQQSIAAFQSHLAQIS 650

H.sapiens 649 RGIQERNQGLVLPYTYLDPPLIENSVSI 676

||||||||||.|||||||||.|||||||

O.garnettii 651 RGIQERNQGLALPYTYLDPPHIENSVSI 678

Arctic ground squirrel (*Urocitellus parryii*)

H.sapiens 1 MAEFRVRVSTGEAFGAGTWDKVSVSIVGTRGESPPLPLDNLGKEFTAGAE 50

||:||:|||||:|.||||||||||||||.|||||.||||:||||||||||

U.parryii 1 MAKFRIRVSTGKACGAGTWDKVSVSIVGMRGESPLLPLDHLGKEFTAGAE 50

H.sapiens 51 EDFQVTLPEDVGRVLLLRVHKAPPVLPL-LGPLAP-DAWFCRWFQLTPPR 98

|||:||||.|||.||||||||||..||. |||||| ||||||||||.||.

U.parryii 51 EDFEVTLPHDVGPVLLLRVHKAPLALPRPLGPLAPNDAWFCRWFQLEPPG 100

H.sapiens 99 GGHLLFPCYQWLEGAGTLVLQEGTAKVSWADHHPVLQQQRQEELQARQEM 148

|..|.|||||||||||.|:|:||.||||||||||:|::|||||||:||.|

U.parryii 101 GVPLRFPCYQWLEGAGDLLLREGAAKVSWADHHPILRRQRQEELQSRQRM 150

H.sapiens 149 YQWKAYNPGWPHCLDEKTVEDLELNIKYSTAKNANFYLQAGSAFAEMKIK 198

|.||.|:||||||||:.|.:||:||:|||..||..||::..|||||:|||

U.parryii 151 YCWKTYSPGWPHCLDQATXKDLDLNVKYSVVKNTKFYMKGSSAFAELKIK 200

H.sapiens 199 GLLDRKGLWRSLNEMKRIFNFRRTPAAEHAFEHWQEDAFFASQFLNGLNP 248

|||||.||||||.||:|:||||::||||:.|||||:|.||||||||||||

U.parryii 201 GLLDRTGLWRSLREMRRMFNFRKSPAAEYVFEHWQDDTFFASQFLNGLNP 250

H.sapiens 249 VLIRRCHYLPKNFPVTDAMVASVLGPGTSLQAELEKGSLFLVDHGILSGI 298

|||:||..|||||||||.|||.:||||||||||||||||||||||||||:

U.parryii 251 VLIQRCRSLPKNFPVTDDMVAPILGPGTSLQAELEKGSLFLVDHGILSGV 300

H.sapiens 299 QTNVINGKPQFSAAPMTLLYQSPGCGPLLPLAIQLSQTPGPNSPIFLPTD 348

:|||||||||||||||||||||||.|||||||||||||||||:|||||||

U.parryii 301 RTNVINGKPQFSAAPMTLLYQSPGNGPLLPLAIQLSQTPGPNNPIFLPTD 350

H.sapiens 349 DKWDWLLAKTWVRNAEFSFHEALTHLLHSHLLPEVFTLATLRQLPHCHPL 398

||||||||||||||||||.|||||||||:||||||||||||||||.||||

U.parryii 351 DKWDWLLAKTWVRNAEFSVHEALTHLLHAHLLPEVFTLATLRQLPSCHPL 400

H.sapiens 399 FKLLIPHTRYTLHINTLARELLIVPGQVVDRSTGIGIEGFSELIQRNMKQ 448

|||||||||||||||||||||||.||||||||||:||.||||||||||:|

U.parryii 401 FKLLIPHTRYTLHINTLARELLIAPGQVVDRSTGLGIGGFSELIQRNMEQ 450

H.sapiens 449 LNYSLLCLPEDIRTRGVEDIPGYYYRDDGMQIWGAVERFVSEIIGIYYPS 498

|:||:|||||||..|||||||.|||||||||||||||||||||:|||||:

U.parryii 451 LSYSVLCLPEDIXARGVEDIPNYYYRDDGMQIWGAVERFVSEIVGIYYPN 500

H.sapiens 499 DESVQDDRELQAWVREIFSKGFLNQESSGIPSSLETREALVQYVTMVIFT 548

|.||.||:|||||||||||:|||::||||:||||:|:||||:||||||||

U.parryii 501 DASVXDDQELQAWVREIFSRGFLDRESSGVPSSLDTQEALVRYVTMVIFT 550

H.sapiens 549 CSAKHAAVSAGQFDSCAWMPNLPPSMQLPPPTSKGLATCEGFIATLPPVN 598

|||:|:|||||||||..|||||||:||||||||||.|..|||||||||||

U.parryii 551 CSAQHSAVSAGQFDSSVWMPNLPPTMQLPPPTSKGQAQPEGFIATLPPVN 600

H.sapiens 599 ATCDVILALWLLSKEPGDQRPLGTYPDEHFTEEAPRRSIATFQSRLAQIS 648

||||:|:||||||:|||||||||||||||||||||:||||.||:.|.|||

U.parryii 601 ATCDIIIALWLLSREPGDQRPLGTYPDEHFTEEAPQRSIAAFQNHLTQIS 650

H.sapiens 649 RGIQERNQGLVLPYTYLDPPLIENSVSI 676

|.|:||||.|.|||.|||||||||||||

U.parryii 651 RDIKERNQKLALPYPYLDPPLIENSVSI 678

Yellow-bellied marmot (*Marmota flaviventris*)

H.sapiens 1 MAEFRVRVSTGEAFGAGTWDKVSVSIVGTRGESPPLPLDNLGKEFTAGAE 50

||:||:|||||:|.||||||||||.|||.|||||.||||:||||||||||

M.flaviventris 1 MAKFRIRVSTGKACGAGTWDKVSVCIVGRRGESPLLPLDHLGKEFTAGAE 50

H.sapiens 51 EDFQVTLPEDVGRVLLLRVHKAPPVLPL-LGPLAP-DAWFCRWFQLTPPR 98

|||:||||.|||.||||.|||||..||. |||||| ||||||||||.||.

M.flaviventris 51 EDFEVTLPHDVGPVLLLHVHKAPLALPRPLGPLAPDDAWFCRWFQLEPPG 100

H.sapiens 99 GGHLLFPCYQWLEGAGTLVLQEGTAKVSWADHHPVLQQQRQEELQARQEM 148

|..|.|||||||||||.|:|:||.|||||||||.:|::|||||||:||.|

M.flaviventris 101 GIPLRFPCYQWLEGAGDLLLREGAAKVSWADHHSILRRQRQEELQSRQRM 150

H.sapiens 149 YQWKAYNPGWPHCLDEKTVEDLELNIKYSTAKNANFYLQAGSAFAEMKIK 198

|:||.|:||||||||:.|.:||:||:|||..||..||::..|||||:|||

M.flaviventris 151 YRWKTYSPGWPHCLDQATAKDLDLNVKYSIVKNTKFYMKGSSAFAELKIK 200

H.sapiens 199 GLLDRKGLWRSLNEMKRIFNFRRTPAAEHAFEHWQEDAFFASQFLNGLNP 248

|||||.||||||.||:|:||||::||||:.|||||:|.||||||||||||

M.flaviventris 201 GLLDRTGLWRSLREMRRMFNFRKSPAAEYVFEHWQDDTFFASQFLNGLNP 250

H.sapiens 249 VLIRRCHYLPKNFPVTDAMVASVLGPGTSLQAELEKGSLFLVDHGILSGI 298

|||.||..|||||||||.|||.:||||||||||||||||||||||||||:

M.flaviventris 251 VLIHRCRSLPKNFPVTDDMVAPILGPGTSLQAELEKGSLFLVDHGILSGV 300

H.sapiens 299 QTNVINGKPQFSAAPMTLLYQSPGCGPLLPLAIQLSQTPGPNSPIFLPTD 348

.|||||||||||||||||||||||.|||||||||||||||||:|||||||

M.flaviventris 301 STNVINGKPQFSAAPMTLLYQSPGNGPLLPLAIQLSQTPGPNNPIFLPTD 350

H.sapiens 349 DKWDWLLAKTWVRNAEFSFHEALTHLLHSHLLPEVFTLATLRQLPHCHPL 398

||||||||||||||||||.|||||||||:||||||||||||||||.||||

M.flaviventris 351 DKWDWLLAKTWVRNAEFSVHEALTHLLHAHLLPEVFTLATLRQLPSCHPL 400

H.sapiens 399 FKLLIPHTRYTLHINTLARELLIVPGQVVDRSTGIGIEGFSELIQRNMKQ 448

||||||||||||:||||||||||.||||||||||:||.||||||||||:|

M.flaviventris 401 FKLLIPHTRYTLYINTLARELLIAPGQVVDRSTGLGIGGFSELIQRNMEQ 450

H.sapiens 449 LNYSLLCLPEDIRTRGVEDIPGYYYRDDGMQIWGAVERFVSEIIGIYYPS 498

|:||:||||||||.|||||||.|||||||||||||||||||||:||||||

M.flaviventris 451 LSYSVLCLPEDIRARGVEDIPNYYYRDDGMQIWGAVERFVSEIVGIYYPS 500

H.sapiens 499 DESVQDDRELQAWVREIFSKGFLNQESSGIPSSLETREALVQYVTMVIFT 548

|.||:||:|||||||||||||||::||||:||||:|:||||:||||||||

M.flaviventris 501 DASVRDDQELQAWVREIFSKGFLDRESSGVPSSLDTQEALVRYVTMVIFT 550

H.sapiens 549 CSAKHAAVSAGQFDSCAWMPNLPPSMQLPPPTSKGLATCEGFIATLPPVN 598

|||:|:|||||||||..|||||||:||||||||||.|..|||||||||||

M.flaviventris 551 CSAQHSAVSAGQFDSSVWMPNLPPTMQLPPPTSKGQAQPEGFIATLPPVN 600

H.sapiens 599 ATCDVILALWLLSKEPGDQRPLGTYPDEHFTEEAPRRSIATFQSRLAQIS 648

||||:|:||||||:|||||||||||||||||||||:||||.||:.|.|||

M.flaviventris 601 ATCDIIIALWLLSREPGDQRPLGTYPDEHFTEEAPQRSIAAFQNHLTQIS 650

H.sapiens 649 RGIQERNQGLVLPYTYLDPPLIENSVSI 676

|.|:||||.|.|||.||||.||||||||

M.flaviventris 651 RDIKERNQKLALPYPYLDPSLIENSVSI 678

Banner-tailed kangaroo rat (*Dipodomys spectabilis*)

H.sapiens 1 MAEFRVRVSTGEAFGAGTWDKVSVSIVGTRGESPPLPLDNLGKEFTAGAE 50

||:||||||||||.|||||||||||:|||.|||..||||.||:||:||||

D.spectabilis 1 MAKFRVRVSTGEACGAGTWDKVSVSLVGTEGESALLPLDRLGREFSAGAE 50

H.sapiens 51 EDFQVTLPEDVGRVLLLRVHKAPPVLPLLGPLAPDAWFCRWFQLTPPRGG 100

|||:||||:|||:|||||.||:.|..|||.|.||||||||||||..|:|.

D.spectabilis 51 EDFEVTLPQDVGQVLLLRAHKSLPAQPLLCPPAPDAWFCRWFQLELPQGA 100

H.sapiens 101 HLLFPCYQWLEGAGTLVLQEGTAKVSWADHHPVLQQQRQEELQARQEMYQ 150

.|.|||||||||||.|||:||||||||||.||.||||||:||:.|:.||.

D.spectabilis 101 ALRFPCYQWLEGAGHLVLREGTAKVSWADRHPTLQQQRQKELEVRRAMYS 150

H.sapiens 151 WKAYNPGWPHCLDEKTVEDLELNIKYSTAKNANFYLQAGSAFAEMKIKGL 200

||.|..|||.|||:.||::|:||||||..|||||||:|.|||.|:|.|||

D.spectabilis 151 WKNYIQGWPQCLDQMTVKELDLNIKYSVVKNANFYLKAKSAFIELKFKGL 200

H.sapiens 201 LDRKGLWRSLNEMKRIFNFRRTPAAEHAFEHWQEDAFFASQFLNGLNPVL 250

|||.||||||.||:|:|||.||||||:.:|||||||||||||||||||||

D.spectabilis 201 LDRTGLWRSLREMRRMFNFHRTPAAEYIYEHWQEDAFFASQFLNGLNPVL 250

H.sapiens 251 IRRCHYLPKNFPVTDAMVASVLGPGTSLQAELEKGSLFLVDHGILSGIQT 300

|||||.||:||||||||||.||||.|:||||||:||||||||.|||.|.|

D.spectabilis 251 IRRCHSLPENFPVTDAMVAPVLGPETNLQAELERGSLFLVDHAILSDIHT 300

H.sapiens 301 NVINGKPQFSAAPMTLLYQSPGCGPLLPLAIQLSQTPGPNSPIFLPTDDK 350

|||||||||||||||||||.||.|||||||||||||||||||||||:|||

D.spectabilis 301 NVINGKPQFSAAPMTLLYQRPGHGPLLPLAIQLSQTPGPNSPIFLPSDDK 350

H.sapiens 351 WDWLLAKTWVRNAEFSFHEALTHLLHSHLLPEVFTLATLRQLPHCHPLFK 400

|||||||||||.||||.|||||||||:|||||||.:||||||||||||||

D.spectabilis 351 WDWLLAKTWVRYAEFSVHEALTHLLHAHLLPEVFAMATLRQLPHCHPLFK 400

H.sapiens 401 LLIPHTRYTLHINTLARELLIVPGQVVDRSTGIGIEGFSELIQRNMKQLN 450

|||||.|||||||||||||||.||||||||||:|||||||||:|||.||:

D.spectabilis 401 LLIPHIRYTLHINTLARELLIAPGQVVDRSTGLGIEGFSELIKRNMTQLS 450

H.sapiens 451 YSLLCLPEDIRTRGVEDIPGYYYRDDGMQIWGAVERFVSEIIGIYYPSDE 500

|:.|||||||:.|||||||||||||||||||.|||||||||||||||||.

D.spectabilis 451 YTDLCLPEDIQARGVEDIPGYYYRDDGMQIWRAVERFVSEIIGIYYPSDV 500

H.sapiens 501 SVQDDRELQAWVREIFSKGFLNQESSGIPSSLETREALVQYVTMVIFTCS 550

|||||:|||||||||||||||::||||:||||:||||||:|:||||||||

D.spectabilis 501 SVQDDQELQAWVREIFSKGFLDRESSGMPSSLDTREALVKYLTMVIFTCS 550

H.sapiens 551 AKHAAVSAGQFDSCAWMPNLPPSMQLPPPTSKGLATCEGFIATLPPVNAT 600

|:||||||||||||.||||||.:||||||||||.|..||||||||.||.|

D.spectabilis 551 ARHAAVSAGQFDSCVWMPNLPSTMQLPPPTSKGKARPEGFIATLPSVNVT 600

H.sapiens 601 CDVILALWLLSKEPGDQRPLGTYPDEHFTEEAPRRSIATFQSRLAQISRG 650

||||:.||:|||||||:|||||||||||.||||::||||||:|||||||.

D.spectabilis 601 CDVIITLWMLSKEPGDRRPLGTYPDEHFIEEAPKQSIATFQNRLAQISRA 650

H.sapiens 651 IQERNQGLVLPYTYLDPPLIENSVSI 676

|:||||.|.|||||||||||||||||

D.spectabilis 651 IRERNQTLPLPYTYLDPPLIENSVSI 676

North American beaver (*Castor canadensis*)

H.sapiens 1 MAEFRVRVSTGEAFGAGTWDKVSVSIVGTRGESPPLPLDNLGKEFTAGAE 50

||:||||||||||.|||||||||||||||:||||.||||:||||||||||

C.canadensis 1 MAKFRVRVSTGEACGAGTWDKVSVSIVGTKGESPLLPLDHLGKEFTAGAE 50

H.sapiens 51 EDFQVTLPEDVGRVLLLRVHKAPPVL-PLLGPLAPDAWFCRWFQLTPPRG 99

|||:||||:|||:||||||.||||.| |.||..||||||||||||.||||

C.canadensis 51 EDFEVTLPQDVGQVLLLRVRKAPPDLPPRLGLRAPDAWFCRWFQLVPPRG 100

H.sapiens 100 GHLLFPCYQWLEGAGTLVLQEGTAKVSWADHHPVLQQQRQEELQARQEMY 149

..|.|||||||||||.|||:||.||||||||||.|.|||||||||:||.|

C.canadensis 101 TPLHFPCYQWLEGAGDLVLREGAAKVSWADHHPTLLQQRQEELQAKQEKY 150

H.sapiens 150 QWKAYNPGWPHCLDEKTVEDLELNIKYSTAKNANFYLQAGSAFAEMKIKG 199

:||.|..|||||||:.||:||:||||||..|||.|:|:..|||.|:|:||

C.canadensis 151 KWKTYIQGWPHCLDQVTVKDLDLNIKYSAVKNAKFHLKVRSAFIELKLKG 200

H.sapiens 200 LLDRKGLWRSLNEMKRIFNFRRTPAAEHAFEHWQEDAFFASQFLNGLNPV 249

|||||||||||.||:|:|||.||||:::.|:|||||||||||||||||||

C.canadensis 201 LLDRKGLWRSLREMRRMFNFHRTPASDYVFKHWQEDAFFASQFLNGLNPV 250

H.sapiens 250 LIRRCHYLPKNFPVTDAMVASVLGPGTSLQAELEKGSLFLVDHGILSGIQ 299

:|.||..|||||||||||||.|||||||||||||:||||||||||||.|.

C.canadensis 251 MIHRCRSLPKNFPVTDAMVAPVLGPGTSLQAELERGSLFLVDHGILSDIH 300

H.sapiens 300 TNVINGKPQFSAAPMTLLYQSPGCGPLLPLAIQLSQTPGPNSPIFLPTDD 349

||:|||:|||||||||||||.||.|||||||||||||||||||||||:||

C.canadensis 301 TNIINGRPQFSAAPMTLLYQRPGRGPLLPLAIQLSQTPGPNSPIFLPSDD 350

H.sapiens 350 KWDWLLAKTWVRNAEFSFHEALTHLLHSHLLPEVFTLATLRQLPHCHPLF 399

|||||||||||||||||.|||||||||:||||||||||||||||||||||

C.canadensis 351 KWDWLLAKTWVRNAEFSVHEALTHLLHAHLLPEVFTLATLRQLPHCHPLF 400

H.sapiens 400 KLLIPHTRYTLHINTLARELLIVPGQVVDRSTGIGIEGFSELIQRNMKQL 449

||||||.|||||||||||||||.||||||||||||||||||||:||||||

C.canadensis 401 KLLIPHMRYTLHINTLARELLIAPGQVVDRSTGIGIEGFSELIKRNMKQL 450

H.sapiens 450 NYSLLCLPEDIRTRGVEDIPGYYYRDDGMQIWGAVERFVSEIIGIYYPSD 499

|||.||.||||:.||||||||||:|||||:||.||||||||::.|||||:

C.canadensis 451 NYSNLCFPEDIQARGVEDIPGYYFRDDGMKIWEAVERFVSEMVSIYYPSN 500

H.sapiens 500 ESVQDDRELQAWVREIFSKGFLNQESSGIPSSLETREALVQYVTMVIFTC 549

.|||:|:||||||:||||.|||.|||||:||||:|::||||:||||||||

C.canadensis 501 VSVQNDQELQAWVKEIFSNGFLGQESSGMPSSLDTQKALVQFVTMVIFTC 550

H.sapiens 550 SAKHAAVSAGQFDSCAWMPNLPPSMQLPPPTSKGLATCEGFIATLPPVNA 599

||||.|||..|||||.|||||||:|||||||:||.|..||||||||||||

C.canadensis 551 SAKHTAVSLSQFDSCVWMPNLPPTMQLPPPTTKGQARPEGFIATLPPVNA 600

H.sapiens 600 TCDVILALWLLSKEPGDQRPLGTYPDEHFTEEAPRRSIATFQSRLAQISR 649

|||||:|||||||||||:||||||||||||||||::|||:||:.||||||

C.canadensis 601 TCDVIIALWLLSKEPGDRRPLGTYPDEHFTEEAPKQSIASFQNHLAQISR 650

H.sapiens 650 GIQERNQGLVLPYTYLDPPLIENSVSI 676

.|:||||.|.|||||||||||||||||

C.canadensis 651 DIEERNQILALPYTYLDPPLIENSVSI 677

Golden hamster (*Mesocricetus auratus*)

H.sapiens 1 MAEFRVRVSTGEAFGAGTWDKVSVSIVGTRGESPPLPLDNLGKEFTAGAE 50

||.|||||:||||.|||||||||||||||:||||.:|||.||||||||||

M.auratus 1 MATFRVRVATGEACGAGTWDKVSVSIVGTQGESPLVPLDRLGKEFTAGAE 50

H.sapiens 51 EDFQVTLPEDVGRVLLLRVHKAPPVLPL-LGPLAPDAWFCRWFQLTPPRG 99

|||:||||:|||.||:||||||||.||| ||.|.|||||||||||....|

M.auratus 51 EDFEVTLPQDVGAVLMLRVHKAPPELPLSLGSLPPDAWFCRWFQLEWLPG 100

H.sapiens 100 GHLLFPCYQWLEGAGTLVLQEGTAKVSWADHHPVLQQQRQEELQARQEMY 149

..|.||||||||.||.|||::|.|||||.|.||.||.||||||:||:|||

M.auratus 101 AALRFPCYQWLEAAGDLVLRQGAAKVSWEDQHPKLQHQRQEELKARKEMY 150

H.sapiens 150 QWKAYNPGWPHCLDEKTVEDLELNIKYSTAKNANFYLQAGSAFAEMKIKG 199

.||.:..|||.|||..||:||:||||||..|||.|||:..||..|:||||

M.auratus 151 SWKTHIEGWPRCLDHMTVKDLDLNIKYSAVKNARFYLKVQSAVTELKIKG 200

H.sapiens 200 LLDRKGLWRSLNEMKRIFNFRRTPAAEHAFEHWQEDAFFASQFLNGLNPV 249

.|.|.||||||.||:|:|||.:|||||:.|||||||||||||||||||||

M.auratus 201 FLSRTGLWRSLREMRRMFNFHKTPAAEYVFEHWQEDAFFASQFLNGLNPV 250

H.sapiens 250 LIRRCHYLPKNFPVTDAMVASVLGPGTSLQAELEKGSLFLVDHGILSGIQ 299

||.||..|||||||||.|||.|||||||||||||||||||||||||||:|

M.auratus 251 LIHRCRSLPKNFPVTDDMVAPVLGPGTSLQAELEKGSLFLVDHGILSGVQ 300

H.sapiens 300 TNVINGKPQFSAAPMTLLYQSPGCGPLLPLAIQLSQTPGPNSPIFLPTDD 349

||||||||||||||||||||..|.||||||||||.|||||::|||||:||

M.auratus 301 TNVINGKPQFSAAPMTLLYQCSGSGPLLPLAIQLKQTPGPDNPIFLPSDD 350

H.sapiens 350 KWDWLLAKTWVRNAEFSFHEALTHLLHSHLLPEVFTLATLRQLPHCHPLF 399

|||||||||||||||||.|||:|||||:|||||||.||.||.||.|||||

M.auratus 351 KWDWLLAKTWVRNAEFSVHEAVTHLLHAHLLPEVFALAMLRHLPRCHPLF 400

H.sapiens 400 KLLIPHTRYTLHINTLARELLIVPGQVVDRSTGIGIEGFSELIQRNMKQL 449

||:|||.|||||||||||||||.||||||||||:||.||||||:|||:||

M.auratus 401 KLMIPHIRYTLHINTLARELLIAPGQVVDRSTGLGIGGFSELIKRNMEQL 450

H.sapiens 450 NYSLLCLPEDIRTRGVEDIPGYYYRDDGMQIWGAVERFVSEIIGIYYPSD 499

:||:||||||||.|.||||||||||||||:||||::.|||||:.||||||

M.auratus 451 SYSVLCLPEDIRARDVEDIPGYYYRDDGMRIWGAIKSFVSEIVSIYYPSD 500

H.sapiens 500 ESVQDDRELQAWVREIFSKGFLNQESSGIPSSLETREALVQYVTMVIFTC 549

.||.:|:|||||||||||:|||::||||:||||:|:|||:||||||||||

M.auratus 501 VSVGEDQELQAWVREIFSEGFLSRESSGMPSSLDTQEALIQYVTMVIFTC 550

H.sapiens 550 SAKHAAVSAGQFDSCAWMPNLPPSMQLPPPTSKGLATCEGFIATLPPVNA 599

|||||||||||||||.|||||||:||||||||||....||||||||||||

M.auratus 551 SAKHAAVSAGQFDSCVWMPNLPPTMQLPPPTSKGQVRPEGFIATLPPVNA 600

H.sapiens 600 TCDVILALWLLSKEPGDQRPLGTYPDEHFTEEAPRRSIATFQSRLAQISR 649

|||||:|||:|||||||:||||.||||||||:|||||::.||.:|.||||

M.auratus 601 TCDVIIALWMLSKEPGDRRPLGHYPDEHFTEDAPRRSMSAFQRQLVQISR 650

H.sapiens 650 GIQERNQGLVLPYTYLDPPLIENSVSI 676

.|.|||:.|.|||.|||||.|||||||

M.auratus 651 DIGERNRSLALPYIYLDPPHIENSVSI 677

Chinese hamster (*Cricetulus griseus*)

H.sapiens 1 MAEFRVRVSTGEAFGAGTWDKVSVSIVGTRGESPPLPLDNLGKEFTAGAE 50

||:|||||:||||.|||||||||||||||:||||.:|||.||||||||||

C.griseus 1 MAKFRVRVATGEACGAGTWDKVSVSIVGTQGESPLVPLDRLGKEFTAGAE 50

H.sapiens 51 EDFQVTLPEDVGRVLLLRVHKAPPVLPL-LGPLAPDAWFCRWFQLTPPRG 99

|||:||||:|||.||:||||||||.||| ||.|.|||||||||||....|

C.griseus 51 EDFEVTLPQDVGAVLMLRVHKAPPELPLSLGSLHPDAWFCRWFQLEWLPG 100

H.sapiens 100 GHLLFPCYQWLEGAGTLVLQEGTAKVSWADHHPVLQQQRQEELQARQEMY 149

..|.|||||||||||.|||::|.|||||.||||.||.||||||:||:|||

C.griseus 101 AALRFPCYQWLEGAGDLVLRQGAAKVSWEDHHPKLQHQRQEELKARKEMY 150

H.sapiens 150 QWKAYNPGWPHCLDEKTVEDLELNIKYSTAKNANFYLQAGSAFAEMKIKG 199

.||.|..|||||||..||:||:||||||..|||.|:.:..||..|:||||

C.griseus 151 SWKTYIEGWPHCLDHMTVKDLDLNIKYSAIKNARFFFKVQSAVTELKIKG 200

H.sapiens 200 LLDRKGLWRSLNEMKRIFNFRRTPAAEHAFEHWQEDAFFASQFLNGLNPV 249

.|.|.||||||.||:|:|||.:|||||:.|||||||||||||||||||||

C.griseus 201 FLSRTGLWRSLREMRRMFNFHKTPAAEYVFEHWQEDAFFASQFLNGLNPV 250

H.sapiens 250 LIRRCHYLPKNFPVTDAMVASVLGPGTSLQAELEKGSLFLVDHGILSGIQ 299

||||||.|||||||||.|||.|||||||||||||||||||||||||||||

C.griseus 251 LIRRCHSLPKNFPVTDDMVAPVLGPGTSLQAELEKGSLFLVDHGILSGIQ 300

H.sapiens 300 TNVINGKPQFSAAPMTLLYQSPGCGPLLPLAIQLSQTPGPNSPIFLPTDD 349

||||||||||||||||||||..|.||||||||||.|||||::|||||:||

C.griseus 301 TNVINGKPQFSAAPMTLLYQCSGSGPLLPLAIQLKQTPGPDNPIFLPSDD 350

H.sapiens 350 KWDWLLAKTWVRNAEFSFHEALTHLLHSHLLPEVFTLATLRQLPHCHPLF 399

|||||||||||||||||.|||:|||||:|||||||.|||||.||||||||

C.griseus 351 KWDWLLAKTWVRNAEFSVHEAVTHLLHAHLLPEVFALATLRHLPHCHPLF 400

H.sapiens 400 KLLIPHTRYTLHINTLARELLIVPGQVVDRSTGIGIEGFSELIQRNMKQL 449

||||||.|||||||||||||||.||||||||||:||.||||||:|||:||

C.griseus 401 KLLIPHIRYTLHINTLARELLIAPGQVVDRSTGLGIGGFSELIKRNMEQL 450

H.sapiens 450 NYSLLCLPEDIRTRGVEDIPGYYYRDDGMQIWGAVERFVSEIIGIYYPSD 499

:||:||||||||.|.|.|||||||||||||||||::.|||||:.||||||

C.griseus 451 SYSVLCLPEDIRARDVADIPGYYYRDDGMQIWGAIKSFVSEIVSIYYPSD 500

H.sapiens 500 ESVQDDRELQAWVREIFSKGFLNQESSGIPSSLETREALVQYVTMVIFTC 549

.||::|:|||||||||||:|||::||||:||||:|:||||||||||||||

C.griseus 501 VSVREDQELQAWVREIFSEGFLSRESSGMPSSLDTQEALVQYVTMVIFTC 550

H.sapiens 550 SAKHAAVSAGQFDSCAWMPNLPPSMQLPPPTSKGLATCEGFIATLPPVNA 599

|||||||||||||:|.|||||||:||||||||||....||||||||||||

C.griseus 551 SAKHAAVSAGQFDACVWMPNLPPTMQLPPPTSKGQVRPEGFIATLPPVNA 600

H.sapiens 600 TCDVILALWLLSKEPGDQRPLGTYPDEHFTEEAPRRSIATFQSRLAQISR 649

|||||:|||:|||||||:||||.||||||||:|||||:|.||.:|.||||

C.griseus 601 TCDVIIALWMLSKEPGDRRPLGHYPDEHFTEDAPRRSMAAFQRQLVQISR 650

H.sapiens 650 GIQERNQGLVLPYTYLDPPLIENSVSI 676

.|.|||:.|.|||.|||||||||||||

C.griseus 651 DIGERNRSLALPYIYLDPPLIENSVSI 677

Lesser Egyptian jerboa (*Jaculus jaculus*)

H.sapiens 1 ----------------------------------------MAEFRVRVST 10

:|..||||||

J.jaculus 1 MVDRLQRAQAGAPSEHDPQSTAVSTDQVPPAKRLRILASTVAMIRVRVST 50

H.sapiens 11 GEAFGAGTWDKVSVSIVGTRGESPPLPLDNLGKEFTAGAEEDFQVTLPED 60

|||||:|||||||||||||:||||.||||.|||.||||:||.|:||:|:|

J.jaculus 51 GEAFGSGTWDKVSVSIVGTQGESPLLPLDRLGKTFTAGSEEVFEVTIPKD 100

H.sapiens 61 VGRVLLLRVHKAPPVLPLLGPLAPDAWFCRWFQLTPPRGGHLLFPCYQWL 110

||.||||||||.||.|| .::||||:||||||....|..|.|||||||

J.jaculus 101 VGPVLLLRVHKVPPRLP---GVSPDAWYCRWFQLEMLPGAPLRFPCYQWL 147

H.sapiens 111 EGAGTLVLQEGTAKVSWADHHPVLQQQRQEELQARQEMYQWKAYNPGWPH 160

|.||:|||::|.|||.|||:|..||:|||||||||:|||.||.|..||||

J.jaculus 148 EAAGSLVLRDGAAKVCWADNHLRLQRQRQEELQARREMYCWKTYVQGWPH 197

H.sapiens 161 CLDEKTVEDLELNIKYSTAKNANFYLQAGSAFAEMKIKGLLDRKGLWRSL 210

||||.:|.||:.||||||.|.|.|.|:..|||.|:||||||||.|||:||

J.jaculus 198 CLDEVSVSDLDRNIKYSTVKAAKFKLKTSSAFTELKIKGLLDRTGLWKSL 247

H.sapiens 211 NEMKRIFNFRRTPAAEHAFEHWQEDAFFASQFLNGLNPVLIRRCHYLPKN 260

.||||:.||..||.||:.|:|||||:|||||||||||||:|||||.||||

J.jaculus 248 QEMKRMLNFHSTPVAEYVFQHWQEDSFFASQFLNGLNPVMIRRCHSLPKN 297

H.sapiens 261 FPVTDAMVASVLGPGTSLQAELEKGSLFLVDHGILSGIQTNVINGKPQFS 310

|||||.|||.|||||||||||||||||||||||||||::||||||:||||

J.jaculus 298 FPVTDDMVAPVLGPGTSLQAELEKGSLFLVDHGILSGVRTNVINGRPQFS 347

H.sapiens 311 AAPMTLLYQSPGCGPLLPLAIQLSQTPGPNSPIFLPTDDKWDWLLAKTWV 360

|||||||||.|..||||||||||||:||||||||||.||.||||||||||

J.jaculus 348 AAPMTLLYQCPDHGPLLPLAIQLSQSPGPNSPIFLPGDDTWDWLLAKTWV 397

H.sapiens 361 RNAEFSFHEALTHLLHSHLLPEVFTLATLRQLPHCHPLFKLLIPHTRYTL 410

||||||.||||||||.:|||||||.||.|||||.|||||||||||.||||

J.jaculus 398 RNAEFSVHEALTHLLRAHLLPEVFALALLRQLPRCHPLFKLLIPHIRYTL 447

H.sapiens 411 HINTLARELLIVPGQVVDRSTGIGIEGFSELIQRNMKQLNYSLLCLPEDI 460

||||||||.|:.||:|||||||:|||||||||||||:||||:.|||||||

J.jaculus 448 HINTLAREQLVAPGKVVDRSTGLGIEGFSELIQRNMEQLNYTDLCLPEDI 497

H.sapiens 461 RTRGVEDIPGYYYRDDGMQIWGAVERFVSEIIGIYYPSDESVQDDRELQA 510

:.||||||||||||||||||||||:.|||:|:|||||||.:||||:||||

J.jaculus 498 KARGVEDIPGYYYRDDGMQIWGAVKSFVSDIVGIYYPSDAAVQDDQELQA 547

H.sapiens 511 WVREIFSKGFLNQESSGIPSSLETREALVQYVTMVIFTCSAKHAAVSAGQ 560

||:|||.:|||.::||||||||:|:|||||||||||||||||||||||.|

J.jaculus 548 WVKEIFVEGFLGRKSSGIPSSLDTQEALVQYVTMVIFTCSAKHAAVSASQ 597

H.sapiens 561 FDSCAWMPNLPPSMQLPPPTSKGLATCEGFIATLPPVNATCDVILALWLL 610

||||.|||||||||||||||.||.|..|.|||||||||||||||:|||||

J.jaculus 598 FDSCVWMPNLPPSMQLPPPTFKGQAHAESFIATLPPVNATCDVIIALWLL 647

H.sapiens 611 SKEPGDQRPLGTYPDEHFTEEAPRRSIATFQSRLAQISRGIQERNQGLVL 660

||||||:|||||||||||||||||:||..||:.||||||.|:||||||.|

J.jaculus 648 SKEPGDRRPLGTYPDEHFTEEAPRQSITAFQNHLAQISRSIKERNQGLPL 697

H.sapiens 661 PYTYLDPPLIENSVSI 676

||||||||.|||||||

J.jaculus 698 PYTYLDPPTIENSVSI 713

Deer mouse (*Peromyscus maniculatus bairdii*)

H.sapiens 1 MAEFRVRVSTGEAFGAGTWDKVSVSIVGTRGESPPLPLDNLGKEFTAGAE 50

||:.|||||||||.|||||||||||||||:||||.:|||.||||||||||

P.maniculatus ba. 1 MAKVRVRVSTGEACGAGTWDKVSVSIVGTQGESPLVPLDRLGKEFTAGAE 50

H.sapiens 51 EDFQVTLPEDVGRVLLLRVHKAPPVLPL-LGPLAPDAWFCRWFQLTPPRG 99

|||:||||:|||.||:||||||||.||| ||...|||||||||||....|

P.maniculatus ba. 51 EDFEVTLPQDVGAVLMLRVHKAPPELPLPLGARPPDAWFCRWFQLEWGPG 100

H.sapiens 100 GHLLFPCYQWLEGAGTLVLQEGTAKVSWADHHPVLQQQRQEELQARQEMY 149

..|.|||||||||||.||||||.||||..||||.||.||.|||:||:|||

P.maniculatus ba.101 ATLHFPCYQWLEGAGDLVLQEGAAKVSSEDHHPKLQLQRHEELKARKEMY 150

H.sapiens 150 QWKAYNPGWPHCLDEKTVEDLELNIKYSTAKNANFYLQAGSAFAEMKIKG 199

.||.|..|||||||:.||:||:||:|||..|||.|||:..|||.|:||||

P.maniculatus ba.151 SWKTYIEGWPHCLDQVTVKDLDLNVKYSEVKNARFYLKVYSAFTELKIKG 200

H.sapiens 200 LLDRKGLWRSLNEMKRIFNFRRTPAAEHAFEHWQEDAFFASQFLNGLNPV 249

.|:|.||||||.||:|:|:|.:|||||:..||||||||||||||||||||

P.maniculatus ba.201 FLNRTGLWRSLREMRRMFHFHKTPAAEYVLEHWQEDAFFASQFLNGLNPV 250

H.sapiens 250 LIRRCHYLPKNFPVTDAMVASVLGPGTSLQAELEKGSLFLVDHGILSGIQ 299

||||||.|||||||||.|||.|||||||||||||||||||||||||||:|

P.maniculatus ba.251 LIRRCHSLPKNFPVTDEMVAPVLGPGTSLQAELEKGSLFLVDHGILSGVQ 300

H.sapiens 300 TNVINGKPQFSAAPMTLLYQSPGCGPLLPLAIQLSQTPGPNSPIFLPTDD 349

|||||||||||||||||||||...||||||||||.|||||::|||||:|.

P.maniculatus ba.301 TNVINGKPQFSAAPMTLLYQSSEAGPLLPLAIQLKQTPGPDNPIFLPSDG 350

H.sapiens 350 KWDWLLAKTWVRNAEFSFHEALTHLLHSHLLPEVFTLATLRQLPHCHPLF 399

:||||||||||||||||.|||||||||:|||||||.||||||||.|||||

P.maniculatus ba.351 RWDWLLAKTWVRNAEFSVHEALTHLLHAHLLPEVFALATLRQLPRCHPLF 400

H.sapiens 400 KLLIPHTRYTLHINTLARELLIVPGQVVDRSTGIGIEGFSELIQRNMKQL 449

||||||.|||||||||||||||.||:|||||||:|..||||||:|||:||

P.maniculatus ba.401 KLLIPHIRYTLHINTLARELLIAPGKVVDRSTGLGTGGFSELIKRNMEQL 450

H.sapiens 450 NYSLLCLPEDIRTRGVEDIPGYYYRDDGMQIWGAVERFVSEIIGIYYPSD 499

:||.||||||||.|.|.|||||||||||||||||::.|||||:.||||||

P.maniculatus ba.451 DYSALCLPEDIRARDVGDIPGYYYRDDGMQIWGAIKSFVSEIVSIYYPSD 500

H.sapiens 500 ESVQDDRELQAWVREIFSKGFLNQESSGIPSSLETREALVQYVTMVIFTC 549

.|:|:|.||||||:||||:|||::||||:||.|:||||||||||||||||

P.maniculatus ba.501 VSIQEDGELQAWVKEIFSEGFLSRESSGVPSLLDTREALVQYVTMVIFTC 550

H.sapiens 550 SAKHAAVSAGQFDSCAWMPNLPPSMQLPPPTSKGLATCEGFIATLPPVNA 599

|||||||||||||||.|||||||:||||||||||.|..||||||||||||

P.maniculatus ba.551 SAKHAAVSAGQFDSCVWMPNLPPTMQLPPPTSKGQARPEGFIATLPPVNA 600

H.sapiens 600 TCDVILALWLLSKEPGDQRPLGTYPDEHFTEEAPRRSIATFQSRLAQISR 649

|||||:|||:|||||||:||||.||||||||:||||||..||.||.||||

P.maniculatus ba.601 TCDVIIALWMLSKEPGDRRPLGHYPDEHFTEDAPRRSITAFQRRLVQISR 650

H.sapiens 650 GIQERNQGLVLPYTYLDPPLIENSVSI 676

.|.|||:.|.|||.|:|||||||||||

P.maniculatus ba.651 EIGERNRSLALPYIYMDPPLIENSVSI 677

Prairie vole (*Microtus ochrogaster*)

H.sapiens 1 MAEFRVRVSTGEAFGAGTWDKVSVSIVGTRGESPPLPLDNLGKEFTAGAE 50

||:||||||||||.|||||||||||||||:||||.:|||.||||||||||

M.ochrogaster 1 MAKFRVRVSTGEACGAGTWDKVSVSIVGTQGESPLVPLDRLGKEFTAGAE 50

H.sapiens 51 EDFQVTLPEDVGRVLLLRVHKAPPVLPL-LGPLAPDAWFCRWFQLTPPRG 99

|||:||||::||.||:||||||||.||| ||.|.|||||||||||....|

M.ochrogaster 51 EDFEVTLPQEVGAVLMLRVHKAPPKLPLRLGSLQPDAWFCRWFQLEWLPG 100

H.sapiens 100 GHLLFPCYQWLEGAGTLVLQEGTAKVSWADHHPVLQQQRQEELQARQEMY 149

..|.|||||||||.|.|||:||.|||...||||.||.||||||:||:|||

M.ochrogaster 101 AALRFPCYQWLEGEGDLVLREGAAKVCQEDHHPALQHQRQEELKARKEMY 150

H.sapiens 150 QWKAYNPGWPHCLDEKTVEDLELNIKYSTAKNANFYLQAGSAFAEMKIKG 199

.||.|..|||.|||..||:||:||||||..|||.||.:..|||.|:||||

M.ochrogaster 151 SWKTYADGWPRCLDHATVKDLDLNIKYSAVKNAKFYFKVHSAFTELKIKG 200

H.sapiens 200 LLDRKGLWRSLNEMKRIFNFRRTPAAEHAFEHWQEDAFFASQFLNGLNPV 249

.|||.||||||.||:|:|||.:|||||:.|.|||||||||||||||||||

M.ochrogaster 201 FLDRTGLWRSLREMRRMFNFHKTPAAEYVFAHWQEDAFFASQFLNGLNPV 250

H.sapiens 250 LIRRCHYLPKNFPVTDAMVASVLGPGTSLQAELEKGSLFLVDHGILSGIQ 299

||||||.|||||||||.|||.|||||||||||||||||||||||||||:|

M.ochrogaster 251 LIRRCHSLPKNFPVTDEMVAPVLGPGTSLQAELEKGSLFLVDHGILSGVQ 300

H.sapiens 300 TNVINGKPQFSAAPMTLLYQSPGCGPLLPLAIQLSQTPGPNSPIFLPTDD 349

||||||||||||||||||||..|.||||||||||.|||||::|||||:||

M.ochrogaster 301 TNVINGKPQFSAAPMTLLYQCSGSGPLLPLAIQLKQTPGPDNPIFLPSDD 350

H.sapiens 350 KWDWLLAKTWVRNAEFSFHEALTHLLHSHLLPEVFTLATLRQLPHCHPLF 399

|||||||||||||||||.|||||||||:|||||||.||||||||||||||

M.ochrogaster 351 KWDWLLAKTWVRNAEFSVHEALTHLLHAHLLPEVFALATLRQLPHCHPLF 400

H.sapiens 400 KLLIPHTRYTLHINTLARELLIVPGQVVDRSTGIGIEGFSELIQRNMKQL 449

||||||.:||||||||||:|||.||||||||||:||.||||||:|||:||

M.ochrogaster 401 KLLIPHIQYTLHINTLARKLLIAPGQVVDRSTGLGIGGFSELIKRNMEQL 450

H.sapiens 450 NYSLLCLPEDIRTRGVEDIPGYYYRDDGMQIWGAVERFVSEIIGIYYPSD 499

|||:||.|||||.|.|||||||||||||||||||::.|||||:.||||||

M.ochrogaster 451 NYSVLCFPEDIRAREVEDIPGYYYRDDGMQIWGAIKSFVSEIVSIYYPSD 500

H.sapiens 500 ESVQDDRELQAWVREIFSKGFLNQESSGIPSSLETREALVQYVTMVIFTC 549

.||::|:|||||||||||:|||::||||:||.|:|:||||||||||||||

M.ochrogaster 501 VSVREDQELQAWVREIFSEGFLSRESSGMPSLLDTQEALVQYVTMVIFTC 550

H.sapiens 550 SAKHAAVSAGQFDSCAWMPNLPPSMQLPPPTSKGLATCEGFIATLPPVNA 599

||||.||||||||||.|||||||:||||||||||.|..|.||||||||||

M.ochrogaster 551 SAKHTAVSAGQFDSCVWMPNLPPTMQLPPPTSKGQARPESFIATLPPVNA 600

H.sapiens 600 TCDVILALWLLSKEPGDQRPLGTYPDEHFTEEAPRRSIATFQSRLAQISR 649

|||||:|||:|||||||:||||.||||||||:|||||:|.||.:|.|||:

M.ochrogaster 601 TCDVIIALWMLSKEPGDRRPLGHYPDEHFTEDAPRRSMAAFQRQLIQISK 650

H.sapiens 650 GIQERNQGLVLPYTYLDPPLIENSVSI 676

||.|||:||.|||||||||||||||||

M.ochrogaster 651 GIAERNRGLALPYTYLDPPLIENSVSI 677

Creeping vole (*Microtus oregoni*)

H.sapiens 1 MAEFRVRVSTGEAFGAGTWDKVSVSIVGTRGESPPLPLDNLGKEFTAGAE 50

|.:||||||||||.|||||||||||||||:||||.:|||.||||||||||

M.oregoni 1 MTKFRVRVSTGEACGAGTWDKVSVSIVGTQGESPLVPLDRLGKEFTAGAE 50

H.sapiens 51 EDFQVTLPEDVGRVLLLRVHKAPPVLPL-LGPLAPDAWFCRWFQLTPPRG 99

|||:||||:|||.||:||||||||.||| ||.|.|||||||||||....|

M.oregoni 51 EDFEVTLPQDVGAVLMLRVHKAPPKLPLPLGSLQPDAWFCRWFQLEWLPG 100

H.sapiens 100 GHLLFPCYQWLEGAGTLVLQEGTAKVSWADHHPVLQQQRQEELQARQEMY 149

..|.|||||||||.|.|||:||.||||..||||.||.||||||:||:|||

M.oregoni 101 AALRFPCYQWLEGEGDLVLREGAAKVSQEDHHPALQHQRQEELKARKEMY 150

H.sapiens 150 QWKAYNPGWPHCLDEKTVEDLELNIKYSTAKNANFYLQAGSAFAEMKIKG 199

.||.|..|||.|||..||:||:||||||..|||.|:.:..|||.|:||||

M.oregoni 151 SWKTYADGWPRCLDHATVKDLDLNIKYSAVKNAKFFFKVHSAFTELKIKG 200

H.sapiens 200 LLDRKGLWRSLNEMKRIFNFRRTPAAEHAFEHWQEDAFFASQFLNGLNPV 249

.|||.||||||.||:|:|||.:|||||:.|.|||||||||||||||||||

M.oregoni 201 FLDRIGLWRSLREMRRMFNFHKTPAAEYVFAHWQEDAFFASQFLNGLNPV 250

H.sapiens 250 LIRRCHYLPKNFPVTDAMVASVLGPGTSLQAELEKGSLFLVDHGILSGIQ 299

||||||.|||||||||.|||.||||||||||||||||||||||.||||:|

M.oregoni 251 LIRRCHSLPKNFPVTDEMVAPVLGPGTSLQAELEKGSLFLVDHSILSGVQ 300

H.sapiens 300 TNVINGKPQFSAAPMTLLYQSPGCGPLLPLAIQLSQTPGPNSPIFLPTDD 349

||||||||||||||||||:|..|.||||||||||.|||||::|||||:||

M.oregoni 301 TNVINGKPQFSAAPMTLLHQRSGSGPLLPLAIQLKQTPGPDNPIFLPSDD 350

H.sapiens 350 KWDWLLAKTWVRNAEFSFHEALTHLLHSHLLPEVFTLATLRQLPHCHPLF 399

|||||||||||||||||.|||||||||:|||||||.||||||||||||||

M.oregoni 351 KWDWLLAKTWVRNAEFSVHEALTHLLHAHLLPEVFALATLRQLPHCHPLF 400

H.sapiens 400 KLLIPHTRYTLHINTLARELLIVPGQVVDRSTGIGIEGFSELIQRNMKQL 449

||||||.|||||||||||:|||.||||||||||:||.||||||:|||:||

M.oregoni 401 KLLIPHIRYTLHINTLARKLLIAPGQVVDRSTGLGIGGFSELIKRNMEQL 450

H.sapiens 450 NYSLLCLPEDIRTRGVEDIPGYYYRDDGMQIWGAVERFVSEIIGIYYPSD 499

|||.||.||||..|.|||||||||||||||||||::.|||||:.||||||

M.oregoni 451 NYSALCFPEDILARDVEDIPGYYYRDDGMQIWGAIKSFVSEIVSIYYPSD 500

H.sapiens 500 ESVQDDRELQAWVREIFSKGFLNQESSGIPSSLETREALVQYVTMVIFTC 549

.||::|:|||||||||||:|||::||||:||.|:|:||||||||||||||

M.oregoni 501 VSVREDQELQAWVREIFSEGFLSRESSGMPSLLDTQEALVQYVTMVIFTC 550

H.sapiens 550 SAKHAAVSAGQFDSCAWMPNLPPSMQLPPPTSKGLATCEGFIATLPPVNA 599

||||.||||||||||.|||||||:||||||||||.|..|.||||||||||

M.oregoni 551 SAKHTAVSAGQFDSCVWMPNLPPTMQLPPPTSKGQARPESFIATLPPVNA 600

H.sapiens 600 TCDVILALWLLSKEPGDQRPLGTYPDEHFTEEAPRRSIATFQSRLAQISR 649

|||||:|||:|||||||:||||.||||||||:|||||:|.||.:|.|||:

M.oregoni 601 TCDVIIALWMLSKEPGDRRPLGHYPDEHFTEDAPRRSMAAFQRQLIQISK 650

H.sapiens 650 GIQERNQGLVLPYTYLDPPLIENSVSI 676

||.|||:||.|||||||||||||||||

M.oregoni 651 GIAERNRGLALPYTYLDPPLIENSVSI 677

European water vole (*Arvicola amphibius*)

H.sapiens 1 MAEFRVRVSTGEAFGAGTWDKVSVSIVGTRGESPPLPLDNLGKEFTAGAE 50

|.:||||||||||.|||||||||||||||:||||.:|||.||||||||||

A.amphibius 1 MTKFRVRVSTGEACGAGTWDKVSVSIVGTQGESPLVPLDRLGKEFTAGAE 50

H.sapiens 51 EDFQVTLPEDVGRVLLLRVHKAPPVLPL-LGPLAPDAWFCRWFQLTPPRG 99

|||:||||:|||.||:||||||||.||| ||.|.|||||||||||....|

A.amphibius 51 EDFEVTLPQDVGAVLMLRVHKAPPKLPLPLGSLRPDAWFCRWFQLEWLPG 100

H.sapiens 100 GHLLFPCYQWLEGAGTLVLQEGTAKVSWADHHPVLQQQRQEELQARQEMY 149

..|.|||||||||.|.|||:||.||||..||||.||.||||||:||:|||

A.amphibius 101 AALRFPCYQWLEGEGDLVLREGAAKVSQEDHHPALQHQRQEELKARKEMY 150

H.sapiens 150 QWKAYNPGWPHCLDEKTVEDLELNIKYSTAKNANFYLQAGSAFAEMKIKG 199

.||.|..|||.|||..||:||:||||||..|||.||.:..|||.|:||||

A.amphibius 151 SWKTYADGWPRCLDHATVKDLDLNIKYSAVKNAKFYFKVHSAFTELKIKG 200

H.sapiens 200 LLDRKGLWRSLNEMKRIFNFRRTPAAEHAFEHWQEDAFFASQFLNGLNPV 249

.|||.||||||.||:|:|||.:|||||:.|.|||||||||||||||||||

A.amphibius 201 FLDRTGLWRSLREMRRMFNFHKTPAAEYVFAHWQEDAFFASQFLNGLNPV 250

H.sapiens 250 LIRRCHYLPKNFPVTDAMVASVLGPGTSLQAELEKGSLFLVDHGILSGIQ 299

||||||.|||||||||.|||.|||||||||||||||||||||||||||:|

A.amphibius 251 LIRRCHSLPKNFPVTDEMVAPVLGPGTSLQAELEKGSLFLVDHGILSGVQ 300

H.sapiens 300 TNVINGKPQFSAAPMTLLYQSPGCGPLLPLAIQLSQTPGPNSPIFLPTDD 349

||||||||||||||||||||..|.||||||||||.|.|||::|||||:||

A.amphibius 301 TNVINGKPQFSAAPMTLLYQRSGSGPLLPLAIQLKQIPGPDNPIFLPSDD 350

H.sapiens 350 KWDWLLAKTWVRNAEFSFHEALTHLLHSHLLPEVFTLATLRQLPHCHPLF 399

|||||||||||||||||.|||||||||:||:||||.||||||||||||||

A.amphibius 351 KWDWLLAKTWVRNAEFSVHEALTHLLHAHLVPEVFALATLRQLPHCHPLF 400

H.sapiens 400 KLLIPHTRYTLHINTLARELLIVPGQVVDRSTGIGIEGFSELIQRNMKQL 449

||||||.|||||||||||:|||.||||||||||:|..||||||:|||:||

A.amphibius 401 KLLIPHIRYTLHINTLARKLLIAPGQVVDRSTGLGTGGFSELIKRNMEQL 450

H.sapiens 450 NYSLLCLPEDIRTRGVEDIPGYYYRDDGMQIWGAVERFVSEIIGIYYPSD 499

|||:||.|||||.|.|||||||||||||.|||||::.|||||:.||||||

A.amphibius 451 NYSVLCFPEDIRARDVEDIPGYYYRDDGTQIWGAIKSFVSEIVSIYYPSD 500

H.sapiens 500 ESVQDDRELQAWVREIFSKGFLNQESSGIPSSLETREALVQYVTMVIFTC 549

.||::|:|||||||||||:|||::||||:||.|||:||||||||||||||

A.amphibius 501 VSVREDQELQAWVREIFSEGFLSRESSGMPSLLETQEALVQYVTMVIFTC 550

H.sapiens 550 SAKHAAVSAGQFDSCAWMPNLPPSMQLPPPTSKGLATCEGFIATLPPVNA 599

||||.||||||||||.|||||||:||||||||||.|..|.||||||||||

A.amphibius 551 SAKHTAVSAGQFDSCVWMPNLPPTMQLPPPTSKGQAQPESFIATLPPVNA 600

H.sapiens 600 TCDVILALWLLSKEPGDQRPLGTYPDEHFTEEAPRRSIATFQSRLAQISR 649

|||||:|||:|||||||:||||.||||||||:|||||:|.||.:|.|||:

A.amphibius 601 TCDVIIALWMLSKEPGDRRPLGHYPDEHFTEDAPRRSMAAFQRQLIQISK 650

H.sapiens 650 GIQERNQGLVLPYTYLDPPLIENSVSI 676

||.|||:.|.|||||||||||||||||

A.amphibius 651 GIAERNRSLALPYTYLDPPLIENSVSI 677

Common degu (*Octodon degus*)

H.sapiens 1 MAEFRVRVSTGEAFGAGTWDKVSVSIVGTRGESPPLPLDNLGKEFTAGAE 50

||:||||||||||.|||||||||||||||:|||||||||:||||||||||

O.degus 1 MAKFRVRVSTGEACGAGTWDKVSVSIVGTQGESPPLPLDHLGKEFTAGAE 50

H.sapiens 51 EDFQVTLPEDVGRVLLLRVHKAPPVLPL-LGPLAPDAWFCRWFQLTPPRG 99

|||:||||||||||||||||||||.||. ||||||||||||||:|.||.|

O.degus 51 EDFEVTLPEDVGRVLLLRVHKAPPPLPRPLGPLAPDAWFCRWFRLEPPLG 100

H.sapiens 100 GHLLFPCYQWLEGAGTLVLQEGTAKVSWADHHPVLQQQRQEELQARQEMY 149

..|||||||||||||||||:||.|||||||..|||::||||||||:|:||

O.degus 101 SPLLFPCYQWLEGAGTLVLREGAAKVSWADDLPVLERQRQEELQAKQKMY 150

H.sapiens 150 QWKAYNPGWPHCLDEKTVEDLELNIKYSTAKNANFYLQAGSAFAEMKIKG 199

.||.|.|||||||||:||:||:||:||...|...|.|:.||..||:|:||

O.degus 151 WWKTYLPGWPHCLDEETVKDLDLNVKYPVVKKTKFSLKLGSGVAELKLKG 200

H.sapiens 200 LLDRKGLWRSLNEMKRIFNFRRTPAAEHAFEHWQEDAFFASQFLNGLNPV 249

||||.||||||.||||:.|||.:|..|:.::|||||:|||||||||||||

O.degus 201 LLDRTGLWRSLREMKRMLNFRSSPVGEYVYKHWQEDSFFASQFLNGLNPV 250

H.sapiens 250 LIRRCHYLPKNFPVTDAMVASVLGPGTSLQAELEKGSLFLVDHGILSGIQ 299

|||||..|||||||||||||.:||||.||||||||||||||||||||.||

O.degus 251 LIRRCRLLPKNFPVTDAMVAPMLGPGNSLQAELEKGSLFLVDHGILSSIQ 300

H.sapiens 300 TNVINGKPQFSAAPMTLLYQSPGCGPLLPLAIQLSQTPGPNSPIFLPTDD 349

||||||:|||||||||||||.|..|||||||||||||||||||||||:|:

O.degus 301 TNVINGRPQFSAAPMTLLYQHPRGGPLLPLAIQLSQTPGPNSPIFLPSDE 350

H.sapiens 350 KWDWLLAKTWVRNAEFSFHEALTHLLHSHLLPEVFTLATLRQLPHCHPLF 399

|||||||||||||||||.|||:|||||:|||||||:||||||||.|||||

O.degus 351 KWDWLLAKTWVRNAEFSVHEAITHLLHAHLLPEVFSLATLRQLPQCHPLF 400

H.sapiens 400 KLLIPHTRYTLHINTLARELLIVPGQVVDRSTGIGIEGFSELIQRNMKQL 449

|||||||||||:||||||||||.||:|||||||:||.||||||||||:||

O.degus 401 KLLIPHTRYTLYINTLARELLIAPGKVVDRSTGLGIGGFSELIQRNMEQL 450

H.sapiens 450 NYSLLCLPEDIRTRGVEDIPGYYYRDDGMQIWGAVERFVSEIIGIYYPSD 499

|||:||||||||.|||||||||||||||:::|.|||:||||||.||||||

O.degus 451 NYSVLCLPEDIRDRGVEDIPGYYYRDDGLRVWEAVEQFVSEIISIYYPSD 500

H.sapiens 500 ESVQDDRELQAWVREIFSKGFLNQESSGIPSSLETREALVQYVTMVIFTC 549

||||||:|||||||:|||.|||.:||||:||||:|||||::|.|||||||

O.degus 501 ESVQDDQELQAWVRDIFSNGFLGRESSGMPSSLDTREALIRYTTMVIFTC 550

H.sapiens 550 SAKHAAVSAGQFDSCAWMPNLPPSMQLPPPTSKGLATCEGFIATLPPVNA 599

||||.|||:||||||.||||||.:|||||||:||.|..|||:||||||||

O.degus 551 SAKHTAVSSGQFDSCVWMPNLPSTMQLPPPTAKGQARPEGFVATLPPVNA 600

H.sapiens 600 TCDVILALWLLSKEPGDQRPLGTYPDEHFTEEAPRRSIATFQSRLAQISR 649

|||:|:|||||||||||:||||.||||:|||||||||||||||.||||||

O.degus 601 TCDIIIALWLLSKEPGDRRPLGIYPDEYFTEEAPRRSIATFQSHLAQISR 650

H.sapiens 650 GIQERNQGLVLPYTYLDPPLIENSVSI 676

.|::|||.|.|||||||||||||||||

O.degus 651 DIRKRNQDLKLPYTYLDPPLIENSVSI 677

European rabbit (*Oryctolagus cuniculus*)

H.sapiens 1 MAEFRVRVSTGEAFGAGTWDKVSVSIVGTRGESPPLPLDNLGKEFTAGAE 50

||:|.|:||||:|||||||||||:|||||.|||||||||:.|||||||||

O.cuniculus 1 MAKFTVKVSTGKAFGAGTWDKVSISIVGTLGESPPLPLDHFGKEFTAGAE 50

H.sapiens 51 EDFQVTLPEDVGRVLLLRVHKAPPVL--PLLGPLAPDAWFCRWFQLTPPR 98

|||:||||:|||.|||||:|.|||.| ||....|.|||||||.|||||.

O.cuniculus 51 EDFEVTLPQDVGPVLLLRLHLAPPALSRPLCLAAAGDAWFCRWVQLTPPG 100

H.sapiens 99 GGHLLFPCYQWLEGAGTLVLQEGTAKVSWADHHPVLQQQRQEELQARQEM 148

|..|.||||||||...:|.|:||.|:||.||.||.|.||||||||||.:.

O.cuniculus 101 GAPLRFPCYQWLEAPSSLALREGAARVSRADQHPALWQQRQEELQARPQA 150

H.sapiens 149 YQWKAYNPGWPHCLDEKTVEDLELNIKYSTAKNANFYLQAGSAFAEMKIK 198

|.||.|.|||||||:|.||:||:||:||..||||.|||..||..||:|||

O.cuniculus 151 YGWKTYLPGWPHCLNEVTVKDLDLNLKYPAAKNAAFYLNIGSVVAELKIK 200

H.sapiens 199 GLLDRKGLWRSLNEMKRIFNFRRTPAAEHAFEHWQEDAFFASQFLNGLNP 248

||||||||||||.||:|||||.||||.|:.|:||||||||||||||||||

O.cuniculus 201 GLLDRKGLWRSLREMRRIFNFHRTPAIEYVFDHWQEDAFFASQFLNGLNP 250

H.sapiens 249 VLIRRCHYLPKNFPVTDAMVASVLGPGTSLQAELEKGSLFLVDHGILSGI 298

||||||.:|||||||||||||.|||||:|||||||:||||||||||||.|

O.cuniculus 251 VLIRRCRHLPKNFPVTDAMVAPVLGPGSSLQAELERGSLFLVDHGILSSI 300

H.sapiens 299 QTNVINGKPQFSAAPMTLLYQSPGCGPLLPLAIQLSQTPGPNSPIFLPTD 348

:|||:||:|||||||:|||:|.||.|||:|||||||||||||||||||:|

O.cuniculus 301 RTNVVNGRPQFSAAPLTLLHQRPGRGPLVPLAIQLSQTPGPNSPIFLPSD 350

H.sapiens 349 DKWDWLLAKTWVRNAEFSFHEALTHLLHSHLLPEVFTLATLRQLPHCHPL 398

:|||||||||||||||||.||||||||..||||||||||.||||||||||

O.cuniculus 351 NKWDWLLAKTWVRNAEFSVHEALTHLLQGHLLPEVFTLAMLRQLPHCHPL 400

H.sapiens 399 FKLLIPHTRYTLHINTLARELLIVPGQVVDRSTGIGIEGFSELIQRNMKQ 448

|||||||||||||||||||||||.||||||||||:||.||||||||||:|

O.cuniculus 401 FKLLIPHTRYTLHINTLARELLIAPGQVVDRSTGLGIGGFSELIQRNMEQ 450

H.sapiens 449 LNYSLLCLPEDIRTRGVEDIPGYYYRDDGMQIWGAVERFVSEIIGIYYPS 498

|:|:.||||||||.|||||:|||||||||||||||:|||||||:||||||

O.cuniculus 451 LSYATLCLPEDIRARGVEDLPGYYYRDDGMQIWGAMERFVSEIVGIYYPS 500

H.sapiens 499 DESVQDDRELQAWVREIFSKGFLNQESSGIPSSLETREALVQYVTMVIFT 548

|.:||||:||||||||:|:||||:|||||:|||||||.||||||||||||

O.cuniculus 501 DAAVQDDQELQAWVREVFTKGFLSQESSGVPSSLETRGALVQYVTMVIFT 550

H.sapiens 549 CSAKHAAVSAGQFDSCAWMPNLPPSMQLPPPTSKGLATCEGFIATLPPVN 598

|||:|:::||||||||||||||||:||||||||||.||.|||:|||||||

O.cuniculus 551 CSARHSSISAGQFDSCAWMPNLPPTMQLPPPTSKGQATPEGFLATLPPVN 600

H.sapiens 599 ATCDVILALWLLSKEPGDQRPLGTYPDEHFTEEAPRRSIATFQSRLAQIS 648

|||||::||.:||||||.:|||||||||||||:|||||||.|||||||||

O.cuniculus 601 ATCDVVVALRVLSKEPGVRRPLGTYPDEHFTEDAPRRSIAAFQSRLAQIS 650

H.sapiens 649 RGIQERNQGLVLPYTYLDPPLIENSVSI 676

|.|:|||:||.|||.|||||||||||||

O.cuniculus 651 RDIRERNRGLALPYAYLDPPLIENSVSI 678

Grammomys (*Grammomys surdaster*)

H.sapiens 1 MAEFRVRVSTGEAFGAGTWDKVSVSIVGTRGESPPLPLDNLGKEFTAGAE 50

||:||:|||||||.|||||||||||||||.||||.:|||:|||||.||||

G.surdaster 1 MAKFRLRVSTGEACGAGTWDKVSVSIVGTHGESPLVPLDHLGKEFNAGAE 50

H.sapiens 51 EDFQVTLPEDVGRVLLLRVHKAPPVLPLLGPLA---PDAWFCRWFQLTPP 97

|||:||||:|||.||:||||||||..|| ||. |||||||||||...

G.surdaster 51 EDFEVTLPQDVGAVLMLRVHKAPPESPL--PLVSFPPDAWFCRWFQLEWL 98

H.sapiens 98 RGGHLLFPCYQWLEGAGTLVLQEGTAKVSWADHHPVLQQQRQEELQARQE 147

.|..|.|||||||||||.|||:||.|||||.||||.||.|||:||:.||:

G.surdaster 99 PGAALHFPCYQWLEGAGELVLREGAAKVSWQDHHPALQDQRQKELETRQD 148

H.sapiens 148 MYQWKAYNPGWPHCLDEKTVEDLELNIKYSTAKNANFYLQAGSAFAEMKI 197

||.||.|..|||||||.|||:||:||||||..|||.|:.:|.||...:|.

G.surdaster 149 MYSWKTYIEGWPHCLDHKTVKDLDLNIKYSAMKNAKFFFKAYSAITGLKF 198

H.sapiens 198 KGLLDRKGLWRSLNEMKRIFNFRRTPAAEHAFEHWQEDAFFASQFLNGLN 247

||||||.||||||.||:|:|||.:|||||:.|.|||||||||||||||||

G.surdaster 199 KGLLDRTGLWRSLREMRRMFNFHKTPAAEYVFAHWQEDAFFASQFLNGLN 248

H.sapiens 248 PVLIRRCHYLPKNFPVTDAMVASVLGPGTSLQAELEKGSLFLVDHGILSG 297

||||||||.|||||||||.|||.|||||||||||||||||||||||||||

G.surdaster 249 PVLIRRCHSLPKNFPVTDEMVAPVLGPGTSLQAELEKGSLFLVDHGILSG 298

H.sapiens 298 IQTNVINGKPQFSAAPMTLLYQSPGCGPLLPLAIQLSQTPGPNSPIFLPT 347

:||||||||||||||||||||||.|.|||||:||||.|||||::|||||:

G.surdaster 299 VQTNVINGKPQFSAAPMTLLYQSSGSGPLLPIAIQLKQTPGPDNPIFLPS 348

H.sapiens 348 DDKWDWLLAKTWVRNAEFSFHEALTHLLHSHLLPEVFTLATLRQLPHCHP 397

|||||||||||||||||:|.|||:|||.|:||:||||.||||||||.|||

G.surdaster 349 DDKWDWLLAKTWVRNAEYSIHEAVTHLFHAHLIPEVFALATLRQLPQCHP 398

H.sapiens 398 LFKLLIPHTRYTLHINTLARELLIVPGQVVDRSTGIGIEGFSELIQRNMK 447

||||||||.|||||||||||||||.||:|||:|||:||.||||||:|||:

G.surdaster 399 LFKLLIPHIRYTLHINTLARELLIAPGKVVDKSTGLGIGGFSELIKRNME 448

H.sapiens 448 QLNYSLLCLPEDIRTRGVEDIPGYYYRDDGMQIWGAVE-RFVSEIIGIYY 496

|||||:||||||||.|.||||||||||||||||||.:. .|||||:||||

G.surdaster 449 QLNYSVLCLPEDIRARDVEDIPGYYYRDDGMQIWGDIPCSFVSEIVGIYY 498

H.sapiens 497 PSDESVQDDRELQAWVREIFSKGFLNQESSGIPSSLETREALVQYVTMVI 546

|||.||:||:|||||||||||:|||::||||:||.|.|:|||||||||||

G.surdaster 499 PSDASVRDDQELQAWVREIFSEGFLSRESSGMPSLLNTQEALVQYVTMVI 548

H.sapiens 547 FTCSAKHAAVSAGQFDSCAWMPNLPPSMQLPPPTSKGLATCEGFIATLPP 596

||||||||||||||||.|||||||||:||||||||||....||||||||.

G.surdaster 549 FTCSAKHAAVSAGQFDFCAWMPNLPPTMQLPPPTSKGQTRPEGFIATLPA 598

H.sapiens 597 VNATCDVILALWLLSKEPGDQRPLGTYPDEHFTEEAPRRSIATFQSRLAQ 646

||||||||:|||||||||||.||||.||||||||:|||||:|.||.:|.|

G.surdaster 599 VNATCDVIIALWLLSKEPGDSRPLGHYPDEHFTEDAPRRSVAAFQRKLIQ 648

H.sapiens 647 ISRGIQERNQGLVLPYTYLDPPLIENSVSI 676

||.||::|||.|.|||||||||||||||||

G.surdaster 649 ISSGIRKRNQSLPLPYTYLDPPLIENSVSI 678

House mouse (*Mus musculus*)

H.sapiens 1 MAEFRVRVSTGEAFGAGTWDKVSVSIVGTRGESPPLPLDNLGKEFTAGAE 50

||:.|||||||||.|||||||||||||||.||||.:|||:|||||:||||

M.musculus 1 MAKCRVRVSTGEACGAGTWDKVSVSIVGTHGESPLVPLDHLGKEFSAGAE 50

H.sapiens 51 EDFQVTLPEDVGRVLLLRVHKAPP--VLPLLGPLAPDAWFCRWFQLTPPR 98

|||:||||:|||.||:|||||||| .|||:. ...||||||||:|....

M.musculus 51 EDFEVTLPQDVGTVLMLRVHKAPPEVSLPLMS-FRSDAWFCRWFELEWLP 99

H.sapiens 99 GGHLLFPCYQWLEGAGTLVLQEGTAKVSWADHHPVLQQQRQEELQARQEM 148

|..|.|||||||||||.|||:||.|||||.||||.||.|||:||::||:|

M.musculus 100 GAALHFPCYQWLEGAGELVLREGAAKVSWQDHHPTLQDQRQKELESRQKM 149

H.sapiens 149 YQWKAYNPGWPHCLDEKTVEDLELNIKYSTAKNANFYLQAGSAFAEMKIK 198

|.||.|..|||.|||.:||:||:||||||..|||..:.:|.||:.|:|:|

M.musculus 150 YSWKTYIEGWPRCLDHETVKDLDLNIKYSAMKNAKLFFKAHSAYTELKVK 199

H.sapiens 199 GLLDRKGLWRSLNEMKRIFNFRRTPAAEHAFEHWQEDAFFASQFLNGLNP 248

|||||.||||||.||:|:||||:|||||:.|.|||||||||||||||:||

M.musculus 200 GLLDRTGLWRSLREMRRLFNFRKTPAAEYVFAHWQEDAFFASQFLNGINP 249

H.sapiens 249 VLIRRCHYLPKNFPVTDAMVASVLGPGTSLQAELEKGSLFLVDHGILSGI 298

|||||||.||.||||||.|||.|||||||||||||||||||||||||||:

M.musculus 250 VLIRRCHSLPNNFPVTDEMVAPVLGPGTSLQAELEKGSLFLVDHGILSGV 299

H.sapiens 299 QTNVINGKPQFSAAPMTLLYQSPGCGPLLPLAIQLSQTPGPNSPIFLPTD 348

.||::||||||||||||||:||.|.|||||:||||.|||||::|||||:|

M.musculus 300 HTNILNGKPQFSAAPMTLLHQSSGSGPLLPIAIQLKQTPGPDNPIFLPSD 349

H.sapiens 349 DKWDWLLAKTWVRNAEFSFHEALTHLLHSHLLPEVFTLATLRQLPHCHPL 398

|.||||||||||||:||..|||:|||||:||:||||.||||||||.||||

M.musculus 350 DTWDWLLAKTWVRNSEFYIHEAVTHLLHAHLIPEVFALATLRQLPRCHPL 399

H.sapiens 399 FKLLIPHTRYTLHINTLARELLIVPGQVVDRSTGIGIEGFSELIQRNMKQ 448

|||||||.||||||||||||||:.||:::|:|||:|..|||:||:|||:|

M.musculus 400 FKLLIPHIRYTLHINTLARELLVAPGKLIDKSTGLGTGGFSDLIKRNMEQ 449

H.sapiens 449 LNYSLLCLPEDIRTRGVEDIPGYYYRDDGMQIWGAVERFVSEIIGIYYPS 498

||||:||||||||.|||||||||||||||||||||::.|||||:.|||||

M.musculus 450 LNYSVLCLPEDIRARGVEDIPGYYYRDDGMQIWGAIKSFVSEIVSIYYPS 499

H.sapiens 499 DESVQDDRELQAWVREIFSKGFLNQESSGIPSSLETREALVQYVTMVIFT 548

|.|||||:|||||||||||:|||.:||||:||.|:||||||||:||||||

M.musculus 500 DTSVQDDQELQAWVREIFSEGFLGRESSGMPSLLDTREALVQYITMVIFT 549

H.sapiens 549 CSAKHAAVSAGQFDSCAWMPNLPPSMQLPPPTSKGLATCEGFIATLPPVN 598

|||||||||:||||||.|||||||:||||||||||.|..|.||||||.||

M.musculus 550 CSAKHAAVSSGQFDSCVWMPNLPPTMQLPPPTSKGQARPESFIATLPAVN 599

H.sapiens 599 ATCDVILALWLLSKEPGDQRPLGTYPDEHFTEEAPRRSIATFQSRLAQIS 648

::...|:||||||.|||||||||.||||||||:|||||:|.||.:|.|||

M.musculus 600 SSSYHIIALWLLSAEPGDQRPLGHYPDEHFTEDAPRRSVAAFQRKLIQIS 649

H.sapiens 649 RGIQERNQGLVLPYTYLDPPLIENSVSI 676

:||:|||:||.|||||||||||||||||

M.musculus 650 KGIRERNRGLALPYTYLDPPLIENSVSI 677

Sikkim mouse (*Mus pahari*)

H.sapiens 1 MAEFRVRVSTGEAFGAGTWDKVSVSIVGTRGESPPLPLDNLGKEFTAGAE 50

||:||||||||||.|||||||||||||||.||||.:|||:|||||:||||

M.pahari 1 MAKFRVRVSTGEACGAGTWDKVSVSIVGTHGESPLIPLDHLGKEFSAGAE 50

H.sapiens 51 EDFQVTLPEDVGRVLLLRVHKAPP--VLPLLGPLAPDAWFCRWFQLTPPR 98

|||:||||:|||.||:|||||||| .|||:. ...||||||||:|....

M.pahari 51 EDFEVTLPQDVGAVLMLRVHKAPPEASLPLMS-FPSDAWFCRWFELEWLP 99

H.sapiens 99 GGHLLFPCYQWLEGAGTLVLQEGTAKVSWADHHPVLQQQRQEELQARQEM 148

|..|.|||||||||||.|||:||.|||||.||||.||.|||:||::||:|

M.pahari 100 GAALHFPCYQWLEGAGELVLREGAAKVSWQDHHPTLQDQRQKELESRQKM 149

H.sapiens 149 YQWKAYNPGWPHCLDEKTVEDLELNIKYSTAKNANFYLQAGSAFAEMKIK 198

|.||.|..|||.|||.:||:||:||||||..|||.|:.:|.||:.|:|||

M.pahari 150 YSWKTYIEGWPRCLDHETVKDLDLNIKYSAMKNAKFFFKAHSAYTELKIK 199

H.sapiens 199 GLLDRKGLWRSLNEMKRIFNFRRTPAAEHAFEHWQEDAFFASQFLNGLNP 248

|||||.||||||.||:|:||||:|||||:.|.|||||||||||||||:||

M.pahari 200 GLLDRTGLWRSLREMRRLFNFRKTPAAEYVFAHWQEDAFFASQFLNGINP 249

H.sapiens 249 VLIRRCHYLPKNFPVTDAMVASVLGPGTSLQAELEKGSLFLVDHGILSGI 298

|||||||.|||||||||.|||.|||||||||||||||||||||||||||:

M.pahari 250 VLIRRCHSLPKNFPVTDEMVAPVLGPGTSLQAELEKGSLFLVDHGILSGV 299

H.sapiens 299 QTNVINGKPQFSAAPMTLLYQSPGCGPLLPLAIQLSQTPGPNSPIFLPTD 348

|||::||||||||||||||:||.|.|.|||:||||.|||||::|||||:|

M.pahari 300 QTNILNGKPQFSAAPMTLLHQSSGSGTLLPIAIQLKQTPGPDNPIFLPSD 349

H.sapiens 349 DKWDWLLAKTWVRNAEFSFHEALTHLLHSHLLPEVFTLATLRQLPHCHPL 398

|.||||||||||||:||..|||:|||||:||:||||.||||||||.||||

M.pahari 350 DTWDWLLAKTWVRNSEFYIHEAVTHLLHAHLIPEVFALATLRQLPTCHPL 399

H.sapiens 399 FKLLIPHTRYTLHINTLARELLIVPGQVVDRSTGIGIEGFSELIQRNMKQ 448

|||||||.|||||||||||||||.||::||:|||:|..|||:||:|||:|

M.pahari 400 FKLLIPHIRYTLHINTLARELLIAPGKLVDKSTGLGTGGFSDLIKRNMEQ 449

H.sapiens 449 LNYSLLCLPEDIRTRGVEDIPGYYYRDDGMQIWGAVERFVSEIIGIYYPS 498

||||:|||||||:.|||||||||||||||||||||::.|||||:||||||

M.pahari 450 LNYSVLCLPEDIQARGVEDIPGYYYRDDGMQIWGAIKSFVSEIVGIYYPS 499

H.sapiens 499 DESVQDDRELQAWVREIFSKGFLNQESSGIPSSLETREALVQYVTMVIFT 548

|.|||||:||||||.||||:|||::||||:||.|:|:||||||:||||||

M.pahari 500 DTSVQDDQELQAWVGEIFSEGFLSRESSGMPSLLDTQEALVQYITMVIFT 549

H.sapiens 549 CSAKHAAVSAGQFDSCAWMPNLPPSMQLPPPTSKGLATCEGFIATLPPVN 598

|||||||||:||||||.|||||||:||||||||||....|.||.|||.||

M.pahari 550 CSAKHAAVSSGQFDSCVWMPNLPPTMQLPPPTSKGQTRPESFITTLPAVN 599

H.sapiens 599 ATCDVILALWLLSKEPGDQRPLGTYPDEHFTEEAPRRSIATFQSRLAQIS 648

::...|:||||||.|||||||||.||||||||:|||||:|.||.:|.|||

M.pahari 600 SSSYHIIALWLLSAEPGDQRPLGHYPDEHFTEDAPRRSVAAFQRKLIQIS 649

H.sapiens 649 RGIQERNQGLVLPYTYLDPPLIENSVSI 676

:||:|||:||.|||||||||||||||||

M.pahari 650 KGIRERNRGLALPYTYLDPPLIENSVSI 677

mouse (*Mus caroli*)

H.sapiens 1 MAEFRVRVSTGEAFGAGTWDKVSVSIVGTRGESPPLPLDNLGKEFTAGAE 50

||:.|||||||||.||||||||||||:||.||||.:|||:|||||:||||

M.caroli 1 MAKCRVRVSTGEACGAGTWDKVSVSILGTHGESPLVPLDHLGKEFSAGAE 50

H.sapiens 51 EDFQVTLPEDVGRVLLLRVHKAPP--VLPLLGPLAPDAWFCRWFQLTPPR 98

|||:||||:|||.||:|||||||| .|||:. ...||||||||:|....

M.caroli 51 EDFEVTLPQDVGTVLMLRVHKAPPEASLPLMS-FRSDAWFCRWFELEWLP 99

H.sapiens 99 GGHLLFPCYQWLEGAGTLVLQEGTAKVSWADHHPVLQQQRQEELQARQEM 148

|..|.|||||||||||.|||:||.|||||.||||.||.|||:||::||:|

M.caroli 100 GAALHFPCYQWLEGAGELVLREGAAKVSWQDHHPTLQDQRQKELESRQKM 149

H.sapiens 149 YQWKAYNPGWPHCLDEKTVEDLELNIKYSTAKNANFYLQAGSAFAEMKIK 198

|.||.|..|||.|||.:|.:||:||||||..|||.|:.:|.||:.|:|:|

M.caroli 150 YSWKTYIEGWPRCLDHETAKDLDLNIKYSAMKNAKFFFKAYSAYTELKVK 199

H.sapiens 199 GLLDRKGLWRSLNEMKRIFNFRRTPAAEHAFEHWQEDAFFASQFLNGLNP 248

|||||.||||||.||:|:||||:.||||:.|.|||||||||||||||:||

M.caroli 200 GLLDRTGLWRSLREMRRLFNFRKAPAAEYVFAHWQEDAFFASQFLNGINP 249

H.sapiens 249 VLIRRCHYLPKNFPVTDAMVASVLGPGTSLQAELEKGSLFLVDHGILSGI 298

|||||||.||.||||||.|||.|||||||||||||||||||||||||||:

M.caroli 250 VLIRRCHNLPNNFPVTDEMVAPVLGPGTSLQAELEKGSLFLVDHGILSGV 299

H.sapiens 299 QTNVINGKPQFSAAPMTLLYQSPGCGPLLPLAIQLSQTPGPNSPIFLPTD 348

.||::||||||||||||||:||.|.|||||:||||.|||||::|||||:|

M.caroli 300 HTNILNGKPQFSAAPMTLLHQSSGSGPLLPIAIQLKQTPGPDNPIFLPSD 349

H.sapiens 349 DKWDWLLAKTWVRNAEFSFHEALTHLLHSHLLPEVFTLATLRQLPHCHPL 398

|.||||||||||||:||..|||:|||||:||:||||.||||||||.||||

M.caroli 350 DTWDWLLAKTWVRNSEFYIHEAITHLLHAHLIPEVFALATLRQLPRCHPL 399

H.sapiens 399 FKLLIPHTRYTLHINTLARELLIVPGQVVDRSTGIGIEGFSELIQRNMKQ 448

|||||||.||||||||||||.|:.||:::|:|||:|.||||:||:|||:|

M.caroli 400 FKLLIPHIRYTLHINTLAREQLVAPGKLIDKSTGLGTEGFSDLIKRNMEQ 449

H.sapiens 449 LNYSLLCLPEDIRTRGVEDIPGYYYRDDGMQIWGAVERFVSEIIGIYYPS 498

||||:||||||||.|||||||||||||||||||||::.|||||:.|||||

M.caroli 450 LNYSVLCLPEDIRARGVEDIPGYYYRDDGMQIWGAIKSFVSEIVSIYYPS 499

H.sapiens 499 DESVQDDRELQAWVREIFSKGFLNQESSGIPSSLETREALVQYVTMVIFT 548

|.|||||:|||||||||||:|||.:||||:||.|:|:||||||:||||||

M.caroli 500 DTSVQDDQELQAWVREIFSEGFLGRESSGMPSLLDTQEALVQYITMVIFT 549

H.sapiens 549 CSAKHAAVSAGQFDSCAWMPNLPPSMQLPPPTSKGLATCEGFIATLPPVN 598

|||||||||.||||||.|||||||:||||||||||.|..|.||||||.||

M.caroli 550 CSAKHAAVSLGQFDSCVWMPNLPPTMQLPPPTSKGQARPESFIATLPAVN 599

H.sapiens 599 ATCDVILALWLLSKEPGDQRPLGTYPDEHFTEEAPRRSIATFQSRLAQIS 648

::...|:||||||.|||||||||.||||||||:|||||:|.||.:|.|||

M.caroli 600 SSSYHIIALWLLSAEPGDQRPLGHYPDEHFTEDAPRRSVAAFQRKLIQIS 649

H.sapiens 649 RGIQERNQGLVLPYTYLDPPLIENSVSI 676

.||:|||:||.|||||||||||||||||

M.caroli 650 EGIRERNRGLALPYTYLDPPLIENSVSI 677

Southern multimammate mouse (*Mastomys coucha*)

H.sapiens 1 MAEFRVRVSTGEAFGAGTWDKVSVSIVGTRGESPPLPLDNLGKEFTAGAE 50

||:|:||||||||.|||||||||||||||.||||.:|||:|||||:|.||

M.coucha 1 MAKFKVRVSTGEACGAGTWDKVSVSIVGTHGESPLVPLDHLGKEFSASAE 50

H.sapiens 51 EDFQVTLPEDVGRVLLLRVHKAPPVLPLLGPLA---PDAWFCRWFQLTPP 97

|||:||||:|||.||:||:||||...|| ||. .||||||||||...

M.coucha 51 EDFEVTLPQDVGAVLMLRIHKAPREAPL--PLVSFPSDAWFCRWFQLEWL 98

H.sapiens 98 RGGHLLFPCYQWLEGAGTLVLQEGTAKVSWADHHPVLQQQRQEELQARQE 147

.|..|.|||||||||||.|||:||.|||||.||||:||.|||:||:|||:

M.coucha 99 PGAALRFPCYQWLEGAGELVLREGAAKVSWEDHHPILQDQRQKELKARQK 148

H.sapiens 148 MYQWKAYNPGWPHCLDEKTVEDLELNIKYSTAKNANFYLQAGSAFAEMKI 197

||.||.|..|||||||.:||:||:||||||..|||.|:.:|.|||.|:||

M.coucha 149 MYSWKTYIEGWPHCLDHETVKDLDLNIKYSVMKNAKFFFKAHSAFTELKI 198

H.sapiens 198 KGLLDRKGLWRSLNEMKRIFNFRRTPAAEHAFEHWQEDAFFASQFLNGLN 247

||.|||.||||||.||||:||||:|||||:.|.|||||||||||||||:|

M.coucha 199 KGFLDRTGLWRSLREMKRMFNFRKTPAAEYVFAHWQEDAFFASQFLNGVN 248

H.sapiens 248 PVLIRRCHYLPKNFPVTDAMVASVLGPGTSLQAELEKGSLFLVDHGILSG 297

|||||||..|||||||||.|||.|||||||||||||||||||||||||||

M.coucha 249 PVLIRRCRSLPKNFPVTDEMVAPVLGPGTSLQAELEKGSLFLVDHGILSG 298

H.sapiens 298 IQTNVINGKPQFSAAPMTLLYQSPGCGPLLPLAIQLSQTPGPNSPIFLPT 347

:||||||||||||||||||||||.|.|||||:||||:|||||::|||||:

M.coucha 299 VQTNVINGKPQFSAAPMTLLYQSSGSGPLLPIAIQLTQTPGPDNPIFLPS 348

H.sapiens 348 DDKWDWLLAKTWVRNAEFSFHEALTHLLHSHLLPEVFTLATLRQLPHCHP 397

|||||||||||||||||||.|||:|||||:||:||||.||||||||.|||

M.coucha 349 DDKWDWLLAKTWVRNAEFSVHEAVTHLLHAHLIPEVFALATLRQLPRCHP 398

H.sapiens 398 LFKLLIPHTRYTLHINTLARELLIVPGQVVDRSTGIGIEGFSELIQRNMK 447

||||||||.|:|||||||||||||.||::||:|||:|..|||:||:|||:

M.coucha 399 LFKLLIPHIRFTLHINTLARELLIAPGKLVDKSTGLGTGGFSDLIKRNME 448

H.sapiens 448 QLNYSLLCLPEDIRTRGVEDIPGYYYRDDGMQIWGAVERFVSEIIGIYYP 497

|||||:||||||||.|.|||||||||||||||||||::.|||||:||||.

M.coucha 449 QLNYSVLCLPEDIRARDVEDIPGYYYRDDGMQIWGAIKSFVSEIVGIYYS 498

H.sapiens 498 SDESVQDDRELQAWVREIFSKGFLNQESSGIPSSLETREALVQYVTMVIF 547

||.||:||:|||||||||||:|||::||||:||.|:|:||||||:|||||

M.coucha 499 SDTSVRDDQELQAWVREIFSEGFLSRESSGMPSLLDTQEALVQYITMVIF 548

H.sapiens 548 TCSAKHAAVSAGQFDSCAWMPNLPPSMQLPPPTSKGLATCEGFIATLPPV 597

|||||||||||||||:|.|||||||:||||||||||.|..|.||||||.|

M.coucha 549 TCSAKHAAVSAGQFDACVWMPNLPPTMQLPPPTSKGQARPESFIATLPAV 598

H.sapiens 598 NATCDVILALWLLSKEPGDQRPLGTYPDEHFTEEAPRRSIATFQSRLAQI 647

|::|..|:||||||.|||||||||.||||||||:|||||:|.||.:|.||

M.coucha 599 NSSCYHIIALWLLSTEPGDQRPLGHYPDEHFTEDAPRRSVAAFQRKLFQI 648

H.sapiens 648 SRGIQERNQGLVLPYTYLDPPLIENSVSI 676

|:.|||||:.|.|||||||||||||||||

M.coucha 649 SKDIQERNRSLALPYTYLDPPLIENSVSI 677

Long-tailed chinchilla (*Chinchilla lanigera*)

H.sapiens 1 MAEFRVRVSTGEAFGAGTWDKVSVSIVGTRGESPPLPLDNLGKEFTAGAE 50

||:||||||||||.|||||||||||||||:|||||||||:||||||.|.|

C.lanigera 1 MAKFRVRVSTGEACGAGTWDKVSVSIVGTQGESPPLPLDHLGKEFTQGTE 50

H.sapiens 51 EDFQVTLPEDVGRVLLLRVHKAPPVLPL-LGPLAPDAWFCRWFQLTPPRG 99

|||:|.||||||.||||||.||||.||. ||||.|||||||||||.||||

C.lanigera 51 EDFEVMLPEDVGTVLLLRVQKAPPPLPSPLGPLRPDAWFCRWFQLEPPRG 100

H.sapiens 100 GHLLFPCYQWLEGAGTLVLQEGTAKVSWADHHPVLQQQRQEELQARQEMY 149

..|:||||||||.||||||:||.|||||||.||:|:.||:|||||:|:||

C.lanigera 101 APLVFPCYQWLERAGTLVLREGAAKVSWADDHPILEHQRREELQAKQKMY 150

H.sapiens 150 QWKAYNPGWPHCLDEKTVEDLELNIKYSTAKNANFYLQAGSAFAEMKIKG 199

.||.|.|||||||.|:||:||:||:|||..|.|.|.|:.||..||:|:||

C.lanigera 151 WWKTYLPGWPHCLGEETVKDLDLNVKYSIVKKAKFSLKLGSGVAELKLKG 200

H.sapiens 200 LLDRKGLWRSLNEMKRIFNFRRTPAAEHAFEHWQEDAFFASQFLNGLNPV 249

||||.||||||.||||:.||||:||.|:.|:|||||:|||||||||||||

C.lanigera 201 LLDRTGLWRSLREMKRMLNFRRSPAGEYVFKHWQEDSFFASQFLNGLNPV 250

H.sapiens 250 LIRRCHYLPKNFPVTDAMVASVLGPGTSLQAELEKGSLFLVDHGILSGIQ 299

|||||..|||||||||||||.|||||||||||||:||:||||||||||::

C.lanigera 251 LIRRCRSLPKNFPVTDAMVAPVLGPGTSLQAELERGSIFLVDHGILSGLK 300

H.sapiens 300 TNVINGKPQFSAAPMTLLYQSPGCGPLLPLAIQLSQTPGPNSPIFLPTDD 349

||||||:|||||||||||||.||.||||||||||||||||:||||||:|:

C.lanigera 301 TNVINGRPQFSAAPMTLLYQHPGGGPLLPLAIQLSQTPGPDSPIFLPSDE 350

H.sapiens 350 KWDWLLAKTWVRNAEFSFHEALTHLLHSHLLPEVFTLATLRQLPHCHPLF 399

|||||||||||||||||.|||||||||:||||||||||||.|||||||||

C.lanigera 351 KWDWLLAKTWVRNAEFSVHEALTHLLHAHLLPEVFTLATLCQLPHCHPLF 400

H.sapiens 400 KLLIPHTRYTLHINTLARELLIVPGQVVDRSTGIGIEGFSELIQRNMKQL 449

|||||||||||:|||||||.||.||:|||||||:||.||||||||||:||

C.lanigera 401 KLLIPHTRYTLYINTLAREQLIAPGKVVDRSTGLGIGGFSELIQRNMEQL 450

H.sapiens 450 NYSLLCLPEDIRTRGVEDIPGYYYRDDGMQIWGAVERFVSEIIGIYYPSD 499

.||:||||||||.|||||||.|||||||::||.|||:|||||||||||||

C.lanigera 451 TYSVLCLPEDIRARGVEDIPDYYYRDDGLRIWEAVEQFVSEIIGIYYPSD 500

H.sapiens 500 ESVQDDRELQAWVREIFSKGFLNQESSGIPSSLETREALVQYVTMVIFTC 549

.||:||||||||||:|||.|||.:||||:||||:|:|||::|:|||||||

C.lanigera 501 ASVRDDRELQAWVRDIFSNGFLGRESSGMPSSLDTQEALIRYLTMVIFTC 550

H.sapiens 550 SAKHAAVSAGQFDSCAWMPNLPPSMQLPPPTSKGLATCEGFIATLPPVNA 599

||||||||:||||||.|||||||:||||||||||.|..||||||||||||

C.lanigera 551 SAKHAAVSSGQFDSCVWMPNLPPTMQLPPPTSKGQARPEGFIATLPPVNA 600

H.sapiens 600 TCDVILALWLLSKEPGDQRPLGTYPDEHFTEEAPRRSIATFQSRLAQISR 649

|||:|:.|||||:||||:|||||||||:||||||::||.||||.|||||:

C.lanigera 601 TCDIIITLWLLSQEPGDRRPLGTYPDEYFTEEAPKQSITTFQSHLAQISQ 650

H.sapiens 650 GIQERNQGLVLPYTYLDPPLIENSVSI 676

.|:.|||||.|||||||||||||||||

C.lanigera 651 DIRRRNQGLDLPYTYLDPPLIENSVSI 677

Guinea pig (*Cavia porcellus*)

H.sapiens 1 MAEFRVRVSTGEAFGAGTWDKVSVSIVGTRGESPPLPLDNLGKEFTAGAE 50

||:||:|||||||.|||||||||||||||.|||||||||:|||||:||||

C.porcellus 1 MAKFRLRVSTGEACGAGTWDKVSVSIVGTLGESPPLPLDHLGKEFSAGAE 50

H.sapiens 51 EDFQVTLPEDVGRVLLLRVHKAPPVL-PLLGPLAPDAWFCRWFQLTPPRG 99

|||:||||:|||.||||||||.||:| .|||....|||||||.||.||:|

C.porcellus 51 EDFEVTLPKDVGSVLLLRVHKEPPLLSTLLGSQPSDAWFCRWIQLEPPQG 100

H.sapiens 100 GHLLFPCYQWLEGAGTLVLQEGTAKVSWADHHPVLQQQRQEELQARQEMY 149

..|||||||||:||.||||:||.|||||||.||.|:|||||||||:|.||

C.porcellus 101 APLLFPCYQWLDGASTLVLREGAAKVSWADEHPTLEQQRQEELQAKQRMY 150

H.sapiens 150 QWKAYNPGWPHCLDEKTVEDLELNIKYSTAKNANFYLQAGSAFAEMKIKG 199

.||.|.||||.||||.:|:||:||:|||.||...|.|:.||..||:|:||

C.porcellus 151 WWKTYLPGWPRCLDEDSVKDLDLNVKYSVAKKTKFTLKLGSGIAELKLKG 200

H.sapiens 200 LLDRKGLWRSLNEMKRIFNFRRTPAAEHAFEHWQEDAFFASQFLNGLNPV 249

||||.||||||.||||:.||||:.|.|:|..||:||:|||||||||||||

C.porcellus 201 LLDRTGLWRSLREMKRMLNFRRSAAGEYALTHWREDSFFASQFLNGLNPV 250

H.sapiens 250 LIRRCHYLPKNFPVTDAMVASVLGPGTSLQAELEKGSLFLVDHGILSGIQ 299

|||||..||:||||||.|||.|||||||||||||:||||||||.||||||

C.porcellus 251 LIRRCRSLPENFPVTDVMVAPVLGPGTSLQAELERGSLFLVDHSILSGIQ 300

H.sapiens 300 TNVINGKPQFSAAPMTLLYQSPGCGPLLPLAIQLSQTPGPNSPIFLPTDD 349

||||||:|||||||:|||||.||.||||||||||||||||:||||||:|:

C.porcellus 301 TNVINGRPQFSAAPVTLLYQPPGGGPLLPLAIQLSQTPGPSSPIFLPSDE 350

H.sapiens 350 KWDWLLAKTWVRNAEFSFHEALTHLLHSHLLPEVFTLATLRQLPHCHPLF 399

.||||||||||||||||.||||||||.:||||||||:|||||||.|||||

C.porcellus 351 TWDWLLAKTWVRNAEFSVHEALTHLLQAHLLPEVFTMATLRQLPRCHPLF 400

H.sapiens 400 KLLIPHTRYTLHINTLARELLIVPGQVVDRSTGIGIEGFSELIQRNMKQL 449

|||||||||||:||||||:|||.||||||||||:|:|||||||||||:||

C.porcellus 401 KLLIPHTRYTLYINTLARQLLIAPGQVVDRSTGLGVEGFSELIQRNMQQL 450

H.sapiens 450 NYSLLCLPEDIRTRGVEDIPGYYYRDDGMQIWGAVERFVSEIIGIYYPSD 499

:||.||||||||.|||||||.|||||||:::|.|||.||||:||||||||

C.porcellus 451 SYSSLCLPEDIRARGVEDIPDYYYRDDGLRVWAAVEHFVSEMIGIYYPSD 500

H.sapiens 500 ESVQDDRELQAWVREIFSKGFLNQESSGIPSSLETREALVQYVTMVIFTC 549

.||.||.|||||||:|||.|||.:||||:||||:||||||||:|||||||

C.porcellus 501 ASVLDDGELQAWVRDIFSNGFLGRESSGVPSSLDTREALVQYITMVIFTC 550

H.sapiens 550 SAKHAAVSAGQFDSCAWMPNLPPSMQLPPPTSKGLATCEGFIATLPPVNA 599

||||||||:.|||||.||||||.:||||||||||.||.||||||||||||

C.porcellus 551 SAKHAAVSSSQFDSCVWMPNLPSTMQLPPPTSKGQATPEGFIATLPPVNA 600

H.sapiens 600 TCDVILALWLLSKEPGDQRPLGTYPDEHFTEEAPRRSIATFQSRLAQISR 649

|||:|.|||||||||||:|||||||||||||:.|::|||.||:|||||||

C.porcellus 601 TCDIINALWLLSKEPGDRRPLGTYPDEHFTEDTPKQSIAAFQNRLAQISR 650

H.sapiens 650 GIQERNQGLVLPYTYLDPPLIENSVSI 676

.||:|||||.|||||||||||||||||

C.porcellus 651 DIQQRNQGLELPYTYLDPPLIENSVSI 677

American pika (*Ochotona princeps*)

H.sapiens 1 MAEFRVRVSTGEAFGAGTWDKVSVSIVGTRGESPPLPLDNLGKEFTAGAE 50

||:|.||||||:||||||||.||:|||||.||||.:.||:|||||.||||

O.princeps 1 MAKFGVRVSTGKAFGAGTWDSVSISIVGTLGESPRMRLDHLGKEFAAGAE 50

H.sapiens 51 EDFQVTLPEDVGRVLLLRVHKAPPVLP-LLGPLAPDAWFCRWFQLTPPRG 99

|||:||||:|:|.|||||||.|||.|| .||..|.|||||||.||.||||

O.princeps 51 EDFEVTLPQDLGPVLLLRVHLAPPALPRALGLPAADAWFCRWIQLKPPRG 100

H.sapiens 100 GHLLFPCYQWLEGAGTLVLQEGTAKVSWADHHPVLQQQRQEELQARQEMY 149

..|.|||||||||..:|||:||.|||||||.||.||||||||||||:::|

O.princeps 101 APLRFPCYQWLEGPRSLVLREGAAKVSWADQHPALQQQRQEELQARRQLY 150

H.sapiens 150 QWKAYNPGWPHCLDEKTVEDLELNIKYSTAKNANFYLQAGSAFAEMKIKG 199

.||.|.||||.||||.||:||:||:|||.||:|.|||...|..||:||||

O.princeps 151 SWKTYAPGWPRCLDEATVKDLDLNLKYSAAKSATFYLNLNSVRAELKIKG 200

H.sapiens 200 LLDRKGLWRSLNEMKRIFNFRRTPAAEHAFEHWQEDAFFASQFLNGLNPV 249

|||||||||||.|||||||||:|||.|:|.||||||||||||||||||||

O.princeps 201 LLDRKGLWRSLREMKRIFNFRKTPAVEYACEHWQEDAFFASQFLNGLNPV 250

H.sapiens 250 LIRRCHYLPKNFPVTDAMVASVLGPGTSLQAELEKGSLFLVDHGILSGIQ 299

|||||.:||.||||||||||.|||||||||||||:||||||||||||||.

O.princeps 251 LIRRCRHLPSNFPVTDAMVAPVLGPGTSLQAELERGSLFLVDHGILSGIP 300

H.sapiens 300 TNVINGKPQFSAAPMTLLYQSPGCGPLLPLAIQLSQTPGPNSPIFLPTDD 349

||||||:|||||||:|||||.||.|||:||||||||||||::|||||:||

O.princeps 301 TNVINGRPQFSAAPLTLLYQHPGHGPLVPLAIQLSQTPGPDNPIFLPSDD 350

H.sapiens 350 KWDWLLAKTWVRNAEFSFHEALTHLLHSHLLPEVFTLATLRQLPHCHPLF 399

|||||||||||||||||.|||||||||:||||||||||.|||||||||||

O.princeps 351 KWDWLLAKTWVRNAEFSVHEALTHLLHAHLLPEVFTLAMLRQLPHCHPLF 400

H.sapiens 400 KLLIPHTRYTLHINTLARELLIVPGQVVDRSTGIGIEGFSELIQRNMKQL 449

||||||.|||||||||||:|||.||||||:|||:||.||||||||||:||

O.princeps 401 KLLIPHMRYTLHINTLARQLLIAPGQVVDKSTGLGIGGFSELIQRNMEQL 450

H.sapiens 450 NYSLLCLPEDIRTRGVEDIPGYYYRDDGMQIWGAVERFVSEIIGIYYPSD 499

||:.||||:||.:|||||||||||||||:|||.||||||||::..|||||

O.princeps 451 NYATLCLPQDIHSRGVEDIPGYYYRDDGLQIWEAVERFVSEMVSTYYPSD 500

H.sapiens 500 ESVQDDRELQAWVREIFSKGFLNQESSGIPSSLETREALVQYVTMVIFTC 549

.||.:|.||||||||||.:|||::||||.|||||||||||||||||||||

O.princeps 501 MSVWEDSELQAWVREIFCEGFLSRESSGFPSSLETREALVQYVTMVIFTC 550

H.sapiens 550 SAKHAAVSAGQFDSCAWMPNLPPSMQLPPPTSKGLATCEGFIATLPPVNA 599

||||:|||||||||||||||||.|||||||..||.|..||||||||||||

O.princeps 551 SAKHSAVSAGQFDSCAWMPNLPASMQLPPPKCKGQARPEGFIATLPPVNA 600

H.sapiens 600 TCDVILALWLLSKEPGDQRPLGTYPDEHFTEEAPRRSIATFQSRLAQISR 649

|||:|:|||||||||||:|||||||||||||||||.|||.|||||||||.

O.princeps 601 TCDIIIALWLLSKEPGDRRPLGTYPDEHFTEEAPRCSIAAFQSRLAQISC 650

H.sapiens 650 GIQERNQGLVLPYTYLDPPLIENSVSI 676

.|:.||:||.|||||||||||||||||

O.princeps 651 NIRARNRGLALPYTYLDPPLIENSVSI 677

Plateau pika (*Ochotona curzoniae*)

H.sapiens 1 MAEFRVRVSTGEAFGAGTWDKVSVSIVGTRGESPPLPLDNLGKEFTAGAE 50

||:|.||||||:||||||||.||:|||||.||||.:.||:.||||.||||

O.curzoniae 1 MAKFGVRVSTGKAFGAGTWDSVSISIVGTLGESPRMRLDHFGKEFAAGAE 50

H.sapiens 51 EDFQVTLPEDVGRVLLLRVHKAPPVLP-LLGPLAPDAWFCRWFQLTPPRG 99

|||:||||:|:|.|||||||.|||.|| .||..|.|||||||.|||||||

O.curzoniae 51 EDFEVTLPQDLGPVLLLRVHLAPPELPRALGLPAADAWFCRWIQLTPPRG 100

H.sapiens 100 GHLLFPCYQWLEGAGTLVLQEGTAKVSWADHHPVLQQQRQEELQARQEMY 149

..|.|||||||||..:|||:||.|||||||.||.||||||||||||:::|

O.curzoniae 101 APLRFPCYQWLEGPRSLVLREGAAKVSWADQHPALQQQRQEELQARRQLY 150

H.sapiens 150 QWKAYNPGWPHCLDEKTVEDLELNIKYSTAKNANFYLQAGSAFAEMKIKG 199

.||.|.||||.||||:||:||:||:|||.||:|.|||...|..||:||||

O.curzoniae 151 SWKTYAPGWPRCLDEETVKDLDLNLKYSAAKSATFYLNLNSVRAELKIKG 200

H.sapiens 200 LLDRKGLWRSLNEMKRIFNFRRTPAAEHAFEHWQEDAFFASQFLNGLNPV 249

|||||||||||.|||||||||:|||.|:|.||||||||||||||||:|||

O.curzoniae 201 LLDRKGLWRSLREMKRIFNFRKTPAVEYACEHWQEDAFFASQFLNGINPV 250

H.sapiens 250 LIRRCHYLPKNFPVTDAMVASVLGPGTSLQAELEKGSLFLVDHGILSGIQ 299

||.||.:||.||||||||||.||||||||||||::||||||||||||||.

O.curzoniae 251 LIHRCRHLPSNFPVTDAMVAPVLGPGTSLQAELKRGSLFLVDHGILSGIP 300

H.sapiens 300 TNVINGKPQFSAAPMTLLYQSPGCGPLLPLAIQLSQTPGPNSPIFLPTDD 349

||||||:|||||||:|||||.||.|||:||||||||||||:||||||:||

O.curzoniae 301 TNVINGRPQFSAAPLTLLYQRPGHGPLVPLAIQLSQTPGPDSPIFLPSDD 350

H.sapiens 350 KWDWLLAKTWVRNAEFSFHEALTHLLHSHLLPEVFTLATLRQLPHCHPLF 399

|||||||||||||||||.|||||||||:||||||||||.||||||||||.

O.curzoniae 351 KWDWLLAKTWVRNAEFSVHEALTHLLHAHLLPEVFTLAMLRQLPHCHPLS 400

H.sapiens 400 KLLIPHTRYTLHINTLARELLIVPGQVVDRSTGIGIEGFSELIQRNMKQL 449

||||||.|||||||||||:|||.||||||:|||:||.||||||||||:||

O.curzoniae 401 KLLIPHMRYTLHINTLARQLLIAPGQVVDKSTGLGIRGFSELIQRNMEQL 450

H.sapiens 450 NYSLLCLPEDIRTRGVEDIPGYYYRDDGMQIWGAVERFVSEIIGIYYPSD 499

||:.||||:||::|||||||||||||||::||.||||||||::.||||||

O.curzoniae 451 NYATLCLPQDIQSRGVEDIPGYYYRDDGLRIWEAVERFVSEMVSIYYPSD 500

H.sapiens 500 ESVQDDRELQAWVREIFSKGFLNQESSGIPSSLETREALVQYVTMVIFTC 549

|||..|.|||||||||||:|||::||||.|||||||||||||||||||||

O.curzoniae 501 ESVWKDSELQAWVREIFSEGFLSRESSGFPSSLETREALVQYVTMVIFTC 550

H.sapiens 550 SAKHAAVSAGQFDSCAWMPNLPPSMQLPPPTSKGLATCEGFIATLPPVNA 599

||||:|||||||||||||||||.||||||||.||.|..||||||||||||

O.curzoniae 551 SAKHSAVSAGQFDSCAWMPNLPASMQLPPPTCKGQARPEGFIATLPPVNA 600

H.sapiens 600 TCDVILALWLLSKEPGDQRPLGTYPDEHFTEEAPRRSIATFQSRLAQISR 649

|||:|:|||||||||||:|||||||||||||||||||||.|||||||||.

O.curzoniae 601 TCDIIIALWLLSKEPGDRRPLGTYPDEHFTEEAPRRSIAAFQSRLAQISC 650

H.sapiens 650 GIQERNQGLVLPYTYLDPPLIENSVSI 676

.|:|||:||.|||.|||||||||||||

O.curzoniae 651 DIRERNRGLALPYAYLDPPLIENSVSI 677

Brown rat (*Rattus norvegicus*)

H.sapiens 1 MAEFRVRVSTGEAFGAGTWDKVSVSIVGTRGESPPLPLDNLGKEFTAGAE 50

||:||||||||||.|||||||||||||||.||||.:|||:|||||:||||

R.norvegicus 1 MAKFRVRVSTGEACGAGTWDKVSVSIVGTHGESPLVPLDHLGKEFSAGAE 50

H.sapiens 51 EDFQVTLPEDVGRVLLLRVHKAPPVLPLLGPL---APDAWFCRWFQLTPP 97

|||:||||:|||.||:||:|||||..|| || .||||:||||:|...

R.norvegicus 51 EDFEVTLPQDVGTVLMLRIHKAPPEAPL--PLLSFPPDAWYCRWFELEWL 98

H.sapiens 98 RGGHLLFPCYQWLEGAGTLVLQEGTAKVSWADHHPVLQQQRQEELQARQE 147

.|..|.|||||||||||.|||:||.|||||.|||..||.|||:||::|::

R.norvegicus 99 PGAALRFPCYQWLEGAGELVLREGAAKVSWQDHHRTLQDQRQKELESRKD 148

H.sapiens 148 MYQWKAYNPGWPHCLDEKTVEDLELNIKYSTAKNANFYLQAGSAFAEMKI 197

||.||.|..|||||||.:||:||:||||||..|||.|:.:|.|||.|:|.

R.norvegicus 149 MYSWKTYIEGWPHCLDHETVKDLDLNIKYSAMKNAKFFFKAQSAFTELKF 198

H.sapiens 198 KGLLDRKGLWRSLNEMKRIFNFRRTPAAEHAFEHWQEDAFFASQFLNGLN 247

||||||.||||||.||||:|||..|||||:.|.|||||||||||||||||

R.norvegicus 199 KGLLDRTGLWRSLREMKRMFNFHNTPAAEYVFAHWQEDAFFASQFLNGLN 248

H.sapiens 248 PVLIRRCHYLPKNFPVTDAMVASVLGPGTSLQAELEKGSLFLVDHGILSG 297

|||||||..||:||||||.|||.|||||||||||||||||||||||||||

R.norvegicus 249 PVLIRRCRRLPENFPVTDEMVAPVLGPGTSLQAELEKGSLFLVDHGILSG 298

H.sapiens 298 IQTNVINGKPQFSAAPMTLLYQSPGCGPLLPLAIQLSQTPGPNSPIFLPT 347

:||||||||||||||||||||||||.|||||:||||.|||||::|||||:

R.norvegicus 299 VQTNVINGKPQFSAAPMTLLYQSPGSGPLLPIAIQLKQTPGPDNPIFLPS 348

H.sapiens 348 DDKWDWLLAKTWVRNAEFSFHEALTHLLHSHLLPEVFTLATLRQLPHCHP 397

|||||||||||||||||||.|||||||||:||:||||.||||||||||||

R.norvegicus 349 DDKWDWLLAKTWVRNAEFSIHEALTHLLHAHLIPEVFALATLRQLPHCHP 398

H.sapiens 398 LFKLLIPHTRYTLHINTLARELLIVPGQVVDRSTGIGIEGFSELIQRNMK 447

||||||||||||||||||||||||.||:|||:|||:||.|||:||:|||:

R.norvegicus 399 LFKLLIPHTRYTLHINTLARELLIAPGKVVDKSTGLGIGGFSDLIKRNME 448

H.sapiens 448 QLNYSLLCLPEDIRTRGVEDIPGYYYRDDGMQIWGAVERFVSEIIGIYYP 497

||:||:||||||||.|.|.|:|||||||||||||.|:..|||||:.||||

R.norvegicus 449 QLSYSVLCLPEDIRARDVGDLPGYYYRDDGMQIWSAIRSFVSEIVDIYYP 498

H.sapiens 498 SDESVQDDRELQAWVREIFSKGFLNQESSGIPSSLETREALVQYVTMVIF 547

||.||:||:||||||.||||:|||:|||||:||.|:|:||||||||||||

R.norvegicus 499 SDASVRDDQELQAWVGEIFSEGFLSQESSGMPSLLDTQEALVQYVTMVIF 548

H.sapiens 548 TCSAKHAAVSAGQFDSCAWMPNLPPSMQLPPPTSKGLATCEGFIATLPPV 597

|||||||||||.|||||.||||||||||||||||||.|:.||||||||.|

R.norvegicus 549 TCSAKHAAVSASQFDSCVWMPNLPPSMQLPPPTSKGQASPEGFIATLPAV 598

H.sapiens 598 NATCDVILALWLLSKEPGDQRPLGTYPDEHFTEEAPRRSIATFQSRLAQI 647

|||||||:|||||||||||:||||.|||||||||.||||||.||.:|.||

R.norvegicus 599 NATCDVIIALWLLSKEPGDRRPLGHYPDEHFTEEVPRRSIAAFQRKLIQI 648

H.sapiens 648 SRGIQERNQGLVLPYTYLDPPLIENSVSI 676

|.||::|||.|.|||||||||||||||||

R.norvegicus 649 SSGIRKRNQSLALPYTYLDPPLIENSVSI 677

Black rat (*Rattus rattus*)

H.sapiens 1 MAEFRVRVSTGEAFGAGTWDKVSVSIVGTRGESPPLPLDNLGKEFTAGAE 50

||:||||||||||.|||||||||||||||.||||.:|||:|||||:||||

R.rattus 1 MAKFRVRVSTGEACGAGTWDKVSVSIVGTHGESPLVPLDHLGKEFSAGAE 50

H.sapiens 51 EDFQVTLPEDVGRVLLLRVHKAPPVLPLLGPL---APDAWFCRWFQLTPP 97

|||:||||:|||.||:||:|||||..|| || .||||:||||:|...

R.rattus 51 EDFEVTLPQDVGTVLMLRIHKAPPEAPL--PLLSFPPDAWYCRWFELEWL 98

H.sapiens 98 RGGHLLFPCYQWLEGAGTLVLQEGTAKVSWADHHPVLQQQRQEELQARQE 147

.|..|.|||||||||||.|||:||.|||||.|||..||.|||:||::|::

R.rattus 99 PGAALRFPCYQWLEGAGELVLREGAAKVSWQDHHRTLQDQRQKELESRKD 148

H.sapiens 148 MYQWKAYNPGWPHCLDEKTVEDLELNIKYSTAKNANFYLQAGSAFAEMKI 197

||.||.|..|||||||.:||:||:||||||..|||.|:.:|.|||.|:|.

R.rattus 149 MYSWKTYIEGWPHCLDHETVKDLDLNIKYSAMKNAKFFFKAQSAFTELKF 198

H.sapiens 198 KGLLDRKGLWRSLNEMKRIFNFRRTPAAEHAFEHWQEDAFFASQFLNGLN 247

||||||.||||||.||||:|||..|||||:.|.|||||||||||||||||

R.rattus 199 KGLLDRTGLWRSLREMKRMFNFHNTPAAEYVFAHWQEDAFFASQFLNGLN 248

H.sapiens 248 PVLIRRCHYLPKNFPVTDAMVASVLGPGTSLQAELEKGSLFLVDHGILSG 297

|||||||..||:||||||.|||.|||||||||||||||||||||||||||

R.rattus 249 PVLIRRCRRLPENFPVTDEMVAPVLGPGTSLQAELEKGSLFLVDHGILSG 298

H.sapiens 298 IQTNVINGKPQFSAAPMTLLYQSPGCGPLLPLAIQLSQTPGPNSPIFLPT 347

:||||||||||||||||||||||||.|||||:||||.|||||::|||||:

R.rattus 299 VQTNVINGKPQFSAAPMTLLYQSPGSGPLLPIAIQLKQTPGPDNPIFLPS 348

H.sapiens 348 DDKWDWLLAKTWVRNAEFSFHEALTHLLHSHLLPEVFTLATLRQLPHCHP 397

|||||||||||||||||||.|||||||||:||:||||.||||||||||||

R.rattus 349 DDKWDWLLAKTWVRNAEFSIHEALTHLLHAHLIPEVFALATLRQLPHCHP 398

H.sapiens 398 LFKLLIPHTRYTLHINTLARELLIVPGQVVDRSTGIGIEGFSELIQRNMK 447

||||||||||||||||||||:|||.||:|||:|||:||.|||:||:|||:

R.rattus 399 LFKLLIPHTRYTLHINTLARQLLIAPGKVVDKSTGLGIGGFSDLIKRNME 448

H.sapiens 448 QLNYSLLCLPEDIRTRGVEDIPGYYYRDDGMQIWGAVERFVSEIIGIYYP 497

||:||:||||||||.|.|.|||||||||||||||.|:..|||||:.||||

R.rattus 449 QLSYSVLCLPEDIRARDVGDIPGYYYRDDGMQIWSAIRSFVSEIVDIYYP 498

H.sapiens 498 SDESVQDDRELQAWVREIFSKGFLNQESSGIPSSLETREALVQYVTMVIF 547

||.||:||:||||||.||||:|||:|||||:||.|:|:||||||||||||

R.rattus 499 SDASVRDDQELQAWVGEIFSEGFLSQESSGMPSLLDTQEALVQYVTMVIF 548

H.sapiens 548 TCSAKHAAVSAGQFDSCAWMPNLPPSMQLPPPTSKGLATCEGFIATLPPV 597

|||||||||||.|||||.|||||||||||||.||||.|:.||||||||.|

R.rattus 549 TCSAKHAAVSASQFDSCVWMPNLPPSMQLPPSTSKGQASPEGFIATLPAV 598

H.sapiens 598 NATCDVILALWLLSKEPGDQRPLGTYPDEHFTEEAPRRSIATFQSRLAQI 647

|||||||:|||||||||||:||||.|||||||||||||||:.||.:|.||

R.rattus 599 NATCDVIIALWLLSKEPGDRRPLGHYPDEHFTEEAPRRSISAFQRKLIQI 648

H.sapiens 648 SRGIQERNQGLVLPYTYLDPPLIENSVSI 676

|.||::|||.|.|||||||||||||||||

R.rattus 649 SSGIRKRNQSLALPYTYLDPPLIENSVSI 677

African grass rat (*Arvicanthis niloticus*)

H.sapiens 1 MAEFRVRVSTGEAFGAGTWDKVSVSIVGTRGESPPLPLDNLGKEFTAGAE 50

||:||:|||||||.|||||||||||||||.||||.:|||:|||||.||||

A.niloticus 1 MAKFRLRVSTGEACGAGTWDKVSVSIVGTHGESPLVPLDHLGKEFNAGAE 50

H.sapiens 51 EDFQVTLPEDVGRVLLLRVHKAPPVLPL-LGPLAPDAWFCRWFQLTPPRG 99

|||:||||.|||.||:||||||||..|| |..|.||||||.||||....|

A.niloticus 51 EDFEVTLPHDVGAVLMLRVHKAPPEAPLPLLSLPPDAWFCCWFQLEWLPG 100

H.sapiens 100 GHLLFPCYQWLEGAGTLVLQEGTAKVSWADHHPVLQQQRQEELQARQEMY 149

..|.|||||||||||.|||:||.|||||.|||||||.|||:||:.||:||

A.niloticus 101 AALRFPCYQWLEGAGELVLREGAAKVSWQDHHPVLQDQRQKELETRQKMY 150

H.sapiens 150 QWKAYNPGWPHCLDEKTVEDLELNIKYSTAKNANFYLQAGSAFAEMKIKG 199

.||.|..|||||||.:||:||:||||||..|||.|:.:|.||.||:|.||

A.niloticus 151 SWKTYIEGWPHCLDHETVKDLDLNIKYSAMKNAKFFFKAHSAIAELKFKG 200

H.sapiens 200 LLDRKGLWRSLNEMKRIFNFRRTPAAEHAFEHWQEDAFFASQFLNGLNPV 249

||||.||||||.||:|:|||.:|||||:.|.|||||||||||||||||||

A.niloticus 201 LLDRTGLWRSLREMRRMFNFHKTPAAEYVFAHWQEDAFFASQFLNGLNPV 250

H.sapiens 250 LIRRCHYLPKNFPVTDAMVASVLGPGTSLQAELEKGSLFLVDHGILSGIQ 299

||||||.|||||||||.|||.|||||.|||||||||||||||||||||:|

A.niloticus 251 LIRRCHSLPKNFPVTDEMVAPVLGPGASLQAELEKGSLFLVDHGILSGVQ 300

H.sapiens 300 TNVINGKPQFSAAPMTLLYQSPGCGPLLPLAIQLSQTPGPNSPIFLPTDD 349

|||||||||||||||||||||.|.|||||:|:||.|||||::|||||:||

A.niloticus 301 TNVINGKPQFSAAPMTLLYQSSGSGPLLPIAVQLKQTPGPDNPIFLPSDD 350

H.sapiens 350 KWDWLLAKTWVRNAEFSFHEALTHLLHSHLLPEVFTLATLRQLPHCHPLF 399

|||||||||||||||||.|||:|||.|:||:||||.||||||||.|||||

A.niloticus 351 KWDWLLAKTWVRNAEFSIHEAVTHLFHAHLIPEVFALATLRQLPQCHPLF 400

H.sapiens 400 KLLIPHTRYTLHINTLARELLIVPGQVVDRSTGIGIEGFSELIQRNMKQL 449

||||||.||||:||||||||||.||:|||:|||:||.||||||:|||:||

A.niloticus 401 KLLIPHIRYTLYINTLARELLIAPGKVVDKSTGLGIGGFSELIKRNMEQL 450

H.sapiens 450 NYSLLCLPEDIRTRGVEDIPGYYYRDDGMQIWGAVERFVSEIIGIYYPSD 499

|||:||.|||||.|.:||||||||||||||||||::.|||||:.||||||

A.niloticus 451 NYSVLCFPEDIRARDMEDIPGYYYRDDGMQIWGAIKSFVSEIVSIYYPSD 500

H.sapiens 500 ESVQDDRELQAWVREIFSKGFLNQESSGIPSSLETREALVQYVTMVIFTC 549

.||:||:|||||||||||:|||::||||:||.|:|:||||||||||||||

A.niloticus 501 ASVRDDQELQAWVREIFSEGFLSRESSGMPSLLDTQEALVQYVTMVIFTC 550

H.sapiens 550 SAKHAAVSAGQFDSCAWMPNLPPSMQLPPPTSKGLATCEGFIATLPPVNA 599

|||||||||||||||||||||||:||||||||||.|..|.||||||.|||

A.niloticus 551 SAKHAAVSAGQFDSCAWMPNLPPTMQLPPPTSKGQARPESFIATLPAVNA 600

H.sapiens 600 TCDVILALWLLSKEPGDQRPLGTYPDEHFTEEAPRRSIATFQSRLAQISR 649

||..|:|||||||||||:||||.||||||||:|||||:|.||.:|.|||:

A.niloticus 601 TCYHIIALWLLSKEPGDRRPLGHYPDEHFTEDAPRRSVAAFQRKLIQISK 650

H.sapiens 650 GIQERNQGLVLPYTYLDPPLIENSVSI 676

||::|||.|.|||||||||||||||||

A.niloticus 651 GIRKRNQSLALPYTYLDPPLIENSVSI 677

Star-nosed mole (*Condylura cristata*)

H.sapiens 1 MAEFRVRVSTGEAFGAGTWDKVSVSIVGTRGESPPLPLDNLGKEFTAGAE 50

|.::||||:||...|:||||.||||:||..||:..|.|:|.||:|..|||

C.cristata 1 MGKYRVRVATGSYLGSGTWDNVSVSLVGPAGETAALRLNNFGKDFCRGAE 50

H.sapiens 51 EDFQVTLPEDVGRVLLLRVHKAPPVLPL-LGPLAPDAWFCRWFQLTPPRG 99

|||:||.|:||||||||||||.||.||. |||||||||||||||||||||

C.cristata 51 EDFEVTSPQDVGRVLLLRVHKTPPDLPRPLGPLAPDAWFCRWFQLTPPRG 100

H.sapiens 100 GHLLFPCYQWLEGAGTLVLQEGTAKVSWADHHPVLQQQRQEELQARQEMY 149

..|.|||||||.|:..|||:||.||:.|.|.||:|..|||.|::.||:.|

C.cristata 101 TPLRFPCYQWLGGSEGLVLREGAAKLIWQDSHPLLCAQRQAEIKDRQQKY 150

H.sapiens 150 QWKAYNPGWPHCLDEKTVEDLELNIKYSTAKNANFYLQAGSAFAEMKIKG 199

:|..:.||.|.|||.:.:::|:.|::||..|:.:...:..|....:|:||

C.cristata 151 RWSEFFPGIPRCLDAENLKELDPNLRYSVTKDISQKFRLLSPNIALKLKG 200

H.sapiens 200 LLDRKGLWRSLNEMKRIFNFRRTPAAEHAFEHWQEDAFFASQFLNGLNPV 249

|||.:..||||:|:||.|.|.::|.:|:.|||||||||||||||||||||

C.cristata 201 LLDSQRPWRSLDEIKRSFVFPKSPVSEYVFEHWQEDAFFASQFLNGLNPV 250

H.sapiens 250 LIRRCHYLPKNFPVTDAMVASVLGPGTSLQAELEKGSLFLVDHGILSGIQ 299

||||||.|||||||||||||.||||||.||||||||||:||||.||||::

C.cristata 251 LIRRCHCLPKNFPVTDAMVAPVLGPGTCLQAELEKGSLYLVDHAILSGLR 300

H.sapiens 300 TNVINGKPQFSAAPMTLLYQSPGCGPLLPLAIQLSQTPGPNSPIFLPTDD 349

.::|||:|||.|||:|||.|.|| ||||||||||:|||||:||||||:|.

C.cristata 301 PSIINGRPQFVAAPLTLLRQCPG-GPLLPLAIQLTQTPGPDSPIFLPSDA 349

H.sapiens 350 KWDWLLAKTWVRNAEFSFHEALTHLLHSHLLPEVFTLATLRQLPHCHPLF 399

..||||||||||::||..||.::|||.:|.:.|.|.|:||||||.|||:|

C.cristata 350 VEDWLLAKTWVRHSEFLVHEMVSHLLCTHFVMESFFLSTLRQLPMCHPIF 399

H.sapiens 400 KLLIPHTRYTLHINTLARELLIVPGQVVDRSTGIGIEGFSELIQRNMKQL 449

||||||.|||.|||.|||..|..||:::|:||.:|..|..|||.:.::.:

C.cristata 400 KLLIPHFRYTFHINILARTGLFAPGKLIDQSTSLGRVGSLELIAKGLEAV 449

H.sapiens 450 NYSLLCLPEDIRTRGVEDIPGYYYRDDGMQIWGAVERFVSEIIGIYYPSD 499

.|...|||..:..|.|:|:..|||||||:|||.|:|.|||:|:|||||.|

C.cristata 450 TYRSFCLPHQLADREVQDLTHYYYRDDGLQIWAAIESFVSDIVGIYYPDD 499

H.sapiens 500 ESVQDDRELQAWVREIFSKGFLNQESSGIPSSLETREALVQYVTMVIFTC 549

.:|..|.|||||..|||.:|||::..||:||:|.:..||::|:||:||.|

C.cristata 500 SAVSRDSELQAWAMEIFQEGFLSRVQSGVPSTLNSCAALIEYLTMIIFNC 549

H.sapiens 550 SAKHAAVSAGQFDSCAWMPNLPPSMQLPPPTSKGLATCEGFIATLPPVNA 599

||:||||::|||:..|||||:|.:|:|||||:||.| ..:|:||.|||

C.cristata 550 SAQHAAVNSGQFEFSAWMPNVPTTMRLPPPTAKGQA---DLMASLPEVNA 596

H.sapiens 600 TCDVILALWLLSKEPGDQRPLGTYPDEHFTEEAPRRSIATFQSRLAQISR 649

||..::..|.:|.:..|.||||||||||||||||:||||.|||||||:|.

C.cristata 597 TCHALVLFWGVSNDMKDTRPLGTYPDEHFTEEAPQRSIAAFQSRLAQVSG 646

H.sapiens 650 GIQERNQGLVLPYTYLDPPLIENSVSI 676

.|:||||||.||||||||.:::||:::

C.cristata 647 AIRERNQGLALPYTYLDPAVVDNSIAV 673

Cat (*Felis catus*)

H.sapiens 1 MAEFRVRVSTGEAFGAGTWDKVSVSIVGTRGESPPLPLDNLGKEFTAGAE 50

|||:.||||||||.|||||::::||||||.||:|||.||:.||||:.||.

F.catus 1 MAEYGVRVSTGEAIGAGTWNRIAVSIVGTLGETPPLRLDHFGKEFSKGAV 50

H.sapiens 51 EDFQVTLPEDVGRVLLLRVHKAPPVLPL-LGPLAPDAWFCRWFQLTPPRG 99

|||:|..|:|||||||||:||||.:||. :||||.|||||.|.|||||:|

F.catus 51 EDFRVASPQDVGRVLLLRLHKAPLLLPSPVGPLARDAWFCNWLQLTPPQG 100

H.sapiens 100 GHLLFPCYQWLEGAGTLVLQEGTAKVSWADHHPVLQQQRQEELQARQEMY 149

..|.||||||:||:|.|.|:||..||||||.||||||||:|||||||:.|

F.catus 101 APLRFPCYQWMEGSGILTLREGAGKVSWADDHPVLQQQRREELQARQDSY 150

H.sapiens 150 QWKAYNPGWPHCLDEKTVEDLELNIKYSTAKNANFYLQAGSAFAEMKIKG 199

|||.|.||||.|||||||:||:||||||..||.:|||:|||.|.||.:||

F.catus 151 QWKTYRPGWPRCLDEKTVKDLDLNIKYSVTKNVHFYLKAGSGFTEMTLKG 200

H.sapiens 200 LLDRKGLWRSLNEMKRIFNFRRTPAAEHAFEHWQEDAFFASQFLNGLNPV 249

||.|||.|:||:||||:|||.::||||:.:||||||||||||||||||||

F.catus 201 LLCRKGPWKSLHEMKRMFNFHKSPAAEYVYEHWQEDAFFASQFLNGLNPV 250

H.sapiens 250 LIRRCHYLPKNFPVTDAMVASVLGPGTSLQAELEKGSLFLVDHGILSGIQ 299

|||||..||:||||||.|||.|||||.|||||||:||||||||.||||::

F.catus 251 LIRRCRSLPENFPVTDDMVAPVLGPGASLQAELERGSLFLVDHAILSGVR 300

H.sapiens 300 TNVINGKPQFSAAPMTLLYQSPGCGPLLPLAIQLSQTPGPNSPIFLPTDD 349

||||||||||||||||||||:||.||||||||||||||||.||||||:|:

F.catus 301 TNVINGKPQFSAAPMTLLYQTPGGGPLLPLAIQLSQTPGPTSPIFLPSDN 350

H.sapiens 350 KWDWLLAKTWVRNAEFSFHEALTHLLHSHLLPEVFTLATLRQLPHCHPLF 399

|.|||||||||||:|||.||||||||.:|||.||||:|||||||||||||

F.catus 351 KRDWLLAKTWVRNSEFSVHEALTHLLQAHLLSEVFTMATLRQLPHCHPLF 400

H.sapiens 400 KLLIPHTRYTLHINTLARELLIVPGQVVDRSTGIGIEGFSELIQRNMKQL 449

||||||||||||||||||||||.|||||:||||||:.||||||||.|::|

F.catus 401 KLLIPHTRYTLHINTLARELLIAPGQVVERSTGIGLGGFSELIQRRMEEL 450

H.sapiens 450 NYSLLCLPEDIRTRGVEDIPGYYYRDDGMQIWGAVERFVSEIIGIYYPSD 499

:|:.||||||||.|||||||||||||||:|||||||.|||||:.||||||

F.catus 451 SYTALCLPEDIRARGVEDIPGYYYRDDGLQIWGAVESFVSEIVSIYYPSD 500

H.sapiens 500 ESVQDDRELQAWVREIFSKGFLNQESSGIPSSLETREALVQYVTMVIFTC 549

.||:||.|||||||||||:|||.:|.||:||||:|||:||:|.|||||.|

F.catus 501 ASVRDDSELQAWVREIFSEGFLGREDSGVPSSLDTRESLVRYATMVIFNC 550

H.sapiens 550 SAKHAAVSAGQFDSCAWMPNLPPSMQLPPPTSKGLATCEGFIATLPPVNA 599

||||.|||||||||..|||||||:||||||||||....||||||||.|||

F.catus 551 SAKHYAVSAGQFDSSIWMPNLPPTMQLPPPTSKGQTEPEGFIATLPAVNA 600

H.sapiens 600 TCDVILALWLLSKEPGDQRPLGTYPDEHFTEEAPRRSIATFQSRLAQISR 649

|||:|:.||||||:|||:|||||||:|||.||||||||..|||||||||:

F.catus 601 TCDIIVTLWLLSKDPGDRRPLGTYPEEHFIEEAPRRSITAFQSRLAQISK 650

H.sapiens 650 GIQERNQGLVLPYTYLDPPLIENSVSI 676

.||:||:.|.|||||||||||||||||

F.catus 651 DIQKRNKELALPYTYLDPPLIENSVSI 677

Striped hyena (*Hyaena hyaena*)

H.sapiens 1 MAEFRVRVSTGEAFGAGTWDKVSVSIVGTRGESPPLPLDNLGKEFTAGAE 50

|||:||.||||||.|||||::::|||||||||:||||||:.||||:.||.

H.hyaena 1 MAEYRVTVSTGEAIGAGTWNRIAVSIVGTRGETPPLPLDHFGKEFSKGAV 50

H.sapiens 51 EDFQVTLPEDVGRVLLLRVHKAPPVLPLLGPLAPDAWFCRWFQLTPPRGG 100

|||||..|:|||:||||::||.|.:...:||||.|||||.|.||:||||.

H.hyaena 51 EDFQVESPQDVGQVLLLQLHKDPLLPRAVGPLARDAWFCNWIQLSPPRGT 100

H.sapiens 101 HLLFPCYQWLEGAGTLVLQEGTAKVSWADHHPVLQQQRQEELQARQEMYQ 150

.|.||||||:||:..|.|:||.|:|||.|.|.:|||||:|||||:|:.|.

H.hyaena 101 PLRFPCYQWMEGSSRLALREGAARVSWGDDHSLLQQQRKEELQAKQDSYS 150

H.sapiens 151 WKAYNPGWPHCLDEKTVEDLELNIKYSTAKNANFYLQAGSAFAEMKIKGL 200

||.|.|||||||:||||.||:||||||.||||||||:|||.|.||..|||

H.hyaena 151 WKTYRPGWPHCLNEKTVRDLDLNIKYSIAKNANFYLKAGSGFTEMIFKGL 200

H.sapiens 201 LDRKGLWRSLNEMKRIFNFRRTPAAEHAFEHWQEDAFFASQFLNGLNPVL 250

|||||||:||:||||:||||:|||.|:.:||||||.||||||||||||||

H.hyaena 201 LDRKGLWKSLDEMKRMFNFRKTPAGEYVYEHWQEDTFFASQFLNGLNPVL 250

H.sapiens 251 IRRCHYLPKNFPVTDAMVASVLGPGTSLQAELEKGSLFLVDHGILSGIQT 300

||||..||:|||||:.|||.||||||||||||.:|||||||||||||:.|

H.hyaena 251 IRRCRSLPENFPVTNDMVAPVLGPGTSLQAELARGSLFLVDHGILSGVST 300

H.sapiens 301 NVINGKPQFSAAPMTLLYQSPGCGPLLPLAIQLSQTPGPNSPIFLPTDDK 350

|||||||||||||||||||.||.||||||||||||||||.||||||:|:|

H.hyaena 301 NVINGKPQFSAAPMTLLYQRPGGGPLLPLAIQLSQTPGPTSPIFLPSDNK 350

H.sapiens 351 WDWLLAKTWVRNAEFSFHEALTHLLHSHLLPEVFTLATLRQLPHCHPLFK 400

||||||||||||||||.:|||||||.:||||||||:|||||||.||||||

H.hyaena 351 WDWLLAKTWVRNAEFSVNEALTHLLEAHLLPEVFTMATLRQLPQCHPLFK 400

H.sapiens 401 LLIPHTRYTLHINTLARELLIVPGQVVDRSTGIGIEGFSELIQRNMKQLN 450

|||||||||||||||||||||.|||||:|||||||||||:||||.|:.|:

H.hyaena 401 LLIPHTRYTLHINTLARELLIAPGQVVERSTGIGIEGFSDLIQRRMETLS 450

H.sapiens 451 YSLLCLPEDIRTRGVEDIPGYYYRDDGMQIWGAVERFVSEIIGIYYPSDE 500

|:.||||.|||.||||.||||||||||:|:|||:|.||||||.||||||.

H.hyaena 451 YTSLCLPADIRARGVEGIPGYYYRDDGLQVWGAMESFVSEIISIYYPSDA 500

H.sapiens 501 SVQDDRELQAWVREIFSKGFLNQESSGIPSSLETREALVQYVTMVIFTCS 550

||:||.||||||.||||:|||.:||||:||||:||::||||||||||.||

H.hyaena 501 SVRDDSELQAWVWEIFSEGFLGRESSGVPSSLDTRKSLVQYVTMVIFNCS 550

H.sapiens 551 AKHAAVSAGQFDSCAWMPNLPPSMQLPPPTSKGLATCEGFIATLPPVNAT 600

|||.|:||||||||.|||||||:||||||||||....|||||.|||||||

H.hyaena 551 AKHYAISAGQFDSCVWMPNLPPTMQLPPPTSKGQTEPEGFIAALPPVNAT 600

H.sapiens 601 CDVILALWLLSKEPGDQRPLGTYPDEHFTEEAPRRSIATFQSRLAQISRG 650

||::..|||||:||||:|||||||||||||||||||||||||||||||:.

H.hyaena 601 CDIVFTLWLLSQEPGDRRPLGTYPDEHFTEEAPRRSIATFQSRLAQISKD 650

H.sapiens 651 IQERNQGLVLPYTYLDPPLIENSVSI 676

|::||..|.|||||||||||||||||

H.hyaena 651 IRKRNMELALPYTYLDPPLIENSVSI 676

Leopard (Panthera pardus)

H.sapiens 1 MAEFRVRVSTGEAFGAGTWDKVSVSIVGTRGESPPLPLDNLGKEFTAGAE 50

|||:.||||||||.|||||::::||||||.||:||||||:.||||:.||.

P.pardus 1 MAEYGVRVSTGEAIGAGTWNRIAVSIVGTLGETPPLPLDHFGKEFSKGAV 50

H.sapiens 51 EDFQVTLPEDVGRVLLLRVHKAPPVLPL-LGPLAPDAWFCRWFQLTPPRG 99

|||:|..|:|||||||||:||.|.:||. :||||.|||||.|.|||||:|

P.pardus 51 EDFRVASPQDVGRVLLLRLHKGPLLLPSPVGPLARDAWFCNWLQLTPPQG 100

H.sapiens 100 GHLLFPCYQWLEGAGTLVLQEGTAKVSWADHHPVLQQQRQEELQARQEMY 149

..|.||||||:||:|.|.|:||..||||||.||:|||||:|||||||:.|

P.pardus 101 APLRFPCYQWMEGSGILTLREGAGKVSWADDHPILQQQRREELQARQDSY 150

H.sapiens 150 QWKAYNPGWPHCLDEKTVEDLELNIKYSTAKNANFYLQAGSAFAEMKIKG 199

|||.|.||||.|||||||:||:||||||..||.:|||:|||.|.||.:||

P.pardus 151 QWKTYRPGWPRCLDEKTVKDLDLNIKYSVTKNVHFYLKAGSGFTEMTLKG 200

H.sapiens 200 LLDRKGLWRSLNEMKRIFNFRRTPAAEHAFEHWQEDAFFASQFLNGLNPV 249

||.|||||:||:||||:|||.::||||:.:||||||||||||||||||||

P.pardus 201 LLYRKGLWKSLHEMKRMFNFHKSPAAEYVYEHWQEDAFFASQFLNGLNPV 250

H.sapiens 250 LIRRCHYLPKNFPVTDAMVASVLGPGTSLQAELEKGSLFLVDHGILSGIQ 299

|||||..||:||||||.|||.|||||.|||||||:||||||||.||||::

P.pardus 251 LIRRCRSLPENFPVTDDMVAPVLGPGASLQAELERGSLFLVDHAILSGVR 300

H.sapiens 300 TNVINGKPQFSAAPMTLLYQSPGCGPLLPLAIQLSQTPGPNSPIFLPTDD 349

||||||||||||||||||||:||.||||||||||||||||.||||||:|:

P.pardus 301 TNVINGKPQFSAAPMTLLYQTPGGGPLLPLAIQLSQTPGPTSPIFLPSDN 350

H.sapiens 350 KWDWLLAKTWVRNAEFSFHEALTHLLHSHLLPEVFTLATLRQLPHCHPLF 399

|||||||||||||:|||.||||||||.:|||.||||:|||||||||||||

P.pardus 351 KWDWLLAKTWVRNSEFSVHEALTHLLQAHLLSEVFTMATLRQLPHCHPLF 400

H.sapiens 400 KLLIPHTRYTLHINTLARELLIVPGQVVDRSTGIGIEGFSELIQRNMKQL 449

||||||||||||||||||||||.|||||:||||||:.||||||||.|::|

P.pardus 401 KLLIPHTRYTLHINTLARELLIAPGQVVERSTGIGLGGFSELIQRCMEEL 450

H.sapiens 450 NYSLLCLPEDIRTRGVEDIPGYYYRDDGMQIWGAVERFVSEIIGIYYPSD 499

:|:.||||||||.|||||||||||||||:|||||||.|||||:.||||||

P.pardus 451 SYTALCLPEDIRARGVEDIPGYYYRDDGLQIWGAVESFVSEIVSIYYPSD 500

H.sapiens 500 ESVQDDRELQAWVREIFSKGFLNQESSGIPSSLETREALVQYVTMVIFTC 549

.||:||.|||||||||||:|||.:|.||:||||:|||:||.|.|||||.|

P.pardus 501 ASVRDDSELQAWVREIFSEGFLGREDSGVPSSLDTRESLVHYATMVIFNC 550

H.sapiens 550 SAKHAAVSAGQFDSCAWMPNLPPSMQLPPPTSKGLATCEGFIATLPPVNA 599

||||.|||||||||..|||||||:||||||||||....||||||||.|||

P.pardus 551 SAKHYAVSAGQFDSSIWMPNLPPTMQLPPPTSKGQTEPEGFIATLPAVNA 600

H.sapiens 600 TCDVILALWLLSKEPGDQRPLGTYPDEHFTEEAPRRSIATFQSRLAQISR 649

|||:|:.||||||||||:|||||||:|||.|||||||||.|||||||||:

P.pardus 601 TCDIIVTLWLLSKEPGDRRPLGTYPEEHFIEEAPRRSIAAFQSRLAQISK 650

H.sapiens 650 GIQERNQGLVLPYTYLDPPLIENSVSI 676

.||:||:.|.|||||||||||||||||

P.pardus 651 DIQKRNKELALPYTYLDPPLIENSVSI 677

Cheetah (*Acinonyx jubatus*)

H.sapiens 1 MAEFRVRVSTGEAFGAGTWDKVSVSIVGTRGESPPLPLDNLGKEFTAGAE 50

|||:.||||||||.|||||::::||||||.||:||||||:.||||:.||.

A.jubatus 1 MAEYGVRVSTGEAIGAGTWNRIAVSIVGTLGETPPLPLDHFGKEFSKGAV 50

H.sapiens 51 EDFQVTLPEDVGRVLLLRVHKAPPVLPL-LGPLAPDAWFCRWFQLTPPRG 99

|||:|..|:|||||||||:||||.:||. :||||.|||||.|.|||||:|

A.jubatus 51 EDFRVASPQDVGRVLLLRLHKAPLLLPSPVGPLARDAWFCNWLQLTPPQG 100

H.sapiens 100 GHLLFPCYQWLEGAGTLVLQEGTAKVSWADHHPVLQQQRQEELQARQEMY 149

..|.||||||:||:|.|.|:||..||||||.||||||||:|||||||:.|

A.jubatus 101 APLRFPCYQWMEGSGILTLREGAGKVSWADDHPVLQQQRREELQARQDSY 150

H.sapiens 150 QWKAYNPGWPHCLDEKTVEDLELNIKYSTAKNANFYLQAGSAFAEMKIKG 199

|||.|.||||.|||||||:||:||||||..||.:|||:|||.|.||.:||

A.jubatus 151 QWKTYRPGWPRCLDEKTVKDLDLNIKYSVTKNVHFYLKAGSGFTEMTLKG 200

H.sapiens 200 LLDRKGLWRSLNEMKRIFNFRRTPAAEHAFEHWQEDAFFASQFLNGLNPV 249

||.|||.|:||:||||:|||.::||||:.:||||||||||||||||||||

A.jubatus 201 LLCRKGPWKSLHEMKRMFNFHKSPAAEYVYEHWQEDAFFASQFLNGLNPV 250

H.sapiens 250 LIRRCHYLPKNFPVTDAMVASVLGPGTSLQAELEKGSLFLVDHGILSGIQ 299

|||||..||:||||||.|||.|||||.|||||||:||||||||.||||::

A.jubatus 251 LIRRCRSLPENFPVTDDMVAPVLGPGASLQAELERGSLFLVDHAILSGVR 300

H.sapiens 300 TNVINGKPQFSAAPMTLLYQSPGCGPLLPLAIQLSQTPGPNSPIFLPTDD 349

||||||||||||||||||||.||.||||||||||||||||.||||||:|.

A.jubatus 301 TNVINGKPQFSAAPMTLLYQRPGGGPLLPLAIQLSQTPGPTSPIFLPSDS 350

H.sapiens 350 KWDWLLAKTWVRNAEFSFHEALTHLLHSHLLPEVFTLATLRQLPHCHPLF 399

|||||||||||||:|||.||||||||.:|||.||||:|||||||||||||

A.jubatus 351 KWDWLLAKTWVRNSEFSVHEALTHLLQAHLLSEVFTMATLRQLPHCHPLF 400

H.sapiens 400 KLLIPHTRYTLHINTLARELLIVPGQVVDRSTGIGIEGFSELIQRNMKQL 449

||||||||||||||||||||||..||||:||||||:.||||||||.|::|

A.jubatus 401 KLLIPHTRYTLHINTLARELLIALGQVVERSTGIGLGGFSELIQRRMEEL 450

H.sapiens 450 NYSLLCLPEDIRTRGVEDIPGYYYRDDGMQIWGAVERFVSEIIGIYYPSD 499

:|:.||||||||.|||||||||||||||:|||||||.|||||:.||||||

A.jubatus 451 SYTALCLPEDIRARGVEDIPGYYYRDDGLQIWGAVESFVSEIVSIYYPSD 500

H.sapiens 500 ESVQDDRELQAWVREIFSKGFLNQESSGIPSSLETREALVQYVTMVIFTC 549

.||:||.|||||||||||:|||.:|.||:||||:|||:||.|.|||||.|

A.jubatus 501 TSVRDDSELQAWVREIFSEGFLGREDSGVPSSLDTRESLVHYATMVIFNC 550

H.sapiens 550 SAKHAAVSAGQFDSCAWMPNLPPSMQLPPPTSKGLATCEGFIATLPPVNA 599

||||.|||||||||..|||||||:||||||||||....||||||||.|||

A.jubatus 551 SAKHYAVSAGQFDSSIWMPNLPPTMQLPPPTSKGQTEPEGFIATLPAVNA 600

H.sapiens 600 TCDVILALWLLSKEPGDQRPLGTYPDEHFTEEAPRRSIATFQSRLAQISR 649

|||:|:.||||||||||:|||||||:|||.|||||||||.|||||||||:

A.jubatus 601 TCDIIVTLWLLSKEPGDRRPLGTYPEEHFIEEAPRRSIAAFQSRLAQISK 650

H.sapiens 650 GIQERNQGLVLPYTYLDPPLIENSVSI 676

.||:||:.|.|||||||||||||||||

A.jubatus 651 DIQKRNKELALPYTYLDPPLIENSVSI 677

Giant panda (*Ailuropoda melanoleuca*)

H.sapiens 1 MAEFRVRVSTGEAFGAGTWDKVSVSIVGTRGESPPLPLDNLGKEFTAGAE 50

||::.||||||||.|||||:|:::|:||||||:|||.||:|||||:|||.

A.melanoleuca 1 MAKYSVRVSTGEAIGAGTWNKIAISVVGTRGETPPLRLDHLGKEFSAGAV 50

H.sapiens 51 EDFQVTLPEDVGRVLLLRVHKAPPVLPL-LGPLAPDAWFCRWFQLTPPRG 99

|:|:|..|:|||.||||||||||.:||. :|||..|||||||||||||||

A.melanoleuca 51 EEFEVESPQDVGEVLLLRVHKAPLLLPAPIGPLGRDAWFCRWFQLTPPRG 100

H.sapiens 100 GHLLFPCYQWLEGAGTLVLQEGTAKVSWADHHPVLQQQRQEELQARQEMY 149

..|.|||||||:|.|:|.|:.|.||||.||.||.|||||||||||||:.|

A.melanoleuca 101 VPLRFPCYQWLDGEGSLALRAGPAKVSGADDHPTLQQQRQEELQARQDSY 150

H.sapiens 150 QWKAYNPGWPHCLDEKTVEDLELNIKYSTAKNANFYLQAGSAFAEMKIKG 199

.||.|:||||.||:||:|:||:||||||..||:.|:|:..|||.|:|:||

A.melanoleuca 151 HWKTYSPGWPRCLNEKSVKDLDLNIKYSVMKNSCFFLKVSSAFTELKLKG 200

H.sapiens 200 LLDRKGLWRSLNEMKRIFNFRRTPAAEHAFEHWQEDAFFASQFLNGLNPV 249

||||||.|.||:||:.:.|||:|||||:..||||||||||||||||.|||

A.melanoleuca 201 LLDRKGPWESLDEMQTMLNFRKTPAAEYVVEHWQEDAFFASQFLNGHNPV 250

H.sapiens 250 LIRRCHYLPKNFPVTDAMVASVLGPGTSLQAELEKGSLFLVDHGILSGIQ 299

|||||.:||||||||:.|||.||||.||||||||:||:|||||||||||.

A.melanoleuca 251 LIRRCRHLPKNFPVTEDMVAPVLGPETSLQAELERGSVFLVDHGILSGIH 300

H.sapiens 300 TNVINGKPQFSAAPMTLLYQSPGCGPLLPLAIQLSQTPGPNSPIFLPTDD 349

.||:||:|||||||||||||.|..||||||||||||||||:||||||.|:

A.melanoleuca 301 GNVVNGRPQFSAAPMTLLYQRPRGGPLLPLAIQLSQTPGPDSPIFLPIDN 350

H.sapiens 350 KWDWLLAKTWVRNAEFSFHEALTHLLHSHLLPEVFTLATLRQLPHCHPLF 399

|||||||||||||||||.|||||||||:||||||||:|||||||.|||||

A.melanoleuca 351 KWDWLLAKTWVRNAEFSVHEALTHLLHAHLLPEVFTMATLRQLPQCHPLF 400

H.sapiens 400 KLLIPHTRYTLHINTLARELLIVPGQVVDRSTGIGIEGFSELIQRNMKQL 449

|||:||||||||||||||||||.||:|||||||||||||||||||||:||

A.melanoleuca 401 KLLVPHTRYTLHINTLARELLIAPGKVVDRSTGIGIEGFSELIQRNMEQL 450

H.sapiens 450 NYSLLCLPEDIRTRGVEDIPGYYYRDDGMQIWGAVERFVSEIIGIYYPSD 499

|||:||||||||.|||||||.|||||||:|||||||.||||||..||.||

A.melanoleuca 451 NYSVLCLPEDIRARGVEDIPNYYYRDDGLQIWGAVESFVSEIINTYYLSD 500

H.sapiens 500 ESVQDDRELQAWVREIFSKGFLNQESSGIPSSLETREALVQYVTMVIFTC 549

.||:||.|||||||||||:|||.::|||:||:||||||||||:|||||.|

A.melanoleuca 501 VSVRDDGELQAWVREIFSEGFLGRKSSGMPSALETREALVQYITMVIFNC 550

H.sapiens 550 SAKHAAVSAGQFDSCAWMPNLPPSMQLPPPTSKGLATCEGFIATLPPVNA 599

||||.||||||||||.||||||||||||||||||....|||:||||||||

A.melanoleuca 551 SAKHYAVSAGQFDSCVWMPNLPPSMQLPPPTSKGQTKPEGFLATLPPVNA 600

H.sapiens 600 TCDVILALWLLSKEPGDQRPLGTYPDEHFTEEAPRRSIATFQSRLAQISR 649

|||:|:||||||||||||||||||||:|||||||::|||.|||||||||:

A.melanoleuca 601 TCDIIIALWLLSKEPGDQRPLGTYPDKHFTEEAPQQSIAAFQSRLAQISK 650

H.sapiens 650 GIQERNQGLVLPYTYLDPPLIENSVSI 676

.|:||||||.|||||||||||||||||

A.melanoleuca 651 EIRERNQGLPLPYTYLDPPLIENSVSI 677

Dog (*Canis lupus familiaris*)

H.sapiens 1 MAEFRVRVSTGEAFGAGTWDKVSVSIVGTRGESPPLPLDNLGKEFTAGAE 50

||||.|.||||||.|||||:|::||:||||||:|||.||:.||||:|||.

C.lupus fam. 1 MAEFSVTVSTGEAIGAGTWNKIAVSLVGTRGETPPLRLDHPGKEFSAGAV 50

H.sapiens 51 EDFQVTLPEDVGRVLLLRVHKAPPVLPL-LGPLAPDAWFCRWFQLTPPRG 99

|||||..|:|||.:|||||||||.:||. :||:|.|||||||||||||:|

C.lupus fam. 51 EDFQVASPQDVGPLLLLRVHKAPLLLPAPVGPVARDAWFCRWFQLTPPQG 100

H.sapiens 100 GHLLFPCYQWLEGAGTLVLQEGTAKVSWADHHPVLQQQRQEELQARQEMY 149

..|.|||||||||||:|.|:.|.|||.|||.||:||||||:||:|||:.|

C.lupus fam. 101 APLRFPCYQWLEGAGSLALRAGAAKVPWADDHPILQQQRQDELKARQDSY 150

H.sapiens 150 QWKAYNPGWPHCLDEKTVEDLELNIKYSTAKNANFYLQAGSAFAEMKIKG 199

.||.|:||||||||||:|:||:||||||..||:.|||:..||..|:|:||

C.lupus fam. 151 HWKTYSPGWPHCLDEKSVKDLDLNIKYSVMKNSCFYLKVSSAITELKLKG 200

H.sapiens 200 LLDRKGLWRSLNEMKRIFNFRRTPAAEHAFEHWQEDAFFASQFLNGLNPV 249

||||||.|:|||||||:||||:|||||:..||||||.|||||||||||||

C.lupus fam. 201 LLDRKGPWKSLNEMKRMFNFRKTPAAEYTLEHWQEDTFFASQFLNGLNPV 250

H.sapiens 250 LIRRCHYLPKNFPVTDAMVASVLGPGTSLQAELEKGSLFLVDHGILSGIQ 299

|||||.:||:||||||.|||.|||||||||||||:|||||||||||.|::

C.lupus fam. 251 LIRRCGHLPENFPVTDDMVAPVLGPGTSLQAELERGSLFLVDHGILCGVR 300

H.sapiens 300 TNVINGKPQFSAAPMTLLYQSPGCGPLLPLAIQLSQTPGPNSPIFLPTDD 349

||:||||||||||||||||||||.||||||||||||||||.||||||:|:

C.lupus fam. 301 TNLINGKPQFSAAPMTLLYQSPGGGPLLPLAIQLSQTPGPASPIFLPSDN 350

H.sapiens 350 KWDWLLAKTWVRNAEFSFHEALTHLLHSHLLPEVFTLATLRQLPHCHPLF 399

|||||||||||||||||.|||:|||||:||||||||:|||||||||||||

C.lupus fam. 351 KWDWLLAKTWVRNAEFSVHEAVTHLLHAHLLPEVFTMATLRQLPHCHPLF 400

H.sapiens 400 KLLIPHTRYTLHINTLARELLIVPGQVVDRSTGIGIEGFSELIQRNMKQL 449

||||||||||||||||||||||.|||||||||||||.||||||||||:||

C.lupus fam. 401 KLLIPHTRYTLHINTLARELLIAPGQVVDRSTGIGIGGFSELIQRNMEQL 450

H.sapiens 450 NYSLLCLPEDIRTRGVEDIPGYYYRDDGMQIWGAVERFVSEIIGIYYPSD 499

|||:|||||||:.|||:|||.|||||||::||||||.||||||.||||||

C.lupus fam. 451 NYSVLCLPEDIQARGVKDIPNYYYRDDGLKIWGAVESFVSEIINIYYPSD 500

H.sapiens 500 ESVQDDRELQAWVREIFSKGFLNQESSGIPSSLETREALVQYVTMVIFTC 549

.||.:|.||||||.||||:|||.:||||:|.|||||||||||||||||.|

C.lupus fam. 501 ASVCEDGELQAWVWEIFSEGFLGRESSGMPFSLETREALVQYVTMVIFNC 550

H.sapiens 550 SAKHAAVSAGQFDSCAWMPNLPPSMQLPPPTSKGLATCEGFIATLPPVNA 599

||||.||||||||||.||||||.|||||||||||....|||:||||||||

C.lupus fam. 551 SAKHYAVSAGQFDSCVWMPNLPASMQLPPPTSKGQTRPEGFLATLPPVNA 600

H.sapiens 600 TCDVILALWLLSKEPGDQRPLGTYPDEHFTEEAPRRSIATFQSRLAQISR 649

|||:|:|||||||||||||||||||:||||||||::|||||||||.|||:

C.lupus fam. 601 TCDIIVALWLLSKEPGDQRPLGTYPEEHFTEEAPQQSIATFQSRLTQISK 650

H.sapiens 650 GIQERNQGLVLPYTYLDPPLIENSVSI 676

.|:||||.|.|||||||||||||||||

C.lupus fam. 651 EIRERNQELALPYTYLDPPLIENSVSI 677

Red fox (*Vulpes vulpes*)

H.sapiens 1 MAEFRVRVSTGEAFGAGTWDKVSVSIVGTRGESPPLPLDNLGKEFTAGAE 50

||||.|.||||:|.|||||:|::||:||||||:|||.||:.||||:|||.

V.vulpes 1 MAEFSVTVSTGDAIGAGTWNKIAVSLVGTRGETPPLRLDHPGKEFSAGAV 50

H.sapiens 51 EDFQVTLPEDVGRVLLLRVHKAPPVLPL-LGPLAPDAWFCRWFQLTPPRG 99

|||||..|:|||.:|||||||||.:||. :||:|.|||||||||||||:|

V.vulpes 51 EDFQVASPQDVGPLLLLRVHKAPLLLPAPVGPVARDAWFCRWFQLTPPQG 100

H.sapiens 100 GHLLFPCYQWLEGAGTLVLQEGTAKVSWADHHPVLQQQRQEELQARQEMY 149

..|.|||||||||||:|.|:.|.|||.|||.||:||||||:||:|||:.|

V.vulpes 101 APLRFPCYQWLEGAGSLALRAGAAKVPWADDHPILQQQRQDELKARQDSY 150

H.sapiens 150 QWKAYNPGWPHCLDEKTVEDLELNIKYSTAKNANFYLQAGSAFAEMKIKG 199

.||.|:||||||||||:|:||:||||||..||:.|||:..||..|:|:||

V.vulpes 151 HWKTYSPGWPHCLDEKSVKDLDLNIKYSVMKNSCFYLKVSSAITELKLKG 200

H.sapiens 200 LLDRKGLWRSLNEMKRIFNFRRTPAAEHAFEHWQEDAFFASQFLNGLNPV 249

||||||.|:||:||||:||||:|||||:..||||||.|||||||||||||

V.vulpes 201 LLDRKGPWKSLDEMKRMFNFRKTPAAEYTLEHWQEDTFFASQFLNGLNPV 250

H.sapiens 250 LIRRCHYLPKNFPVTDAMVASVLGPGTSLQAELEKGSLFLVDHGILSGIQ 299

|||||.:||:||||||.|||.|||||||||||||:|||||||||||||:.

V.vulpes 251 LIRRCGHLPENFPVTDDMVAPVLGPGTSLQAELERGSLFLVDHGILSGVH 300

H.sapiens 300 TNVINGKPQFSAAPMTLLYQSPGCGPLLPLAIQLSQTPGPNSPIFLPTDD 349

||:||||||||||||||||||||.||||||||||||||||.||||||:|:

V.vulpes 301 TNLINGKPQFSAAPMTLLYQSPGGGPLLPLAIQLSQTPGPASPIFLPSDN 350

H.sapiens 350 KWDWLLAKTWVRNAEFSFHEALTHLLHSHLLPEVFTLATLRQLPHCHPLF 399

|||||||||||||||||..||:|||||.||||||||:|||||||||||||

V.vulpes 351 KWDWLLAKTWVRNAEFSVQEAVTHLLHGHLLPEVFTMATLRQLPHCHPLF 400

H.sapiens 400 KLLIPHTRYTLHINTLARELLIVPGQVVDRSTGIGIEGFSELIQRNMKQL 449

||||||||||||||||||||||.|||||||||||||.||||||||||:||

V.vulpes 401 KLLIPHTRYTLHINTLARELLIAPGQVVDRSTGIGIGGFSELIQRNMEQL 450

H.sapiens 450 NYSLLCLPEDIRTRGVEDIPGYYYRDDGMQIWGAVERFVSEIIGIYYPSD 499

|||:|||||||:.|||:|||.|||||||::||||||.||||||.||||||

V.vulpes 451 NYSVLCLPEDIQARGVKDIPNYYYRDDGLKIWGAVESFVSEIINIYYPSD 500

H.sapiens 500 ESVQDDRELQAWVREIFSKGFLNQESSGIPSSLETREALVQYVTMVIFTC 549

.||::|.||||||.||||:|||.:||||:||||||.||||||||||||.|

V.vulpes 501 ASVREDGELQAWVWEIFSEGFLGRESSGMPSSLETLEALVQYVTMVIFNC 550

H.sapiens 550 SAKHAAVSAGQFDSCAWMPNLPPSMQLPPPTSKGLATCEGFIATLPPVNA 599

||||.||||||||||.||||||.|||||||||||....|||:||||||||

V.vulpes 551 SAKHYAVSAGQFDSCVWMPNLPASMQLPPPTSKGQTRPEGFLATLPPVNA 600

H.sapiens 600 TCDVILALWLLSKEPGDQRPLGTYPDEHFTEEAPRRSIATFQSRLAQISR 649

|||:|:|||||||||||||||||||:||||||||::|||||||.|.|||:

V.vulpes 601 TCDIIVALWLLSKEPGDQRPLGTYPEEHFTEEAPQQSIATFQSHLTQISK 650

H.sapiens 650 GIQERNQGLVLPYTYLDPPLIENSVSI 676

.|:||||.|.|||||||||||||||||

V.vulpes 651 EIRERNQELALPYTYLDPPLIENSVSI 677

Northern fur seal (*Callorhinus ursinus*)

H.sapiens 1 MAEFRVRVSTGEAFGAGTWDKVSVSIVGTRGESPPLPLDNLGKEFTAGAE 50

||:|.||||||||.|||||:|:::||||||||:|||.||:.||||:|||.

C.ursinus 1 MAKFSVRVSTGEAIGAGTWNKIAISIVGTRGETPPLRLDHAGKEFSAGAV 50

H.sapiens 51 EDFQVTLPEDVGRVLLLRVHKAPPVLPL-LGPLAPDAWFCRWFQLTPPRG 99

||||:..|:|||.||||||||||.:||: :|.:|.|||||||||||||:|

C.ursinus 51 EDFQLEFPQDVGAVLLLRVHKAPLLLPVPVGAVARDAWFCRWFQLTPPQG 100

H.sapiens 100 GHLLFPCYQWLEGAGTLVLQEGTAKVSWADHHPVLQQQRQEELQARQEMY 149

..|.|||||||||||:|.|:.|.|||||||.||:|:|||||||||||:.|

C.ursinus 101 APLRFPCYQWLEGAGSLALRAGAAKVSWADDHPILRQQRQEELQARQDSY 150

H.sapiens 150 QWKAYNPGWPHCLDEKTVEDLELNIKYSTAKNANFYLQAGSAFAEMKIKG 199

.||.|:||||||||||:|:||:||||||..||:.|||:..|||.|:|:||

C.ursinus 151 HWKTYSPGWPHCLDEKSVKDLDLNIKYSVMKNSCFYLKVSSAFMELKLKG 200

H.sapiens 200 LLDRKGLWRSLNEMKRIFNFRRTPAAEHAFEHWQEDAFFASQFLNGLNPV 249

||||||.|:|||||:|:.|||:|||||:.|:|||||||||||||||||||

C.ursinus 201 LLDRKGPWKSLNEMRRMLNFRKTPAAEYVFKHWQEDAFFASQFLNGLNPV 250

H.sapiens 250 LIRRCHYLPKNFPVTDAMVASVLGPGTSLQAELEKGSLFLVDHGILSGIQ 299

||.||.:||:||||||.|||.|||||||||||||:||||||||||||||:

C.ursinus 251 LICRCRHLPENFPVTDDMVAPVLGPGTSLQAELERGSLFLVDHGILSGIR 300

H.sapiens 300 TNVINGKPQFSAAPMTLLYQSPGCGPLLPLAIQLSQTPGPNSPIFLPTDD 349

|||:||||||||||||||:|.||.||||||||||||||||:||||||:|:

C.ursinus 301 TNVVNGKPQFSAAPMTLLFQRPGGGPLLPLAIQLSQTPGPDSPIFLPSDN 350

H.sapiens 350 KWDWLLAKTWVRNAEFSFHEALTHLLHSHLLPEVFTLATLRQLPHCHPLF 399

|||||||||||||||||.|||:|||||:||||||||||||.|||||||||

C.ursinus 351 KWDWLLAKTWVRNAEFSVHEAVTHLLHTHLLPEVFTLATLHQLPHCHPLF 400

H.sapiens 400 KLLIPHTRYTLHINTLARELLIVPGQVVDRSTGIGIEGFSELIQRNMKQL 449

||||||||||||||||||||||.|||||||||||||.||||||||||:||

C.ursinus 401 KLLIPHTRYTLHINTLARELLIAPGQVVDRSTGIGIGGFSELIQRNMEQL 450

H.sapiens 450 NYSLLCLPEDIRTRGVEDIPGYYYRDDGMQIWGAVERFVSEIIGIYYPSD 499

:||:||||||||.|||||||.|||||||:|||||||.||||||.||||||

C.ursinus 451 SYSVLCLPEDIRARGVEDIPNYYYRDDGLQIWGAVESFVSEIINIYYPSD 500

H.sapiens 500 ESVQDDRELQAWVREIFSKGFLNQESSGIPSSLETREALVQYVTMVIFTC 549

.||.||.||||||.||||:|||.:||||:||:||||||||||||||||.|

C.ursinus 501 ASVHDDNELQAWVWEIFSEGFLGRESSGVPSALETREALVQYVTMVIFNC 550

H.sapiens 550 SAKHAAVSAGQFDSCAWMPNLPPSMQLPPPTSKGLATCEGFIATLPPVNA 599

||||.||||||||||.|||||||:||||||||||....|||:||||||||

C.ursinus 551 SAKHYAVSAGQFDSCVWMPNLPPTMQLPPPTSKGQTRPEGFLATLPPVNA 600

H.sapiens 600 TCDVILALWLLSKEPGDQRPLGTYPDEHFTEEAPRRSIATFQSRLAQISR 649

|||:|:|||:|||||||||||||||:|||.||||::||..|||||||||:

C.ursinus 601 TCDIIIALWILSKEPGDQRPLGTYPEEHFMEEAPQQSITAFQSRLAQISK 650

H.sapiens 650 GIQERNQGLVLPYTYLDPPLIENSVSI 676

.|:||||.|.|||||||||||||||||

C.ursinus 651 DIRERNQTLSLPYTYLDPPLIENSVSI 677

California sea lion (*Zalophus californianus*)

H.sapiens 1 MAEFRVRVSTGEAFGAGTWDKVSVSIVGTRGESPPLPLDNLGKEFTAGAE 50

||:|.||||||||.|||||:|:::||||||||:|||.||:.||||:|||.

Z.californian 1 MAKFSVRVSTGEALGAGTWNKIAISIVGTRGETPPLRLDHAGKEFSAGAV 50

H.sapiens 51 EDFQVTLPEDVGRVLLLRVHKAPPVLPL-LGPLAPDAWFCRWFQLTPPRG 99

||||:..|:|||.||||||||||.:||: :|.:|.|||||||||||||||

Z.californianus 51 EDFQLEFPQDVGAVLLLRVHKAPLLLPVPVGAVARDAWFCRWFQLTPPRG 100

H.sapiens 100 GHLLFPCYQWLEGAGTLVLQEGTAKVSWADHHPVLQQQRQEELQARQEMY 149

..|.|||||||||||:|.|:.|.|||||||.||:|:|||||||||||:.|

Z.californianus 101 APLRFPCYQWLEGAGSLALRAGAAKVSWADDHPILRQQRQEELQARQDSY 150

H.sapiens 150 QWKAYNPGWPHCLDEKTVEDLELNIKYSTAKNANFYLQAGSAFAEMKIKG 199

.||.|:||||||||||:|:||:||||||..||:.|||:..|||.|:|:||

Z.californianus 151 HWKTYSPGWPHCLDEKSVKDLDLNIKYSVMKNSCFYLKVSSAFMELKLKG 200

H.sapiens 200 LLDRKGLWRSLNEMKRIFNFRRTPAAEHAFEHWQEDAFFASQFLNGLNPV 249

||||||.|:|||||:|:.|||:|||||:.|:|||||||||||||||||||

Z.californianus 201 LLDRKGPWKSLNEMRRMLNFRKTPAAEYVFKHWQEDAFFASQFLNGLNPV 250

H.sapiens 250 LIRRCHYLPKNFPVTDAMVASVLGPGTSLQAELEKGSLFLVDHGILSGIQ 299

|||||.:||:||||||.|||.|||||||||||||:||||||||||||||:

Z.californianus 251 LIRRCRHLPENFPVTDDMVAPVLGPGTSLQAELERGSLFLVDHGILSGIR 300

H.sapiens 300 TNVINGKPQFSAAPMTLLYQSPGCGPLLPLAIQLSQTPGPNSPIFLPTDD 349

|||:||||||||||||||:|.||.|||||||||||||||||||||||:|:

Z.californianus 301 TNVVNGKPQFSAAPMTLLFQRPGGGPLLPLAIQLSQTPGPNSPIFLPSDN 350

H.sapiens 350 KWDWLLAKTWVRNAEFSFHEALTHLLHSHLLPEVFTLATLRQLPHCHPLF 399

:||||||||||||||||.|||:|||||:||||||||||||.|||||||||

Z.californianus 351 RWDWLLAKTWVRNAEFSVHEAVTHLLHTHLLPEVFTLATLHQLPHCHPLF 400

H.sapiens 400 KLLIPHTRYTLHINTLARELLIVPGQVVDRSTGIGIEGFSELIQRNMKQL 449

||||||||||||||||||||||.|||||||||||||.||||||||||:||

Z.californianus 401 KLLIPHTRYTLHINTLARELLIAPGQVVDRSTGIGIGGFSELIQRNMEQL 450

H.sapiens 450 NYSLLCLPEDIRTRGVEDIPGYYYRDDGMQIWGAVERFVSEIIGIYYPSD 499

:||:||||||||.|||||||.|||||||:|||||||.||||||.||||||

Z.californianus 451 SYSVLCLPEDIRARGVEDIPNYYYRDDGLQIWGAVESFVSEIINIYYPSD 500

H.sapiens 500 ESVQDDRELQAWVREIFSKGFLNQESSGIPSSLETREALVQYVTMVIFTC 549

.||.||.||||||.||||:|||.:||||:||:||||||||||||||||.|

Z.californianus 501 ASVHDDNELQAWVWEIFSEGFLGRESSGVPSALETREALVQYVTMVIFNC 550

H.sapiens 550 SAKHAAVSAGQFDSCAWMPNLPPSMQLPPPTSKGLATCEGFIATLPPVNA 599

||||.||||||||||.|||||||:||||||||||....|||:||||||||

Z.californianus 551 SAKHYAVSAGQFDSCVWMPNLPPTMQLPPPTSKGQTRPEGFLATLPPVNA 600

H.sapiens 600 TCDVILALWLLSKEPGDQRPLGTYPDEHFTEEAPRRSIATFQSRLAQISR 649

|||:|:|||:|||||||||||||||:|||.||||::||..|||||||||:

Z.californianus 601 TCDIIIALWILSKEPGDQRPLGTYPEEHFMEEAPQQSITAFQSRLAQISK 650

H.sapiens 650 GIQERNQGLVLPYTYLDPPLIENSVSI 676

.|:||||.|.|||||||||||||||||

Z.californianus 651 DIRERNQRLSLPYTYLDPPLIENSVSI 677

Steller sea lion (*Eumetopias jubatus*)

H.sapiens 1 MAEFRVRVSTGEAFGAGTWDKVSVSIVGTRGESPPLPLDNLGKEFTAGAE 50

||:|.||||||||.|||||:|:::||||||||:|||.||:.||||:|||.

E.jubatus 1 MAKFSVRVSTGEALGAGTWNKIAISIVGTRGETPPLRLDHAGKEFSAGAV 50

H.sapiens 51 EDFQVTLPEDVGRVLLLRVHKAPPVLPL-LGPLAPDAWFCRWFQLTPPRG 99

||||:..|:|||.||||||||||.:||: :|.:|.|||||||||||||||

E.jubatus 51 EDFQLAFPQDVGAVLLLRVHKAPLLLPVPVGAVARDAWFCRWFQLTPPRG 100

H.sapiens 100 GHLLFPCYQWLEGAGTLVLQEGTAKVSWADHHPVLQQQRQEELQARQEMY 149

..|.|||||||||||:|.|:.|.|||||||.||:|:|||||||||||:.|

E.jubatus 101 APLRFPCYQWLEGAGSLALRAGAAKVSWADDHPILRQQRQEELQARQDSY 150

H.sapiens 150 QWKAYNPGWPHCLDEKTVEDLELNIKYSTAKNANFYLQAGSAFAEMKIKG 199

.||.|:||||||||||:|:||:||||||..||:.|||:..|||.|:|:||

E.jubatus 151 HWKTYSPGWPHCLDEKSVKDLDLNIKYSVMKNSCFYLKVSSAFIELKLKG 200

H.sapiens 200 LLDRKGLWRSLNEMKRIFNFRRTPAAEHAFEHWQEDAFFASQFLNGLNPV 249

||||||.|:|||||:|:.|||:|||||:.|:|||||||||||||||||||

E.jubatus 201 LLDRKGPWKSLNEMRRMLNFRKTPAAEYVFKHWQEDAFFASQFLNGLNPV 250

H.sapiens 250 LIRRCHYLPKNFPVTDAMVASVLGPGTSLQAELE-KGSLFLVDHGILSGI 298

|||||.:||:||||||.|||.:|||||||||||| :||..|...||||||

E.jubatus 251 LIRRCRHLPENFPVTDDMVAPMLGPGTSLQAELEVRGSGGLPVPGILSGI 300

H.sapiens 299 QTNVINGKPQFSAAPMTLLYQSPGCGPLLPLAIQLSQTPGPNSPIFLPTD 348

:|||:||||||||||||||:|.||.|||||||||||||||||||||||:|

E.jubatus 301 RTNVVNGKPQFSAAPMTLLFQRPGGGPLLPLAIQLSQTPGPNSPIFLPSD 350

H.sapiens 349 DKWDWLLAKTWVRNAEFSFHEALTHLLHSHLLPEVFTLATLRQLPHCHPL 398

::||||||||||||||||.|||:|||||:||||||||||||.||||||||

E.jubatus 351 NRWDWLLAKTWVRNAEFSVHEAVTHLLHTHLLPEVFTLATLHQLPHCHPL 400

H.sapiens 399 FKLLIPHTRYTLHINTLARELLIVPGQVVDRSTGIGIEGFSELIQRNMKQ 448

|||||||||||||||||||||||.|||||||||||||.||||||||||:|

E.jubatus 401 FKLLIPHTRYTLHINTLARELLIAPGQVVDRSTGIGIGGFSELIQRNMEQ 450

H.sapiens 449 LNYSLLCLPEDIRTRGVEDIPGYYYRDDGMQIWGAVERFVSEIIGIYYPS 498

|:||:||||||||.|.|||||.|||||||:|||||||.||||||.|||||

E.jubatus 451 LSYSVLCLPEDIRARAVEDIPNYYYRDDGLQIWGAVESFVSEIINIYYPS 500

H.sapiens 499 DESVQDDRELQAWVREIFSKGFLNQESSGIPSSLETREALVQYVTMVIFT 548

|.||.||.||||||.||||:|||.:||||:||:||||||||||||||||.

E.jubatus 501 DASVHDDNELQAWVWEIFSEGFLGRESSGVPSTLETREALVQYVTMVIFN 550

H.sapiens 549 CSAKHAAVSAGQFDSCAWMPNLPPSMQLPPPTSKGLATCEGFIATLPPVN 598

|||||.||||||||||.|||||||:||||||||||....|||:|||||||

E.jubatus 551 CSAKHYAVSAGQFDSCVWMPNLPPTMQLPPPTSKGQTRPEGFLATLPPVN 600

H.sapiens 599 ATCDVILALWLLSKEPGDQRPLGTYPDEHFTEEAPRRSIATFQSRLAQIS 648

||||:|:|||:|||||||||||||||:|||.||||::||..|||||||||

E.jubatus 601 ATCDIIIALWILSKEPGDQRPLGTYPEEHFMEEAPQQSITAFQSRLAQIS 650

H.sapiens 649 RGIQERNQGLVLPYTYLDPPLIENSVSI 676

:.|:||||.|.|||||||||||||||||

E.jubatus 651 KDIRERNQRLSLPYTYLDPPLIENSVSI 678

Pacific walrus (*Odobenus rosmarus divergens*)

H.sapiens 1 MAEFRVRVSTGEAFGAGTWDKVSVSIVGTRGESPPLPLDNLGKEFTAGAE 50

||:|.||||||||.|||||:|:::||||||||:.||.||:.||||:|||.

O.rasmarus div. 1 MAKFSVRVSTGEAIGAGTWNKIAISIVGTRGETTPLRLDHAGKEFSAGAV 50

H.sapiens 51 EDFQVTLPEDVGRVLLLRVHKAPPVLPL-LGPLAPDAWFCRWFQLTPPRG 99

||||:..|:|||.||||||||||.:||: :|.:|.|||||||||||||||

O.rasmarus div. 51 EDFQLEFPQDVGAVLLLRVHKAPLLLPVPVGFVARDAWFCRWFQLTPPRG 100

H.sapiens 100 GHLLFPCYQWLEGAGTLVLQEGTAKVSWADHHPVLQQQRQEELQARQEMY 149

..|.|||||||||||:|.|:.|.||||.||.||:|:|||||||||||:.|

O.rasmarus div. 101 APLRFPCYQWLEGAGSLALRAGAAKVSRADDHPILRQQRQEELQARQDSY 150

H.sapiens 150 QWKAYNPGWPHCLDEKTVEDLELNIKYSTAKNANFYLQAGSAFAEMKIKG 199

.||.|:||||||||||:|:||:||||||..||:.|||:..|||.|:|:||

O.rasmarus div. 151 HWKTYSPGWPHCLDEKSVKDLDLNIKYSVMKNSCFYLKVNSAFMELKLKG 200

H.sapiens 200 LLDRKGLWRSLNEMKRIFNFRRTPAAEHAFEHWQEDAFFASQFLNGLNPV 249

||||||.|:|||||:|:.||.:|||||:.|:|||||||||||||||||||

O.rasmarus div. 201 LLDRKGPWKSLNEMRRMLNFHKTPAAEYVFKHWQEDAFFASQFLNGLNPV 250

H.sapiens 250 LIRRCHYLPKNFPVTDAMVASVLGPGTSLQAELEKGSLFLVDHGILSGIQ 299

|||||.:||:||||||.|||.|||||||||||||:||||||||||||||:

O.rasmarus div. 251 LIRRCRHLPENFPVTDDMVAPVLGPGTSLQAELERGSLFLVDHGILSGIR 300

H.sapiens 300 TNVINGKPQFSAAPMTLLYQSPGCGPLLPLAIQLSQTPGPNSPIFLPTDD 349

|||:||:|||||||||||:|.||.||||||||||||||||:||||||:|:

O.rasmarus div. 301 TNVVNGRPQFSAAPMTLLFQRPGGGPLLPLAIQLSQTPGPDSPIFLPSDN 350

H.sapiens 350 KWDWLLAKTWVRNAEFSFHEALTHLLHSHLLPEVFTLATLRQLPHCHPLF 399

||||||||.||||||||.|||:|||||:||||||||||||||||||||||

O.rasmarus div. 351 KWDWLLAKMWVRNAEFSVHEAVTHLLHAHLLPEVFTLATLRQLPHCHPLF 400

H.sapiens 400 KLLIPHTRYTLHINTLARELLIVPGQVVDRSTGIGIEGFSELIQRNMKQL 449

||||||||||||||||||||||.||||||||||||:.||||||||||:||

O.rasmarus div. 401 KLLIPHTRYTLHINTLARELLIAPGQVVDRSTGIGVGGFSELIQRNMEQL 450

H.sapiens 450 NYSLLCLPEDIRTRGVEDIPGYYYRDDGMQIWGAVERFVSEIIGIYYPSD 499

:||:||||||||.|||||||.|||||||:|||||||.||||||.||||||

O.rasmarus div. 451 SYSVLCLPEDIRARGVEDIPNYYYRDDGLQIWGAVESFVSEIINIYYPSD 500

H.sapiens 500 ESVQDDRELQAWVREIFSKGFLNQESSGIPSSLETREALVQYVTMVIFTC 549

.||.||.||||||.||||:|||.:||||:||:||||||||||||||||.|

O.rasmarus div. 501 VSVHDDHELQAWVWEIFSEGFLGRESSGVPSTLETREALVQYVTMVIFNC 550

H.sapiens 550 SAKHAAVSAGQFDSCAWMPNLPPSMQLPPPTSKGLATCEGFIATLPPVNA 599

||||.||||||||||.|||||||:||||||||||....|||:||||||||

O.rasmarus div. 551 SAKHYAVSAGQFDSCVWMPNLPPTMQLPPPTSKGQTRPEGFLATLPPVNA 600

H.sapiens 600 TCDVILALWLLSKEPGDQRPLGTYPDEHFTEEAPRRSIATFQSRLAQISR 649

|||:|:|||:|||||||||||||||:||||||||::||..|||||||||:

O.rasmarus div. 601 TCDIIIALWILSKEPGDQRPLGTYPEEHFTEEAPQQSITAFQSRLAQISK 650

H.sapiens 650 GIQERNQGLVLPYTYLDPPLIENSVSI 676

.|:||||.|.|||||||||||||||||

O.rasmarus div. 651 DIRERNQRLSLPYTYLDPPLIENSVSI 677

Grey seal (*Halichoerus grypus*)

H.sapiens 1 MAEFRVRVSTGEAFGAGTWDKVSVSIVGTRGESPPLPLDNLGKEFTAGAE 50

||:..||||||||.|||||:|::|||||||||:|||.||:.||||:|||.

H.grypus 1 MAKLSVRVSTGEAIGAGTWNKIAVSIVGTRGETPPLRLDHPGKEFSAGAV 50

H.sapiens 51 EDFQVTLPEDVGRVLLLRVHKAPPVLPL-LGPLAPDAWFCRWFQLTPPRG 99

||||:..|:|||.||||||||||.:||: :|.:|.|||||||||||||||

H.grypus 51 EDFQLESPQDVGPVLLLRVHKAPLLLPVPVGAVARDAWFCRWFQLTPPRG 100

H.sapiens 100 GHLLFPCYQWLEGAGTLVLQEGTAKVSWADHHPVLQQQRQEELQARQEMY 149

..|.|||||||||||:|.|:.|.|||||||.||:|:|||||||||||:.|

H.grypus 101 APLRFPCYQWLEGAGSLSLRAGAAKVSWADDHPILRQQRQEELQARQDSY 150

H.sapiens 150 QWKAYNPGWPHCLDEKTVEDLELNIKYSTAKNANFYLQAGSAFAEMKIKG 199

.||.|.||||||||||:|:||:||||||..||:.|||:..|||.|:|:||

H.grypus 151 HWKTYRPGWPHCLDEKSVKDLDLNIKYSVMKNSCFYLKFSSAFTELKLKG 200

H.sapiens 200 LLDRKGLWRSLNEMKRIFNFRRTPAAEHAFEHWQEDAFFASQFLNGLNPV 249

||||||||:|||||:|:.||.:|||||:.|:|||||||||||||||||||

H.grypus 201 LLDRKGLWKSLNEMRRMLNFCKTPAAEYVFKHWQEDAFFASQFLNGLNPV 250

H.sapiens 250 LIRRCHYLPKNFPVTDAMVASVLGPGTSLQAELEKGSLFLVDHGILSGIQ 299

||.||.:||:||||||.|||.|||||||||||||:||||||||||||||:

H.grypus 251 LIHRCRHLPENFPVTDDMVAPVLGPGTSLQAELERGSLFLVDHGILSGIR 300

H.sapiens 300 TNVINGKPQFSAAPMTLLYQSPGCGPLLPLAIQLSQTPGPNSPIFLPTDD 349

||.:||:|||||||||||:|.||.||||||||||||||||:||||||.|:

H.grypus 301 TNAVNGRPQFSAAPMTLLFQRPGGGPLLPLAIQLSQTPGPDSPIFLPGDN 350

H.sapiens 350 KWDWLLAKTWVRNAEFSFHEALTHLLHSHLLPEVFTLATLRQLPHCHPLF 399

||||||||||||:||||.|||:|||||:|||||||.||||||||||||||

H.grypus 351 KWDWLLAKTWVRHAEFSVHEAVTHLLHAHLLPEVFALATLRQLPHCHPLF 400

H.sapiens 400 KLLIPHTRYTLHINTLARELLIVPGQVVDRSTGIGIEGFSELIQRNMKQL 449

||||||||||||||||||||||.|||||||||.||:.||||||||||:||

H.grypus 401 KLLIPHTRYTLHINTLARELLIAPGQVVDRSTSIGVGGFSELIQRNMEQL 450

H.sapiens 450 NYSLLCLPEDIRTRGVEDIPGYYYRDDGMQIWGAVERFVSEIIGIYYPSD 499

:||:|||||||:.|||||||.|||||||:|||||||.||.|||.||||||

H.grypus 451 SYSVLCLPEDIQARGVEDIPNYYYRDDGLQIWGAVESFVFEIINIYYPSD 500

H.sapiens 500 ESVQDDRELQAWVREIFSKGFLNQESSGIPSSLETREALVQYVTMVIFTC 549

.||..|.||||||.||||:|||.:||||:||:||||||||:|||||||.|

H.grypus 501 ASVHADHELQAWVWEIFSEGFLGRESSGVPSALETREALVKYVTMVIFNC 550

H.sapiens 550 SAKHAAVSAGQFDSCAWMPNLPPSMQLPPPTSKGLATCEGFIATLPPVNA 599

||||.||||||||.|.|||||||:||||||||||....|||:||||||||

H.grypus 551 SAKHYAVSAGQFDFCVWMPNLPPTMQLPPPTSKGQTRPEGFLATLPPVNA 600

H.sapiens 600 TCDVILALWLLSKEPGDQRPLGTYPDEHFTEEAPRRSIATFQSRLAQISR 649

|||:|:|||||||||||||||||||:||||||||::||..|||||||||:

H.grypus 601 TCDIIIALWLLSKEPGDQRPLGTYPEEHFTEEAPQQSITAFQSRLAQISK 650

H.sapiens 650 GIQERNQGLVLPYTYLDPPLIENSVSI 676

.|:|||:.|.|||||||||||||||||

H.grypus 651 DIRERNRRLSLPYTYLDPPLIENSVSI 677

Harbor seal (*Phoca vitulina*)

H.sapiens 1 MAEFRVRVSTGEAFGAGTWDKVSVSIVGTRGESPPLPLDNLGKEFTAGAE 50

||:..||||||||.|||||:|::|||||||||:|||.||:.||||:|||.

P.vitulina 1 MAKLSVRVSTGEAIGAGTWNKIAVSIVGTRGETPPLRLDHPGKEFSAGAV 50

H.sapiens 51 EDFQVTLPEDVGRVLLLRVHKAPPVLPL-LGPLAPDAWFCRWFQLTPPRG 99

||||:..|:|||.||||||||||.:||: :|.:|.|||||||||||||||

P.vitulina 51 EDFQLESPQDVGPVLLLRVHKAPLLLPVPVGAVARDAWFCRWFQLTPPRG 100

H.sapiens 100 GHLLFPCYQWLEGAGTLVLQEGTAKVSWADHHPVLQQQRQEELQARQEMY 149

..|.|||||||||||:|.|:.|.|||||||.||:|||||||||||||:.|

P.vitulina 101 APLRFPCYQWLEGAGSLSLRAGAAKVSWADDHPILQQQRQEELQARQDSY 150

H.sapiens 150 QWKAYNPGWPHCLDEKTVEDLELNIKYSTAKNANFYLQAGSAFAEMKIKG 199

.||.|.||||||||||:|:||:||||||..||:.|||:..|||.|:|:||

P.vitulina 151 HWKTYRPGWPHCLDEKSVKDLDLNIKYSVMKNSCFYLKFSSAFTELKLKG 200

H.sapiens 200 LLDRKGLWRSLNEMKRIFNFRRTPAAEHAFEHWQEDAFFASQFLNGLNPV 249

||||||||:|||||:|:.||.:|||||:.|:|||||||||||||||||||

P.vitulina 201 LLDRKGLWKSLNEMRRMLNFCKTPAAEYVFKHWQEDAFFASQFLNGLNPV 250

H.sapiens 250 LIRRCHYLPKNFPVTDAMVASVLGPGTSLQAELEKGSLFLVDHGILSGIQ 299

||.||.:||:||||||.|||.|||||||||||||:||||||||||||||:

P.vitulina 251 LIHRCRHLPENFPVTDDMVAPVLGPGTSLQAELERGSLFLVDHGILSGIR 300

H.sapiens 300 TNVINGKPQFSAAPMTLLYQSPGCGPLLPLAIQLSQTPGPNSPIFLPTDD 349

||.:||:|||||||||||:|.||.||||||||||||||||:||||||.|:

P.vitulina 301 TNAVNGRPQFSAAPMTLLFQRPGGGPLLPLAIQLSQTPGPDSPIFLPGDN 350

H.sapiens 350 KWDWLLAKTWVRNAEFSFHEALTHLLHSHLLPEVFTLATLRQLPHCHPLF 399

||||||||||||:||||.|||:|||||:|||||||.||||||||||||||

P.vitulina 351 KWDWLLAKTWVRHAEFSVHEAVTHLLHAHLLPEVFALATLRQLPHCHPLF 400

H.sapiens 400 KLLIPHTRYTLHINTLARELLIVPGQVVDRSTGIGIEGFSELIQRNMKQL 449

||||||||||||||||||||||.|||||||||.||:.||||||||||:||

P.vitulina 401 KLLIPHTRYTLHINTLARELLIAPGQVVDRSTSIGVGGFSELIQRNMEQL 450

H.sapiens 450 NYSLLCLPEDIRTRGVEDIPGYYYRDDGMQIWGAVERFVSEIIGIYYPSD 499

:||:|||||||:.|||||||.|||||||:|||||||.||.|||.||||||

P.vitulina 451 SYSVLCLPEDIQARGVEDIPNYYYRDDGLQIWGAVESFVFEIINIYYPSD 500

H.sapiens 500 ESVQDDRELQAWVREIFSKGFLNQESSGIPSSLETREALVQYVTMVIFTC 549

.||..|.||||||.||||:|||.:||||:||:||||||||:|||||||.|

P.vitulina 501 ASVHADHELQAWVWEIFSEGFLGRESSGVPSALETREALVKYVTMVIFNC 550

H.sapiens 550 SAKHAAVSAGQFDSCAWMPNLPPSMQLPPPTSKGLATCEGFIATLPPVNA 599

||||.||||||||||.|||||||:||||||||||....|||:||||||||

P.vitulina 551 SAKHYAVSAGQFDSCVWMPNLPPTMQLPPPTSKGQTRPEGFLATLPPVNA 600

H.sapiens 600 TCDVILALWLLSKEPGDQRPLGTYPDEHFTEEAPRRSIATFQSRLAQISR 649

|||:|:|||||||||||||||||||:||||||||::||..|||||||||:

P.vitulina 601 TCDIIIALWLLSKEPGDQRPLGTYPEEHFTEEAPQQSITAFQSRLAQISK 650

H.sapiens 650 GIQERNQGLVLPYTYLDPPLIENSVSI 676

.|:|||:.|.|||||||||||||||||

P.vitulina 651 DIRERNRRLSLPYTYLDPPLIENSVSI 677

Blue whale (*Balaenoptera musculus*)

H.sapiens 1 MAEFRVRVSTGEAFGAGTWDKVSVSIVGTRGESPPLPLDNLGKEFTAGAE 50

|||||||||||||||||||||:||.||||.||:.|||||:.|||||||||

B.musculus 1 MAEFRVRVSTGEAFGAGTWDKMSVIIVGTEGETLPLPLDHFGKEFTAGAE 50

H.sapiens 51 EDFQVTLPEDVGRVLLLRVHKAPPVL--PLLGPLAPDAWFCRWFQLTPPR 98

|||:||.|:||||||||||||.||.| | ..|||.||||||||||||||

B.musculus 51 EDFEVTCPQDVGRVLLLRVHKTPPALLRP-FWPLASDAWFCRWFQLTPPR 99

H.sapiens 99 GGHLLFPCYQWLEGAGTLVLQEGTAKVSWADHHPVLQQQRQEELQARQEM 148

|..|.||||||||..|:|||:|||||:||.||||.||||||||||||:|.

B.musculus 100 GAPLRFPCYQWLEDKGSLVLREGTAKISWEDHHPTLQQQRQEELQARRET 149

H.sapiens 149 YQWKAYNPGWPHCLDEKTVEDLELNIKYSTAKNANFYLQAGSAFAEMKIK 198

|.||.|.|||||||:|:|:::|:||||||.|||..|||:.||..|::|:|

B.musculus 150 YSWKTYIPGWPHCLNEETIKNLDLNIKYSLAKNITFYLRGGSMLADLKLK 199

H.sapiens 199 GLLDRKGLWRSLNEMKRIFNFRRTPAAEHAFEHWQEDAFFASQFLNGLNP 248

|||||||||:||.||:.:||.|:|||.|:.|||||||||||.||||||||

B.musculus 200 GLLDRKGLWKSLEEMRSVFNSRKTPAVEYVFEHWQEDAFFAYQFLNGLNP 249

H.sapiens 249 VLIRRCHYLPKNFPVTDAMVASVLGPGTSLQAELEKGSLFLVDHGILSGI 298

||||||.:|||||||||||||..|||||||||||||||||||||||||||

B.musculus 250 VLIRRCRHLPKNFPVTDAMVAPALGPGTSLQAELEKGSLFLVDHGILSGI 299

H.sapiens 299 QTNVINGKPQFSAAPMTLLYQSPGCGPLLPLAIQLSQTPGPNSPIFLPTD 348

.||||||:|||||||||||||.|||||||||||||||||||:||||||:|

B.musculus 300 CTNVINGRPQFSAAPMTLLYQRPGCGPLLPLAIQLSQTPGPDSPIFLPSD 349

H.sapiens 349 DKWDWLLAKTWVRNAEFSFHEALTHLLHSHLLPEVFTLATLRQLPHCHPL 398

||||||||||||||||||.||||||||..||:.|||||||||||||||||

B.musculus 350 DKWDWLLAKTWVRNAEFSIHEALTHLLQVHLVTEVFTLATLRQLPHCHPL 399

H.sapiens 399 FKLLIPHTRYTLHINTLARELLIVPGQVVDRSTGIGIEGFSELIQRNMKQ 448

|||||||||||||||||||||||.||:|||||||:|||||||||||.|:|

B.musculus 400 FKLLIPHTRYTLHINTLARELLIAPGKVVDRSTGLGIEGFSELIQRYMEQ 449

H.sapiens 449 LNYSLLCLPEDIRTRGVEDIPGYYYRDDGMQIWGAVERFVSEIIGIYYPS 498

||||:||||||||.|||||||||||||||||||||||.||||:|||||.|

B.musculus 450 LNYSVLCLPEDIRARGVEDIPGYYYRDDGMQIWGAVEGFVSEMIGIYYLS 499

H.sapiens 499 DESVQDDRELQAWVREIFSKGFLNQESSGIPSSLETREALVQYVTMVIFT 548

||.|:||.||||||.||||:|||.:||||:||||.|||||:||:||||||

B.musculus 500 DEFVRDDSELQAWVWEIFSEGFLGRESSGVPSSLGTREALIQYITMVIFT 549

H.sapiens 549 CSAKHAAVSAGQFDSCAWMPNLPPSMQLPPPTSKGLATCEGFIATLPPVN 598

|||||:||..||||..||||||||:||||||||||.|:.|||||||||||

B.musculus 550 CSAKHSAVGTGQFDFSAWMPNLPPTMQLPPPTSKGQASPEGFIATLPPVN 599

H.sapiens 599 ATCDVILALWLLSKEPGDQRPLGTYPDEHFTEEAPRRSIATFQSRLAQIS 648

||||||.|||:|||||||:|||||||||||||.||..|||.|||||||||

B.musculus 600 ATCDVITALWVLSKEPGDRRPLGTYPDEHFTEAAPCWSIAAFQSRLAQIS 649

H.sapiens 649 RGIQERNQGLVLPYTYLDPPLIENSVSI 676

|.||||||.|.|||||||||:|||||||

B.musculus 650 RDIQERNQDLELPYTYLDPPIIENSVSI 677

Chinese river dolphin (Lipotes vexillifer)

H.sapiens 1 MAEFRVRVSTGEAFGAGTWDKVSVSIVGTRGESPPLPLDNLGKEFTAGAE 50

|||||||||||||||||||||:||.||||.||:.|||||:.|||||||||

L.vexillifer 1 MAEFRVRVSTGEAFGAGTWDKMSVIIVGTEGETLPLPLDHFGKEFTAGAE 50

H.sapiens 51 EDFQVTLPEDVGRVLLLRVHKAPPVLPL-LGPLAPDAWFCRWFQLTPPRG 99

|||:||.|:||||||||||||.||.||. ...||.|||||||.|||||.|

L.vexillifer 51 EDFEVTCPQDVGRVLLLRVHKTPPALPRPFWSLASDAWFCRWVQLTPPWG 100

H.sapiens 100 GHLLFPCYQWLEGAGTLVLQEGTAKVSWADHHPVLQQQRQEELQARQEMY 149

..|.||||||||..|:|||:|||.::...| ..

L.vexillifer 101 APLRFPCYQWLEDQGSLVLREGTGQLPLCD------------------AS 132

H.sapiens 150 QWKAYNPGWPHCLDEKTVEDLELNIKYSTAKNANFYLQAGSAFAEMKIKG 199

.||.|.||||||.:|:|:::|:||||||.|||..|||:..|..|::|:||

L.vexillifer 133 VWKTYIPGWPHCFNEETIKNLDLNIKYSLAKNITFYLRGVSLLADLKLKG 182

H.sapiens 200 LLDRKGLWRSLNEMKRIFNFRRTPAAEHAFEHWQEDAFFASQFLNGLNPV 249

||||||||:||.||:.:||..:.||.|:.|||||||||||.|||||||||

L.vexillifer 183 LLDRKGLWKSLEEMRSVFNSLKNPAVEYVFEHWQEDAFFAYQFLNGLNPV 232

H.sapiens 250 LIRRCHYLPKNFPVTDAMVASVLGPGTSLQAELEKGSLFLVDHGILSGIQ 299

|||||.:|||||||||||||.||||||||||||||||||||||||||||:

L.vexillifer 233 LIRRCRHLPKNFPVTDAMVAPVLGPGTSLQAELEKGSLFLVDHGILSGIR 282

H.sapiens 300 TNVINGKPQFSAAPMTLLYQSPGCGPLLPLAIQLSQTPGPNSPIFLPTDD 349

|||||.:|||||||||||||.|||||||||||||||||||:||||||:||

L.vexillifer 283 TNVINRRPQFSAAPMTLLYQCPGCGPLLPLAIQLSQTPGPDSPIFLPSDD 332

H.sapiens 350 KWDWLLAKTWVRNAEFSFHEALTHLLHSHLLPEVFTLATLRQLPHCHPLF 399

|||||||||||||||||.||||||||..||:.||||||||||||||||||

L.vexillifer 333 KWDWLLAKTWVRNAEFSIHEALTHLLQVHLVTEVFTLATLRQLPHCHPLF 382

H.sapiens 400 KLLIPHTRYTLHINTLARELLIVPGQVVDRSTGIGIEGFSELIQRNMKQL 449

||||||||||||||||||||||.||:|||||||:|||||||||||.|:||

L.vexillifer 383 KLLIPHTRYTLHINTLARELLIAPGKVVDRSTGLGIEGFSELIQRYMEQL 432

H.sapiens 450 NYSLLCLPEDIRTRGVEDIPGYYYRDDGMQIWGAVERFVSEIIGIYYPSD 499

|||:||||||||.|||||||||||||||||||||||.||||:|||||.||

L.vexillifer 433 NYSVLCLPEDIRARGVEDIPGYYYRDDGMQIWGAVECFVSEMIGIYYLSD 482

H.sapiens 500 ESVQDDRELQAWVREIFSKGFLNQESSGIPSSLETREALVQYVTMVIFTC 549

|||:||.||||||.||||:|||.:||||:||||.|||||:||||||||||

L.vexillifer 483 ESVRDDSELQAWVWEIFSEGFLGRESSGVPSSLGTREALIQYVTMVIFTC 532

H.sapiens 550 SAKHAAVSAGQFDSCAWMPNLPPSMQLPPPTSKGLATCEGFIATLPPVNA 599

||||:|:..||||..||||||||:||||||||||.|:.||||||||||||

L.vexillifer 533 SAKHSAIGTGQFDFSAWMPNLPPTMQLPPPTSKGQASPEGFIATLPPVNA 582

H.sapiens 600 TCDVILALWLLSKEPGDQRPLGTYPDEHFTEEAPRRSIATFQSRLAQISR 649

|||||:|||:|||||||:|||||||||||||.||..|||.||||||||||

L.vexillifer 583 TCDVIIALWVLSKEPGDRRPLGTYPDEHFTEAAPCWSIAAFQSRLAQISR 632

H.sapiens 650 GIQERNQGLVLPYTYLDPPLIENSVSI 676

.||||||.|.|||||||||:|||||||

L.vexillifer 633 DIQERNQDLELPYTYLDPPIIENSVSI 659

Vaquita (*Phocoena sinus*)

H.sapiens 1 MAEFRVRVSTGEAFGAGTWDKVSVSIVGTRGESPPLPLDNLGKEFTAGAE 50

||||||||||||||||||||::||.||||.||:.|||||:.||||:||||

P.sinus 1 MAEFRVRVSTGEAFGAGTWDEMSVIIVGTEGETLPLPLDHFGKEFSAGAE 50

H.sapiens 51 EDFQVTLPEDVGRVLLLRVHKAPPVLPL-LGPLAPDAWFCRWFQLTPPRG 99

|||:||.|:|||||||||:||.||.||. ..|||.|||||||.|||||||

P.sinus 51 EDFEVTCPQDVGRVLLLRLHKTPPALPRPFWPLASDAWFCRWVQLTPPRG 100

H.sapiens 100 GHLLFPCYQWLEGAGTLVLQEGTAKVSWADHHPVLQQQRQEELQARQEMY 149

..|.||||||||..|:|||:|||||:||.|.||.||||||||||||:|||

P.sinus 101 APLRFPCYQWLEDQGSLVLREGTAKISWEDRHPTLQQQRQEELQARREMY 150

H.sapiens 150 QWKAYNPGWPHCLDEKTVEDLELNIKYSTAKNANFYLQAGSAFAEMKIKG 199

.||.|.||||.||:|:|:::|:||||||.|||..|||:.||..|.:|:||

P.sinus 151 SWKTYIPGWPRCLNEETIKNLDLNIKYSLAKNITFYLRGGSMLAHLKLKG 200

H.sapiens 200 LLDRKGLWRSLNEMKRIFNFRRTPAAEHAFEHWQEDAFFASQFLNGLNPV 249

||||||||:||.||:.:|:||:|||.|:.|||||||||||.|||||||||

P.sinus 201 LLDRKGLWKSLEEMRSVFSFRKTPAVEYVFEHWQEDAFFAYQFLNGLNPV 250

H.sapiens 250 LIRRCHYLPKNFPVTDAMVASVLGPGTSLQAELEKGSLFLVDHGILSGIQ 299

|||||.:|||||||||||||.||||||||||||||||||||||||||||:

P.sinus 251 LIRRCRHLPKNFPVTDAMVALVLGPGTSLQAELEKGSLFLVDHGILSGIR 300

H.sapiens 300 TNVINGKPQFSAAPMTLLYQSPGCGPLLPLAIQLSQTPGPNSPIFLPTDD 349

|||||.:|||||||||||||.|||||||||||||||||||:||||||:||

P.sinus 301 TNVINRRPQFSAAPMTLLYQRPGCGPLLPLAIQLSQTPGPDSPIFLPSDD 350

H.sapiens 350 KWDWLLAKTWVRNAEFSFHEALTHLLHSHLLPEVFTLATLRQLPHCHPLF 399

|||||||||||||||||.||||||||..||:.||||||||||||||||||

P.sinus 351 KWDWLLAKTWVRNAEFSIHEALTHLLQVHLVAEVFTLATLRQLPHCHPLF 400

H.sapiens 400 KLLIPHTRYTLHINTLARELLIVPGQVVDRSTGIGIEGFSELIQRNMKQL 449

||||||||||||||||||||||.|.:|||||||||||||||||||.|:||

P.sinus 401 KLLIPHTRYTLHINTLARELLIAPRKVVDRSTGIGIEGFSELIQRYMEQL 450

H.sapiens 450 NYSLLCLPEDIRTRGVEDIPGYYYRDDGMQIWGAVERFVSEIIGIYYPSD 499

|||:||||||||.|||||||||||||||||||||||.||||:|||||.||

P.sinus 451 NYSILCLPEDIRARGVEDIPGYYYRDDGMQIWGAVECFVSEMIGIYYLSD 500

H.sapiens 500 ESVQDDRELQAWVREIFSKGFLNQESSGIPSSLETREALVQYVTMVIFTC 549

|||:||.||||||.||||:|||.:||||:||||.|||||:||||||||||

P.sinus 501 ESVRDDSELQAWVWEIFSEGFLGRESSGVPSSLGTREALIQYVTMVIFTC 550

H.sapiens 550 SAKHAAVSAGQFDSCAWMPNLPPSMQLPPPTSKGLATCEGFIATLPPVNA 599

||||:||..||||..||||||||:||||||||||.|:.|.||||||||||

P.sinus 551 SAKHSAVGTGQFDFSAWMPNLPPTMQLPPPTSKGQASLEKFIATLPPVNA 600

H.sapiens 600 TCDVILALWLLSKEPGDQRPLGTYPDEHFTEEAPRRSIATFQSRLAQISR 649

|||||:|||.|||||||:|.|||||||||||.||..|||.||||||||||

P.sinus 601 TCDVIIALWALSKEPGDRRSLGTYPDEHFTEVAPCWSIAAFQSRLAQISR 650

H.sapiens 650 GIQERNQGLVLPYTYLDPPLIENSVSI 676

.||||||.|.|||||||||:|||||||

P.sinus 651 DIQERNQDLELPYTYLDPPIIENSVSI 677

Orca (*Orcinus orca*)

H.sapiens 1 MAEFRVRVSTGEAFGAGTWDKVSVSIVGTRGESPPLPLDNLGKEFTAGA- 49

|||||||||||||||||||||:||.||||.||:.|||||:.||||:|||

O.orca 1 MAEFRVRVSTGEAFGAGTWDKMSVIIVGTEGETLPLPLDHFGKEFSAGAD 50

H.sapiens 50 ------EEDFQVTLPEDVGRVLLLRVHKAPPVL--PLLGPLAPDAWFCRW 91

||||:||.|:|||||||||||||||.| | ..|||.|||||||

O.orca 51 TKLAPQEEDFKVTCPQDVGRVLLLRVHKAPPTLLRP-FWPLASDAWFCRW 99

H.sapiens 92 FQLTPPRGGHLLFPCYQWLEGAGTLVLQEGTAKVSWADHHPVLQQQRQEE 141

.|||||||..|.||||||||..|:|||:|||||:|..|.||.||||||||

O.orca 100 VQLTPPRGAPLRFPCYQWLEDQGSLVLREGTAKISREDRHPTLQQQRQEE 149

H.sapiens 142 LQARQEMYQWKAYNPGWPHCLDEKTVEDLELNIKYSTAKNANFYLQAGSA 191

||||:|.|.||.|.||||.||:|:|:::|:||||||.|||..|||:..|.

O.orca 150 LQARRETYSWKTYIPGWPRCLNEETIKNLDLNIKYSLAKNITFYLRGSSM 199

H.sapiens 192 FAEMKIKGLLDRKGLWRSLNEMKRIFNFRRTPAAEHAFEHWQEDAFFASQ 241

.|.:|:||||||||||:||.||:.:|||.:|||.|:.|||||||||||.|

O.orca 200 LAHLKLKGLLDRKGLWKSLEEMRSVFNFWKTPAVEYVFEHWQEDAFFAYQ 249

H.sapiens 242 FLNGLNPVLIRRCHYLPKNFPVTDAMVASVLGPGTSLQAELEKGSLFLVD 291

|||||||||||||.:|||||||||||||.|||||||||||||||||||||

O.orca 250 FLNGLNPVLIRRCRHLPKNFPVTDAMVAPVLGPGTSLQAELEKGSLFLVD 299

H.sapiens 292 HGILSGIQTNVINGKPQFSAAPMTLLYQSPGCGPLLPLAIQLSQTPGPNS 341

:||||||:|||||.:|||||||||||||.|||||||||||||||||||:|

O.orca 300 YGILSGIRTNVINRRPQFSAAPMTLLYQRPGCGPLLPLAIQLSQTPGPDS 349

H.sapiens 342 PIFLPTDDKWDWLLAKTWVRNAEFSFHEALTHLLHSHLLPEVFTLATLRQ 391

|||||:|||||||||||||||||||.||||||||..||:.||||||||||

O.orca 350 PIFLPSDDKWDWLLAKTWVRNAEFSIHEALTHLLQVHLVTEVFTLATLRQ 399

H.sapiens 392 LPHCHPLFKLLIPHTRYTLHINTLARELLIVPGQVVDRSTGIGIEGFSEL 441

||||||||||||||||||||||||||||||.||:|||||||:||||||||

O.orca 400 LPHCHPLFKLLIPHTRYTLHINTLARELLIAPGKVVDRSTGLGIEGFSEL 449

H.sapiens 442 IQRNMKQLNYSLLCLPEDIRTRGVEDIPGYYYRDDGMQIWGAVERFVSEI 491

|||.|:|||||:||||||||.|||||||||||||||||||||||.||||:

O.orca 450 IQRYMEQLNYSVLCLPEDIRARGVEDIPGYYYRDDGMQIWGAVECFVSEM 499

H.sapiens 492 IGIYYPSDESVQDDRELQAWVREIFSKGFLNQESSGIPSSLETREALVQY 541

|||||.|||||:||.||||||.||||:|||.:||||:||||.|||||:||

O.orca 500 IGIYYLSDESVRDDSELQAWVWEIFSEGFLGRESSGVPSSLGTREALIQY 549

H.sapiens 542 VTMVIFTCSAKHAAVSAGQFDSCAWMPNLPPSMQLPPPTSKGLATCEGFI 591

||||||||||||:||..||||..||||||||:||||||||||.|:.|.||

O.orca 550 VTMVIFTCSAKHSAVGTGQFDFSAWMPNLPPTMQLPPPTSKGQASPERFI 599

H.sapiens 592 ATLPPVNATCDVILALWLLSKEPGDQRPLGTYPDEHFTEEAPRRSIATFQ 641

|||||||||||||:|||.|||||||:|.|||||:|||||.||..|||.||

O.orca 600 ATLPPVNATCDVIVALWALSKEPGDRRSLGTYPEEHFTEAAPCWSIAAFQ 649

H.sapiens 642 SRLAQISRGIQERNQGLVLPYTYLDPPLIENSVSI 676

||||||||.||||||.|.|||||||||:|||||||

O.orca 650 SRLAQISRDIQERNQDLELPYTYLDPPIIENSVSI 684

Narwhale (*Monodon monoceros*)

H.sapiens 1 MAEFRVRVSTGEAFGAGTWDKVSVSIVGTRGESPPLPLDNLGKEFTAGAE 50

|||||||||||||||||||||:||.||||.||:.|||||:.||||:||||

M.monoceros 1 MAEFRVRVSTGEAFGAGTWDKMSVIIVGTEGETLPLPLDHFGKEFSAGAE 50

H.sapiens 51 EDFQVTLPEDVGRVLLLRVHKAPPVL--PLLGPLAPDAWFCRWFQLTPPR 98

|||:||.|:|||||||||:||.||.| | ..|||.|||||||.||||||

M.monoceros 51 EDFEVTCPQDVGRVLLLRLHKTPPALLRP-FWPLASDAWFCRWVQLTPPR 99

H.sapiens 99 GGHLLFPCYQWLEGAGTLVLQEGTAKVSWADHHPVLQQQRQEELQARQEM 148

|..|.||||||||..|:|||:|||||:||.|.||.||||||||||||:||

M.monoceros 100 GAPLRFPCYQWLEDQGSLVLREGTAKISWEDRHPTLQQQRQEELQARREM 149

H.sapiens 149 YQWKAYNPGWPHCLDEKTVEDLELNIKYSTAKNANFYLQAGSAFAEMKIK 198

|.||.|.||||.||:|:|:::|:||||||.|||..|||:.||..|.:|:|

M.monoceros 150 YSWKTYIPGWPRCLNEETIKNLDLNIKYSLAKNITFYLRGGSMLAHLKLK 199

H.sapiens 199 GLLDRKGLWRSLNEMKRIFNFRRTPAAEHAFEHWQEDAFFASQFLNGLNP 248

|||||||||:||.||:.:||||:|||.|:.|||||||||||.||||||||

M.monoceros 200 GLLDRKGLWKSLEEMRSVFNFRKTPAVEYVFEHWQEDAFFAYQFLNGLNP 249

H.sapiens 249 VLIRRCHYLPKNFPVTDAMVASVLGPGTSLQAELEKGSLFLVDHGILSGI 298

||||||.:|||||||||||||.||||||||||||||||||||||||||||

M.monoceros 250 VLIRRCRHLPKNFPVTDAMVAPVLGPGTSLQAELEKGSLFLVDHGILSGI 299

H.sapiens 299 QTNVINGKPQFSAAPMTLLYQSPGCGPLLPLAIQLSQTPGPNSPIFLPTD 348

.|||||.:|||||||||||||.|||||||||||||||||||:||||||:|

M.monoceros 300 HTNVINRRPQFSAAPMTLLYQRPGCGPLLPLAIQLSQTPGPDSPIFLPSD 349

H.sapiens 349 DKWDWLLAKTWVRNAEFSFHEALTHLLHSHLLPEVFTLATLRQLPHCHPL 398

||||||||||||||||||.||||||||..||:.|||||||||||||||||

M.monoceros 350 DKWDWLLAKTWVRNAEFSIHEALTHLLQVHLVTEVFTLATLRQLPHCHPL 399

H.sapiens 399 FKLLIPHTRYTLHINTLARELLIVPGQVVDRSTGIGIEGFSELIQRNMKQ 448

|||||||||||||||||||||||.||:|||||||:||||||||:||.|:|

M.monoceros 400 FKLLIPHTRYTLHINTLARELLIAPGKVVDRSTGLGIEGFSELVQRYMEQ 449

H.sapiens 449 LNYSLLCLPEDIRTRGVEDIPGYYYRDDGMQIWGAVERFVSEIIGIYYPS 498

||||:|||||||:.|||||||||||||||||||||||.||||:|||||.|

M.monoceros 450 LNYSVLCLPEDIQARGVEDIPGYYYRDDGMQIWGAVECFVSEMIGIYYLS 499

H.sapiens 499 DESVQDDRELQAWVREIFSKGFLNQESSGIPSSLETREALVQYVTMVIFT 548

||||:||.||||||.||||:|||.:||||:||||.|||||:|||||||||

M.monoceros 500 DESVRDDSELQAWVWEIFSEGFLGRESSGVPSSLGTREALIQYVTMVIFT 549

H.sapiens 549 CSAKHAAVSAGQFDSCAWMPNLPPSMQLPPPTSKGLATCEGFIATLPPVN 598

|||||:||..||||..||||||||:||||||||||.|:.|.|||||||||

M.monoceros 550 CSAKHSAVGTGQFDFSAWMPNLPPTMQLPPPTSKGQASPERFIATLPPVN 599

H.sapiens 599 ATCDVILALWLLSKEPGDQRPLGTYPDEHFTEEAPRRSIATFQSRLAQIS 648

||||||:|||.|||||||:|.|||||||||||.||..|||.|||||||||

M.monoceros 600 ATCDVIIALWALSKEPGDRRSLGTYPDEHFTEAAPCWSIAAFQSRLAQIS 649

H.sapiens 649 RGIQERNQGLVLPYTYLDPPLIENSVSI 676

|.||||||.|.|||||||||:|||||||

M.monoceros 650 RDIQERNQDLELPYTYLDPPIIENSVSI 677

Common bottlenose dolphin (*Tursiops truncatus*)

H.sapiens 1 MAEFRVRVSTGEAFGAGTWDKVSVSIVGTRGESPPLPLDNLGKEFTAGA- 49

|||||||||||||||||||||:||.||||.||:.|||||:.||||:|||

T.truncatus 1 MAEFRVRVSTGEAFGAGTWDKMSVIIVGTEGETLPLPLDHFGKEFSAGAD 50

H.sapiens 50 ------EEDFQVTLPEDVGRVLLLRVHKAPPVL--PLLGPLAPDAWFCRW 91

||||:||.|:|||||||||||||||.| | ..|||.|||||||

T.truncatus 51 TKLAPQEEDFKVTCPQDVGRVLLLRVHKAPPTLLRP-FWPLASDAWFCRW 99

H.sapiens 92 FQLTPPRGGHLLFPCYQWLEGAGTLVLQEGTAKVSWADHHPVLQQQRQEE 141

.|||||||..|.||||||||..|:|||:|||||:||.|.||.||||||||

T.truncatus 100 VQLTPPRGAPLRFPCYQWLEDQGSLVLREGTAKISWEDRHPTLQQQRQEE 149

H.sapiens 142 LQARQEMYQWKAYNPGWPHCLDEKTVEDLELNIKYSTAKNANFYLQAGSA 191

||||:|.|.||.|.||||.||:|:|:::|:||||||.|||..|||:..|.

T.truncatus 150 LQARRETYSWKTYIPGWPRCLNEETIKNLDLNIKYSLAKNITFYLRGSSM 199

H.sapiens 192 FAEMKIKGLLDRKGLWRSLNEMKRIFNFRRTPAAEHAFEHWQEDAFFASQ 241

.|.:|:||||||||||:||.||:.:|||.:|||.|:.|||||||||||.|

T.truncatus 200 LAHLKLKGLLDRKGLWKSLEEMRSVFNFWKTPAVEYVFEHWQEDAFFAYQ 249

H.sapiens 242 FLNGLNPVLIRRCHYLPKNFPVTDAMVASVLGPGTSLQAELEKGSLFLVD 291

|||||||||||||.:|||||||||||||.|||||||||||||||||||||

T.truncatus 250 FLNGLNPVLIRRCRHLPKNFPVTDAMVAPVLGPGTSLQAELEKGSLFLVD 299

H.sapiens 292 HGILSGIQTNVINGKPQFSAAPMTLLYQSPGCGPLLPLAIQLSQTPGPNS 341

:||||||:|||||.:|||||||||||||.|||||||||||||||||||:|

T.truncatus 300 YGILSGIRTNVINRRPQFSAAPMTLLYQRPGCGPLLPLAIQLSQTPGPDS 349

H.sapiens 342 PIFLPTDDKWDWLLAKTWVRNAEFSFHEALTHLLHSHLLPEVFTLATLRQ 391

|||||:|||||||||||||||||||.||||||||..||:.||||||||||

T.truncatus 350 PIFLPSDDKWDWLLAKTWVRNAEFSIHEALTHLLQVHLVTEVFTLATLRQ 399

H.sapiens 392 LPHCHPLFKLLIPHTRYTLHINTLARELLIVPGQVVDRSTGIGIEGFSEL 441

||||||||||||||||||||||||||||||.||:|||||||:||||||||

T.truncatus 400 LPHCHPLFKLLIPHTRYTLHINTLARELLIAPGKVVDRSTGLGIEGFSEL 449

H.sapiens 442 IQRNMKQLNYSLLCLPEDIRTRGVEDIPGYYYRDDGMQIWGAVERFVSEI 491

|||.|:|||||:||||||||.|||||||||||||||||||||||.||||:

T.truncatus 450 IQRYMEQLNYSVLCLPEDIRARGVEDIPGYYYRDDGMQIWGAVECFVSEM 499

H.sapiens 492 IGIYYPSDESVQDDRELQAWVREIFSKGFLNQESSGIPSSLETREALVQY 541

|||||.|||||:||.||||||.||||:|||.:||||:||||.|||||:||

T.truncatus 500 IGIYYLSDESVRDDSELQAWVWEIFSEGFLGRESSGVPSSLGTREALIQY 549

H.sapiens 542 VTMVIFTCSAKHAAVSAGQFDSCAWMPNLPPSMQLPPPTSKGLATCEGFI 591

||||||||||||:||..||||..||||||||:||||||||||.|:.|.||

T.truncatus 550 VTMVIFTCSAKHSAVGTGQFDFSAWMPNLPPTMQLPPPTSKGQASPERFI 599

H.sapiens 592 ATLPPVNATCDVILALWLLSKEPGDQRPLGTYPDEHFTEEAPRRSIATFQ 641

|||||||||||||:|||.|||||||:|.|||||||||||.||..|||.||

T.truncatus 600 ATLPPVNATCDVIVALWALSKEPGDRRSLGTYPDEHFTEAAPCWSIAAFQ 649

H.sapiens 642 SRLAQISRGIQERNQGLVLPYTYLDPPLIENSVSI 676

||||||||.||||||.|.|||||||||:|||||||

T.truncatus 650 SRLAQISRDIQERNQDLELPYTYLDPPIIENSVSI 684

Polar bear (*Ursus maritimus*)

H.sapiens 1 MAEFRVRVSTGEAFGAGTWDKVSVSIVGTRGESPPLPLDNLGKEFTAGAE 50

||::.||||||||.|||||:|::||:||||||:|||.||:|||||:|||.

U.maritimus 1 MAKYSVRVSTGEAIGAGTWNKIAVSVVGTRGETPPLRLDHLGKEFSAGAV 50

H.sapiens 51 EDFQVTLPEDVGRVLLLRVHKAPPVLPL-LGPLAPDAWFCRWFQLTPPRG 99

|:|:|..|:|||.||||||||||.:||. :|||:.|||||||||||||:|

U.maritimus 51 EEFEVESPQDVGEVLLLRVHKAPLLLPAPIGPLSRDAWFCRWFQLTPPQG 100

H.sapiens 100 GHLLFPCYQWLEGAGTLVLQEGTAKVSWADHHPVLQQQRQEELQARQEMY 149

..|.|||||||:||.:|.|:.|.||||.||.||.|||||||||||||:.|

U.maritimus 101 VPLRFPCYQWLDGAVSLALRAGPAKVSGADDHPTLQQQRQEELQARQDSY 150

H.sapiens 150 QWKAYNPGWPHCLDEKTVEDLELNIKYSTAKNANFYLQAGSAFAEMKIKG 199

.||.|:|||||||:||:|:||:||||||..||:.|||:..|||:|||:||

U.maritimus 151 HWKTYSPGWPHCLNEKSVKDLDLNIKYSVMKNSCFYLKVSSAFSEMKLKG 200

H.sapiens 200 LLDRKGLWRSLNEMKRIFNFRRTPAAEHAFEHWQEDAFFASQFLNGLNPV 249

||||||.|.||:||:.:.|||:|||||:..||||||||||||||||.|||

U.maritimus 201 LLDRKGPWESLDEMQTMLNFRKTPAAEYVVEHWQEDAFFASQFLNGHNPV 250

H.sapiens 250 LIRRCHYLPKNFPVTDAMVASVLGPGTSLQAELEKGSLFLVDHGILSGIQ 299

|||||.:||:|||||:.|||.||||||||:||||:||:|||||||||||.

U.maritimus 251 LIRRCRHLPENFPVTEDMVAPVLGPGTSLKAELERGSVFLVDHGILSGIH 300

H.sapiens 300 TNVINGKPQFSAAPMTLLYQSPGCGPLLPLAIQLSQTPGPNSPIFLPTDD 349

.||:||:|||||||||||||.|..||||||||||||||||:||||||:|:

U.maritimus 301 GNVVNGQPQFSAAPMTLLYQRPRGGPLLPLAIQLSQTPGPDSPIFLPSDN 350

H.sapiens 350 KWDWLLAKTWVRNAEFSFHEALTHLLHSHLLPEVFTLATLRQLPHCHPLF 399

|||||||||||||||||.:|||||||.:||||||||:|||||||.|||||

U.maritimus 351 KWDWLLAKTWVRNAEFSVNEALTHLLQAHLLPEVFTMATLRQLPQCHPLF 400

H.sapiens 400 KLLIPHTRYTLHINTLARELLIVPGQVVDRSTGIGIEGFSELIQRNMKQL 449

|||:|||||||||||||||.||.||:|||||||||||||||||||||:||

U.maritimus 401 KLLVPHTRYTLHINTLAREQLIAPGKVVDRSTGIGIEGFSELIQRNMEQL 450

H.sapiens 450 NYSLLCLPEDIRTRGVEDIPGYYYRDDGMQIWGAVERFVSEIIGIYYPSD 499

:||:||||||||.|||||||.|||||||:|||.|||.||||||.||||||

U.maritimus 451 SYSVLCLPEDIRARGVEDIPNYYYRDDGLQIWAAVESFVSEIINIYYPSD 500

H.sapiens 500 ESVQDDRELQAWVREIFSKGFLNQESSGIPSSLETREALVQYVTMVIFTC 549

.||:||.||||||:|||.:|||.::|||:||:||||||||:|:|||||:|

U.maritimus 501 VSVRDDSELQAWVQEIFLEGFLGRKSSGMPSALETREALVRYITMVIFSC 550

H.sapiens 550 SAKHAAVSAGQFDSCAWMPNLPPSMQLPPPTSKGLATCEGFIATLPPVNA 599

||:|.||||||||||.||||||||||||||||||....|||:||||||||

U.maritimus 551 SARHYAVSAGQFDSCVWMPNLPPSMQLPPPTSKGQTKPEGFLATLPPVNA 600

H.sapiens 600 TCDVILALWLLSKEPGDQRPLGTYPDEHFTEEAPRRSIATFQSRLAQISR 649

|||:|:.|||||||||||||||||||||||||||::|||.|||||||||:

U.maritimus 601 TCDIIITLWLLSKEPGDQRPLGTYPDEHFTEEAPQQSIAAFQSRLAQISK 650

H.sapiens 650 GIQERNQGLVLPYTYLDPPLIENSVSI 676

.||||||||.|||||||||||||||||

U.maritimus 651 EIQERNQGLPLPYTYLDPPLIENSVSI 677

African wild donkey (*Equus asinus*)

H.sapiens 1 MAEFRVRVSTGEAFGAGTWDKVSVSIVGTRGESPPLPLDNLGKEFTAGAE 50

||::|||||||||||||||||:|::||||:||:|.||||:||||||.|||

E.asinus 1 MAKYRVRVSTGEAFGAGTWDKMSITIVGTQGETPRLPLDHLGKEFTVGAE 50

H.sapiens 51 EDFQVTLPEDVGRVLLLRVHKAPP-VLPLLGPLAPDAWFCRWFQLTPPRG 99

|||:|..|:|||.||||||||||| :|.|||||||||||||.||||||||

E.asinus 51 EDFEVKSPQDVGPVLLLRVHKAPPALLLLLGPLAPDAWFCRSFQLTPPRG 100

H.sapiens 100 GHLLFPCYQWLEGAGTLVLQEGTAKVSWADHHPVLQQQRQEELQARQEMY 149

..|.||.||||||||:|||:||.||||||||||.|||||:||||||||||

E.asinus 101 SPLRFPAYQWLEGAGSLVLREGAAKVSWADHHPKLQQQRREELQARQEMY 150

H.sapiens 150 QWKAYNPGWPHCLDEKTVEDLELNIKYSTAKNANFYLQAGSAFAEMKIKG 199

:||.|.||||||||.:||:||:||.|||..|:.|.||:|.|||||:||||

E.asinus 151 RWKTYQPGWPHCLDMETVKDLDLNTKYSVVKSTNLYLRAQSAFAELKIKG 200

H.sapiens 200 LLDRKGLWRSLNEMKRIFNFRRTPAAEHAFEHWQEDAFFASQFLNGLNPV 249

||||||.||||:||:.:.||::|||.|:.|||||||||||||||||||||

E.asinus 201 LLDRKGSWRSLSEMEMVLNFQKTPATEYVFEHWQEDAFFASQFLNGLNPV 250

H.sapiens 250 LIRRCHYLPKNFPVTDAMVASVLGPGTSLQAELEKGSLFLVDHGILSGIQ 299

||.||..||:||||||||||.|||||||||||||:|||||||||||||::

E.asinus 251 LIHRCRRLPENFPVTDAMVAPVLGPGTSLQAELERGSLFLVDHGILSGVR 300

H.sapiens 300 TNVINGKPQFSAAPMTLLYQSPGCGPLLPLAIQLSQTPGPNSPIFLPTDD 349

||||||:|||||||||||||.||.|||||||||||||||||||||||:||

E.asinus 301 TNVINGRPQFSAAPMTLLYQCPGHGPLLPLAIQLSQTPGPNSPIFLPSDD 350

H.sapiens 350 KWDWLLAKTWVRNAEFSFHEALTHLLHSHLLPEVFTLATLRQLPHCHPLF 399

||||||||||||||:||.||||||||.:|||.|||.||||||||.|||||

E.asinus 351 KWDWLLAKTWVRNADFSVHEALTHLLQTHLLSEVFVLATLRQLPRCHPLF 400

H.sapiens 400 KLLIPHTRYTLHINTLARELLIVPGQVVDRSTGIGIEGFSELIQRNMKQL 449

|||:||||||||||||||||||.|.:|||||||:|:.||||||||:|:||

E.asinus 401 KLLVPHTRYTLHINTLARELLITPKRVVDRSTGLGLRGFSELIQRSMEQL 450

H.sapiens 450 NYSLLCLPEDIRTRGVEDIPGYYYRDDGMQIWGAVERFVSEIIGIYYPSD 499

|||:||||||||.|||||||||||||||||||||||.|||||||||||||

E.asinus 451 NYSVLCLPEDIRARGVEDIPGYYYRDDGMQIWGAVEHFVSEIIGIYYPSD 500

H.sapiens 500 ESVQDDRELQAWVREIFSKGFLNQESSGIPSSLETREALVQYVTMVIFTC 549

.||.:|.||||||.||||||||.:||||:|||||||||||||||||||.|

E.asinus 501 VSVCNDSELQAWVWEIFSKGFLGRESSGLPSSLETREALVQYVTMVIFNC 550

H.sapiens 550 SAKHAAVSAGQFDSCAWMPNLPPSMQLPPPTSKGLATCEGFIATLPPVNA 599

||||:||.:||||.||||||||||||||||||||.|:.||||||||||||

E.asinus 551 SAKHSAVGSGQFDYCAWMPNLPPSMQLPPPTSKGQASLEGFIATLPPVNA 600

H.sapiens 600 TCDVILALWLLSKEPGDQRPLGTYPDEHFTEEAPRRSIATFQSRLAQISR 649

:||||:|||||||||||||||||||:|||||||.|:|||.|||.|||||:

E.asinus 601 SCDVIIALWLLSKEPGDQRPLGTYPEEHFTEEASRQSIAAFQSHLAQISQ 650

H.sapiens 650 GIQERNQGLVLPYTYLDPPLIENSVSI 676

.|:.||||||||||||||||||:|:||

E.asinus 651 DIRVRNQGLVLPYTYLDPPLIESSISI 677

Przewalski's horse (*Equus przewalskii*)

H.sapiens 1 MAEFRVRVSTGEAFGAGTWDKVSVSIVGTRGESPPLPLDNLGKEFTAGAE 50

||::|||||||||||||||||:|::||||:||:|.||||:||||||.|||

E.przewalskii 1 MAKYRVRVSTGEAFGAGTWDKMSITIVGTQGETPRLPLDHLGKEFTVGAE 50

H.sapiens 51 EDFQVTLPEDVGRVLLLRVHKAPPVLP-LLGPLAPDAWFCRWFQLTPPRG 99

|||:|..|:|||.|||||||||||.|| |||||||||||||.||||||||

E.przewalskii 51 EDFEVKSPQDVGPVLLLRVHKAPPALPLLLGPLAPDAWFCRSFQLTPPRG 100

H.sapiens 100 GHLLFPCYQWLEGAGTLVLQEGTAKVSWADHHPVLQQQRQEELQARQEMY 149

..|.||.||||||||:|||:||.||||||||||.|||||:||||||||||

E.przewalskii 101 SLLRFPAYQWLEGAGSLVLREGAAKVSWADHHPKLQQQRREELQARQEMY 150

H.sapiens 150 QWKAYNPGWPHCLDEKTVEDLELNIKYSTAKNANFYLQAGSAFAEMKIKG 199

:||.|.||||||||.:||:||:||.|||..|:.|.||:|.|||||:||||

E.przewalskii 151 RWKTYQPGWPHCLDMETVKDLDLNTKYSVVKSTNLYLRAQSAFAELKIKG 200

H.sapiens 200 LLDRKGLWRSLNEMKRIFNFRRTPA------------------------- 224

||:|||.||||:||:.:.||::|||

E.przewalskii 201 LLERKGSWRSLSEMEMVLNFQKTPATGDKSGSRRTSYEALVVVSRGWWPQ 250

H.sapiens 225 -------------------------------------------------- 224

E.przewalskii 251 QSSDSRDGVDRWTCPQDGESVGCDEKRKRGPQALPQQPGGWRCHFPGCQA 300

H.sapiens 225 --------------------AEHAFEHWQEDAFFASQFLNGLNPVLIRRC 254

||:.|||||||||||||||||||||||.||

E.przewalskii 301 MGGAGLKEEPTFSLRYAAFEAEYVFEHWQEDAFFASQFLNGLNPVLIHRC 350

H.sapiens 255 HYLPKNFPVTDAMVASVLGPGTSLQAELEKGSLFLVDHGILSGIQTNVIN 304

..||:||||||||||.|||||||||||||:|||||||||||||::|||||

E.przewalskii 351 RRLPENFPVTDAMVAPVLGPGTSLQAELERGSLFLVDHGILSGVRTNVIN 400

H.sapiens 305 GKPQFSAAPMTLLYQSPGCGPLLPLAIQLSQTPGPNSPIFLPTDDKWDWL 354

|:|||||||||||||.||.|.|||||||||||||||||||||:|||||||

E.przewalskii 401 GRPQFSAAPMTLLYQCPGHGLLLPLAIQLSQTPGPNSPIFLPSDDKWDWL 450

H.sapiens 355 LAKTWVRNAEFSFHEALTHLLHSHLLPEVFTLATLRQLPHCHPLFKLLIP 404

|||||||||:||.|||||||||:|||.|||.|||||||||||||||||:|

E.przewalskii 451 LAKTWVRNADFSVHEALTHLLHTHLLSEVFVLATLRQLPHCHPLFKLLVP 500

H.sapiens 405 HTRYTLHINTLARELLIVPGQVVDRSTGIGIEGFSELIQRNMKQLNYSLL 454

|||||||||||||||||.|.:|||||||:|:.||||||||:|:|||||:|

E.przewalskii 501 HTRYTLHINTLARELLITPKRVVDRSTGLGLRGFSELIQRSMEQLNYSVL 550

H.sapiens 455 CLPEDIRTRGVEDIPGYYYRDDGMQIWGAVERFVSEIIGIYYPSDESVQD 504

|||||||.|||||||||||||||||||||||.|||||||||||||.||:|

E.przewalskii 551 CLPEDIRARGVEDIPGYYYRDDGMQIWGAVEHFVSEIIGIYYPSDVSVRD 600

H.sapiens 505 DRELQAWVREIFSKGFLNQESSGIPSSLETREALVQYVTMVIFTCSAKHA 554

|.||||||.||||||||.:||||:|||||||||||||||||||.|||||:

E.przewalskii 601 DSELQAWVWEIFSKGFLGRESSGLPSSLETREALVQYVTMVIFNCSAKHS 650

H.sapiens 555 AVSAGQFDSCAWMPNLPPSMQLPPPTSKGLATCEGFIATLPPVNATCDVI 604

||.:||||.||||||||||||||||||||.|:.||||||||||||:||||

E.przewalskii 651 AVGSGQFDYCAWMPNLPPSMQLPPPTSKGQASLEGFIATLPPVNASCDVI 700

H.sapiens 605 LALWLLSKEPGDQRPLGTYPDEHFTEEAPRRSIATFQSRLAQISRGIQER 654

:|||||||||||||||||||:|||||||.|:|||.|||||||||:.|:.|

E.przewalskii 701 IALWLLSKEPGDQRPLGTYPEEHFTEEASRQSIAAFQSRLAQISQDIRVR 750

H.sapiens 655 NQGLVLPYTYLDPPLIENSVSI 676

|||||||||||||||||:|:||

E.przewalskii 751 NQGLVLPYTYLDPPLIESSISI 772

Cattle (*Bos taurus*)

H.sapiens 1 MAEFRVRVSTGEAFGAGTWDKVSVSIVGTRGESPPLPLDNLGKEFTAGAE 50

||:||||||||||||||||||:||||||||||:||||||.|||||.||||

B.taurus 1 MAKFRVRVSTGEAFGAGTWDKISVSIVGTRGETPPLPLDRLGKEFNAGAE 50

H.sapiens 51 EDFQVTLPEDVGRVLLLRVHKAPPVLPL-LGPLAPDAWFCRWFQLTPPRG 99

|||:||.|||||||||:|||||||.... |.|.|.|||||||.||||...

B.taurus 51 EDFEVTPPEDVGRVLLVRVHKAPPARSRPLAPSAGDAWFCRWLQLTPHGA 100

H.sapiens 100 GHLLFPCYQWLEGAGTLVLQEGTAKVSWADHHPVLQQQRQEELQARQEMY 149

..|.|||||||||..:|||:|||||:||.||||.|||||:||||||:|.|

B.taurus 101 APLCFPCYQWLEGERSLVLREGTAKISWEDHHPTLQQQRREELQARRETY 150

H.sapiens 150 QWKAYNPGWPHCLDEKTVEDLELNIKYSTAKNANFYLQAGSAFAEMKIKG 199

:||.|.||||.||||:||::|.||||||.|||..|||:..||.||:|:||

B.taurus 151 RWKTYIPGWPRCLDEETVKNLNLNIKYSVAKNTTFYLRGSSALAELKLKG 200

H.sapiens 200 LLDRKGLWRSLNEMKRIFNFRRTPAAEHAFEHWQEDAFFASQFLNGLNPV 249

||||||||:||.||:|:||||:|||.|:..||||||||||.|||||||||

B.taurus 201 LLDRKGLWKSLKEMRRVFNFRKTPAVEYVCEHWQEDAFFAYQFLNGLNPV 250

H.sapiens 250 LIRRCHYLPKNFPVTDAMVASVLGPGTSLQAELEKGSLFLVDHGILSGIQ 299

||||||:|||||||||||||.|||||||||||||||||||||:.:|:|::

B.taurus 251 LIRRCHHLPKNFPVTDAMVAPVLGPGTSLQAELEKGSLFLVDYDLLAGVR 300

H.sapiens 300 TNVINGKPQFSAAPMTLLYQSPGCGPLLPLAIQLSQTPGPNSPIFLPTDD 349

||||||:||||.||||||||.||.|||||||||||||||||||||||:||

B.taurus 301 TNVINGRPQFSTAPMTLLYQRPGRGPLLPLAIQLSQTPGPNSPIFLPSDD 350

H.sapiens 350 KWDWLLAKTWVRNAEFSFHEALTHLLHSHLLPEVFTLATLRQLPHCHPLF 399

|||||||||||||||||.||||||||.:||:||||.||||||||||||||

B.taurus 351 KWDWLLAKTWVRNAEFSIHEALTHLLQAHLVPEVFALATLRQLPHCHPLF 400

H.sapiens 400 KLLIPHTRYTLHINTLARELLIVPGQVVDRSTGIGIEGFSELIQRNMKQL 449

|||||||||||||||||||.||.||||||||||:||.|||||||:|||||

B.taurus 401 KLLIPHTRYTLHINTLARERLIAPGQVVDRSTGLGIGGFSELIQKNMKQL 450

H.sapiens 450 NYSLLCLPEDIRTRGVEDIPGYYYRDDGMQIWGAVERFVSEIIGIYYPSD 499

|||.||||:|||.|||||||.||||||||||||||||||||:||||||||

B.taurus 451 NYSALCLPDDIRARGVEDIPDYYYRDDGMQIWGAVERFVSEMIGIYYPSD 500

H.sapiens 500 ESVQDDRELQAWVREIFSKGFLNQESSGIPSSLETREALVQYVTMVIFTC 549

|||:||.|||||||||||:|||.:||||:||:|.|||||:||||||||.|

B.taurus 501 ESVRDDSELQAWVREIFSEGFLGRESSGLPSTLGTREALIQYVTMVIFNC 550

H.sapiens 550 SAKHAAVSAGQFDSCAWMPNLPPSMQLPPPTSKGLATCEGFIATLPPVNA 599

||||:||||||||..|||||||||||||||||||.|..|||:||||||||

B.taurus 551 SAKHSAVSAGQFDFAAWMPNLPPSMQLPPPTSKGQARLEGFLATLPPVNA 600

H.sapiens 600 TCDVILALWLLSKEPGDQRPLGTYPDEHFTEEAPRRSIATFQSRLAQISR 649

||||::|||||||||||:|||||||:|:|||||||||||.||||||:|||

B.taurus 601 TCDVVIALWLLSKEPGDRRPLGTYPEEYFTEEAPRRSIAAFQSRLAEISR 650

H.sapiens 650 GIQERNQGLVLPYTYLDPPLIENSVSI 676

.|||||..|.|||.|||||||||||||

B.taurus 651 DIQERNHSLALPYPYLDPPLIENSVSI 677

Bactrian camel (*Camelus ferus*)

H.sapiens 1 MA--EFRVRVSTGEAFGAGTWDKVSVSIVGTRGESPPLPLDNLGKEFTAG 48

|| ..|||||.|||||||||:|:|||||||:||:||||||:||||||||

C.ferus 1 MAGLRVRVRVSMGEAFGAGTWNKISVSIVGTQGETPPLPLDHLGKEFTAG 50

H.sapiens 49 AEEDFQVTLPEDVGRVLLLRVHKAPPVLPL-LGPLAPDAWFCRWFQLTPP 97

|||||:||||:|||.||||||||.|..||. ||.||.||||||||||..|

C.ferus 51 AEEDFEVTLPQDVGSVLLLRVHKTPLALPCPLGSLAQDAWFCRWFQLMLP 100

H.sapiens 98 RGGHLLFPCYQWLEGAGTLVLQEGTAKVSWADHHPVLQQQRQEELQARQE 147

:|..|.|||||||| ||:|..||||.|||||:||||||:|

C.ferus 101 QGSPLRFPCYQWLE-----------AKISCEDHHPRLQQQREEELQARRE 139

H.sapiens 148 MYQWKAYNPGWPHCLDEKTVEDLELNIKYSTAKNANFYLQAGSAFAEMKI 197

||:||.|||||||||||:||::|.||||||..|.||||||.||..||:|:

C.ferus 140 MYRWKTYNPGWPHCLDEETVKNLNLNIKYSVTKKANFYLQGGSLLAELKL 189

H.sapiens 198 KGLLDRKGLWRSLNEMKRIFNFRRTPAAEHAFEHWQEDAFFASQFLNGLN 247

|||...||||:||.||.::|:|.:|||..:.|||.|||||||.||||||:

C.ferus 190 KGLWHLKGLWKSLKEMTKVFSFWKTPAVGYVFEHXQEDAFFAYQFLNGLS 239

H.sapiens 248 PVLIRRCHYLPKNFPVTDAMVASVLGPGTSLQAELEKGSLFLVDHGILSG 297

||||:||..|||||||||||||.:|||||||||||||||:||||||||||

C.ferus 240 PVLIQRCRCLPKNFPVTDAMVAPMLGPGTSLQAELEKGSVFLVDHGILSG 289

H.sapiens 298 IQTNVINGKPQFSAAPMTLLYQSPGCGPLLPLAIQLSQTPGPNSPIFLPT 347

::||||||:|||||||||||||.|||||||||||||||||||:|||||||

C.ferus 290 VRTNVINGRPQFSAAPMTLLYQWPGCGPLLPLAIQLSQTPGPDSPIFLPT 339

H.sapiens 348 DDKWDWLLAKTWVRNAEFSFHEALTHLLHSHLLPEVFTLATLRQLPHCHP 397

||||||||||||||||||:|||||.|||..||:.||||||.|||||||||

C.ferus 340 DDKWDWLLAKTWVRNAEFTFHEALAHLLMGHLIAEVFTLALLRQLPHCHP 389

H.sapiens 398 LFKLLIPHTRYTLHINTLARELLIVPGQVVDRSTGIGIEGFSELIQRNMK 447

|||||||||||||:|:||.|||||..|:|||||||:|:||.||||||:|:

C.ferus 390 LFKLLIPHTRYTLYISTLGRELLIASGKVVDRSTGLGLEGLSELIQRHME 439

H.sapiens 448 QLNYSLLCLPEDIRTRGVEDIPGYYYRDDGMQIWGAVERFVSEIIGIYYP 497

:||||.||||||||.|||||||||||:||||:||.||||||||||..|||

C.ferus 440 ELNYSALCLPEDIRARGVEDIPGYYYQDDGMKIWDAVERFVSEIINNYYP 489

H.sapiens 498 SDESVQDDRELQAWVREIFSKGFLNQESSGIPSSLETREALVQYVTMVIF 547

|||||:||.||||||.||||:|||.:||||:||||.|||||:||||||||

C.ferus 490 SDESVRDDSELQAWVWEIFSEGFLGRESSGLPSSLRTREALIQYVTMVIF 539

H.sapiens 548 TCSAKHAAVSAGQFDSCAWMPNLPPSMQLPPPTSKGLATCEGFIATLPPV 597

.|||||:||||||||..|||||||||||||||||||.|..|||:||||||

C.ferus 540 NCSAKHSAVSAGQFDFGAWMPNLPPSMQLPPPTSKGQAKLEGFLATLPPV 589

H.sapiens 598 NATCDVILALWLLSKEPGDQRPLGTYPDEHFTEEAPRRSIATFQSRLAQI 647

|||||:|:..|:||||||||||||||||:||||||||||||.||:.||||

C.ferus 590 NATCDMIIIFWMLSKEPGDQRPLGTYPDKHFTEEAPRRSIAAFQNHLAQI 639

H.sapiens 648 SRGIQERNQGLVLPYTYLDPPLIENSVSI 676

||.|:||||||.||||||||||||||:||

C.ferus 640 SRDIRERNQGLELPYTYLDPPLIENSISI 668

Dromedary (*Camelus dromedarius*)

H.sapiens 1 MA--EFRVRVSTGEAFGAGTWDKVSVSIVGTRGESPPLPLDNLGKEFTAG 48

|| ..|||||.|||||||||:|:|||||||:||:||||||:||||||||

C.dromedarius 1 MAGLRVRVRVSMGEAFGAGTWNKISVSIVGTQGETPPLPLDHLGKEFTAG 50

H.sapiens 49 AEEDFQVTLPEDVGRVLLLRVHKAPPVLPL-LGPLAPDAWFCRWFQLTPP 97

|||||:||||:|||.||||||||.|..||. ||.||.||||||||||..|

C.dromedarius 51 AEEDFEVTLPQDVGSVLLLRVHKTPLALPCPLGSLAQDAWFCRWFQLMLP 100

H.sapiens 98 RGGHLLFPCYQWLEGAGTLVLQEGTAKVSWADHHPVLQQQRQEELQARQE 147

:|..|.|||||||||..:|||:|||||:|..||||.|||||:||||||:|

C.dromedarius 101 QGSPLRFPCYQWLEGEESLVLREGTAKISCEDHHPRLQQQREEELQARRE 150

H.sapiens 148 MYQWKAYNPGWPHCLDEKTVEDLELNIKYSTAKNANFYLQAGSAFAEMKI 197

||:||.|||||||||||:||::|.||||||..|.||||||.||..||:|:

C.dromedarius 151 MYRWKTYNPGWPHCLDEETVKNLNLNIKYSVTKKANFYLQGGSLLAELKL 200

H.sapiens 198 KGLLDRKGLWRSLNEMKRIFNFRRTPAAEHAFEHWQEDAFFASQFLNGLN 247

|||..|||||:||.||.::|:||:|||..:.|||||||||||.||||||:

C.dromedarius 201 KGLWHRKGLWKSLKEMTKVFSFRKTPAVGYVFEHWQEDAFFAYQFLNGLS 250

H.sapiens 248 PVLIRRCHYLPKNFPVTDAMVASVLGPGTSLQAELEKGSLFLVDHGILSG 297

||||:||..|||||||||||||.:|||||||||||||||:||||||||||

C.dromedarius 251 PVLIQRCCCLPKNFPVTDAMVAPMLGPGTSLQAELEKGSVFLVDHGILSG 300

H.sapiens 298 IQTNVINGKPQFSAAPMTLLYQSPGCGPLLPLAIQLSQTPGPNSPIFLPT 347

:.||||||:.||||||||||||.|||||||||||||||||||:|||||||

C.dromedarius 301 VHTNVINGRSQFSAAPMTLLYQRPGCGPLLPLAIQLSQTPGPDSPIFLPT 350

H.sapiens 348 DDKWDWLLAKTWVRNAEFSFHEALTHLLHSHLLPEVFTLATLRQLPHCHP 397

||| |||||||||.||||:|||||||||..||:.||||||.|||||||||

C.dromedarius 351 DDK-DWLLAKTWVHNAEFTFHEALTHLLMGHLIAEVFTLALLRQLPHCHP 399

H.sapiens 398 LFKLLIPHTRYTLHINTLARELLIVPGQVVDRSTGIGIEGFSELIQRNMK 447

|||||||||||||:|:||.|.|||..|:|||||||:|:||.||||||:|:

C.dromedarius 400 LFKLLIPHTRYTLYISTLGRVLLIASGKVVDRSTGLGLEGLSELIQRHME 449

H.sapiens 448 QLNYSLLCLPEDIRTRGVEDIPGYYYRDDGMQIWGAVERFVSEIIGIYYP 497

:||||.|.||||||.||||||||||||||||:||.||||||||||..|||

C.dromedarius 450 ELNYSALYLPEDIRARGVEDIPGYYYRDDGMKIWDAVERFVSEIINNYYP 499

H.sapiens 498 SDESVQDDRELQAWVREIFSKGFLNQESSGIPSSLETREALVQYVTMVIF 547

|||||.||.||||||.||||:|||.:||||:||||.|||||:||||||||

C.dromedarius 500 SDESVHDDSELQAWVWEIFSEGFLGRESSGLPSSLRTREALIQYVTMVIF 549

H.sapiens 548 TCSAKHAAVSAGQFDSCAWMPNLPPSMQLPPPTSKGLATCEGFIATLPPV 597

.|||||:||||||||..|||||||||||||||||||.|..|||:||||||

C.dromedarius 550 NCSAKHSAVSAGQFDFGAWMPNLPPSMQLPPPTSKGQAKLEGFLATLPPV 599

H.sapiens 598 NATCDVILALWLLSKEPGDQRPLGTYPDEHFTEEAPRRSIATFQSRLAQI 647

|||||:|:..|:|||||||||||||||||||||||||||||.||:.||||

C.dromedarius 600 NATCDMIIIFWMLSKEPGDQRPLGTYPDEHFTEEAPRRSIAAFQNHLAQI 649

H.sapiens 648 SRGIQERNQGLVLPYTYLDPPLIENSVSI 676

||.|:||||||.||||||||||||||:||

C.dromedarius 650 SRDIRERNQGLELPYTYLDPPLIENSISI 678

Meerkat (*Suricata suricatta*)

H.sapiens 1 MAEFRVRVSTGEAFGAGTWDKVSVSIVGTRGESPPLPLDNLGKEFTAGAE 50

|||:||.||||||.|||||::::|||||||||:||||||:.||||:.||.

S.suricatta 1 MAEYRVTVSTGEAIGAGTWNRIAVSIVGTRGETPPLPLDHFGKEFSKGAV 50

H.sapiens 51 EDFQVTLPEDVGRVLLLRVHKAPPVLPLLGPLAPDAWFCRWFQLTPPRGG 100

|||||..|:|||||||||:||.|.:...:.|||.|||||.|.|||||||.

S.suricatta 51 EDFQVAAPQDVGRVLLLRLHKEPLLPSAVRPLARDAWFCNWVQLTPPRGA 100

H.sapiens 101 HLLFPCYQWLEGAGTLVLQEGTAKVSWADHHPVLQQQRQEE------LQA 144

.|.||||:|:||.|:|.|:||.|||||||.|||||||..:. |.:

S.suricatta 101 PLRFPCYRWMEGPGSLTLREGAAKVSWADDHPVLQQQPPDASPWDAFLNS 150

H.sapiens 145 RQEMYQ---WKAYNPGWPHCLDEKTVEDLELNIKYSTAKNANFYLQAGSA 191

...|.. ||.|.||||.|||||||:||:||||||..|||||||:.|||

S.suricatta 151 LLTMAAGPLWKTYLPGWPRCLDEKTVKDLDLNIKYSVTKNANFYLKMGSA 200

H.sapiens 192 FAEMKIKGLLDRKGLWRSLNEMKRIFNFRRTPAAEHAFEHWQEDAFFASQ 241

|.|||:||||||||||:||:||||:|:||::||||:.:||||||||||||

S.suricatta 201 FTEMKLKGLLDRKGLWKSLDEMKRMFSFRKSPAAEYMYEHWQEDAFFASQ 250

H.sapiens 242 FLNGLNPVLIRRCHYLPKNFPVTDAMVASVLGPGTSLQAELEKGSLFLVD 291

|||||||||||.|..||:||||||.|||.|||||||||||||:|||||||

S.suricatta 251 FLNGLNPVLIRHCRSLPENFPVTDDMVALVLGPGTSLQAELERGSLFLVD 300

H.sapiens 292 HGILSGIQTNVINGKPQFSAAPMTLLYQSPGCGPLLPLAIQLSQTPGPNS 341

||||||:.||||||||||||||||||||.||.||||||||||||||||.|

S.suricatta 301 HGILSGVSTNVINGKPQFSAAPMTLLYQHPGGGPLLPLAIQLSQTPGPTS 350

H.sapiens 342 PIFLPTDDKWDWLLAKTWVRNAEFSFHEALTHLLHSHLLPEVFTLATLRQ 391

|||||||::|||||||||||||||||.|||||||.:||||||||:|||||

S.suricatta 351 PIFLPTDNEWDWLLAKTWVRNAEFSFSEALTHLLQAHLLPEVFTMATLRQ 400

H.sapiens 392 LPHCHPLFKLLIPHTRYTLHINTLARELLIVPGQVVDRSTGIGIEGFSEL 441

||.|||||||||.||:||||||||.|||||.|||||:||||||::|||||

S.suricatta 401 LPPCHPLFKLLISHTQYTLHINTLGRELLITPGQVVERSTGIGLQGFSEL 450

H.sapiens 442 IQRNMKQLNYSLLCLPEDIRTRGVEDIPGYYYRDDGMQIWGAVERFVSEI 491

|||:|:.|:|:.||||||||.|||||||||||||||:|||.|||.|||||

S.suricatta 451 IQRHMETLSYTTLCLPEDIRARGVEDIPGYYYRDDGLQIWDAVESFVSEI 500

H.sapiens 492 IGIYYPSDESVQDDRELQAWVREIFSKGFLNQESSGIPSSLETREALVQY 541

|.||||||.||:||.||||||.||||:|||::||||:||||:||::||||

S.suricatta 501 ISIYYPSDASVRDDSELQAWVWEIFSEGFLSRESSGMPSSLDTRKSLVQY 550

H.sapiens 542 VTMVIFTCSAKHAAVSAGQFDSCAWMPNLPPSMQLPPPTSKGLATCEGFI 591

||||||.|||||.||||||||||.||||||.:||||||||||....|||:

S.suricatta 551 VTMVIFNCSAKHHAVSAGQFDSCVWMPNLPSTMQLPPPTSKGRTDPEGFV 600

H.sapiens 592 ATLPPVNATCDVILALWLLSKEPGDQRPLGTYPDEHFTEEAPRRSIATFQ 641

|.|||||.|||:.:|||||||||||.|||||||.||||||||:|||..||

S.suricatta 601 AALPPVNVTCDIAIALWLLSKEPGDHRPLGTYPQEHFTEEAPQRSITAFQ 650

H.sapiens 642 SRLAQISRGIQERNQGLVLPYTYLDPPLIENSVSI 676

:||||||:.|::||..|.:||||||||||||||||

S.suricatta 651 NRLAQISKDIRKRNMKLAVPYTYLDPPLIENSVSI 685

Ferret (*Mustela putorius furo*)

H.sapiens 1 MAEFRVRVSTGEAFGAGTWDKVSVSIVGTRGESPPLPLDNLGKEFTAGAE 50

||||.||||||:|.|||||:|::|||||||||:|||.||:.||||:||..

M.putorius furo 1 MAEFSVRVSTGDAIGAGTWNKIAVSIVGTRGETPPLRLDHPGKEFSAGTV 50

H.sapiens 51 EDFQVTLPEDVGRVLLLRVHKAPPVLPL-LGPLAPDAWFCRWFQLTPPRG 99

|||||..|:|||.||||||||||.:|.. :|.:|.|||||||.||:.|||

M.putorius furo 51 EDFQVESPQDVGPVLLLRVHKAPLLLSAPVGAVARDAWFCRWIQLSQPRG 100

H.sapiens 100 GHLLFPCYQWLEGAGTLVLQEGTAKVSWADHHPVLQQQRQEELQARQEMY 149

..|.||||||||||.:|.|:.|.|||||||.||:|:|||||||||||:.|

M.putorius furo 101 APLRFPCYQWLEGAESLALRAGAAKVSWADDHPILRQQRQEELQARQDSY 150

H.sapiens 150 QWKAYNPGWPHCLDEKTVEDLELNIKYSTAKNANFYLQAGSAFAEMKIKG 199

.||.|:||||.|||||:|:||:||||||..||:.|||:.||||.|:|:||

M.putorius furo 151 HWKTYSPGWPRCLDEKSVKDLDLNIKYSVKKNSCFYLKVGSAFTELKLKG 200

H.sapiens 200 LLDRKGLWRSLNEMKRIFNFRRTPAAEHAFEHWQEDAFFASQFLNGLNPV 249

||||||||:|||||:.:.|||.|||||:..:|||||||||||||||||||

M.putorius furo 201 LLDRKGLWKSLNEMRMMLNFRMTPAAEYVVKHWQEDAFFASQFLNGLNPV 250

H.sapiens 250 LIRRCHYLPKNFPVTDAMVASVLGPGTSLQAELEKGSLFLVDHGILSGIQ 299

|||||.:||:||||||.|||.:||||||||||||:||||||||||||.:.

M.putorius furo 251 LIRRCRHLPENFPVTDDMVAPMLGPGTSLQAELERGSLFLVDHGILSSVH 300

H.sapiens 300 TNVINGKPQFSAAPMTLLYQSPGCGPLLPLAIQLSQTPGPNSPIFLPTDD 349

|||:||||||||||||||||.||.||||||||||||:|||:||||||:|:

M.putorius furo 301 TNVVNGKPQFSAAPMTLLYQRPGGGPLLPLAIQLSQSPGPDSPIFLPSDN 350

H.sapiens 350 KWDWLLAKTWVRNAEFSFHEALTHLLHSHLLPEVFTLATLRQLPHCHPLF 399

|||||||||||||||||.|||:|||||:||||||||:|||||||.|||||

M.putorius furo 351 KWDWLLAKTWVRNAEFSVHEAVTHLLHAHLLPEVFTMATLRQLPQCHPLF 400

H.sapiens 400 KLLIPHTRYTLHINTLARELLIVPGQVVDRSTGIGIEGFSELIQRNMKQL 449

||||||||||||||||||||||.||||||||||||:.||||||||||:||

M.putorius furo 401 KLLIPHTRYTLHINTLARELLIAPGQVVDRSTGIGVRGFSELIQRNMQQL 450

H.sapiens 450 NYSLLCLPEDIRTRGVEDIPGYYYRDDGMQIWGAVERFVSEIIGIYYPSD 499

:||:||||||||.|||||||.|||||||::||||||.||||||.||||||

M.putorius furo 451 SYSVLCLPEDIRARGVEDIPNYYYRDDGLKIWGAVESFVSEIIHIYYPSD 500

H.sapiens 500 ESVQDDRELQAWVREIFSKGFLNQESSGIPSSLETREALVQYVTMVIFTC 549

.:||||.|||||||||||:|||.::|||:||:||||||||||||||||..

M.putorius furo 501 TAVQDDPELQAWVREIFSEGFLGRKSSGVPSALETREALVQYVTMVIFNS 550

H.sapiens 550 SAKHAAVSAGQFDSCAWMPNLPPSMQLPPPTSKGLATCEGFIATLPPVNA 599

||||.||||||||||.|||||||:||||||||||....|||:||||||||

M.putorius furo 551 SAKHYAVSAGQFDSCIWMPNLPPTMQLPPPTSKGQTRPEGFLATLPPVNA 600

H.sapiens 600 TCDVILALWLLSKEPGDQRPLGTYPDEHFTEEAPRRSIATFQSRLAQISR 649

|||:|:||||||:||||:|||||||||||||||||||||.||:|||||||

M.putorius furo 601 TCDIIIALWLLSQEPGDRRPLGTYPDEHFTEEAPRRSIAAFQNRLAQISR 650

H.sapiens 650 GIQERNQGLVLPYTYLDPPLIENSVSI 676

.|:||||||.|||||||||||||||||

M.putorius furo 651 EIRERNQGLELPYTYLDPPLIENSVSI 677

Stoat (*Mustela erminea*)

H.sapiens 1 MAEFRVRVSTGEAFGAGTWDKVSVSIVGTRGESPPLPLDNLGKEFTAGAE 50

||||.||||||:|.|||||:|::|||||||||:|||.||:.||||:||..

M.erminea 1 MAEFSVRVSTGDAIGAGTWNKIAVSIVGTRGETPPLRLDHPGKEFSAGTV 50

H.sapiens 51 EDFQVTLPEDVGRVLLLRVHKAPPVLPL-LGPLAPDAWFCRWFQLTPPRG 99

|||||..|:|||.||||||||||.:|.. :|.:|.|||||||.||:.|||

M.erminea 51 EDFQVESPQDVGPVLLLRVHKAPLLLSAPVGAVARDAWFCRWIQLSQPRG 100

H.sapiens 100 GHLLFPCYQWLEGAGTLVLQEGTAKVSWADHHPVLQQQRQEELQARQEMY 149

..|.||||||||||.::.|:.|.|||||||.||:|:|||||||||||:.|

M.erminea 101 APLRFPCYQWLEGAESVALRAGAAKVSWADDHPILRQQRQEELQARQDSY 150

H.sapiens 150 QWKAYNPGWPHCLDEKTVEDLELNIKYSTAKNANFYLQAGSAFAEMKIKG 199

.||.|:||||.|||||:|:||:||||||..||:.|||:.||||.|:|:||

M.erminea 151 HWKTYSPGWPRCLDEKSVKDLDLNIKYSVKKNSCFYLKVGSAFTELKLKG 200

H.sapiens 200 LLDRKGLWRSLNEMKRIFNFRRTPAAEHAFEHWQEDAFFASQFLNGLNPV 249

||||||||:||:||:.:.|||.|||||:..:|||||||||||||||||||

M.erminea 201 LLDRKGLWKSLSEMRTMLNFRMTPAAEYVVKHWQEDAFFASQFLNGLNPV 250

H.sapiens 250 LIRRCHYLPKNFPVTDAMVASVLGPGTSLQAELEKGSLFLVDHGILSGIQ 299

|||||.:||:||||||.|||.:||||||||||||:||||||||||||.:.

M.erminea 251 LIRRCRHLPENFPVTDDMVAPMLGPGTSLQAELERGSLFLVDHGILSSVH 300

H.sapiens 300 TNVINGKPQFSAAPMTLLYQSPGCGPLLPLAIQLSQTPGPNSPIFLPTDD 349

|||:||||||||||||||||.||.|||||||||||::|||:||||||:|:

M.erminea 301 TNVVNGKPQFSAAPMTLLYQRPGGGPLLPLAIQLSRSPGPDSPIFLPSDN 350

H.sapiens 350 KWDWLLAKTWVRNAEFSFHEALTHLLHSHLLPEVFTLATLRQLPHCHPLF 399

|||||||||||||||||.|||:|||||:||||||||:|||||||.|||||

M.erminea 351 KWDWLLAKTWVRNAEFSVHEAVTHLLHAHLLPEVFTMATLRQLPQCHPLF 400

H.sapiens 400 KLLIPHTRYTLHINTLARELLIVPGQVVDRSTGIGIEGFSELIQRNMKQL 449

||||||||||||||||||||||.||||||||||||:.||||||||||:||

M.erminea 401 KLLIPHTRYTLHINTLARELLIAPGQVVDRSTGIGVRGFSELIQRNMQQL 450

H.sapiens 450 NYSLLCLPEDIRTRGVEDIPGYYYRDDGMQIWGAVERFVSEIIGIYYPSD 499

:||:||||||||.|||||||.|||||||::||||||.||||||.||||||

M.erminea 451 SYSVLCLPEDIRARGVEDIPNYYYRDDGLKIWGAVESFVSEIIHIYYPSD 500

H.sapiens 500 ESVQDDRELQAWVREIFSKGFLNQESSGIPSSLETREALVQYVTMVIFTC 549

.:||||.|||||||||||:|||.:||||:||:||||||||||||||||..

M.erminea 501 TAVQDDPELQAWVREIFSEGFLGRESSGVPSALETREALVQYVTMVIFNS 550

H.sapiens 550 SAKHAAVSAGQFDSCAWMPNLPPSMQLPPPTSKGLATCEGFIATLPPVNA 599

||||.||||||||||.|||||||:||||||||||....|||:||||||||

M.erminea 551 SAKHYAVSAGQFDSCIWMPNLPPTMQLPPPTSKGQTRPEGFLATLPPVNA 600

H.sapiens 600 TCDVILALWLLSKEPGDQRPLGTYPDEHFTEEAPRRSIATFQSRLAQISR 649

|||:|:||||||:||||::||||||||||||||||||||.||:|||||||

M.erminea 601 TCDIIIALWLLSQEPGDRQPLGTYPDEHFTEEAPRRSIAAFQNRLAQISR 650

H.sapiens 650 GIQERNQGLVLPYTYLDPPLIENSVSI 676

.|:||||||.|||||||||||||||||

M.erminea 651 EIRERNQGLELPYTYLDPPLIENSVSI 677
